# Network pharmacology and molecular docking reveal multi-target mechanisms of *Butea monosperma* stem bark extract in ulcerative colitis

**DOI:** 10.1038/s41598-025-24091-8

**Published:** 2025-11-17

**Authors:** Suman Mondal, Atreyee Ganguly, Arijit Nandi, Anwesha Das, Surya Mondal, Sourabh Mukherjee, Sharad D. Pawar, Yadu Nandan Dey

**Affiliations:** 1 Pharmacology Division, Dr. B.C. Roy College of Pharmacy and Allied Health Sciences, Durgapur, West Bengal 713206 India; 2 School of Pharmacy and Pharmaceutical Sciences, The University of Queensland, Woolloongabba, QLD 4102 Australia; 3 Pharmacology Division, Central Ayurveda Research Institute, Bidhannagar, Kolkata, West Bengal 700091 India

**Keywords:** Gene ontology, KEGG, Network pharmacology, Ulcerative colitis, Computational biology and bioinformatics, Cellular signalling networks, Gene ontology

## Abstract

Ulcerative colitis (UC) is a chronic inflammatory disorder primarily affecting the colonic mucosa and submucosa. *Butea monosperma* stem bark is traditionally used in Ayurveda for diarrhea, inflammatory diseases, and *Grahani Roga*, which shares similarities with UC. However, the molecular mechanisms of *B. monosperma* in UC are unexplored. This study aimed to predict the molecular mechanisms of *B. monosperma* in UC by integrating network pharmacology, molecular docking, and dynamics-the first systematic effort to decode its phytochemical-target-pathway interactions, bridging traditional knowledge with computational pharmacology. A standardized *B. monosperma* stem bark was prepared. LC-MS analysis was used to identify phytochemicals in the stem bark, and their potential targets, along with disease targets, were obtained from relevant databases. Protein-protein interaction, Gene Ontology, and KEGG pathway enrichment analysis were performed to identify key biological processes and signaling pathways modulated by stem bark in UC. Molecular docking and dynamics studies predicted interactions between phytoconstituents and key target proteins. The results suggest that stem bark of *B. monosperma* modulates pathways involved in cell migration, oxidative stress, and epithelial cell apoptosis, particularly *via* cancer-related pathways, IL-17 signaling, and Th17 cell differentiation, all of which are implicated in UC pathogenesis. Key target proteins, including MAPK1, AKT1, NF-κB, RELA, and MMP9, were predicted to interact with active compounds. These findings suggest a potential molecular basis for the therapeutic effects of *B. monosperma* stem bark in UC, warranting further experimental validation.

**Supplementary Information:**

The online version contains supplementary material available at 10.1038/s41598-025-24091-8.

## Introduction

Ulcerative Colitis (UC), a chronic intestinal inflammation affecting the colon and rectum with increasing prevalence in recent decades. Although the exact etiology of UC remains unclear, several primary factors contribute to the development of the disease such as genetic predisposition, intestinal immune dysfunction, and gut microbiome. Due to all these factors, the integrity of the mucosal barrier is disrupted leading to ulceration of colonic mucosa and internal bleeding resulting in symptoms such as bloody diarrhea, abdominal pain, and fecal urgency. The global burden of UC is higher than that of Crohn’s disease and the incidence of the cases is more common in developed and industrialized countries than in developing countries, which highlights the effect of the environment on the disease onset^1^. The prevalence of UC varies significantly across regions. In the United States, the National Health and Nutrition Examination Survey (NHANES) reported a prevalence of approximately 1%, affecting about 1.9 million adults^2^. Conversely, studies indicate a lower prevalence in India, highlighting regional disparities^3^. However, the adoption of western dietary habits and sedentary lifestyles may lead to a future increase in UC prevalence in India. While aminosalicylates, glucocorticoids, and immunomodulatory agents are commonly used to treat UC, their adverse effects can limit treatment efficacy^4^. Therefore, there is a need for safer alternative therapies.

*Butea monosperma* (Lam.) Kuntze (Family Fabaceae), is commonly known as Flames of Forest, Palasa, Purasu, Paras, Moduga, and Bastard Teak. It is a tree growing up to 12–15 m and is indigenous to India, Sri Lanka, Myanmar, Nepal, Bhutan, Bangladesh, Pakistan, China, Vietnam, and Thailand^5^. The plant is extensively described in Ayurvedic classics such as the Charaka Samhita, where it is recommended for conditions including diarrhoea, dysentery, intestinal worms, abdominal disorders, and inflammatory diseases^6^. Traditionally, the stem bark has been used in the management of *Grahani Roga*, a disorder with symptomatic resemblance to UC, as well as in other gastrointestinal conditions such as *Gulma* (abdominal tumors), *Arśa* (piles), *Vra*$\d {{\it n}}$*a* (ulcers), and *K*$\d {{\it r}}$*mīroga* (pinworm infection)^7,8^. Phytochemical investigations have revealed the presence of flavonoids, phenolics, and other bioactive compounds such as butin, butrin, gallic acid, catechin, medicarpin, lupeol, lupenone, mirosterol, quercetin, cajanin, cladrin, diadzein/diadzin, kaempferol, rhamnetin, isoformonoretin, buteaspermanol, ferulic acid, caffeic acid, morin, dimethyl quercetin, luteolin, sitosterol, ellagic acid, chlorogenic acid, umbelliferone, epicatchin and coumaric acid, many of which have reported anti-inflammatory and antioxidant activities^9,10^. Despite its traditional use in treating intestinal disorders and gastrointestinal inflammation in Ayurvedic system of medicine, the molecular mechanisms of *B. monosperma* stem bark in UC remain unexplored. This study represents the first systematic attempt to investigate the phytochemical–target–pathway interactions of *B. monosperma* in UC through an integrated approach involving network pharmacology, molecular docking, and molecular dynamics (MD) simulations. By combining LC-MS-based phytochemical profiling with computational analyses, it provides novel mechanistic insights into how *B. monosperma* stem bark may modulate key UC-associated signaling pathways. This integrative strategy not only supports traditional claims with in silico evidence but also identifies potential targets for experimental validation and therapeutic development.

## Materials and methods

### Collection and authentication of *B. monosperma* stem bark

In July 2024, the stem bark of *B. monosperma* was collected from a garden in Purulia, West Bengal, India. The collected material was subsequently identified and authenticated by Dr. Kanad Das, a Botanist at the Botanical Survey of India, Kolkata. A voucher specimen (BCRCP/SM-02) has been deposited in the herbarium. The sample was officially identified as *B. monosperma* (Fabaceae) stem bark. All experimental research and field studies involving plant material collection adhered to relevant institutional, national, and international guidelines and legislation.

### Standardization of *B. monosperma* stem bark

#### Physiochemical constants of *B. monosperma* stem bark

The physicochemical constants of *B. monosperma* stem bark were evaluated using previously described methods^11,12^. The total ash value was determined by incinerating 3 g of powdered bark at 450 °C in a muffle furnace until free from carbon, followed by cooling in a desiccator and weighing, with results expressed as percentage of ash relative to the air-dried sample. Acid-insoluble ash was quantified by boiling the total ash with 25 ml dilute HCl for 5 min, filtering the insoluble residue by ashless filter paper, washing, incinerating, and weighing to calculate the percentage of acid-insoluble ash. For water-soluble extractives, 5 g of coarsely powdered bark was macerated with 100 ml distilled water for 24 h (first 6 h with shaking, followed by 18 h standing), filtered, and 25 ml of the filtrate was evaporated to dryness at 105 °C in a preweighed Petri dish; the residue obtained was used to calculate the water-soluble extractive value (% w/w).

#### Fluorescence analysis of *B. monosperma* stem bark

The fluorescence analysis of *B. monosperma* stem bark was carried out as per the method describe previously^13^. The powder of the *B. monosperma* stem bark was spread evenly on a petriplate for observation under a UV chamber with adjustable wavelength settings at 254 nm (shortwave UV) and 365 nm (longwave UV). After allowing the UV lamps to stabilize, the sample was placed in the chamber with few drops of different chemicals such as conc. HCl, conc. H_2_SO_4_, conc. HNO_3_, 1% picric acid and 1 N NaOH and fluorescence intensity, colour, and uniformity were recorded.

#### Extraction and LC/MS analysis of *B. monosperma* stem bark

The powder of the *B. monosperma* stem bark, weighing approximately 500 mg, was extracted in 10 mL of n-hexane for 24 h using a soxhlet apparatus. After air drying, the sample was further extracted with 10 mL of methanol. The methanol extract (BME) was concentrated at 40 °C using a rotary evaporator, and the residue was reconstituted with 1 mL of methanol. This solution was centrifuged at 12,000 RPM for 6 min, and the supernatant layer was transferred into an LC vial for analysis. The analysis was performed using an Agilent LC-1200 Series coupled with an MS-6400 Series instrument. The injection volume was set to 5 µL. The mobile phase consisted of two components: A (5 mM ammonium formate in 0.1% formic acid, water-based) and B (0.1% formic acid in 100% methanol). A gradient elution program was applied, beginning with 95% A and 5% B, and progressing through several transitions to 5% A and 95% B before returning to the initial composition. Chromatography was conducted on an Agilent Eclipse C-18 column with specifications of 5 μm particle size, 15 cm length, and 4.6 mm inner diameter. The column temperature was maintained at 55 °C throughout the analysis. For the mass spectrometry analysis, the gas temperature was set to 225 °C with a gas flow rate of 6 L/min and a nebulizer pressure of 50 psi. The sheath gas temperature and flow rate were 300 °C and 12 mL/min, respectively. The capillary voltage was maintained at 2000 V, while the nozzle voltage was set to 500 V.

#### In silico studies

##### Identification of phytoconstituents and target of BME

The phytoconstituents were obtained from LC-ESI-MS/MS analysis of BME. Furthermore, additional data such as PubChem ID, CAS, canonical smiles, and 2D structures of phytoconstituents from PubChem (https://pubchem.ncbi.nlm.nih.gov/) database were collected. All active compounds were virtually screened for their ADME characteristics and they were examined using the web programs Molsoft (https://molsoft.com/mprop/) and SwissADME (http://www.swissadme.ch/index.php). The ADME criteria was applied using Lipinski’s rule of five, and compounds with ≥ 3 violations were not included in the study^14^. The phytoconstituents were utilized to identify different targets from the BindingDB (https://www.bindingdb.org/rwd/bind/index.jsp) and Swiss Target Prediction (https://www.swisstargetprediction.ch/). These webservers were screened for the targets for the phytoconstituents including only targets pertaining to ‘*Homo sapiens*’.

##### Identification of UC targets

The disease targets were identified and collected from DisGeNet (https://disgenet.com/*)* database and GeneCard (https://www.genecards.org/*)* by employing the terms “Ulcerative Colitis” and “*Homo sapiens*”. The targets obtained from various databases were checked for duplicates and after their elimination, the relevant targets for UC were selected for inclusion. After that, all the relevant targets were verified using UniProt ID, and their names were uniformed into statutory gene names using the UniProt database (https://www.uniprot.org/*).*

##### Convergence of bioactive targets and UC targets

Venny 2.1.0 (https://bioinfogp.cnb.csic.es/tools/venny/*)* was utilized to generate the intersected genes between phytoconstituents and the disease targets which resulted in the identification of common genes. The Venn diagram, thus generated, envisaged the common targets and employed it in constructing a protein-protein interaction network.

##### Construction of protein-protein interaction

The common targets were employed in String 11.0 (https://string-db.org/*)* to construct the protein-protein interaction (PPI). The minimum interaction threshold of high confidence > 0.7 and *Homo sapiens* were incorporated to create the PPI. To enhance the clarity of the nodes, Cytoscape 3.10.2 was implemented.

##### GO and KEGG enrichment analyses

To anticipate the various biological processes of GO, Shiny GO 0.86 was implemented, and to perform enrichment analysis KEGG (Kyoto Encyclopedia of Genes and Genomes) was utilized. The common targets of BME and UC were included in the study and the top 20 biological processes and pathways were included by setting the false discovery rate at 0.05 and selecting fold enrichment. The findings were reported in bubble charts depicting the gene counts, fold enrichment, and p-value. The GO analyses offered an understanding of different biological processes, cellular components, and molecular functions of the genes. Thus, all the insights into the proteins and the specific pathways involved are developed using online tools and assisted in shaping our understanding of the mechanism by which BME attempts to mitigate UC.

##### Molecular docking studies

Phytochemicals were analyzed in this study through LC-ESI-MS/MS profiling of BME. 37 compounds were selected for molecular docking with the Glide XP module of Schrödinger software (version 2017-2)^15–17^. Energy-optimized ligand conformations were docked with human MAPK1 X-ray structure (PDB code: 4FV1, resolution 1.99 Å), NF-κB X-ray structure (PDB code: 8TQD, resolution 2.02 Å), and MMP-9 X-ray structure (PDB code: 5CUH, resolution 1.83 Å) and AlphaFold-predicted structures of AKT serine/threonine kinase 1 (AKT1) (Gene: AKT1) and v-rel avian reticuloendotheliosis viral oncogene homolog A (RELA) (Gene: RELA) proteins^18^, with validated grid parameters. Prior to the docking protocol, the phytoconstituents and the proteins were prepared using LigPrep module^19^ and the Protein Preparation Wizard, respectively^20–22^. Notably, as the crystal structure of PDB ID 8TQD was found to be covalently linked with a co-crystallized covalent inhibitor ligand, so, at first, the inhibitor was then de-linked, and then the phytoconstituents were docked in the same space.

##### Induced fit docking (IFD)

IFD was performed on the top-scoring docking phytoconstituents against their respective receptors as follows: chlorogenic acid with MAPK1 [4FV1], chlorogenic acid with NF-κB [8TQD], leucocianidol with MMP-9 [5CUH], chlorogenic acid with AKT1 ((Gene: AKT1) (AlphaFold)), and riboflavin with RELA ((Gene: RELA) (AlphaFold)). The IFD module of Schrödinger (version 2017-2) was utilized in this analysis; the specifics of which protocol have been described in previous publications^23–25^. Of the standard and extended sampling protocols, the standard protocol was chosen. It produced up to twenty docked poses with default docking parameters and calculated OPLS3 force field IFD calculations^26,27^. The best-ranked conformations of the initial Glide XP docking of the compounds to their target receptors were taken as input for IFD. A grid box was built on the ligand centroid with no other constraints placed. Ligand ring conformations were sampled within a 2.5 kcal/mol energy window. The initial docking employed van der Waals scaling factors of 0.5 both for receptor and ligand. Subsequently, residues within 5 Å of the ligand were optimized employing Schrödinger’s Prime module. Thus, obtained receptor-ligand complexes in a window of 30 kcal/mol were then redocked employing Glide XP in its default mode.

##### MM-GBSA calculation

Free energy of binding was estimated by the MM-GBSA (molecular mechanics-generalized Born surface area) method, which combines molecular mechanics, the solvent-accessible surface area, and the generalized Born model to allow for free energy estimation without the heavy machinery of full-scale simulations^28^. MM-GBSA method was used to predict the energy difference between bound and unbound state of protein-ligand complex^29,30^. The binding free energies were calculated by the equation as follows: MM-GBSA ΔG_Bind_= G_Complex_ - (G_Receptor_ + G_Ligand_). Here, the Prime software was used for MM-GBSA calculation^29^ by the consideration of OPLS3 Force Field and the VSGB solvation model via minimized sampling.

##### MD simulations

MD simulations of the top-scoring protein–ligand docking complexes were performed using Schrödinger’s Desmond module^31–33^ under NPT ensemble conditions, maintaining a constant atmospheric pressure of 1.013 bar and a physiological temperature of 310 K for a total simulation time of 100 nanoseconds. Prior to initiating the production run, the system was prepared using the System Builder panel, which involved solvation of the complex in “SPC” model, addition of counterions to neutralize the system, and assignment of OPLS4 force field parameters. The simulation aimed to assess the dynamic stability and interaction profile of the complexes under near-physiological conditions. Post-simulation, the Simulation Interactions Diagram (SID) report was generated and analyzed to evaluate key molecular interactions, including hydrogen bonding, hydrophobic contacts, and conformational stability throughout the trajectory.

##### In silico physicochemical and ADME/T studies

Schrödinger suite’s (version 2017-2) QikPropmodule^34^ and Swiss ADME^35^ webserver were utilized to compare the physicochemical and ADME/T properties of these three compounds, to allow for determination of the most significant physicochemical descriptors and pharmacokinetically relevant properties.

## Result

### Standardization of *B. monosperma* stem bark

The ash values and extractive values of the *B. monosperma* stem bark powder was mentioned in (Supplementary Table S1). The values were found to be within pharmacopeial limits. The results of fluorescence analysis are depicted in (Supplementary Table S2).

### Phytochemical composition of BME and target identification

**Fig. 1:**
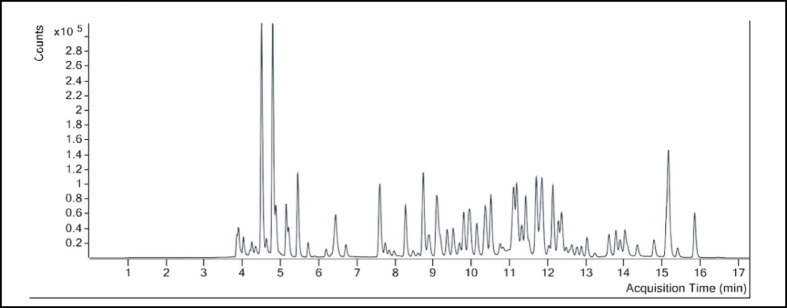
LC MS/MS analysis of BME.

The LC-MS chromatogram of the BME was represented in Fig. 1. Results from LC-MS analysis revealed the identification of 43 phytoconstituents in the extract after integrating with the libraries and interpretation of its MS/MS data. The details of the compounds are mentioned in (supplementary Table S3). Among the 43 identified phytoconstituents, 6 were excluded as they violated ≥ 3 Lipinski’s criteria (Supplementary Table S4). The remaining 37 met the drug-likeness requirements and were selected for target prediction. The PubChem CID of the compounds are mentioned in Supplementary Table S5. We obtained 256 targets by utilizing bindingDB (Supplementary Table S6) and 583 targets from SwissTargetPrediction (Supplementary Table S7) databases. Combining the targets from both databases and removing all the duplicates, 583 relevant targets for the 37 phytoconstituents were identified.

### The targets of UC

By using the term “UC”, the search for the targets was conducted in DisGeNet (Supplementary Table S8) and GeneCard (Supplementary Table S9). A total of 1141 relevant targets (Supplementary Table S10) were finally collected after combining and deleting the duplicates from the databases.

### Intersected targets and PPI network analysis

**Fig. 2:**
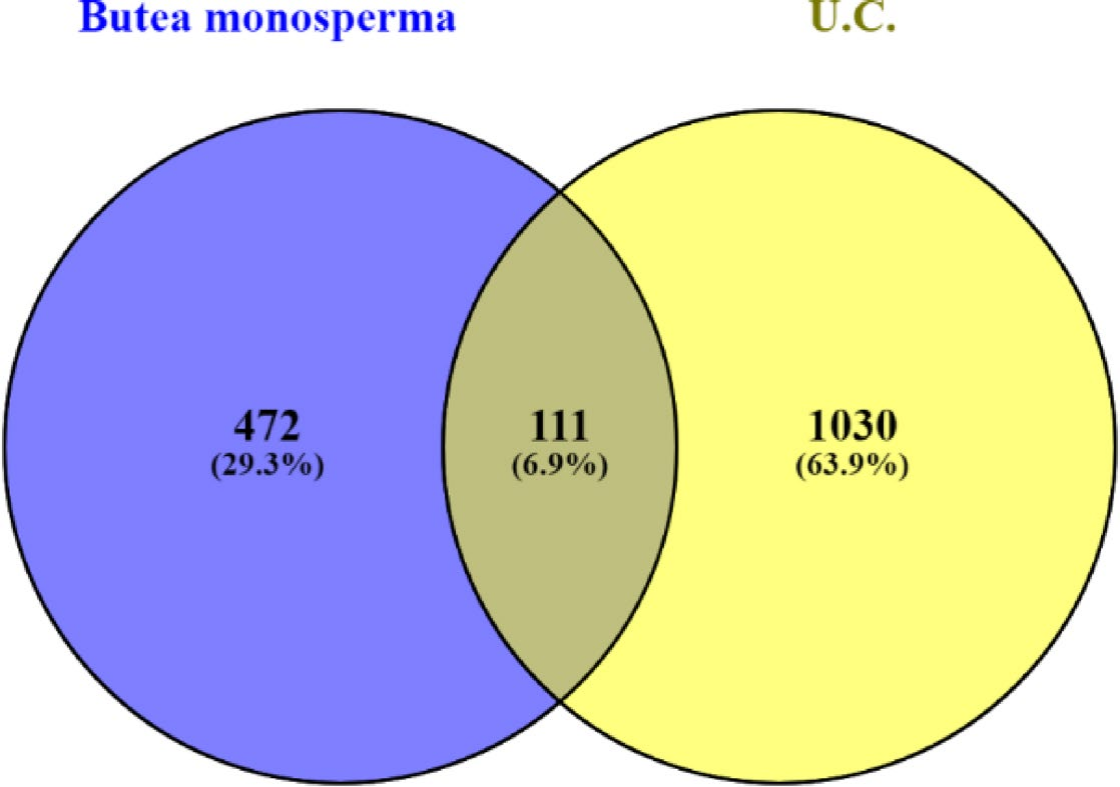
Common targets between phytoconstituents of BME and UC. The Venn diagram depicts the intersection of BME–associated targets (472) and UC-associated genes (1030), revealing 111 shared targets.

**Fig. 3:**
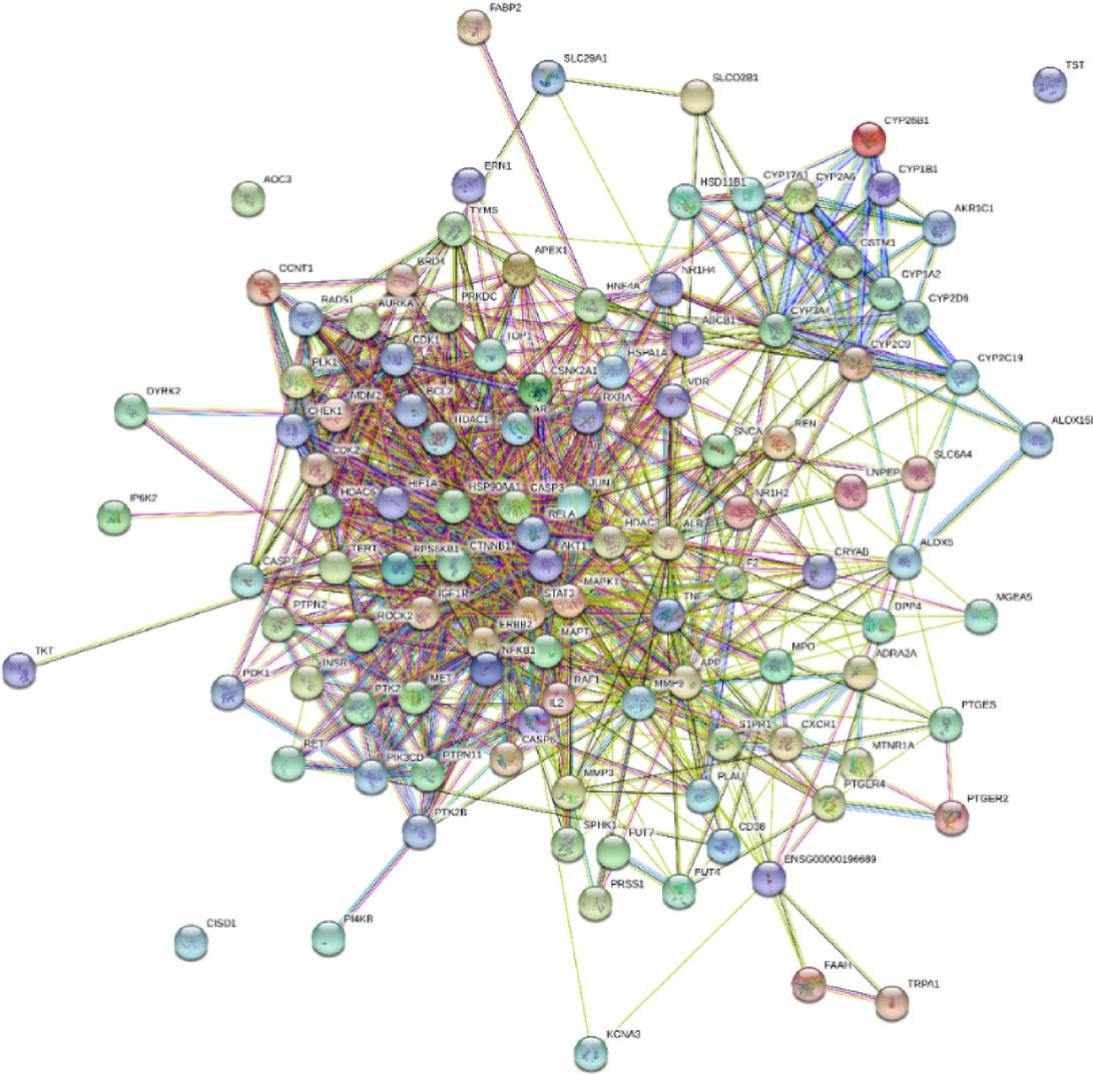
PPI network analysis of potential therapeutic targets for BME in UC. The protein–protein interaction (PPI) network of 111 intersection targets. Nodes represent target proteins, and edges indicate protein–protein associations.

**Fig. 4:**
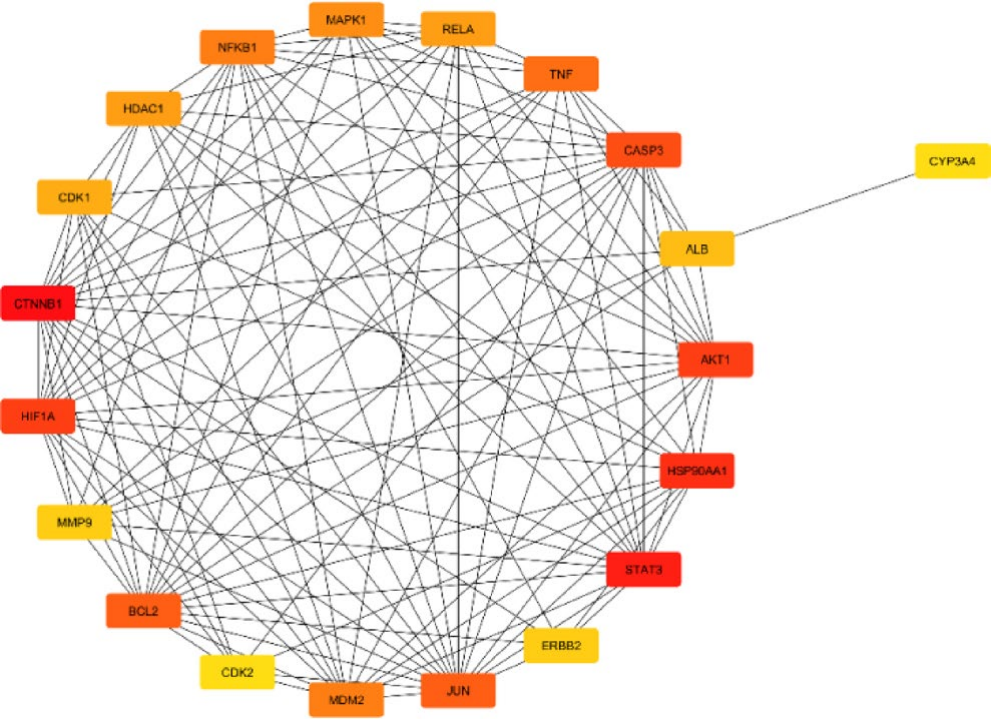
The top 20 hub genes in 111 intersection targets with cytohubba analysis. Nodes represent hub genes, with colour intensity reflecting their degree of connectivity in the network.

**Fig. 5:**
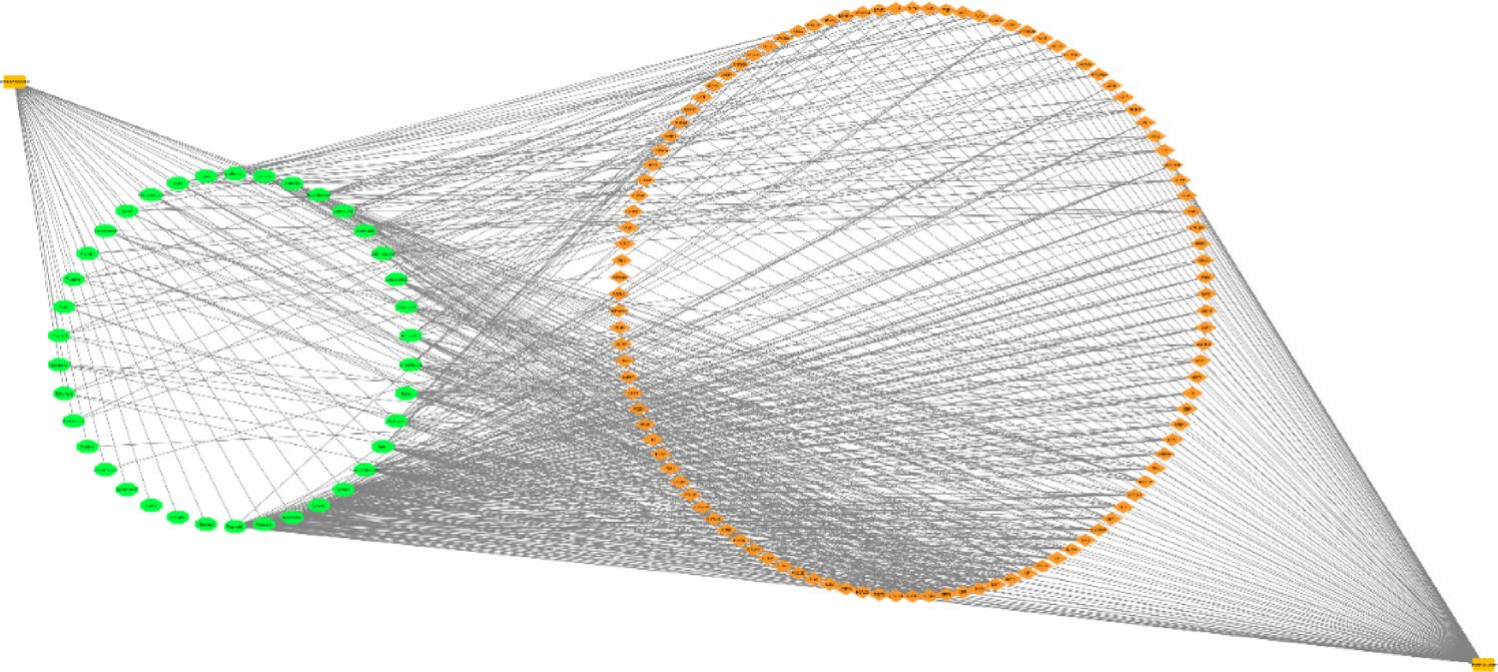
Network between phytoconstituents of BME (37 phytoconstituents) and common targets (111 targets).

37 bioactives were employed and 111 intersected targets (Fig. 2) were achieved from Venny 2.1.0. The 111 common targets (Supplementary Table S11) were introduce in STRING database ver. 12.0 to build protein protein interaction (Fig. 3, Supplementary Table S12,), which revealed 382 edges. A high confidence (0.70) and an enrichment p-value of < 1.0e-16 were defined. Then top 20 hub genes were identified using cytohubba plugin in cytoscape 3.10.2 (Fig. 4). A network was constructed between phytoconstituents and common targets by using cytoscape 3.10.2 (Fig. 5).

### GO and KEGG analysis

**Fig. 6:**
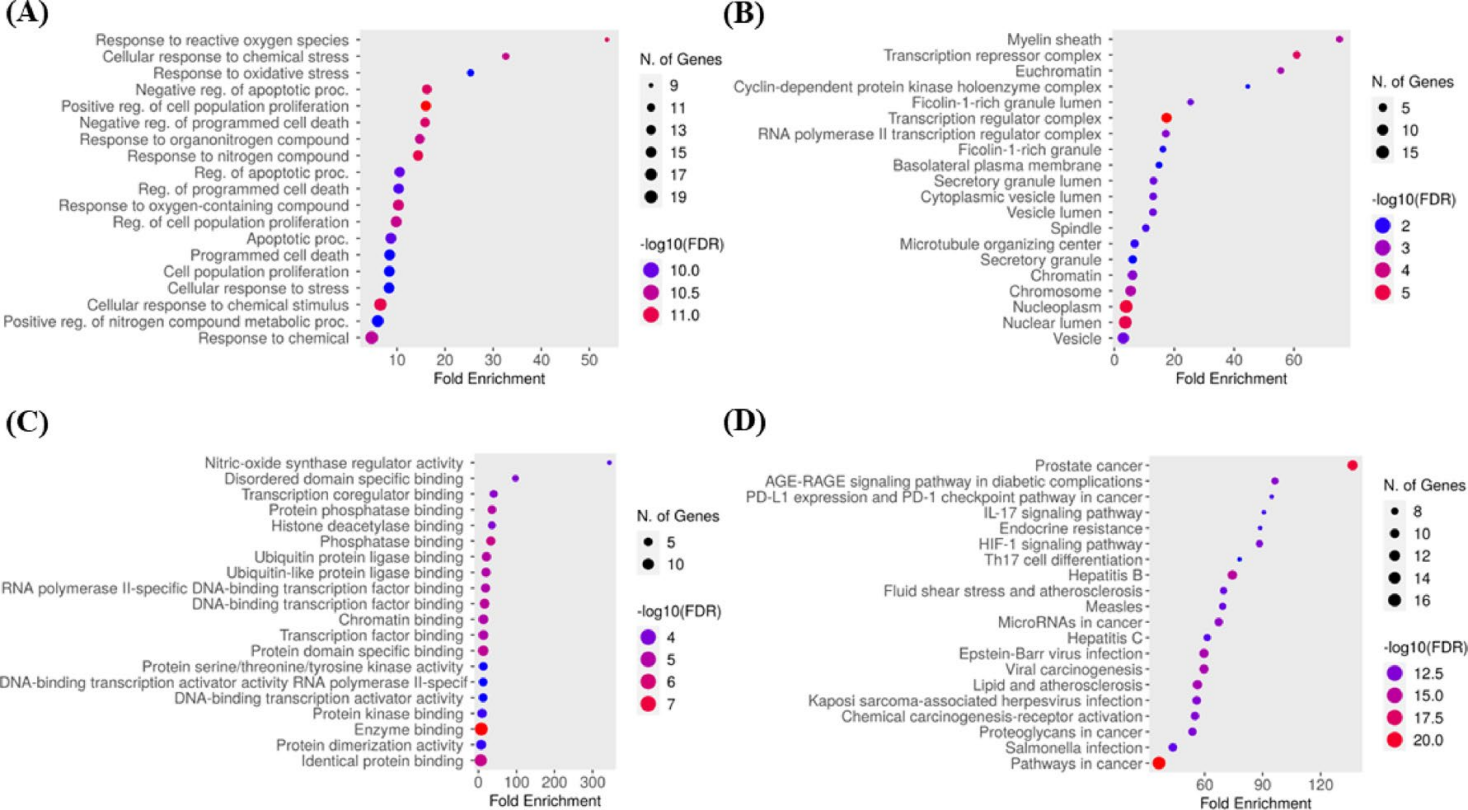
Results of GO and KEGG enrichment analysis. Dot plots of (**A**) biological process (**B**) cellular component (**C**) molecular function category terms from GO enrichment analysis and (**D**) the pathways highly relevant to the treatment effects of BME (the X-axis and Y-axis show the fold enrichment and full names of the processes, respectively, and the colour and size of each bubble represent the gene count and –log10FDR, respectively; the subsequent bubble charts are presented similarly). Pathway diagram adapted from KEGG^36–38^.

The GO enrichment analysis of common BME*-* UC targets identified certain GO terms which are associated with the therapeutic effects of BME on UC. The top 20 terms of the 3 categories i.e. biological process, cellular component, and molecular function were shown as bubble charts in Fig. 6A–C. These include oxidative stress, cell apoptosis and cell proliferation-related terms. The KEGG was employed to explore the signaling pathways, which clarified the mechanisms by which BME helped alleviating UC. A total of 197 signaling pathways were found out of which the top 20 pathways were depicted in Fig. 6D, which are arranged according to the number of genes. The findings indicated that the primary pathways were the pathway in cancer, prostate cancer, IL-17 signaling pathway and Th17 cell differentiation. In this study, the significant key targets of BME bio-actives which are responsible for the pathogenesis of UC were obtained by choosing the top 10 signaling pathways based on the number of genes. Further, the common target genes which were involved in ameliorating UC (Table 1).

**Table 1:** Top 10 pathways on the basis on number of genes.

Pathways	Targets
Pathways in cancer	CDK2, CTNNB1, ERBB2, AKT1, HDAC1, HIF1A, HSP90AA1, JUN, MDM2, MMP9, NFKB1, MAPK1, BCL2, RELA, STAT3, CASP3
Prostate cancer	CDK2, CTNNB1, ERBB2, AKT1, HSP90AA1, MDM2, MMP9, NFKB1, MAPK1, BCL2, RELA
Epstein-Barr virus infection	CDK2, AKT1, HDAC1, JUN, MDM2, NFKB1, BCL2, RELA, STAT3, CASP3
Hepatitis B	CDK2, AKT1, JUN, MMP9, NFKB1, MAPK1, BCL2, RELA, STAT3, CASP3
Lipid and atherosclerosis	AKT1, HSP90AA1, JUN, MMP9, NFKB1, MAPK1, BCL2, RELA, STAT3, CASP3
Viral carcinogenesis	CDK2, HDAC1, JUN, MDM2, NFKB1, MAPK1, RELA, STAT3, CASP3, CDK1
Chemical carcinogenesis-receptor activation	CYP3A4, AKT1, HSP90AA1, JUN, NFKB1, MAPK1, BCL2, RELA, STAT3
Kaposi sarcoma-associated herpesvirus infection	CTNNB1, AKT1, HIF1A, JUN, NFKB1, MAPK1, RELA, STAT3, CASP3
Proteoglycans in cancer	CTNNB1, ERBB2, AKT1, HIF1A, MDM2, MMP9, MAPK1, STAT3, CASP3
Salmonella infection	CTNNB1, AKT1, HSP90AA1, JUN, NFKB1, MAPK1, BCL2, RELA, CASP3

### Molecular docking validation

**Fig. 7:**
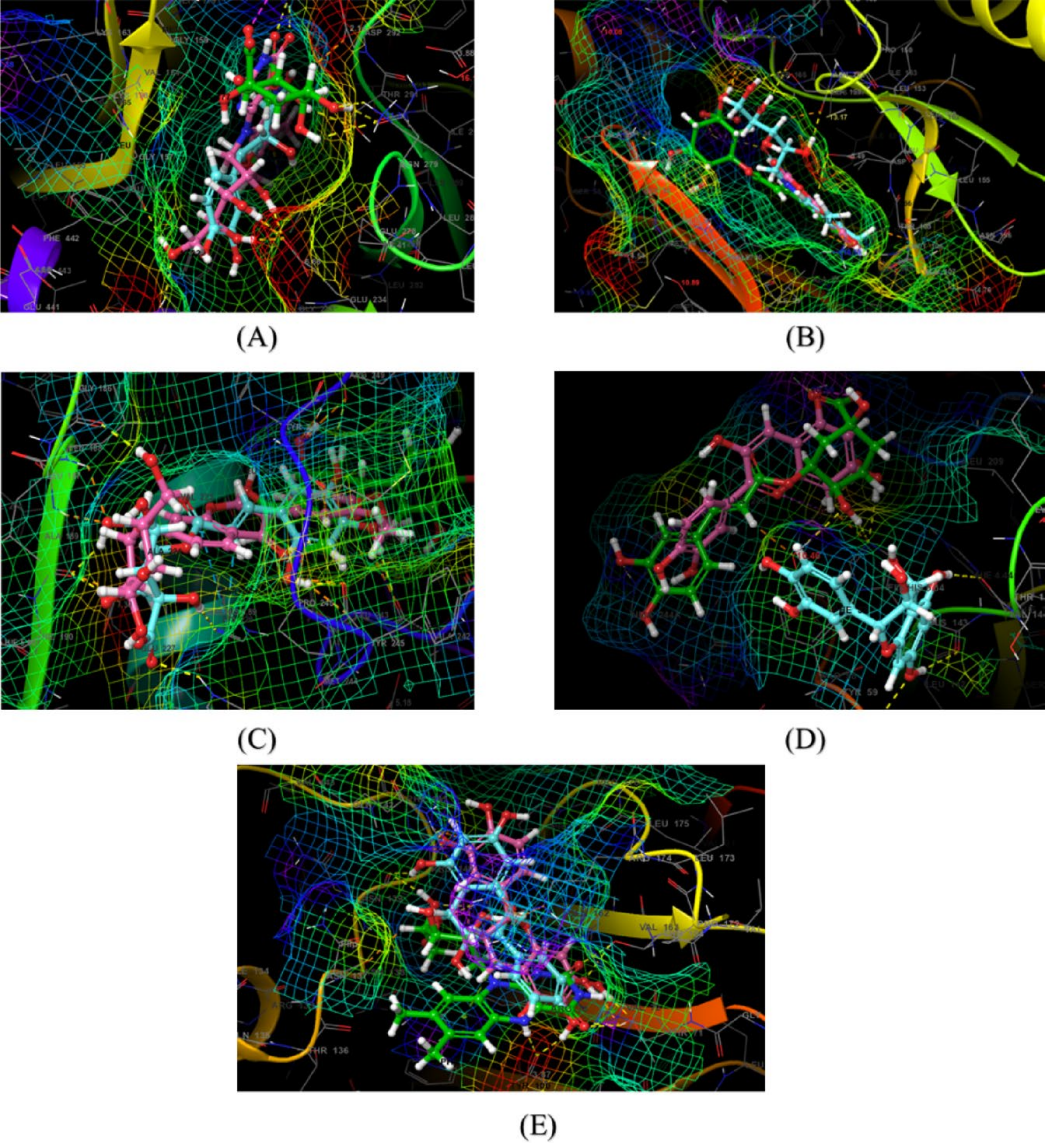
The results of Molecular docking (superimposition of the best active ingredients) of key targets i.e. AKT1 (**A**), MAPK1 (**B**), MMP9 (**C**), NF-κB (**D**) and RELA (**E**) and specific bioactives of BME.

**Fig. 8:**
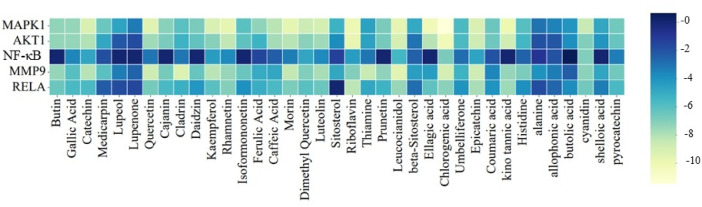
The docking scores of bioactive ingredients and key target proteins. Heatmap showing the binding affinities (docking scores) of bioactive compounds with MAPK1, AKT1, NF-κB, MMP9, and RELA.

On the basis of KEGG enrichment analysis, we took into account the participation of many signaling pathways that play a role in the pathogenesis of UC. This included cancer signaling, prostate cancer, IL-17 signaling, and Th17 cell differentiation, among others. We also identified numerous genes which are most likely to be playing a role in UC pathogenesis, i.e. MAPK1, NFKB1, MMP-9, AKT1, and RELA, that were enriched among the top ten pathways of KEGG. PPI analysis also emphasized these pathways as key targets in UC. For the confirmation of network pharmacology analysis results, molecular docking was performed among the active compounds and the top five most important protein targets. Molecular docking is a widely accepted technique in molecular interaction research that detects active binding sites along with key interacting residues among protein targets and small molecules. The interacting amino acid residues and the Glide XP docking scores are listed in (Fig. 8, Supplementary table S13 and Supplementary table S14, respectively). On the basis of the docking scores, we have analysed the 3D as well as the 2D interaction diagrams of the top three compounds to realize their biological activity (Fig. 7; Supplementary Figures S4-S7). Simulations of docking were carried out twice to establish data reliability. Negative binding energy is reflective of good interaction with the target. Binding energies of less than − 5 kcal/mol are regarded as being characteristic of good binding affinity, and less than − 7 kcal/mol indicative of strong potential for binding, which mirrors a stable ligand–receptor complex.

The top-scoring three phytoconstituents on the basis of GlideScores against MAPK1 (PDB code: 4FV1) were found to be chlorogenic acid (-11.491 Kcal/mol), elagic acid (-10.226 Kcal/mol), and riboflavin (-10.019 Kcal/mol). These three phytocompounds had the optimum interactions with the binding site^39^. The result indicated while both chlorogenic acid and elagic acid were shown to form a common hydrogen bond interaction with Met106 residue, on the other hand, both riboflavin and elagic acid formed a mutual H-bond with Gln103 residue. All three formed a mutual polar contact with Gln103, while elagic acid and riboflavin formed another mutual polar contact with Thr108 residue. Chlorogenic acid also formed a salt bridge interaction with Lys52 residue. Ile29, Val37, Ala50, Ile82, Met106, and Leu154 were the binding residues responsible for hydrophobic interactions in the binding pocket (Supplementary figure S4A and S7A). All three compounds superimposed well inside the binding pocket.

For the binding site^40^ of AKT1 (Gene: AKT1 (AlphaFold)), the best-scoring three phytocompounds on the basis of GlideScores were found to be chlorogenic acid (-10.165 Kcal/mol), riboflavin (-9.784 Kcal/mol), and leucocianidol (-9.010 Kcal/mol). From the ligand-interaction diagrams of these protein-ligand complexes, it was found that both chlorogenic acid and leucocianidol formed mutual hydrogen bonds with Ala230 and Glu291 residues, while riboflavin formed H-bonds with Leu156, Lys179, Glu234, and Asp439 residues. Chlorogenic acid also formed a salt bridge interaction with Lys179 residue. All three phytocompounds were shown to form mutual polar interactions with Thr211 and Thr291 residues. Hydrophobic contacts were largely facilitated by amino acid side chains Leu156, Val164, Ala177, Met227, Tyr229, Ala230, Met281, and Phe438, which shared participation (Supplementary figure S4B and S7B) inside the binding pocket (SiteMap-predicted, Schrödinger Inc.). All three compounds superimposed very well inside the binding pocket.

However, in NF-κB (PDB ID: 8TQD), chlorogenic acid (-6.842 Kcal/mol), cyanidin (-6.949 Kcal/mol), and leucocianidol (-5.509 Kcal/mol) were found to be top-scoring phytocompounds. It can be found that His143 was the mutual amino acid residue for the formation of hydrogen bonds among these three phytoconstituents. The common same residue was responsible for forming polar interaction, too. Tyr59 was found to be the mutual amino acid contributing to hydrophobic contacts. Chlorogenic acid and cyanidin were found to form one salt bridge interaction with Lys243 and Asp241 residues, respectively (Supplementary figure S5A and S7C). These phytoconstituents were not found to superimpose inside the binding pocket.

In the active site^41^ of MMP9 (PDB code: 5CUH), leucocianidol (-9.501 Kcal/mol), cladrin (-9,329 Kcal/mol), and chlorogenic acid (-8.932 Kcal/mol) were found to be the top 3 phytoconstituents. Both chlorogenic acid and leucocianidol were found to have the mutual hydrogen binding interactions with Arg249. Cladrin was also found to form pi-pi stacking interactions with His226. All three phytocompounds formed polar contacts with common residue His226. Amino acids Leu222, Val223, Ala242, Leu243, Tyr245, Pro246, Met247, and Tyr248 formed hydrophobic contacts within the binding pocket (Supplementary figure S5B and S7D). All three compounds superimposed very well inside the binding pocket.

In the binding site^42^ of RELA (Gene: RELA), riboflavin (-8.240 Kcal/mol), epicatetchin (-7.805 Kcal/mol), and leucocianidol (-7.420 Kcal/mol) were found to be the top 3 phytocompounds. From the docked complexes, it was found that Thr71 was the common H-bond forming residue. Thr71, Asn139, Gln162, and Thr164 were found to be the most common polar residues. Riboflavin was found to form a pi-cation interaction with Arg174 residue (Fig. 8).

Pro140, Val163, and Val172 were the mutual amino acids that are responsible for the hydrophobic interactions. On the other hand, Asn 370 was the common amino acids for polar interactions (Supplementary figure S6 and S7E). All three compounds superimposed well inside the binding pocket.

### Induced fit docking

IFD is an in-silico method employed to investigate the structural and conformational variations taking place during ligand–receptor binding. The receptor is considered flexible around the binding site, whereas the ligand is kept rigid.

For the IFD of MAPK1 with chlorogenic acid, the Glide score reduced from − 11.491 kcal/mol (calculated using default XP-mode docking) to − 9.147 kcal/mol. The IFD Score was − 763.93. The count of hydrogen bonds was equal in both IFD and XP-mode poses. Asp104 that was seen in the XP-mode pose was not seen in the pose that had been generated using IFD, which, instead, featured extra interactions with Lys52 and Arg65. The number of polar interacting residues was increased to two from one, including both Gln103 being shared by both poses and Asn152 being newly engaged in the IFD pose. Both docking poses were also well accommodated in the hydrophobic binding pocket facilitated by conserved residues such as Ile29, Ala33, Tyr34, Met36, Val37, Ala50, Ile82, Leu105, Met106, and Leu154. Overall, the ligand exhibited a similar binding orientation in both docking methods (Supplementary Figure S9A).

The AKT1 IFD with chlorogenic acid reduced the Glide score from − 10.165 kcal/mol (by general XP-mode docking) to -8.665 kcal/mol. The respective IFDScore was − 1041.67. The count of the residues involved in hydrogen bond formation decreased from six in XP-mode pose to five in the pose generated by IFD, since Lys276 no longer formed a hydrogen bond. One new residue forming the hydrogen bond was identified as Asp292 instead. The two docking conformations also had the same polar interacting residues, as mentioned earlier in Sect. 3.1. Lys276 was particularly notable in forming a new salt bridge when in the IFD conformation. The two conformations also nicely fit into the hydrophobic binding pocket of conserved amino acids such as Leu156, Val164, Ala177, Met227, Tyr229, Ala230, Met281, and Phe438. In general, the ligand maintained a stable binding conformation with each docking algorithm, well in line with the sitemap-predicted binding site of AKT1 (Supplementary Figure S9B).

IFD of NF-κB with chlorogenic acid resulted in a slight decrease in the Glide score, from − 6.842 kcal/mol (standard XP-mode docking) to − 6.792 kcal/mol. The IFDScore was calculated as − 432.72. Interestingly, the number of hydrogen bonds increased from three in the XP-mode poses to four in the IFD-generated pose, with Ser210 and Asp241 identified as the additional residues contributing to these interactions. The quantity of polar residues was hiked from two to four, and these newly formed residues were Hie111 and Thr145. The salt-bridge forming residue was also changed from Lys243 to Lys146. Moreover, the no. of hydrophobic pocket forming residues had been dramatically increased from two to five, and the newer hydrophobic residues were found to be Val60, Cys61, Val144, and Leu209. Both docked structures were not overlapped at all into the binding site of NF-κB (Supplementary Figure S9C).

IFD of MMP9 with leucocianidol reduced the Glide score, from − 9.501 kcal/mol (regular XP-mode docking) to -9.232 kcal/mol, and its IFDScore was − 347.29. The interesting aspect was that the number of hydrogen bonds was higher in the IFD pose than in the XP-mode pose, from three in XP-mode pose to six in the IFD pose, with Leu222, Glu227, and Ala242 as new hydrogen bond-forming residues. But the number of polar interactions fell from three to two since His257 was not present in the IFD pose. Another new π–π stacking interaction was revealed with His226. Both the poses were able to comfortably fit into the hydrophobic binding pocket, lined with conserved residues like Leu222, Val223, Ala242, Leu243, Tyr245, Pro246, Met247, Tyr248, Phe250, and Pro255. The IFD pose also made three more hydrophobic contacts with Leu188, Ala189, and Ala225. By and large, the two docking structures had minimal overlap within the MMP9 ligand-binding site (Supplementary Figure S9D).

Following IFD of RELA and riboflavin, the Glide score came down from − 8.240 kcal/mol (obtained by routine XP-mode docking) to − 7.575 kcal/mol. The IFDScore was − 927.25. Hydrogen bond interactions were enhanced from five for XP-mode pose to nine for IFD-generated pose, and Arg73, Glu101, Asn137, Asn138, and Gln142 were found as new hydrogen bond-forming residues. However, the number of polar interactions decreased from eight to seven upon addition of the new polar residue Gln142. The two docking poses were nicely fitted into the hydrophobic binding pocket formed by conserved residues Pro140 and Val163. Furthermore, the IFD pose had three extra hydrophobic contacts with Leu175, Pro176, and Pro177 that were not observed in the XP-mode pose. Collectively, the two docked complexes exhibited modest overlap in the sitemap-predicted RELA ligand-binding site (Supplementary Figure S9E).

A bar plot was created in an attempt to graph the IFD scores for the ligand–receptor complexes above. (Supplementary Figure S9F).

### MM-GBSA calculation

To calculate the free binding energies and analyze biophysical origins of molecular recognition in the top three protein–ligand complexes for each target, the MM-GBSA method was utilized. The method yielded comprehensive information regarding various energy aspects, i.e., ΔG Bind, ΔG Coulomb, ΔG Covalent, ΔG H-bond, ΔG Lipo, ΔG Packing, ΔG Solv, and ΔG van der Waals (Supplementary Table S15). MM-GBSA calculations were carried out in duplicate for the verification of results.

### MD simulations

**Fig. 9:**
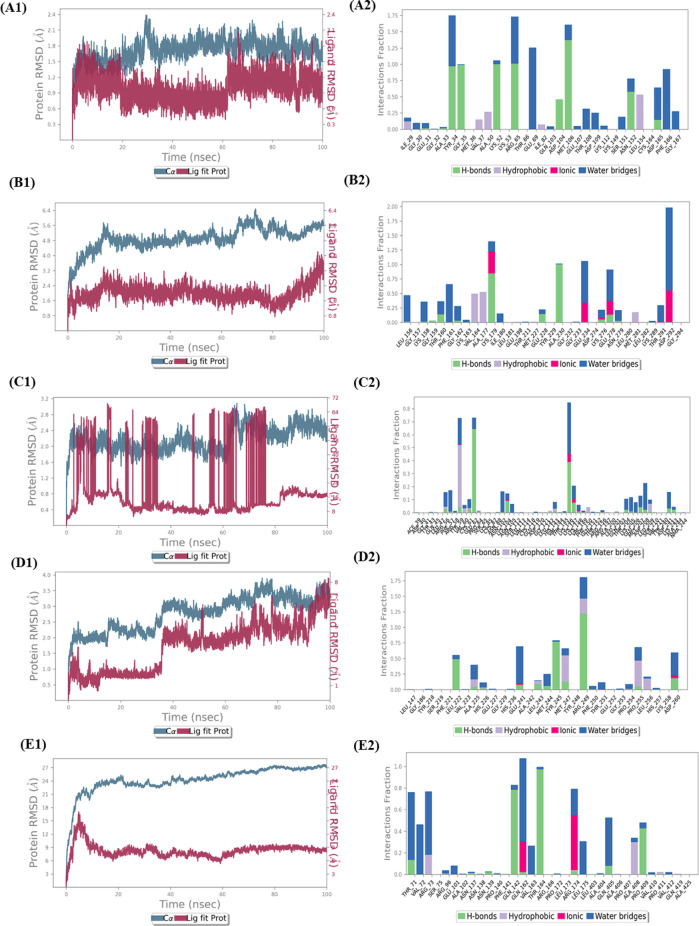
Key target-ligands’ MD simulation results. [Representation in 2D and Protein-Ligands contact analyses of MAPK1-chlorogenic acid (**A1**,**A2**), AKT1- chlorogenic acid (**B1**,**B2**), NF-κB-chlorogenic acid (**C1**,**C2**), MMP9-leucocianidol (**D1**,**D2**), RELA-riboflavin (**E1**,**E2**) complexes].

Although the results of molecular docking could provide an insight about the amino acids interacting with the ligands, but MD simulations could provide the detailed information about the protein-ligand complexes (Fig. 9). In this aspect, from MAPK1-chlorogenic acid complex, for the first 20 ns, the protein-ligand complex was stabilized, then, till 60 ns the complex was fluctuated, and finally, from 60 to 100 ns, the complex was stabilized. Tyr35, Gly35, Lys52, Arg65, Asp104, Asp104, Met106, Asn152, and Asn165 were H-bond forming residues. On the other hand, Tyr34, Arg65, Glu69, Asp165, Phe166, and Gly167 were found to be the most significant water bridge forming residues. Whereas, Ile29, Val37, Als50, and Leu154 were among the contributing hydrophobic residues (Fig. 9).

From AKT1-chlorogenic acid complex, the protein RMSD was not found to be stabilized with that of the ligand throughout 100 nd of dynamics. Out of the responsible amino acid residues, Lys179 and Ala 230 was mostly contributed to H-bond formation, whereas, Val164, Ala177, and Met281 were hydrophobic interaction forming residues. Lys179, Glu234, Glu278, and Asp292 were the most significant ionic bond forming residues. Leu156, Lys158, Thr160, Phe161, Gly162, Glu234, Glu278, and Asp292 were among the most significant water bridge-forming residues (Fig. 9).

From NF-κB-chlorogenic acid complex, the protein RMSD was not found to be stabilized with that of the ligand throughout 100 nd of dynamics. Out of the responsible amino acids, Glu62 and Lys146 were found to be the significant H-bond-forming residues. On the other hand, Tyr59 was an important hydrophobic residue. Most of all the residues were found to form water bridges, in which, Phe57, Lys146, and Asp208 were found to be the most significant ones (Fig. 9).

From MMP9-leucocianidol complex, it was found that uptil around 40 ns, the protein RMSD went till around 3 Å, and then for the next 50 ns, the protein RMSD went till around 3.5 Å. Finally, for the last 10 ns, the protein-ligand RMSD was stabilized at above 3.5 Å. Out of the responsible amino acid residues, Leu222, Tyr245, and Arg249 were found to be most contributing H-bond forming residues. Met247 and Pro255 were found to be the most responsible hydrophobic residues. In this case too, most of the residues were found to form water bridges, in which, Met247 and Pro255 were the most important ones (Fig. 9).

From RELA-riboflavin complex, it was found that the protein RMSD was not found to be stabilized with that of the ligand throughout 100 nd of dynamics. Among the responsible amino acid residues, Gln142, Thr164, and Pro409 were the most significant H-bond forming residues. Arg73 and Ala408 were the solely responsible hydrophobic residues. On the other hand, Gln162 and Arg174 were found to be ionic residues. Lastly, among the water bridging residues, Thr71, Val72, Arg73, Gln162, Val163, Arg174, Leu175, and Gln405 were found to be the most important ones (Fig. 9).

### In silico physicochemical and ADME/T studies

The three lowest binding energies-containing phytoconstituents with the lowest binding energies among the 5 target proteins were chlorogenic acid, leucocianidol, and riboflavin. In silico ADME/T profiles of these were simulated using Schrödinger’s QikProp version 2017-2 and the SwissADME web server, and were listed in Supplementary Table S16. All parameters predicted by QikProp were within acceptable ranges. Molecular volume, surface area, percent human oral absorption, and molecular weight of all the compounds were in the recommended range. Also, their surface area, Log P, and polarizability were within acceptable ranges.

Based on the SwissADME predictions and BOILED-Egg diagram results, none of the three bioactive compounds appeared to be CNS-active (Supplementary Figure S11). Additionally, they were not identified as substrates of P-glycoprotein (PGP). Additionally, they were also not found to be substrates of P-glycoprotein (PGP). The three substances were also found to be non-inhibitors of the Cytochrome P450 enzymes CYP1A2, CYP2C19, CYP2C9, CYP2D6, and CYP3A4. Out of these three compounds, only leucocianidol was found to possess high GI absorption, while, only riboflavin showed no violations of Lipinski’s rule. In general, such phytoconstituents can be further processed for future research in view of their significant pharmacokinetic property and drug-likeness.

## Discussion

UC is a chronic destructive inflammatory disease of the gastrointestinal tract which mainly effects the colon to rectum region. UC is characterized by rectal bleeding, diarrhoea, abdominal pain, body weight loss which are caused by inflammation, ulceration and malabsorption in mucosal layer. Conventional treatment of UC is associated with certain adverse effects; therefore, there is a need to explore alternative traditional medicine approaches. *B. monosperma* is an important medicinal plant in the Ayurvedic system of medicine. Traditionally, its stem bark has been used for various gastrointestinal disorders, including *grahani roga*. The stem bark also exhibits different pharmacological properties, including hepatoprotective, anti-inflammatory, antioxidant and wound-healing activities. So, the aim of our study to explore the therapeutic potential of *B. monosperma* in alleviating UC through in silico approaches.

The results of the network pharmacology analysis indicated that multiple bioactive compounds in BME could target several proteins involved in key biological processes and signaling pathways associated with UC Thus, this study provides a scientific basis for the potential use of *B. monosperma* stem bark in the management of UC.

The result of GO enrichment analysis identifies the different biological processes associated with *B. monosperma* in UC such as cell proliferation, oxidative stress, and epithelial cell apoptosis-related terms. Additionally, KEGG enrichment analysis predicted that major pathways involved in the therapeutic effects of BME on UC include the pathway in cancer, prostate cancer, IL-17 signaling pathway and Th17 cell differentiation etc. These pathways primarily contribute to the production of pro-inflammatory cytokines and colon mucosal damage, which are hallmark features of UC.

The results of the PPI network and GO analysis showed that the BME-UC common targets were mainly the key proteins involving the pathways that are I-kappaB kinase/ NF-κB signaling (RELA TNF HDAC1 CTNNB1 AKT1), cytokines (TNF), cell migration (HDAC1 AKT1 TNF HSP90AA1 HIF1A MMP9 CTNNB1 STAT3 BCL2 JUN MAPK1 CDK1), Oxidative stress (HIF1A CTNNB1 TNF AKT1) epithelial cell apoptosis (CASP3 BCL2 TNF). In UC, antigens are recognized by dendritic cells and macrophages, which then trigger the production of pro-inflammatory cytokines such as TNF-α, IL-12, IL-23, IL-6, and IL-1β^1^. These pro-inflammatory cytokines cause the activation and attraction of the immune cells like neutrophils and T cells which in turn cause colon tissue damage and inflammation by facilitating the apoptotic process^43,44^. In the current study, the result showed that phytoconstituents of BME target both the cytokine TNF and and NF-κB pathway, which is responsible for the production of the pro-inflammatory cytokines. Previous studies suggests that phytoconstituents of BME inhibit the production of pro-inflammatory cytokines by blocking the activation of NF-κB pathway, which supports the current findings^45,46^. Immune cell migration in UC disrupts the intestinal barrier, leading to chronic inflammation. Neutrophils, macrophages, and T cells infiltrate the mucosa, releasing cytokines like TNF, ROS, and Matrix Metalloproteinase 9 (MMP9), exacerbating tissue damage and sustaining disease progression^47^ MMP9 plays a pivotal role in degrading collagen and extracellular matrix (ECM) components, thereby facilitating immune cell migration into inflamed tissue^48^. Its expression is induced by transcription factors like Hypoxia-Inducible Factor 1 Alpha (HIF1A)^49^ and Signal Transducer and Activator of Transcription 3 (STAT3)^50^, which further amplifies tissue destruction and leukocyte infiltration. Additionally, HDAC1 is essential for CD4^+^ T cells to cross endothelial barriers and to enter tissues such as the gut lamina propria and epithelium^51^. BCL2 regulates the survival and longevity of mature dendritic cells^52^. Furthermore, Mitogen-activated protein kinase (MAPK) regulates cell migration by JUN and microtubule associated proteins^53^. Epithelial cell apoptosis plays a significant role in the pathogenesis of UC by disrupting intestinal mucosal integrity and barrier function, which leads to increased permeability and inflammation^54^. Elevated levels of tumor necrosis factor (TNF) in UC can lead to increased activation of the extrinsic apoptotic pathway, resulting in Caspase-3 (CASP3) activation and subsequent epithelial cell apoptosis^55^. Reduced expression of B-cell lymphoma 2 (BCL-2) can lead to epithelial cell apoptosis. BCL-2 is an anti-apoptotic protein that plays a crucial role in cell survival by inhibiting the mitochondrial (intrinsic) apoptotic pathway^56^. Previous studies revealed that phytochemicals such as kaempferol, beta-sitosterol, caffeic acid, ferulic acid, quercetin^57^, chlorogenic acid^58^, luteolin^59^ and gallic acid^46^ treat UC by modulating the apoptosis of intestinal epithelial cells (IECs). In this study, the phytoconstituents of BME also interacts with these targets which supports the previous findings and highlights the anti-apoptotic role of *B. monosperma* in amelioration of UC.

Oxidative stress plays a critical role in the pathogenesis of UC by inducing cellular damage and inflammation in the colon^60^. AKT1 is a major protein of PI3K/AKT pathway which induce the production of pro-inflammatory cytokines^61^ such as TNF correlate with increased neutrophil accumulation and activity, which directly contributes to oxidative stress and subsequent epithelial injury in UC^62^. HIF1A overexpression has been shown to activate pathways that induce oxidative stress^63^. Β-catenin (CTNNB1) can enhance ROS production, which contributes to cellular damage and apoptosis^64^. The present study revealed that the phytoconstituents have regulated AKT1, TNF, HIF1A and CTNNB1 which are involved in inducing oxidative stress. Previous experimental studies states that stem bark of *B. monosperma* exhibit anti-oxidative activity which supports our findings^65^.

Based on recent findings in network pharmacology, five key target proteins AKT1, MAPK1, MMP9, NF-κB, and RELA were identified as being closely associated with the progression of UC. These targets were subsequently subjected to molecular docking studies with the bioactive compounds of BME to evaluate their potential interactions. Negative binding energy indicates the effective binding to the target. Lower binding energy suggested stable ligand-receptor complex better explained as <-5 kcal/mol called as good binding activity and <-7 kcal/mol called as strong binding activity. Except for the NF-κB all the Glide Scores of the top three phytoconstituents against the other four proteins were in the range of -11.491 to -7.420 kcal/mol. The top-scoring phytocompounds for the aforementioned proteins demonstrated a strong binding interaction with their corresponding targets. It involves unforeseeable bias from the materiality, which could expedite to anonymous fallacy in in-vitro or in-vivo studies. However, this molecular docking outcomes throwback plausible treatment mechanisms and it may direct the animal affirmation experiment.

In summary, this study demonstrates the therapeutic potential of *B. monosperma* stem bark in UC through integrative in silico approaches involving network pharmacology, molecular docking, and MD simulations. Nonetheless, as the results are derived solely from computational predictions, critical factors such as pharmacokinetics, bioavailability, and phytochemical interactions remain to be experimentally validated. Future in vitro and in vivo investigations, complemented by omics-based approaches, are essential to validate these findings. Collectively, this study identifies *B. monosperma* as a promising source of lead compounds for UC, bridging its traditional use with modern pharmacological evidence.

## Conclusion

Results revealed that *B. monosperma* stem bark targets key proteins and pathways implicated in UC pathogenesis, including inflammation, oxidative stress, and cell migration. Specifically, compounds from *B. monosperma* stem bark showed strong predicted interactions with targets like MAPK1, AKT1, and NF-κB, suggesting a potential molecular basis for its therapeutic effects. These in silico findings warrant further experimental validation to confirm the efficacy of the stem bark of *B. monosperma* as a novel treatment strategy for UC.

## Supplementary Information

Below is the link to the electronic supplementary material.

Supplementary Material 1

## Data Availability

All relevant data can be found in the main manuscript or supplementary materials.

## Abbreviations

AKT1: AKT serine/threonine kinase 1
BME: *Butea monosperma* extract
GO: Gene ontology
IFD: Induce fit docking
IL: Interleukin
KEGG: Kyoto Encyclopedia of Genes and Genomes
LC: Liquid chromatography
MAPK1: Mitogen-activated protein kinase 1
MMP9: Matrix metallopeptidase-9
NF-κB: Nuclear factor kappa B
MS: Mass spectroscopy
PPI: Protein protein interaction
RELA: v-rel avian reticuloendotheliosis viral oncogene homolog A
Th: T helper
UC: Ulcerative colitis

## References

[1] Ordás, I., Eckmann, L., Talamini, M., Baumgart, D. C. & Sandborn, W. J. Ulcerative colitis. *Lancet* **380**, 1606–1619 (2012). 22914296 10.1016/S0140-6736(12)60150-0

[2] Weisman, M. H. et al. Inflammatory bowel disease prevalence: surveillance data from the U.S. National Health and Nutrition Examination Survey. *Prev. Med. Rep.* **33**, 102173 (2023). 37223580 10.1016/j.pmedr.2023.102173 PMC10201824

[3] Gunisetty, S. et al. The epidemiology and prevalence of ulcerative colitis in the South of India. *Open. J. Immunol.* **2**, 144–148. 10.4236/oji.2012.24018 (2012).

[4] Kucharzik, T., Koletzko, S. & Kannengießer, K. & Dignaß, A. Ulcerative colitis—diagnostic and therapeutic algorithms. *Dtsch Arztebl Int* **117**, (2020). 10.3238/arztebl.2020.0564 PMC8171548 33148393

[5] Sharma, N. & Garg, V. Antidiabetic and antioxidant potential of ethanolic extract of *Butea monosperma* leaves in alloxan-induced diabetic mice. *Indian J. Biochem. Biophys.* **46**, 99–105 (2009). 19374261

[6] Sutariya, B. & Saraf, M. A comprehensive review on pharmacological profile of *Butea monosperma* (Lam.) Taub. *J. Appl. Pharm. Sci.* **5**, 159–166 (2015).

[7] Government of India, Ministry of Health and Family Welfare, Department of AYUSH. *The Ayurvedic Pharmacopoeia of India* Part I, Vol. II (The Controller of Publications).

[8] Yadahalli, S. L., KH, M., B, M. & Desai, A. S. Management of *Grahani roga* with special reference to ulcerative colitis: a case report. *Int. J. Res. Ayurveda Pharm.* **15**, 22–26 (2024).

[9] Sharma, D. et al. Antibacterial and antidiarrheal activity of *Butea monosperma* bark extract against waterborne *Enterobacter cloacae* in rodents: in-vitro, ex-vivo and in-vivo evidences. *J. Ethnopharmacol.* **241**, 112014 (2019). 31181315 10.1016/j.jep.2019.112014

[10] Kaur, V. et al. Hepatoprotective activity of *Butea monosperma* bark against thioacetamide-induced liver injury in rats. *Biomed. Pharmacother*. **89**, 332–341 (2017). 28237915 10.1016/j.biopha.2017.01.165

[11] Khandelwal, K. *R.Practical Pharmacognosy* 9th edn, 149–153 (Nirali Prakashan, 2002).

[12] Kokate, C. K., Purohit, A. P. & Gokhale, S. B. *Pharmacognosy* 21st edn, 377–378 (Nirali Prakashan, 2002).

[13] Singh, P. et al. Pharmacognostical study and establishment of quality parameters of aerial parts of *Costus speciosus*–a well-known tropical folklore medicine. *Asian Pac. J. Trop. Biomed*. **4**(6), 486 –491. 10.12980/APJTB.4.2014C1103 (2014) 10.12980/APJTB.4.2014C1103 PMC3994359 25182951

[14] Jain, N. K. et al. Integrating network pharmacology with molecular docking to rationalize the ethnomedicinal use of *Alchornea laxiflora* (Benth.) Pax & K. Hoffm. for efficient treatment of depression. *Expt. Pharmacol. Drug Discov.* **15**, (2024). 10.3389/fphar.2024.1290398 PMC10949534 38505421

[15] Yang, Y. et al. Efficient exploration of chemical space with docking and deep learning. *J. Chem. Theory Comput.* **17**, 7106–7119 (2021). 34592101 10.1021/acs.jctc.1c00810

[16] Schrödinger *Release 2017-2: Glide* (Schrödinger, LLC, 2017).

[17] Das, A., Nandi, A., Kumari, V. & &Alvala, M. FBDD & de Novo drug design. In *Applied Computer-Aided Drug Design: Models and Methods* 159–201 (Bentham Science Publishers, 2023).

[18] Varadi, M. et al. AlphaFold protein structure database: massively expanding the structural coverage of protein-sequence space with high-accuracy models. *Nucleic Acids Res.* **50**, D439–D444. 10.1093/nar/gkab1061 (2022). 34791371 10.1093/nar/gkab1061 PMC8728224

[19] Schrödinger *Release 2017-2: LigPrep* (Schrödinger, LLC, 2017).

[20] Madan, A. et al. SAR-based review on diverse heterocyclic compounds with various potential molecular targets in the fight against COVID-19: a medicinal chemist perspective. *Curr. Top. Med. Chem.* **23**, 1319–1339 (2023). 36703601 10.2174/1568026623666230126104156

[21] Schrödinger Release 2017-2. *Protein Preparation Workflow; Epik; Impact; Prime* (Schrödinger, LLC, 2017).

[22] Nandi, A., Chattaraj, B., Das, A., Prasad, R. & Dey, Y. N. Inhibiting brushite crystal growth: molecular docking exploration of *Enhydra fluctuans* phytoconstituents and their interaction with human serum albumin. *J. Biomol. Struct. Dyn.* 1–10 10.1080/07391102.2024.2442761 (2024). 10.1080/07391102.2024.2442761 39703176

[23] Allegra, M. et al. Evaluation of the IKKβ binding of indicaxanthin by induced-fit docking, binding pose metadynamics, and molecular dynamics. *Front. Pharmacol.* **12**, 701568 (2021). 34566634 10.3389/fphar.2021.701568 PMC8461089

[24] Farid, R., Day, T., Friesner, R. A. & Pearlstein, R. A. New insights about HERG blockade obtained from protein modeling, potential energy mapping, and Docking studies. *Bioorg. Med. Chem.* **14**, 3160–3173 (2006). 16413785 10.1016/j.bmc.2005.12.032

[25] Schrödinger Release 2017-2. *Induced Fit Docking protocol; Glide; Prime* (Schrödinger, LLC, 2017).

[26] Harder, E. et al. OPLS3: a force field providing broad coverage of drug-like small molecules and proteins. *J. Chem. Theory Comput.* **12**, 281–296 (2016). 26584231 10.1021/acs.jctc.5b00864

[27] Nandi, A., Nigar, T., Das, A. & Dey, Y. N. Network pharmacology analysis of *Plumbago zeylanica* to identify the therapeutic targets and molecular mechanisms involved in ameliorating hemorrhoids. *J. Biomol. Struct. Dyn.* **43**, 161–175. 10.1080/07391102.2023.2280681 (2023). 37948311 10.1080/07391102.2023.2280681

[28] Nandi, A., Auti, P. S., Jagtap, U. A. & Paul, A. T. Investigating the role of indole and quinazolinone-based hybrid analogues with ketoamide fragment and alkyl extension for potential PL inhibition. *J. Mol. Struct.* **1301**, 137337. 10.1016/j.molstruc.2023.137337 (2024).

[29] Jacobson, M. et al. A hierarchical approach to all-atom protein loop prediction. *Proteins* **55**, 351–367 (2004). 15048827 10.1002/prot.10613

[30] Nandi, A., Mandal, N., Das, A. & Dey, Y. N. Approaches based on network pharmacology and molecular docking to predict the molecular mechanism of *Plumbago zeylanica*’s anti-inflammatory action. *J. Biol. Regul. Homeost.* **Agents38**, 2055–2067. 10.23812/j.biol.regul.homeost.agents.20243803.161 (2024).

[31] Bowers, K. J. et al. Molecular dynamics—scalable algorithms for molecular dynamics simulations on commodity clusters. In *Proc. 2006 ACM/IEEE Conf. Supercomputing (SC ’06)*, 84 10.1145/1188455.1188544 (2006).

[32] D. E. Shaw Research & Schrödinger LLC. Schrödinger Release 2017-2: Desmond Molecular Dynamics System and Maestro-Desmond Interoperability Tools. *New York, NY* https://www.deshawresearch.com/resources_desmond.html and https://www.schrodinger.com, (2017).

[33] The University of Queensland Research Computing Centre. Bunya supercomputer. *Brisbane, Queensland, Australia*10.48610/wf6c-qy55 (2024).

[34] Schrödinger, L. L. C. Schrödinger Release 2017-2: QikProp. *New York, NY* https://www.schrodinger.com (2017).

[35] Daina, A., Michielin, O. & Zoete, V. SwissADME: : a free web tool to evaluate pharmacokinetics, drug-likeness and medicinal chemistry friendliness of small molecules. *Sci. Rep.* **7**, 42717 (2017). 28256516 10.1038/srep42717 PMC5335600

[36] Kanehisa, M. et al. KEGG: biological systems database as a model of the real world. *Nucleic Acids Res.* **53**, D672–D677. 10.1093/nar/gkae909 (2025). 39417505 10.1093/nar/gkae909 PMC11701520

[37] Kanehisa, M. & Goto, S. K. E. G. G. Kyoto encyclopedia of genes and genomes. *Nucleic Acids Res.* **28**, 27–30 (2000). http://www.genome.ad.jp/kegg/10592173 10.1093/nar/28.1.27 PMC102409

[38] Kanehisa, M. Toward understanding the origin and evolution of cellular organisms. *Protein Sci.* **28**, 1947–1951. 10.1002/pro.3715 (2019). 31441146 10.1002/pro.3715 PMC6798127

[39] Methot, J. L. et al. Optimization of versatile oxindoles as selective PI3Kδ inhibitors. *ACS Med. Chem. Lett.* **11**, 2461–2469 (2020). 33335668 10.1021/acsmedchemlett.0c00441 PMC7734802

[40] Sogabe, S. et al. Structure-based approach for the discovery of pyrrolo[3,2-d]pyrimidine-based EGFR T790M/L858R mutant inhibitors. *ACS Med. Chem. Lett.* **4**, 201–205 (2013). 24900643 10.1021/ml300327z PMC4027575

[41] Camodeca, C. et al. Discovery of a new selective inhibitor of A disintegrin and metalloprotease 10 (ADAM-10) able to reduce the shedding of NKG2D ligands in Hodgkin’s lymphoma cell models. *Eur. J. Med. Chem.* **111**, 193–201 (2016). 26871660 10.1016/j.ejmech.2016.01.053

[42] Garcin, E. D. et al. Anchored plasticity opens doors for selective inhibitor design in nitric oxide synthase. *Nat. Chem. Biol.* **4**, 700–707 (2008). 18849972 10.1038/nchembio.115 PMC2868503

[43] Song, Y., Yuan, M., Xu, Y. & Xu, H. Tackling inflammatory bowel diseases: targeting proinflammatory cytokines and lymphocyte homing. *Pharmaceuticals* **15**, 1080 (2022). 36145301 10.3390/ph15091080 PMC9502105

[44] McDaniel, D. K., Eden, K., Ringel, V. M. & Allen, I. C. Emerging roles for noncanonical NF-κB signaling in the modulation of inflammatory bowel disease pathobiology. *Inflamm. Bowel Dis.* **22**, 2265–2279 (2016). 27508514 10.1097/MIB.0000000000000858 PMC4992436

[45] Rasheed, Z., Akhtar, N., Khan, A., Khan, K. A. & Haqqi, T. M. Butrin, isobutrin, and butein from medicinal plant *Butea monosperma* selectively inhibit nuclear factor-κB in activated human mast cells: suppression of tumor necrosis factor-α, interleukin (IL)-6, and IL-8. *J. Pharmacol. Exp. Ther.* **333**, 354–363 (2010). 20164300 10.1124/jpet.109.165209 PMC2872957

[46] Zhu, L., Gu, P. & Shen, H. Gallic acid improved inflammation via NF-κB pathway in TNBS-induced ulcerative colitis. *Int. Immunopharmacol.* **67**, 129–137 (2019). 30544066 10.1016/j.intimp.2018.11.049

[47] Kałużna, A., Olczyk, P. & Komosińska-Vassev, K. The role of innate and adaptive immune cells in the pathogenesis and development of the inflammatory response in ulcerative colitis. *J. Clin. Med.* **11**, 400 (2022). 35054093 10.3390/jcm11020400 PMC8780689

[48] He, L. et al. The Immunomodulatory role of matrix metalloproteinases in colitis-associated cancer. *Front. Immunol.* **13**, 1115922 (2023). 10.3389/fimmu.2022.1093990 PMC9910179 36776395

[49] Choi, J. Y., Jang, Y. S., Min, S. Y. & Song, J. Y. Overexpression of MMP-9 and HIF-1α in breast cancer cells under hypoxic conditions. *J. Breast Cancer*. **14**, 88–95 (2011). 21847402 10.4048/jbc.2011.14.2.88 PMC3148536

[50] Jia, Z. H. et al. Phosphorylation of STAT3 at Tyr705 regulates MMP-9 production in epithelial ovarian cancer. *PLoS One*. **12**, e0183622 (2017). 28859117 10.1371/journal.pone.0183622 PMC5578655

[51] Hamminger, P. et al. Histone deacetylase 1 controls CD4⁺ T cell trafficking in autoinflammatory diseases. *J. Autoimmun.* **119**, 102610 (2021). 33621930 10.1016/j.jaut.2021.102610

[52] Nopora, A. & Brocker, T. Bcl-2 controls dendritic cell longevity in vivo. *J. Immunol.* **169**, 3006–3014 (2002). 12218115 10.4049/jimmunol.169.6.3006

[53] Huang, C., Jacobson, K. & Schaller, M. D.MAP kinases and cell migration. *J. Cell. Sci.* **117**, 4619–4628 (2004). 15371522 10.1242/jcs.01481

[54] Qiu, W. et al. PUMA-mediated intestinal epithelial apoptosis contributes to ulcerative colitis in humans and mice. *J. Clin. Invest.* **121**, 1722–1732 (2011). 21490394 10.1172/JCI42917 PMC3083802

[55] Vetere, A., Choudhary, A., Burns, S. M. & Wagner, B. K. Targeting the pancreatic β-cell to treat diabetes. *Nat. Rev. Drug Discov*. **13**, 278–289 (2014). 24525781 10.1038/nrd4231

[56] Lu, Q. L., Abel, P., Foster, C. S. & Lalani, E. N. *bcl-2*: role in epithelial differentiation and oncogenesis. *Hum. Pathol.* **27**, 102–110 (1996). 8617450 10.1016/s0046-8177(96)90362-7

[57] Hassanshahi, N., Masoumi, S. J., Mehrabani, D., Hashemi, S. S. & Zare M. The healing effect of Aloe vera gel on acetic acid-induced ulcerative colitis in rat. *Middle East. J. Dig. Dis.* **12**, 154–161 (2020). 33062220 10.34172/mejdd.2020.177 PMC7548089

[58] Gao, W. et al. Chlorogenic acid attenuates dextran sodium sulfate-induced ulcerative colitis in mice through MAPK/ERK/JNK pathway. *Biomed. Res. Int.* **2019**, 1–13 (2019). 10.1155/2019/6769789 PMC6500688 31139644

[59] Vukelić, I., Detel, D., Batičić, L., Potočnjak, I. & Domitrović, R. Luteolin ameliorates experimental colitis in mice through ERK-mediated suppression of inflammation, apoptosis and autophagy. *Food Chem. Toxicol.* **145**, 111680 (2020). 32783997 10.1016/j.fct.2020.111680

[60] Muro, P. et al. The emerging role of oxidative stress in inflammatory bowel disease. *Front. Endocrinol. (Lausanne*. **15**, 1390351 (2024). 39076514 10.3389/fendo.2024.1390351 PMC11284038

[61] Huang, X. L. et al. PI3K/Akt signaling pathway is involved in the pathogenesis of ulcerative colitis. *Inflamm. Res.* **60**, 727–734 (2011). 21442372 10.1007/s00011-011-0325-6

[62] Naito, Y., Takagi, T. & Yoshikawa, T. Neutrophil-dependent oxidative stress in ulcerative colitis. *J. Clin. Biochem. Nutr.* **41**, 18–26 (2007). 18392100 10.3164/jcbn.2007003 PMC2274988

[63] Lu, G., Wen, Z., Yu, L., Wang, C. & Gao, Y. HIF1A overexpression caused by etomidate activates PGK1-induced oxidative stress in postoperative cognitive dysfunction. *Brain Res.* **1841**, 149069 (2024). 38852658 10.1016/j.brainres.2024.149069

[64] Vallée, A. & &Lecarpentier, Y. Crosstalk between peroxisome proliferator-activated receptor gamma and the canonical WNT/β-catenin pathway in chronic inflammation and oxidative stress during carcinogenesis. *Front. Immunol.* **9**, 745 (2018). 29706964 10.3389/fimmu.2018.00745 PMC5908886

[65] Salar, R. K. & Seasotiya, L. Free radical scavenging activity, phenolic contents and phytochemical evaluation of different extracts of stem bark of *Butea monosperma* (Lam.) Kuntze. *Front. Life Sci.* **5**, 107–116 (2011).

