# Supplementary material for: Network pharmacology and molecular docking reveal multi-target mechanisms of Butea monosperma stem bark extract in ulcerative colitis
Source: Sci Rep. 2025 Nov 17;15:40302. doi: 10.1038/s41598-025-24091-8 (PMC12624123; doi:10.1038/s41598-025-24091-8)
Supplement: Supplementary file 1 — Supplementary Material 1 [file 41598_2025_24091_MOESM1_ESM.docx]

**Supplementary figures.**


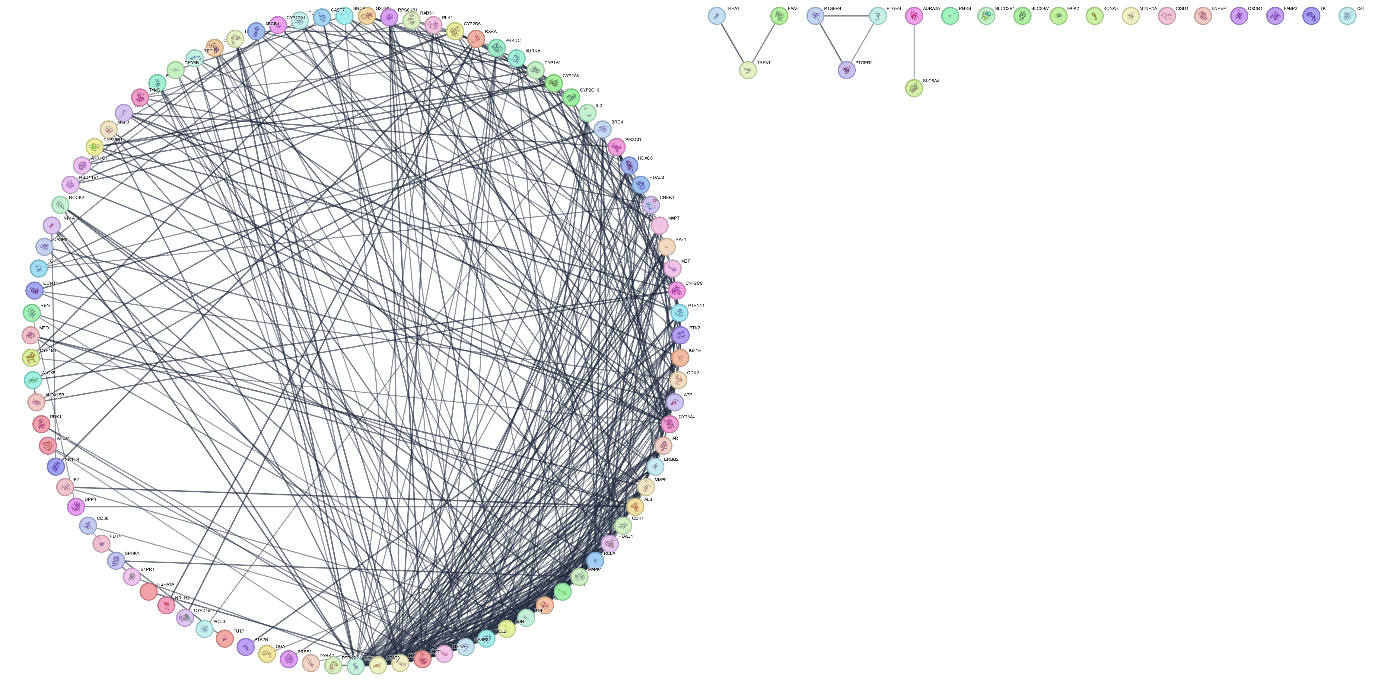


**Figure S1.** Visualization of the PPI network in Cytoscape.
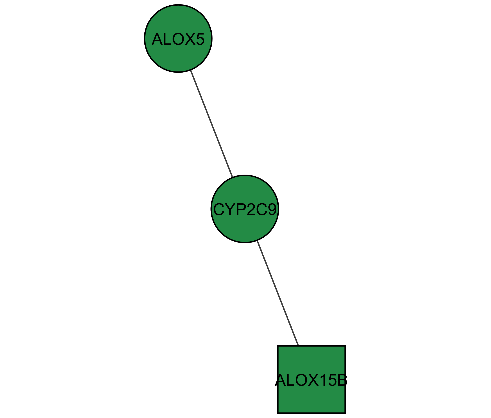


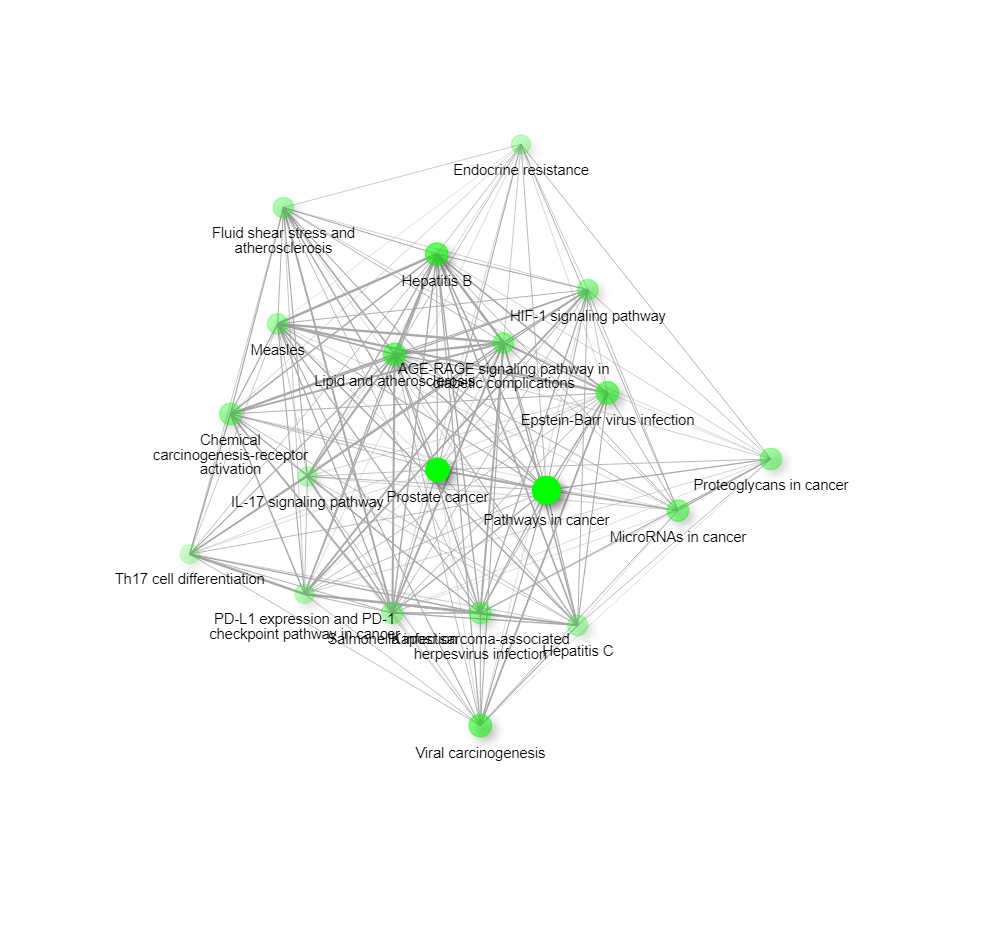


**Figure S2.** Hierarchical clustering tree of the KEGG enrichment analysis [36], [37], [38].

.


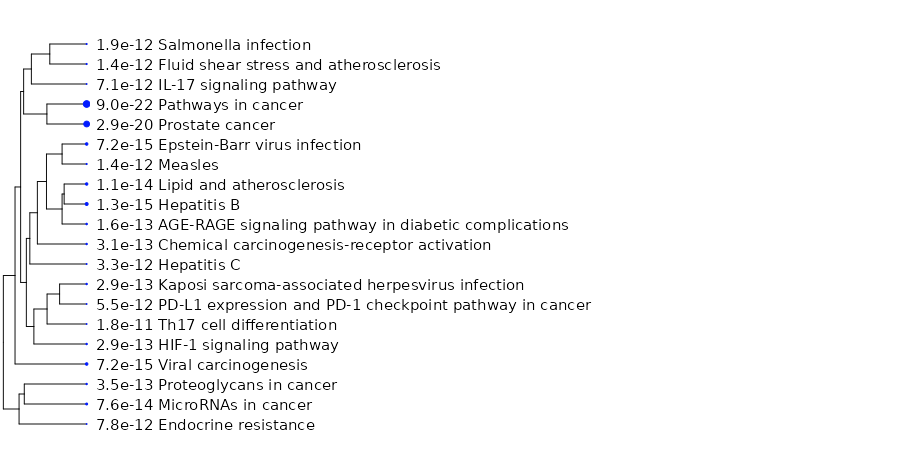


**Figure S3.** Tree tab of the KEGG enrichment analysis [36], [37], [38].

.


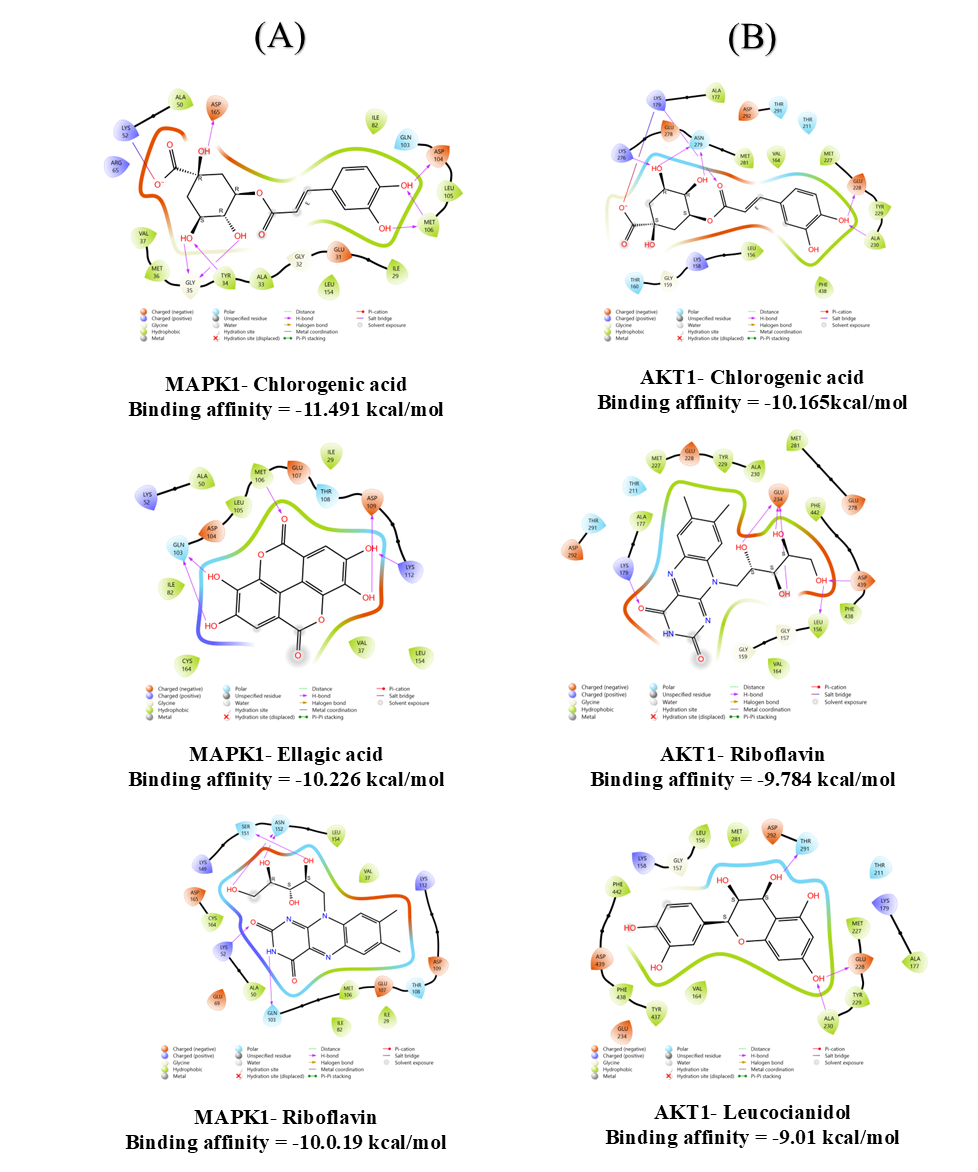


**Figure S4.** Molecular docking results (2D representation of the best active ingredients) of key targets i.e MAPK1 (A) and AKT1 (B), and specific active ingredients of *B. monosperma*.


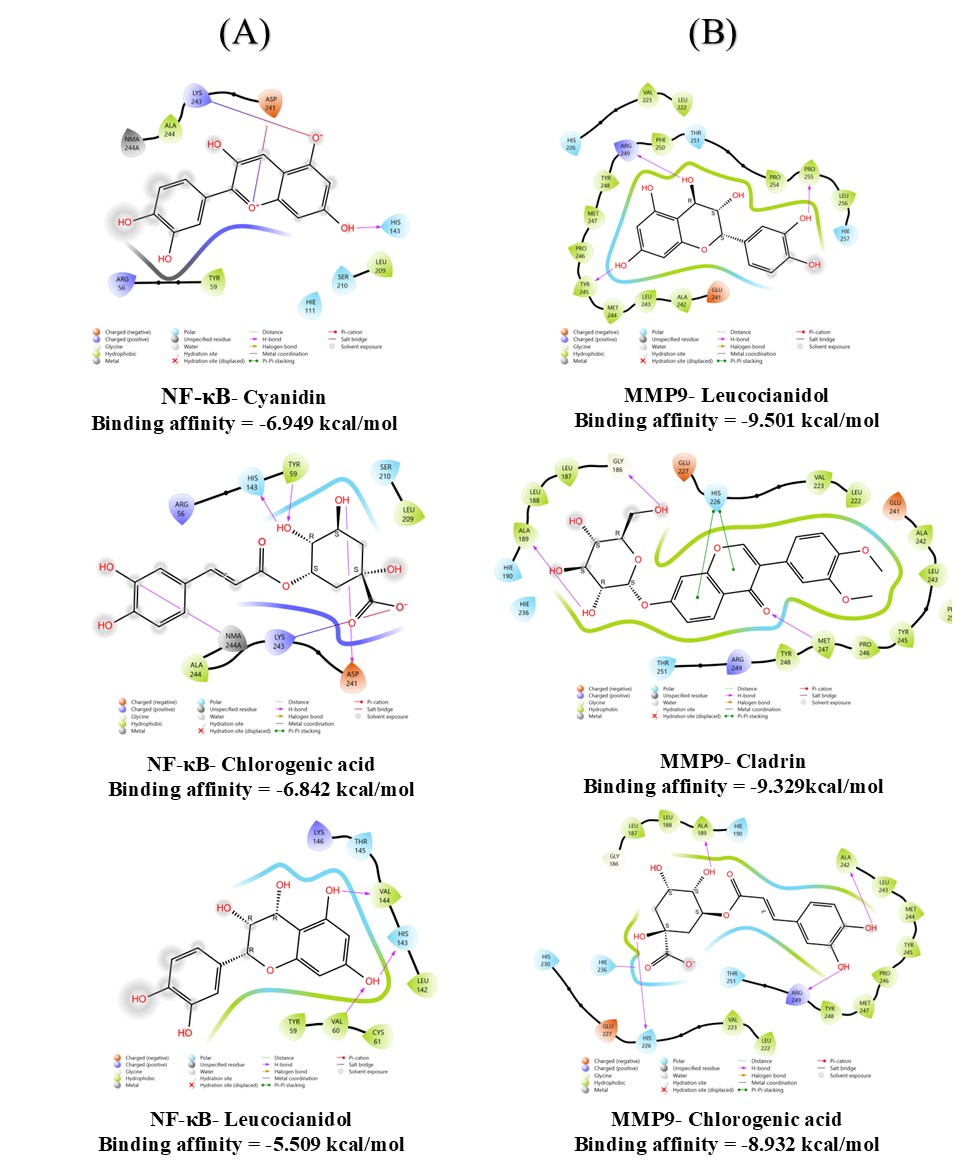


**Figure S5.** Molecular docking results (2D representation of the best active ingredients) of key targets i.e NF-κB (A) and MMP9 (B), and specific active ingredients of *B. monosperma*.


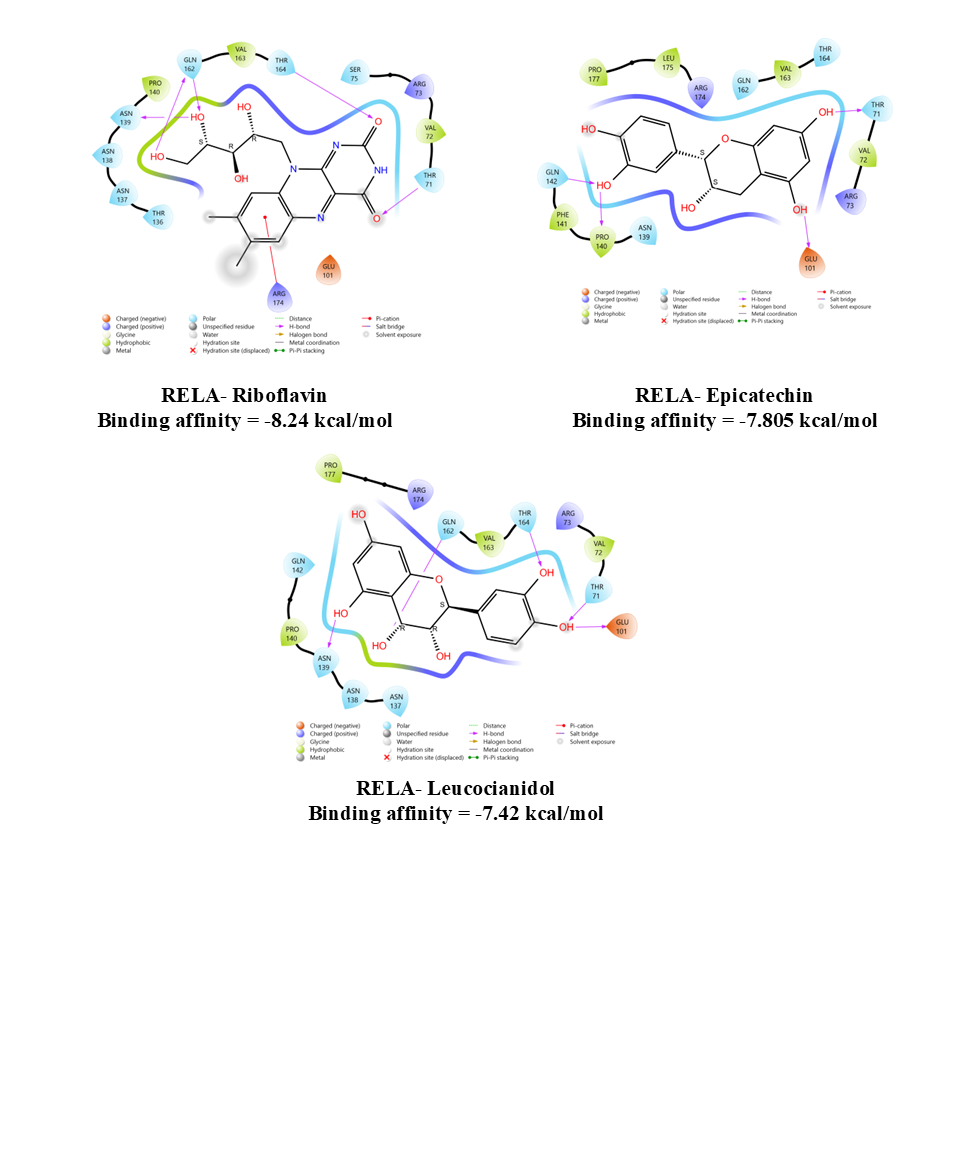


**Figure S6.** Molecular docking results (2D representation of the best active ingredients) of key target RELA and specific active ingredients of *B. monosperma*.


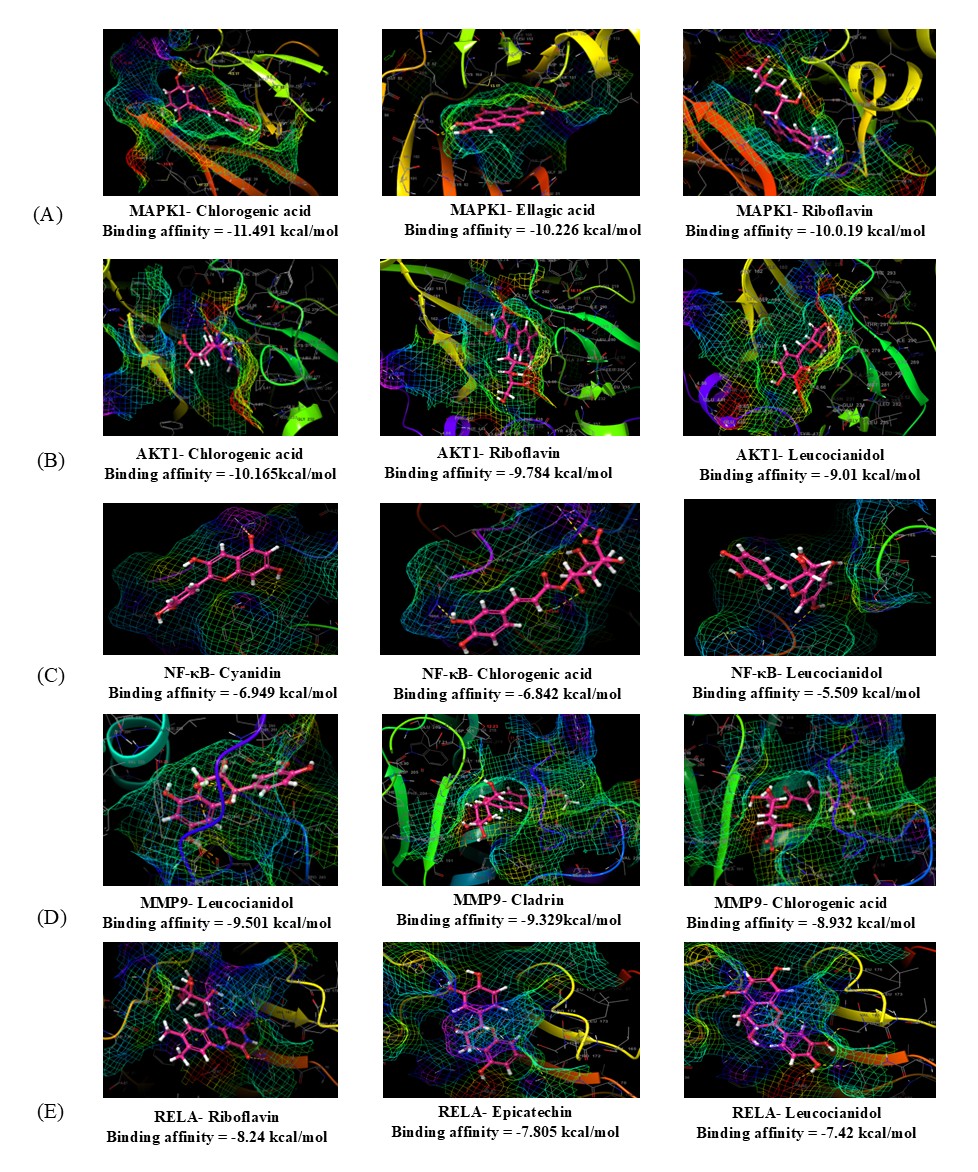


**Figure S7.** Molecular docking results (3D representation of the best active ingredients) of key targets (MAPK1, AKT1, NF-κB, MMP9 and RELA) and specific active ingredients of *B. monosperma*.

**
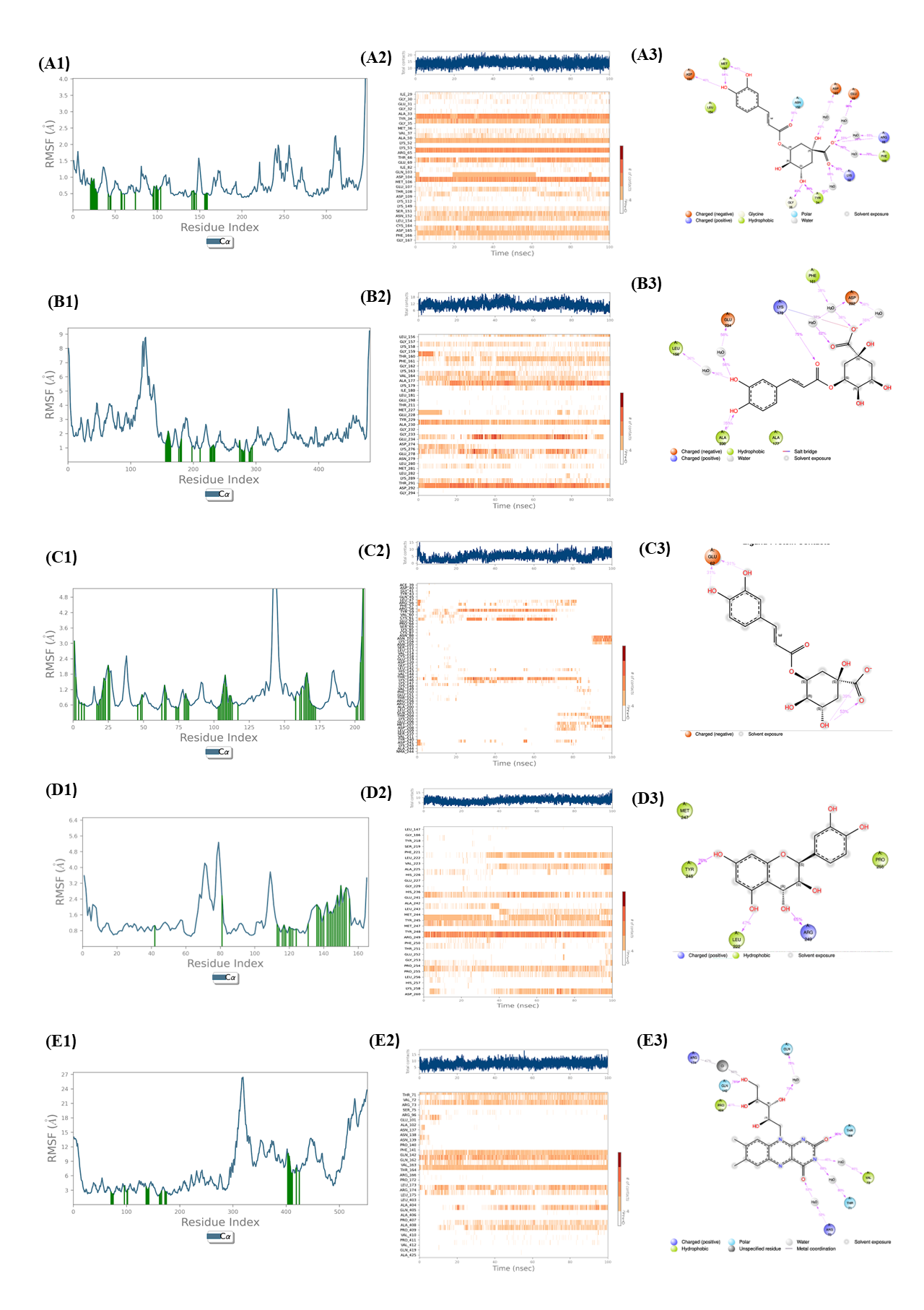
**

**Figure S8**. Molecular dynamics results of key target-ligands. RMSF and Protein-Ligands Contact Analysis of MAPK1-chlorogenic acid (A1, A2, A3), AKT1- chlorogenic acid (B1, B2, B3), NF-κB- Chlorogenic acid (C1, C2, C3), MMP9-leucocianidol (D1, D2, D3), RELA-riboflavin (E1, E2, E3) complexes.

**
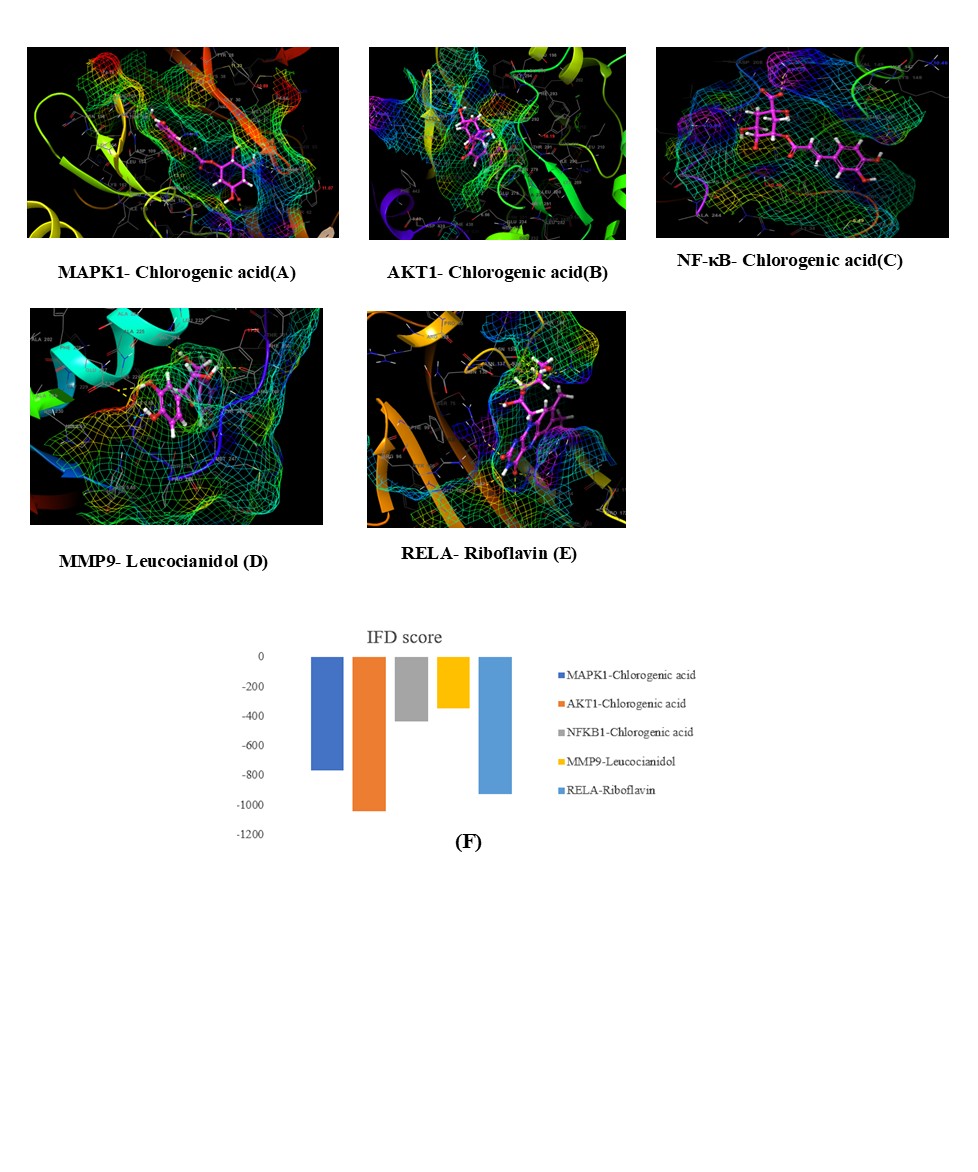
**

**Figure S9**. Induced Fit Docking results of key target-ligands. 3D representation of MAPK1-Chlorogenic acid (A), AKT1- Chlorogenic acid (B), NF-κB -Chlorogenic acid (C), MMP9-Leucocianidol (D), RELA-Riboflavin (E) complexes. Bar plot representation of the IFD score (F).

**
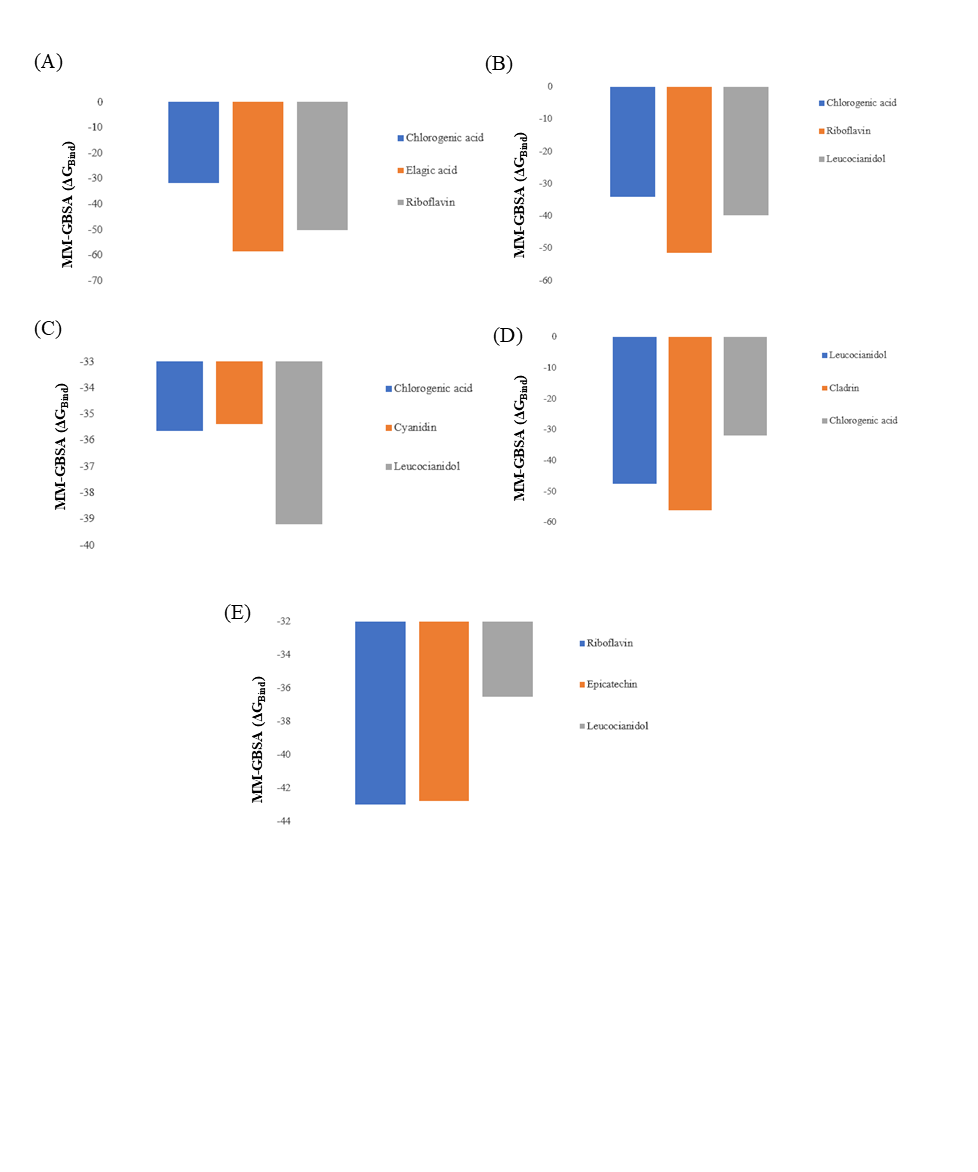
**

**Figure S10**. MM-GBSA (ΔG_Bind_) results (represented in a bar plot) of the best active ingredients and the key targets i.e MAPK1 (A), AKT1 (B), NF-κB (C), MMP9 (D) and RELA (E) and specific active ingredients of *B. monosperma.*

**Supplementary Tables.**

**Supplementary Table S1. Physicochemical constants**

| **Physicochemical constants** | **Value** | **API Limit (%)** |
| --- | --- | --- |
| **Ash value (%)** | 8.33 ± 0.624 | Not more than 12 |
| **Acid insoluble ash (%)** | 0.45 ± 0.15 | Not more than 1.5 |
| **Water soluble Extractive (%)** | 14.76 ± 0.750 | Not less than 14 |

Values are mean± S.D. (n=3)

**Supplementary Table S2. Fluorescence analysis of *B. monosperma* stem bark**

| **Reagent** | **Visible light** | **Short wavelength** | **Long wavelength** |
| --- | --- | --- | --- |
| Powder | brown | brown | brownish black |
| Powder + conc. HCL | dark brown | purplish black | black |
| Powder + conc. HNO3 | light brown | yellowish black | dark brown |
| Powder + 0.1 NaOH Sol. | dark brown | black | black |
| Powder + 1 % picric acid sol. | yellowish brown | black | greenish brown |

**Supplementary Table S3. Phytochemical composition of BME**

| **Compound** | **RT** | **Response** | **Conc.** | **Unit** |
| --- | --- | --- | --- | --- |
| Butin | 3.780 | 1317 | 74.00 | ng/ml |
| Caffeic acid | 3.890 | 169282 | 59.00 | ng/ml |
| Gallic Acid | 3.972 | 4080 | 82.00 | ng/ml |
| Catechin | 4.040 | 57327 | 181.00 | ng/ml |
| Medicarpin | 4.198 | 5833 | 161.00 | ng/ml |
| Lupeol | 4.216 | 4901 | 124.00 | ng/ml |
| Mirosterol | 4.344 | 20630 | 156.00 | ng/ml |
| Quercetin | 4.371 | 6279 | 23.00 | ng/ml |
| Buteaspermanol | 4.803 | 701291 | 125.00 | ng/ml |
| Ferulic Acid | 4.892 | 84710 | 123.00 | ng/ml |
| Butrin | 4.997 | 3389 | 71.00 | ng/ml |
| Sitosterol | 5.729 | 62484 | 176.00 | ng/ml |
| Riboflavin | 6.351 | 8236 | 132.00 | ng/ml |
| Thiamine | 6.448 | 79581 | 60.00 | ng/ml |
| Prunetin | 6.449 | 852 | 28.00 | ng/ml |
| Umbelliferone | 6.727 | 4714 | 93.00 | ng/ml |
| Epicatchin | 6.759 | 1269 | 176.00 | ng/ml |
| Coumaric acid | 7.602 | 36165 | 26.00 | ng/ml |
| shelloic acid | 8.490 | 19527 | 98.00 | ng/ml |
| kino-tannic acid | 8.612 | 9053 | 160.00 | ng/ml |
| pyrocatechin | 8.740 | 59175 | 84.00 | ng/ml |
| palsitrin | 8.746 | 211532 | 158.00 | ng/ml |
| palasitrin | 8.876 | 51918 | 35.00 | ng/ml |
| Isoformononetin | 9.196 | 62716 | 151.00 | ng/ml |
| alanine | 9.348 | 9302 | 188.00 | ng/ml |
| allophonic acid | 9.381 | 97335 | 85.00 | ng/ml |
| butolic acid | 9.533 | 120106 | 139.00 | ng/ml |
| histidin | 9.700 | 31096 | 92.00 | ng/ml |
| miroestrol | 9.736 | 23907 | 158.00 | ng/ml |
| palasimide | 9.807 | 86392 | 184.00 | ng/ml |
| cyanidin | 10.494 | 3421 | 125.00 | ng/ml |
| Leucocianidol | 11.117 | 108758 | 118.00 | ng/ml |
| beta-Sitosterol | 11.117 | 93165 | 85.00 | ng/ml |
| Ellagic acid | 11.177 | 6151 | 44.00 | ng/ml |
| Chlorogenic acid | 11.200 | 46381 | 185.00 | ng/ml |
| Cajanin | 11.888 | 579 | 22.00 | ng/ml |
| Cladrin | 12.042 | 38715 | 182.00 | ng/ml |
| Diadzein | 12.080 | 6458 | 77.00 | ng/ml |
| Kaempferol | 12.143 | 340668 | 127.00 | ng/ml |
| Rhamnetin | 12.312 | 260050 | 63.00 | ng/ml |
| Morin | 13.793 | 117622 | 112.00 | ng/ml |
| Dimethyl Quercetin | 13.909 | 64958 | 151.00 | ng/ml |
| Luteolin | 13.910 | 13642 | 91.00 | ng/ml |

**Supplementary Table S4. Physicochemical Properties of the phytoconstituents**

| **Compound** | **Molecular Weight** | **Nunber of HBA** | **Number of HBD** | **Mol Log P** |
| --- | --- | --- | --- | --- |
| Butin | 257.11 | 3 | 1 | 2.68 |
| Butrin | 596.17 | 15 | 9 | -2.3 |
| Gallic acid | 170.02 | 5 | 4 | 0.78 |
| Catechin | 290.08 | 6 | 5 | 0.53 |
| Medicarpin | 270.09 | 4 | 1 | 3.06 |
| Lupeol | 426.39 | 1 | 1 | 8.35 |
| Lupenone | 424.37 | 1 | 0 | 8.42 |
| Mirosterol | 358.39 | 6 | 4 | 0.98 |
| Quercetin | 302.04 | 7 | 5 | 1.19 |
| Cajanin | 300.06 | 6 | 3 | 2.57 |
| Cladrin | 460.14 | 10 | 4 | -0.23 |
| Diadzein | 254.06 | 4 | 2 | 1.97 |
| Kaempferol | 286.05 | 6 | 4 | 1.61 |
| Rhamnetin | 316.06 | 7 | 4 | 1.62 |
| Isoformonoretin | 268.26 | 4 | 1 | 2.65 |
| Buteaspermanol | 564.52 | 13 | 7 | -1.63 |
| Ferulic acid | 194.06 | 4 | 2 | 1.61 |
| Caffeic acid | 180.04 | 4 | 3 | 1.27 |
| Morin | 316.06 | 7 | 4 | 1.62 |
| Palasimide | 516.14 | 12 | 6 | -0.23 |
| Luteolin | 286.24 | 6 | 4 | 1.73 |
| Sitosterol | 416.72 | 1 | 1 | 7.07 |
| Riboflavin | 376.36 | 8 | 5 | -0.32 |
| Thiamine | 265.35 | 3 | 2 | 0.53 |
| Prunetin | 284.26 | 5 | 2 | 2.43 |
| Leucocianidol | 306.27 | 7 | 6 | 0.07 |
| Beta-Sitosterol | 414.71 | 1 | 1 | 7.19 |
| Ellagic acid | 302.19 | 8 | 4 | 1.00 |
| Chlorogenic acid | 354.31 | 9 | 6 | -0.38 |
| Umbelliferone | 162.14 | 3 | 1 | 1.51 |
| Epicatchin | 290.27 | 6 | 5 | 0.85 |
| Coumaric acid | 164.16 | 3 | 2 | 1.26 |
| Kinotannic acid | 1701.21 | 46 | 25 | 4.84 |
| Palasitrin | 594.52 | 15 | 9 | -2.63 |
| Alanine | 89.09 | 3 | 2 | -1.46 |
| Allophonic acid | 118.09 | 3 | 2 | -0.75 |
| Butolic acid | 244.37 | 3 | 2 | 3.48 |
| Cyanidin | 287.24 | 6 | 5 | 0.32 |
| Shelloic acid | 296.32 | 6 | 4 | 0.49 |
| Pyrocatechin | 188.16 | 5 | 2 | 0.38 |
| Isobutrin | 596.54 | 15 | 10 | - 2.65 |
| Histidine | 155.15 | 4 | 3 | -1.54 |
| Dimethyl quercetin | 257.11 | 3 | 1 | 2.68 |

**Supplementary Table S5. Details about the active ingredients of involved in the treatment of ulcerative colitis**

| **Phytoconstituent** | **Pubchem CID** |
| --- | --- |
| Butin | 92775 |
| Gallic Acid | 370 |
| Catechin | 73160 |
| Medicarpin | 336327 |
| Lupeol | 259846 |
| Lupenone | 92158 |
| Quercetin | 5280343 |
| Cajanin | 5281706 |
| Cladrin | 5748605 |
| Daidzin | 5281708 |
| Kaempferol | 5280863 |
| Rhamnetin | 5281691 |
| Isoformononetin | 3764 |
| Ferulic Acid | 445858 |
| Caffeic Acid | 689043 |
| Morin | 5281670 |
| Dimethyl Quercetin | 5280417 |
| Luteolin | 5280445 |
| Sitosterol | 3084097 |
| Riboflavin | 493570 |
| Thiamine | 1130 |
| Prunetin | 5281804 |
| Leucocianidol | 440833 |
| beta-Sitosterol | 222284 |
| Ellagic acid | 5281855 |
| Chlorogenic acid | 1794427 |
| Umbelliferone | 5281426 |
| Epicatchin | 72276 |
| Coumaric acid | 637542 |
| kino tannic acid |  |
| Histidine | 6274 |
| alanine | 5950 |
| allophonic acid |  |
| butolic acid |  |
| cyanidin | 128861 |
| shelloic acid | 20055026 |
| pyrocatechin | 129810396 |

**Supplementary Table S6. Results of BindingDB Database**

| **Target Name** | **Uploaded compounds generating hits** | **Max Similarity** | **Hits (All Compounds)** | **Phytoconstituent** |
| --- | --- | --- | --- | --- |
| 5-hydroxytryptamine receptor 1A | 1 | 0.7 | 1 | Butin |
| 6-phosphogluconate dehydrogenase, decarboxylating | 1 | 0.7 | 2 | Butin |
| A disintegrin and metalloproteinase with thrombospondin motifs 4 | 1 | 0.7 | 2 | Butin |
| A disintegrin and metalloproteinase with thrombospondin motifs 5 | 1 | 0.7 | 2 | Butin |
| Aldo-keto reductase family 1 member C3 | 1 | 0.79 | 1 | Butin |
| Alkaline phosphatase, tissue-nonspecific isozyme | 1 | 0.72 | 1 | Butin |
| Alpha-(1,3)-fucosyltransferase 7 | 1 | 0.7 | 1 | Butin |
| Alpha-1A adrenergic receptor | 1 | 0.7 | 1 | Butin |
| Alpha-1B adrenergic receptor | 1 | 0.7 | 1 | Butin |
| Alpha-1D adrenergic receptor | 1 | 0.7 | 1 | Butin |
| Alpha-synuclein | 1 | 0.72 | 1 | Butin |
| Amine oxidase [flavin-containing] B | 1 | 0.74 | 19 | Butin |
| Amyloid-beta precursor protein | 1 | 0.7 | 1 | Butin |
| Androgen receptor | 1 | 0.79 | 1 | Butin |
| Apoptosis regulator Bcl-2 | 1 | 0.7 | 4 | Butin |
| Aromatase | 1 | 0.92 | 9 | Butin |
| ATP-dependent translocase ABCB1 | 1 | 0.7 | 11 | Butin |
| Beta-secretase 1 | 1 | 0.7 | 3 | Butin |
| Carbonic anhydrase 1 | 1 | 0.92 | 1 | Butin |
| Carbonic anhydrase 12 | 1 | 0.72 | 4 | Butin |
| Carbonic anhydrase 2 | 1 | 0.92 | 1 | Butin |
| Carbonic anhydrase 4 | 1 | 0.97 | 3 | Butin |
| Carbonic anhydrase 7 | 1 | 0.72 | 5 | Butin |
| Casein kinase I isoform epsilon | 1 | 0.7 | 1 | Butin |
| Cathepsin L2 | 1 | 0.7 | 1 | Butin |
| Collagenase 3 | 1 | 0.74 | 1 | Butin |
| Cyclin-dependent kinase 4/G1/S-specific cyclin-D1 | 1 | 0.7 | 2 | Butin |
| Cytochrome P450 1B1 | 1 | 0.95 | 2 | Butin |
| D(2) dopamine receptor | 1 | 0.71 | 7 | Butin |
| Dipeptidyl peptidase 4 | 1 | 0.97 | 2 | Butin |
| DNA (cytosine-5)-methyltransferase 1 | 1 | 0.7 | 1 | Butin |
| DNA repair protein RAD52 homolog | 1 | 0.7 | 1 | Butin |
| Dual specificity protein kinase CLK1 | 1 | 0.7 | 1 | Butin |
| Dual specificity protein kinase CLK4 | 1 | 0.7 | 1 | Butin |
| Dual specificity tyrosine-phosphorylation-regulated kinase 1A | 1 | 0.7 | 8 | Butin |
| Dual specificity tyrosine-phosphorylation-regulated kinase 1B | 1 | 0.7 | 1 | Butin |
| ELAV-like protein 3 | 1 | 0.7 | 1 | Butin |
| Endothelin receptor type B | 1 | 0.7 | 2 | Butin |
| Estrogen receptor | 1 | 0.78 | 23 | Butin |
| Estrogen receptor beta | 1 | 0.92 | 18 | Butin |
| Group 10 secretory phospholipase A2 | 1 | 0.7 | 1 | Butin |
| Heat shock protein HSP 90-alpha | 1 | 0.7 | 2 | Butin |
| Heat shock protein HSP 90-beta | 1 | 0.99 | 1 | Butin |
| Hepatocyte growth factor receptor | 1 | 0.7 | 1 | Butin |
| Histamine H3 receptor | 1 | 0.7 | 1 | Butin |
| MAP kinase-activated protein kinase 5 | 1 | 0.7 | 1 | Butin |
| Metabotropic glutamate receptor 5 | 1 | 0.71 | 8 | Butin |
| NAD kinase | 1 | 0.7 | 1 | Butin |
| Peroxisome proliferator-activated receptor gamma | 1 | 0.7 | 3 | Butin |
| Phosphatidylinositol 4,5-bisphosphate 3-kinase catalytic subunit alpha isoform | 1 | 0.7 | 1 | Butin |
| Phosphoglycerate mutase 1 | 1 | 0.7 | 1 | Butin |
| Phospholipase A2 group V | 1 | 0.7 | 1 | Butin |
| Phospholipase A2, membrane associated | 1 | 0.7 | 5 | Butin |
| Plasminogen activator inhibitor 1 | 1 | 0.7 | 1 | Butin |
| Polyunsaturated fatty acid 5-lipoxygenase | 1 | 0.76 | 5 | Butin |
| Prostaglandin G/H synthase 1 | 1 | 0.72 | 1 | Butin |
| Proteasome subunit beta type-5 | 1 | 0.7 | 4 | Butin |
| Pyruvate kinase PKM | 1 | 0.7 | 3 | Butin |
| RAC-alpha serine/threonine-protein kinase | 1 | 0.7 | 1 | Butin |
| Receptor-interacting serine/threonine-protein kinase 1 | 1 | 0.79 | 1 | Butin |
| Retinoic acid receptor RXR-alpha | 1 | 0.7 | 2 | Butin |
| Serine/threonine-protein kinase mTOR | 1 | 0.7 | 1 | Butin |
| Sex hormone-binding globulin | 1 | 0.92 | 1 | Butin |
| Signal transducer and activator of transcription 1-alpha/beta | 1 | 0.7 | 1 | Butin |
| Solute carrier organic anion transporter family member 2B1 | 1 | 0.74 | 1 | Butin |
| Telomerase reverse transcriptase | 1 | 0.7 | 1 | Butin |
| Transcription factor p65 | 1 | 0.71 | 1 | Butin |
| Transthyretin | 1 | 0.8 | 6 | Butin |
| Tyrosinase | 1 | 0.88 | 3 | Butin |
| Tyrosine-protein kinase receptor UFO | 1 | 0.92 | 1 | Butin |
| Tyrosine-protein phosphatase non-receptor type 1 | 1 | 0.78 | 1 | Butin |
| Voltage-dependent T-type calcium channel subunit alpha-1G | 1 | 0.79 | 3 | Butin |
| Voltage-dependent T-type calcium channel subunit alpha-1H | 1 | 0.75 | 9 | Butin |
| X-box-binding protein 1 | 1 | 0.78 | 1 | Butin |
| Xanthine dehydrogenase/oxidase | 1 | 0.75 | 1 | Butin |
| Aldo-keto reductase family 1 member C3 | 1 | 0.7 | 1 | Gallic Acid |
| Alpha-(1,3)-fucosyltransferase 7 | 1 | 1 | 1 | Gallic Acid |
| Amyloid-beta precursor protein | 1 | 1 | 1 | Gallic Acid |
| Carbonic anhydrase 1 | 1 | 0.75 | 7 | Gallic Acid |
| Carbonic anhydrase 12 | 1 | 0.7 | 6 | Gallic Acid |
| Carbonic anhydrase 14 | 1 | 0.96 | 8 | Gallic Acid |
| Carbonic anhydrase 2 | 1 | 0.75 | 9 | Gallic Acid |
| Carbonic anhydrase 7 | 1 | 0.7 | 6 | Gallic Acid |
| Carbonic anhydrase 9 | 1 | 0.85 | 5 | Gallic Acid |
| D(1A) dopamine receptor | 1 | 0.75 | 1 | Gallic Acid |
| M18 aspartyl aminopeptidase | 1 | 0.96 | 1 | Gallic Acid |
| Plasminogen activator inhibitor 1 | 1 | 0.71 | 1 | Gallic Acid |
| Polypeptide N-acetylgalactosaminyltransferase 2 | 1 | 1 | 1 | Gallic Acid |
| Transthyretin | 1 | 0.71 | 1 | Gallic Acid |
| Urokinase-type plasminogen activator | 1 | 0.71 | 1 | Gallic Acid |
| 6-phosphogluconate dehydrogenase, decarboxylating | 1 | 0.78 | 2 | Catechin |
| 72 kDa type IV collagenase | 1 | 0.82 | 1 | Catechin |
| A disintegrin and metalloproteinase with thrombospondin motifs 4 | 1 | 0.78 | 2 | Catechin |
| Alkaline phosphatase, tissue-nonspecific isozyme | 1 | 1 | 1 | Catechin |
| Alpha-(1,3)-fucosyltransferase 7 | 1 | 0.78 | 1 | Catechin |
| Alpha-synuclein | 1 | 1 | 1 | Catechin |
| Amyloid-beta precursor protein | 1 | 0.78 | 1 | Catechin |
| Apoptosis regulator Bcl-2 | 1 | 0.8 | 6 | Catechin |
| Aromatase | 1 | 0.7 | 3 | Catechin |
| ATP-dependent translocase ABCB1 | 1 | 0.72 | 32 | Catechin |
| Beta-secretase 1 | 1 | 0.78 | 3 | Catechin |
| Broad substrate specificity ATP-binding cassette transporter ABCG2 | 1 | 0.7 | 1 | Catechin |
| Carbonic anhydrase 1 | 1 | 0.7 | 1 | Catechin |
| Carbonic anhydrase 12 | 1 | 0.73 | 4 | Catechin |
| Carbonic anhydrase 2 | 1 | 0.7 | 1 | Catechin |
| Carbonic anhydrase 4 | 1 | 0.72 | 3 | Catechin |
| Carbonic anhydrase 7 | 1 | 1 | 5 | Catechin |
| Casein kinase I isoform epsilon | 1 | 0.78 | 1 | Catechin |
| Collagenase 3 | 1 | 0.71 | 1 | Catechin |
| Cytochrome P450 1B1 | 1 | 0.71 | 2 | Catechin |
| Dipeptidyl peptidase 4 | 1 | 0.72 | 2 | Catechin |
| DNA (cytosine-5)-methyltransferase 1 | 1 | 0.78 | 1 | Catechin |
| DNA repair protein RAD52 homolog | 1 | 0.78 | 1 | Catechin |
| Dual specificity protein kinase CLK1 | 1 | 0.78 | 1 | Catechin |
| Dual specificity protein kinase CLK4 | 1 | 0.78 | 1 | Catechin |
| Dual specificity tyrosine-phosphorylation-regulated kinase 1A | 1 | 0.78 | 22 | Catechin |
| Dual specificity tyrosine-phosphorylation-regulated kinase 1B | 1 | 0.78 | 1 | Catechin |
| ELAV-like protein 3 | 1 | 0.78 | 2 | Catechin |
| Estrogen receptor | 1 | 0.7 | 6 | Catechin |
| Estrogen receptor beta | 1 | 0.7 | 7 | Catechin |
| Fibroblast growth factor receptor 1 | 1 | 0.76 | 1 | Catechin |
| Glucose-6-phosphate 1-dehydrogenase | 1 | 0.78 | 4 | Catechin |
| Heat shock protein HSP 90-beta | 1 | 0.73 | 1 | Catechin |
| Hepatocyte growth factor receptor | 1 | 0.76 | 1 | Catechin |
| MAP kinase-activated protein kinase 5 | 1 | 0.78 | 1 | Catechin |
| Mast/stem cell growth factor receptor Kit | 1 | 0.76 | 1 | Catechin |
| Multidrug resistance-associated protein 1 | 1 | 0.7 | 1 | Catechin |
| NAD kinase | 1 | 0.78 | 1 | Catechin |
| Phosphatidylinositol 4,5-bisphosphate 3-kinase catalytic subunit alpha isoform | 1 | 0.78 | 1 | Catechin |
| Phosphoglycerate mutase 1 | 1 | 0.75 | 2 | Catechin |
| Placenta growth factor | 1 | 0.76 | 3 | Catechin |
| Plasminogen activator inhibitor 1 | 1 | 0.74 | 2 | Catechin |
| Polyunsaturated fatty acid lipoxygenase ALOX12 | 1 | 0.7 | 1 | Catechin |
| Proteasome subunit beta type-5 | 1 | 0.78 | 4 | Catechin |
| Prothrombin | 1 | 0.7 | 1 | Catechin |
| Proto-oncogene tyrosine-protein kinase Src | 1 | 0.76 | 1 | Catechin |
| Pyruvate kinase PKM | 1 | 0.78 | 2 | Catechin |
| RAC-alpha serine/threonine-protein kinase | 1 | 0.7 | 1 | Catechin |
| Serine/threonine-protein kinase mTOR | 1 | 0.78 | 1 | Catechin |
| Sex hormone-binding globulin | 1 | 0.7 | 1 | Catechin |
| Signal transducer and activator of transcription 1-alpha/beta | 1 | 0.78 | 1 | Catechin |
| Solute carrier organic anion transporter family member 2B1 | 1 | 0.71 | 1 | Catechin |
| Telomerase reverse transcriptase | 1 | 0.78 | 1 | Catechin |
| Transthyretin | 1 | 0.78 | 1 | Catechin |
| Tyrosine-protein kinase receptor UFO | 1 | 0.7 | 1 | Catechin |
| Vascular endothelial growth factor A | 1 | 0.76 | 3 | Catechin |
| Amine oxidase [flavin-containing] B | 1 | 0.82 | 1 | Medicarpin |
| Estrogen receptor | 1 | 0.73 | 7 | Medicarpin |
| Estrogen receptor beta | 1 | 0.73 | 6 | Medicarpin |
| Eukaryotic initiation factor 4A-I | 1 | 0.7 | 3 | Medicarpin |
| Albumin | 1 | 0.73 | 1 | Lupeol |
| Alpha-crystallin A chain | 1 | 0.7 | 1 | Lupeol |
| Alpha-crystallin B chain | 1 | 0.7 | 1 | Lupeol |
| Androgen receptor | 1 | 0.72 | 3 | Lupeol |
| Aromatase | 1 | 0.73 | 8 | Lupeol |
| Bile acid receptor | 1 | 0.7 | 1 | Lupeol |
| Delta(24)-sterol reductase | 1 | 0.7 | 1 | Lupeol |
| Estrogen receptor beta | 1 | 0.7 | 1 | Lupeol |
| G-protein coupled bile acid receptor 1 | 1 | 0.73 | 8 | Lupeol |
| Gag-Pol polyprotein [489-587] | 1 | 0.73 | 1 | Lupeol |
| Glutamate receptor ionotropic, NMDA 2A | 1 | 0.72 | 27 | Lupeol |
| Glutamate receptor ionotropic, NMDA 2B | 1 | 0.72 | 29 | Lupeol |
| Liver carboxylesterase 1 | 1 | 1 | 2 | Lupeol |
| Monoglyceride lipase | 1 | 0.7 | 1 | Lupeol |
| Nuclear receptor ROR-gamma | 1 | 0.7 | 1 | Lupeol |
| Oxysterols receptor LXR-alpha | 1 | 0.85 | 1 | Lupeol |
| RNA-binding protein FXR1 | 1 | 0.7 | 2 | Lupeol |
| Sex hormone-binding globulin | 1 | 0.72 | 4 | Lupeol |
| Tissue factor | 1 | 0.72 | 1 | Lupeol |
| Tyrosine-protein phosphatase non-receptor type 1 | 1 | 1 | 1 | Lupeol |
| Tyrosine-protein phosphatase non-receptor type 1 [1-405] | 1 | 0.73 | 1 | Lupeol |
| UDP-glucuronosyltransferase 2B7 | 1 | 0.7 | 2 | Lupeol |
| Aromatase | 1 | 0.7 | 23 | Lupenone |
| G-protein coupled bile acid receptor 1 | 1 | 0.73 | 1 | Lupenone |
| 6-phosphofructo-2-kinase/fructose-2,6-bisphosphatase 3 | 1 | 0.73 | 1 | Cajanin |
| Acetylcholinesterase | 1 | 0.76 | 1 | Cajanin |
| Aldehyde dehydrogenase, mitochondrial | 1 | 0.79 | 6 | Cajanin |
| Aldehyde oxidase | 1 | 0.93 | 25 | Cajanin |
| Amine oxidase [flavin-containing] A | 1 | 0.87 | 1 | Cajanin |
| Amine oxidase [flavin-containing] B | 1 | 0.7 | 3 | Cajanin |
| Aromatase | 1 | 0.7 | 6 | Cajanin |
| ATP-dependent translocase ABCB1 | 1 | 0.88 | 1 | Cajanin |
| Beta-secretase 1 | 1 | 0.75 | 3 | Cajanin |
| Carbonic anhydrase 12 | 1 | 0.7 | 1 | Cajanin |
| Carbonic anhydrase 4 | 1 | 0.88 | 2 | Cajanin |
| Carbonic anhydrase 7 | 1 | 0.83 | 1 | Cajanin |
| Cytochrome P450 3A4 | 1 | 0.88 | 2 | Cajanin |
| D(1B) dopamine receptor | 1 | 0.7 | 1 | Cajanin |
| Dipeptidyl peptidase 4 | 1 | 0.88 | 1 | Cajanin |
| Dual specificity mitogen-activated protein kinase kinase 4 | 1 | 0.87 | 1 | Cajanin |
| Epidermal growth factor receptor | 1 | 0.87 | 1 | Cajanin |
| Estrogen receptor | 1 | 0.87 | 3 | Cajanin |
| Estrogen receptor beta | 1 | 0.87 | 2 | Cajanin |
| Genome polyprotein | 1 | 0.87 | 3 | Cajanin |
| Histamine H3 receptor | 1 | 0.7 | 2 | Cajanin |
| Isoform 1 of Steroid hormone receptor ERR2 (ERRbeta2-delta10) | 1 | 0.75 | 3 | Cajanin |
| MAP kinase-interacting serine/threonine-protein kinase 2 | 1 | 0.87 | 1 | Cajanin |
| Neuraminidase | 1 | 0.7 | 1 | Cajanin |
| Polyunsaturated fatty acid 5-lipoxygenase | 1 | 0.7 | 1 | Cajanin |
| Receptor-type tyrosine-protein phosphatase S | 1 | 0.7 | 4 | Cajanin |
| Smoothened homolog | 1 | 0.81 | 1 | Cajanin |
| Sphingosine 1-phosphate receptor 1 | 1 | 0.76 | 1 | Cajanin |
| Steroid hormone receptor ERR1 | 1 | 0.85 | 1 | Cajanin |
| Tyrosine-protein phosphatase non-receptor type 1 | 1 | 0.87 | 1 | Cajanin |
| Isoform 1 of Steroid hormone receptor ERR2 (ERRbeta2-delta10) | 1 | 0.7 | 2 | Cajanin |
| MAP kinase-interacting serine/threonine-protein kinase 2 | 1 | 0.73 | 1 | Cajanin |
| Neuraminidase | 1 | 0.76 | 1 | Cajanin |
| Polyunsaturated fatty acid 5-lipoxygenase | 1 | 0.79 | 6 | Cajanin |
| Receptor-type tyrosine-protein phosphatase S | 1 | 0.93 | 25 | Cajanin |
| Smoothened homolog | 1 | 0.87 | 1 | Cajanin |
| Sphingosine 1-phosphate receptor 1 | 1 | 0.7 | 3 | Cajanin |
| Steroid hormone receptor ERR1 | 1 | 0.7 | 6 | Cajanin |
| Tyrosine-protein phosphatase non-receptor type 1 | 1 | 0.88 | 1 | Cajanin |
| 17-beta-hydroxysteroid dehydrogenase type 1 | 1 | 0.88 | 1 | Kaempferol |
| 17-beta-hydroxysteroid dehydrogenase type 2 | 1 | 1 | 1 | Kaempferol |
| Acetylcholinesterase | 1 | 0.8 | 12 | Kaempferol |
| Adenosine receptor A2a | 1 | 0.97 | 1 | Kaempferol |
| Adenosine receptor A3 | 1 | 0.7 | 6 | Kaempferol |
| Aldo-keto reductase family 1 member B1 | 1 | 0.76 | 33 | Kaempferol |
| Aldo-keto reductase family 1 member C3 | 1 | 0.75 | 1 | Kaempferol |
| Alpha-synuclein | 1 | 0.94 | 1 | Kaempferol |
| Amine oxidase [flavin-containing] A | 1 | 0.8 | 10 | Kaempferol |
| Amine oxidase [flavin-containing] B | 1 | 0.82 | 10 | Kaempferol |
| Androgen receptor | 1 | 0.75 | 8 | Kaempferol |
| Aromatase | 1 | 0.77 | 12 | Kaempferol |
| Aryl hydrocarbon receptor | 1 | 0.71 | 5 | Kaempferol |
| ATP-dependent translocase ABCB1 | 1 | 0.7 | 22 | Kaempferol |
| Aurora kinase B | 1 | 0.94 | 1 | Kaempferol |
| BDNF/NT-3 growth factors receptor | 1 | 0.81 | 1 | Kaempferol |
| Beta-secretase 1 | 1 | 0.94 | 6 | Kaempferol |
| Broad substrate specificity ATP-binding cassette transporter ABCG2 | 1 | 0.73 | 11 | Kaempferol |
| Bromodomain-containing protein 4 | 1 | 0.82 | 1 | Kaempferol |
| Carbonic anhydrase 12 | 1 | 0.89 | 5 | Kaempferol |
| Carbonic anhydrase 2 | 1 | 0.89 | 2 | Kaempferol |
| Carbonic anhydrase 4 | 1 | 0.86 | 4 | Kaempferol |
| Carbonic anhydrase 7 | 1 | 0.89 | 5 | Kaempferol |
| Carbonyl reductase [NADPH] 1 | 1 | 0.85 | 1 | Kaempferol |
| Casein kinase II subunit alpha | 1 | 0.91 | 2 | Kaempferol |
| Casein kinase II subunit alpha 3 | 1 | 0.86 | 6 | Kaempferol |
| Cathepsin L2 | 1 | 0.7 | 1 | Kaempferol |
| CDGSH iron-sulfur domain-containing protein 1 | 1 | 1 | 1 | Kaempferol |
| Cyclin homolog/dependent kinase 6 | 1 | 0.91 | 1 | Kaempferol |
| Cyclin-C | 1 | 0.7 | 5 | Kaempferol |
| Cyclin-dependent kinase 1 | 1 | 0.94 | 1 | Kaempferol |
| Cyclin-dependent kinase 2 | 1 | 0.73 | 1 | Kaempferol |
| Cyclin-dependent kinase 5 activator 1 [99-307] | 1 | 0.91 | 1 | Kaempferol |
| Cyclin-dependent kinase 6 | 1 | 0.91 | 1 | Kaempferol |
| Cyclin-dependent kinase 9 | 1 | 0.79 | 1 | Kaempferol |
| Cyclin-T1/Cyclin-dependent kinase 9 | 1 | 0.79 | 1 | Kaempferol |
| Cytochrome P450 1A1 | 1 | 0.71 | 52 | Kaempferol |
| Cytochrome P450 1A2 | 1 | 0.71 | 43 | Kaempferol |
| Cytochrome P450 1B1 | 1 | 0.71 | 55 | Kaempferol |
| Cytochrome P450 2D6 | 1 | 0.77 | 1 | Kaempferol |
| Cytochrome P450 4F2 | 1 | 0.7 | 1 | Kaempferol |
| Delta-type opioid receptor | 1 | 0.7 | 1 | Kaempferol |
| Dipeptidyl peptidase 4 | 1 | 0.73 | 6 | Kaempferol |
| Discoidin domain-containing receptor 2 | 1 | 0.7 | 1 | Kaempferol |
| DNA topoisomerase 2-alpha | 1 | 0.94 | 1 | Kaempferol |
| DNA-(apurinic or apyrimidinic site) endonuclease | 1 | 0.94 | 1 | Kaempferol |
| ELAV-like protein 3 | 1 | 0.94 | 1 | Kaempferol |
| Enoyl-acyl-carrier protein reductase | 1 | 0.91 | 3 | Kaempferol |
| Epidermal growth factor receptor | 1 | 0.87 | 2 | Kaempferol |
| Estrogen receptor | 1 | 0.71 | 1 | Kaempferol |
| Estrogen receptor beta | 1 | 0.73 | 3 | Kaempferol |
| Glycogen synthase kinase-3 beta | 1 | 0.86 | 2 | Kaempferol |
| Hepatocyte growth factor receptor |  | 0.94 | 1 | Kaempferol |
| Inositol hexakisphosphate kinase 2 | 1 | 0.94 | 4 | Kaempferol |
| Insulin-like growth factor 1 receptor | 1 | 0.94 | 1 | Kaempferol |
| Integrase | 1 | 0.92 | 8 | Kaempferol |
| Lactoylglutathione lyase | 1 | 0.94 | 1 | Kaempferol |
| Low molecular weight phosphotyrosine protein phosphatase | 1 | 0.7 | 1 | Kaempferol |
| Macrophage metalloelastase | 1 | 0.86 | 1 | Kaempferol |
| MAP kinase-interacting serine/threonine-protein kinase 1 | 1 | 0.86 | 1 | Kaempferol |
| MAP kinase-interacting serine/threonine-protein kinase 2 | 1 | 0.86 | 4 | Kaempferol |
| Mast/stem cell growth factor receptor Kit | 1 | 0.83 | 2 | Kaempferol |
| Matrix metalloproteinase-9 | 1 | 0.94 | 1 | Kaempferol |
| Muscleblind-like protein 1 | 1 | 0.94 | 1 | Kaempferol |
| NADPH oxidase 4 | 1 | 0.94 | 3 | Kaempferol |
| Nuclear receptor subfamily 0 group B member 1 | 1 | 0.84 | 1 | Kaempferol |
| PC4 and SFRS1-interacting protein | 1 | 0.94 | 2 | Kaempferol |
| Peroxisome proliferator-activated receptor alpha | 1 | 0.7 | 1 | Kaempferol |
| Peroxisome proliferator-activated receptor gamma | 1 | 0.7 | 2 | Kaempferol |
| Phosphatidylinositol 4,5-bisphosphate 3-kinase catalytic subunit gamma isoform | 1 | 0.94 | 2 | Kaempferol |
| Placenta growth factor | 1 | 0.7 | 1 | Kaempferol |
| Poly [ADP-ribose] polymerase 1 | 1 | 0.7 | 11 | Kaempferol |
| Poly [ADP-ribose] polymerase tankyrase-1 | 1 | 0.73 | 8 | Kaempferol |
| Poly [ADP-ribose] polymerase tankyrase-2 | 1 | 0.73 | 6 | Kaempferol |
| Polyphenol oxidase 2 | 1 | 0.82 | 2 | Kaempferol |
| Polyunsaturated fatty acid 5-lipoxygenase | 1 | 0.94 | 38 | Kaempferol |
| Polyunsaturated fatty acid lipoxygenase ALOX12 | 1 | 0.91 | 3 | Kaempferol |
| Polyunsaturated fatty acid lipoxygenase ALOX15 | 1 | 0.86 | 2 | Kaempferol |
| Polyunsaturated fatty acid lipoxygenase ALOX15B | 1 | 0.86 | 1 | Kaempferol |
| Potassium voltage-gated channel subfamily A member 3 | 1 | 0.7 | 1 | Kaempferol |
| Proteasome subunit beta type-5 | 1 | 0.88 | 1 | Kaempferol |
| Prothrombin | 1 | 0.86 | 2 | Kaempferol |
| Pyruvate kinase PKM | 1 | 0.88 | 3 | Kaempferol |
| Receptor-type tyrosine-protein kinase FLT3 | 1 | 0.86 | 6 | Kaempferol |
| Receptor-type tyrosine-protein phosphatase S | 1 | 0.72 | 3 | Kaempferol |
| Serine/threonine-protein kinase pim-1 | 1 | 0.92 | 8 | Kaempferol |
| Short transient receptor potential channel 5 | 1 | 0.97 | 2 | Kaempferol |
| Solute carrier organic anion transporter family member 2B1 | 1 | 0.89 | 1 | Kaempferol |
| Telomerase reverse transcriptase | 1 | 0.82 | 2 | Kaempferol |
| Thiosulfate sulfurtransferase | 1 | 0.89 | 1 | Kaempferol |
| Transthyretin | 1 | 0.88 | 2 | Kaempferol |
| Tyrosinase | 1 | 0.86 | 3 | Kaempferol |
| Tyrosine-protein kinase Lck | 1 | 0.72 | 1 | Kaempferol |
| Tyrosine-protein kinase receptor UFO | 1 | 0.94 | 1 | Kaempferol |
| Tyrosine-protein kinase Yes | 1 | 0.94 | 1 | Kaempferol |
| Tyrosine-protein phosphatase non-receptor type 1 | 1 | 0.7 | 1 | Kaempferol |
| Vascular endothelial growth factor A | 1 | 0.7 | 1 | Kaempferol |
| Vascular endothelial growth factor receptor 2 | 1 | 0.87 | 1 | Kaempferol |
| Xanthine dehydrogenase/oxidase | 1 | 0.94 | 14 | Kaempferol |
| 11-beta-hydroxysteroid dehydrogenase 1 | 1 | 0.75 | 1 | Ferulic Acid |
| 72 kDa type IV collagenase | 1 | 0.88 | 2 | Ferulic Acid |
| Aldo-keto reductase family 1 member B1 | 1 | 0.73 | 2 | Ferulic Acid |
| Aldo-keto reductase family 1 member B10 | 1 | 0.7 | 1 | Ferulic Acid |
| Alpha-synuclein | 1 | 1 | 1 | Ferulic Acid |
| Amyloid-beta precursor protein | 1 | 0.79 | 1 | Ferulic Acid |
| ATP-dependent translocase ABCB1 | 1 | 0.7 | 1 | Ferulic Acid |
| Beta-secretase 1 | 1 | 0.7 | 1 | Ferulic Acid |
| Broad substrate specificity ATP-binding cassette transporter ABCG2 | 1 | 0.7 | 1 | Ferulic Acid |
| Carbonic anhydrase 1 | 1 | 0.7 | 2 | Ferulic Acid |
| Carbonic anhydrase 12 | 1 | 0.7 | 2 | Ferulic Acid |
| Carbonic anhydrase 13 | 1 | 0.7 | 3 | Ferulic Acid |
| Carbonic anhydrase 14 | 1 | 0.7 | 5 | Ferulic Acid |
| Carbonic anhydrase 2 | 1 | 0.73 | 4 | Ferulic Acid |
| Carbonic anhydrase 5A, mitochondrial | 1 | 0.7 | 3 | Ferulic Acid |
| Carbonic anhydrase 5B, mitochondrial | 1 | 0.74 | 3 | Ferulic Acid |
| Carbonic anhydrase 6 | 1 | 0.7 | 3 | Ferulic Acid |
| Carbonic anhydrase 7 | 1 | 0.7 | 5 | Ferulic Acid |
| Carbonic anhydrase 9 | 1 | 0.7 | 4 | Ferulic Acid |
| Caspase-1 | 1 | 0.7 | 1 | Ferulic Acid |
| Cytochrome P450 1A1 | 1 | 0.7 | 4 | Ferulic Acid |
| Cytochrome P450 1A2 | 1 | 0.7 | 1 | Ferulic Acid |
| Cytochrome P450 1B1 | 1 | 0.7 | 1 | Ferulic Acid |
| Cytochrome P450 2D6 | 1 | 0.75 | 1 | Ferulic Acid |
| Interstitial collagenase | 1 | 0.88 | 1 | Ferulic Acid |
| Matrix metalloproteinase-9 | 1 | 0.88 | 1 | Ferulic Acid |
| Melatonin receptor type 1A | 1 | 0.7 | 1 | Ferulic Acid |
| Nuclear factor erythroid 2-related factor 2 | 1 | 0.75 | 1 | Ferulic Acid |
| Polyunsaturated fatty acid 5-lipoxygenase | 1 | 0.7 | 6 | Ferulic Acid |
| Prostaglandin G/H synthase 1 | 1 | 0.7 | 1 | Ferulic Acid |
| Prostaglandin G/H synthase 2 | 1 | 0.7 | 1 | Ferulic Acid |
| Tissue factor | 1 | 0.75 | 1 | Ferulic Acid |
| Transcription factor p65 | 1 | 0.7 | 2 | Ferulic Acid |
| Tubulin beta-1 chain | 1 | 0.7 | 2 | Ferulic Acid |
| Tubulin beta-3 chain | 1 | 0.7 | 1 | Ferulic Acid |
| Tubulin polymerization-promoting protein | 1 | 0.7 | 1 | Ferulic Acid |
| Type-1 angiotensin II receptor | 1 | 0.88 | 1 | Ferulic Acid |
| 11-beta-hydroxysteroid dehydrogenase 1 | 1 | 0.7 | 3 | Sitosterol |
| 17-beta-hydroxysteroid dehydrogenase type 3 | 1 | 0.73 | 18 | Sitosterol |
| Aldo-keto reductase family 1 member B10 | 1 | 0.75 | 2 | Sitosterol |
| Androgen receptor | 1 | 0.73 | 6 | Sitosterol |
| Aromatase | 1 | 0.73 | 1 | Sitosterol |
| Bile acid receptor | 1 | 0.7 | 8 | Sitosterol |
| Carbonic anhydrase 2 | 1 | 0.7 | 2 | Sitosterol |
| Estrogen receptor beta | 1 | 0.93 | 1 | Sitosterol |
| G-protein coupled bile acid receptor 1 | 1 | 0.72 | 22 | Sitosterol |
| Gamma-aminobutyric acid receptor subunit alpha-1/beta-2/gamma-2 | 1 | 0.73 | 4 | Sitosterol |
| Gamma-aminobutyric acid receptor subunit alpha-1/beta-3/gamma-2 | 1 | 0.71 | 1 | Sitosterol |
| Gamma-aminobutyric acid receptor subunit alpha-4/beta-3/delta | 1 | 0.73 | 1 | Sitosterol |
| Glucocorticoid receptor | 1 | 0.73 | 1 | Sitosterol |
| Glucose-6-phosphate 1-dehydrogenase | 1 | 0.71 | 2 | Sitosterol |
| Glutamate receptor ionotropic, NMDA 2A | 1 | 0.98 | 27 | Sitosterol |
| Glutamate receptor ionotropic, NMDA 2B | 1 | 0.98 | 29 | Sitosterol |
| Isoform 2 of Nuclear receptor ROR-alpha (Alpha-2) | 1 | 0.78 | 1 | Sitosterol |
| Liver carboxylesterase 1 | 1 | 0.71 | 1 | Sitosterol |
| Mineralocorticoid receptor | 1 | 0.73 | 1 | Sitosterol |
| Oxysterols receptor LXR-alpha | 1 | 0.77 | 1 | Sitosterol |
| Progesterone receptor | 1 | 0.73 | 1 | Sitosterol |
| RNA-binding protein FXR1 | 1 | 0.71 | 3 | Sitosterol |
| Sex hormone-binding globulin | 1 | 0.73 | 12 | Sitosterol |
| Steroidogenic factor 1 | 1 | 0.78 | 1 | Sitosterol |
| Tyrosine-protein phosphatase non-receptor type 1 | 1 | 0.71 | 1 | Sitosterol |
| UDP-glucuronosyltransferase 2B7 | 1 | 0.73 | 8 | Sitosterol |
| Vitamin D3 receptor | 1 | 0.75 | 3 | Sitosterol |
| Beta-nerve growth factor | 1 | 0.7 | 1 | Riboflavin |
| Thiosulfate sulfurtransferase | 1 | 0.89 | 1 | Riboflavin |
| Carbonic anhydrase 1 | 1 | 1 | 1 | Thiamine |
| Carbonic anhydrase 2 | 1 | 1 | 2 | Thiamine |
| Carbonic anhydrase 6 | 1 | 1 | 2 | Thiamine |
| Transketolase | 1 | 0.74 | 22 | Thiamine |
| 6-phosphofructo-2-kinase/fructose-2,6-bisphosphatase 3 | 1 | 0.8 | 1 | Prunetin |
| Acetylcholinesterase | 1 | 0.83 | 11 | Prunetin |
| Aldehyde dehydrogenase, mitochondrial | 1 | 1 | 28 | Prunetin |
| Aldehyde oxidase | 1 | 0.94 | 1 | Prunetin |
| Amine oxidase [flavin-containing] A | 1 | 0.7 | 3 | Prunetin |
| Amine oxidase [flavin-containing] B | 1 | 0.7 | 8 | Prunetin |
| Aromatase | 1 | 0.95 | 1 | Prunetin |
| ATP-dependent translocase ABCB1 | 1 | 0.78 | 3 | Prunetin |
| Carbonic anhydrase 12 | 1 | 0.95 | 2 | Prunetin |
| Carbonic anhydrase 4 | 1 | 0.9 | 1 | Prunetin |
| Carbonic anhydrase 7 | 1 | 0.95 | 2 | Prunetin |
| Cholinesterase | 1 | 0.7 | 3 | Prunetin |
| D(1B) dopamine receptor | 1 | 0.95 | 1 | Prunetin |
| Dipeptidyl peptidase 4 | 1 | 0.94 | 1 | Prunetin |
| Dual specificity mitogen-activated protein kinase kinase 4 | 1 | 0.94 | 1 | Prunetin |
| Epidermal growth factor receptor | 1 | 0.94 | 4 | Prunetin |
| Estrogen receptor | 1 | 0.94 | 2 | Prunetin |
| Estrogen receptor beta | 1 | 0.94 | 4 | Prunetin |
| Histamine H3 receptor | 1 | 0.79 | 5 | Prunetin |
| Isoform 1 of Steroid hormone receptor ERR2 (ERRbeta2-delta10) | 1 | 0.94 | 1 | Prunetin |
| Macrophage migration inhibitory factor | 1 | 0.75 | 5 | Prunetin |
| MAP kinase-interacting serine/threonine-protein kinase 2 | 1 | 0.7 | 1 | Prunetin |
| Polyunsaturated fatty acid 5-lipoxygenase | 1 | 0.71 | 7 | Prunetin |
| Polyunsaturated fatty acid lipoxygenase ALOX12 | 1 | 0.7 | 4 | Prunetin |
| Polyunsaturated fatty acid lipoxygenase ALOX15 | 1 | 0.72 | 1 | Prunetin |
| Receptor-type tyrosine-protein phosphatase S | 1 | 0.83 | 1 | Prunetin |
| Sphingosine 1-phosphate receptor 1 | 1 | 0.91 | 1 | Prunetin |
| Steroid hormone receptor ERR1 | 1 | 0.94 | 1 | Prunetin |
| Steroidogenic factor 1 | 1 | 0.74 | 1 | Prunetin |
| Tyrosine-protein phosphatase non-receptor type 1 | 1 | 0.7 | 2 | Prunetin |
| 6-phosphogluconate dehydrogenase, decarboxylating | 1 | 0.75 | 2 | Leucocianidol |
| 72 kDa type IV collagenase | 1 | 0.78 | 1 | Leucocianidol |
| A disintegrin and metalloproteinase with thrombospondin motifs 4 | 1 | 0.75 | 2 | Leucocianidol |
| A disintegrin and metalloproteinase with thrombospondin motifs 5 | 1 | 0.75 | 2 | Leucocianidol |
| Alkaline phosphatase, tissue-nonspecific isozyme | 1 | 0.94 | 1 | Leucocianidol |
| Alpha-(1,3)-fucosyltransferase 7 | 1 | 0.75 | 1 | Leucocianidol |
| Alpha-synuclein | 1 | 0.94 | 1 | Leucocianidol |
| Amyloid-beta precursor protein | 1 | 0.75 | 1 | Leucocianidol |
| Apoptosis regulator Bcl-2 | 1 | 0.78 | 6 | Leucocianidol |
| ATP-dependent translocase ABCB1 | 1 | 0.7 | 29 | Leucocianidol |
| Beta-secretase 1 | 1 | 0.75 | 3 | Leucocianidol |
| Broad substrate specificity ATP-binding cassette transporter ABCG2 | 1 | 0.7 | 1 | Leucocianidol |
| Carbonic anhydrase 12 | 1 | 0.77 | 3 | Leucocianidol |
| Carbonic anhydrase 4 | 1 | 0.7 | 2 | Leucocianidol |
| Carbonic anhydrase 7 | 1 | 0.94 | 4 | Leucocianidol |
| Casein kinase I isoform epsilon | 1 | 0.75 | 1 | Leucocianidol |
| Collagenase 3 | 1 | 0.72 | 1 | Leucocianidol |
| Cytochrome P450 1B1 | 1 | 0.7 | 2 | Leucocianidol |
| Dipeptidyl peptidase 4 | 1 | 0.7 | 1 | Leucocianidol |
| DNA (cytosine-5)-methyltransferase 1 | 1 | 0.75 | 1 | Leucocianidol |
| DNA repair protein RAD52 homolog | 1 | 0.75 | 1 | Leucocianidol |
| Dual specificity protein kinase CLK1 | 1 | 0.75 | 1 | Leucocianidol |
| Dual specificity protein kinase CLK4 | 1 | 0.75 | 1 | Leucocianidol |
| Dual specificity tyrosine-phosphorylation-regulated kinase 1A | 1 | 0.75 | 19 | Leucocianidol |
| Dual specificity tyrosine-phosphorylation-regulated kinase 1B | 1 | 0.75 | 1 | Leucocianidol |
| ELAV-like protein 3 | 1 | 0.75 | 2 | Leucocianidol |
| Estrogen receptor | 1 | 0.71 | 1 | Leucocianidol |
| Estrogen receptor beta | 1 | 0.71 | 1 | Leucocianidol |
| Fibroblast growth factor receptor 1 | 1 | 0.79 | 1 | Leucocianidol |
| Heat shock protein HSP 90-beta | 1 | 0.7 | 1 | Leucocianidol |
| Hepatocyte growth factor receptor | 1 | 0.79 | 1 | Leucocianidol |
| MAP kinase-activated protein kinase 5 | 1 | 0.75 | 1 | Leucocianidol |
| Mast/stem cell growth factor receptor Kit | 1 | 0.79 | 1 | Leucocianidol |
| Multidrug resistance-associated protein 1 | 1 | 0.7 | 1 | Leucocianidol |
| NAD kinase | 1 | 0.75 | 1 | Leucocianidol |
| Phosphatidylinositol 4,5-bisphosphate 3-kinase catalytic subunit alpha isoform | 1 | 0.75 | 1 | Leucocianidol |
| Phosphoglycerate mutase 1 | 1 | 0.72 | 2 | Leucocianidol |
| Placenta growth factor | 1 | 0.72 | 3 | Leucocianidol |
| Plasminogen activator inhibitor 1 | 1 | 0.71 | 2 | Leucocianidol |
| Polyunsaturated fatty acid lipoxygenase ALOX12 | 1 | 0.7 | 1 | Leucocianidol |
| Proteasome subunit beta type-5 | 1 | 0.75 | 4 | Leucocianidol |
| Prothrombin | 1 | 0.74 | 1 | Leucocianidol |
| Proto-oncogene tyrosine-protein kinase Src | 1 | 0.79 | 1 | Leucocianidol |
| Pyruvate kinase PKM | 1 | 0.75 | 2 | Leucocianidol |
| Serine/threonine-protein kinase mTOR | 1 | 0.75 | 1 | Leucocianidol |
| Signal transducer and activator of transcription 1-alpha/beta | 1 | 0.75 | 1 | Leucocianidol |
| Solute carrier organic anion transporter family member 2B1 | 1 | 0.72 | 1 | Leucocianidol |
| Telomerase reverse transcriptase | 1 | 0.75 | 1 | Leucocianidol |
| Transthyretin | 1 | 0.75 | 1 | Leucocianidol |
| Tyrosine-protein kinase receptor UFO | 1 | 0.7 | 1 | Leucocianidol |
| Vascular endothelial growth factor A | 1 | 0.72 | 3 | Leucocianidol |
| 3-hydroxy-3-methylglutaryl-coenzyme A reductase | 1 | 0.88 | 8 | beta-Sitosterol |
| Alpha-crystallin A chain | 1 | 0.81 | 1 | beta-Sitosterol |
| Alpha-crystallin B chain | 1 | 0.7 | 4 | beta-Sitosterol |
| Androgen receptor | 1 | 0.8 | 30 | beta-Sitosterol |
| Aromatase | 1 | 0.83 | 32 | beta-Sitosterol |
| Bile acid receptor | 1 | 0.7 | 1 | beta-Sitosterol |
| Coagulation factor X | 1 | 0.82 | 1 | beta-Sitosterol |
| Delta(24)-sterol reductase | 1 | 0.98 | 3 | beta-Sitosterol |
| Estrogen receptor | 1 | 0.91 | 40 | beta-Sitosterol |
| Estrogen receptor beta | 1 | 0.7 | 45 | beta-Sitosterol |
| G-protein coupled receptor 183 | 1 | 0.88 | 4 | beta-Sitosterol |
| Glutamate receptor ionotropic, NMDA 1/2A | 1 | 0.84 | 30 | beta-Sitosterol |
| Glutamate receptor ionotropic, NMDA 1/2B | 1 | 0.84 | 30 | beta-Sitosterol |
| Glutamate receptor ionotropic, NMDA 1/2C | 1 | 0.84 | 1 | beta-Sitosterol |
| Glutamate receptor ionotropic, NMDA 1/2D | 1 | 0.84 | 1 | beta-Sitosterol |
| Glutamate receptor ionotropic, NMDA 2A | 1 | 0.98 | 58 | beta-Sitosterol |
| Glutamate receptor ionotropic, NMDA 2B | 1 | 0.98 | 62 | beta-Sitosterol |
| Integrin alpha-V/beta-3 | 1 | 1 | 1 | beta-Sitosterol |
| Nuclear receptor ROR-gamma | 1 | 0.7 | 7 | beta-Sitosterol |
| Oxysterol-binding protein 2 | 1 | 1 | 2 | beta-Sitosterol |
| Oxysterol-binding protein 2 [1-181,275-916] | 1 | 1 | 2 | beta-Sitosterol |
| Oxysterols receptor LXR-alpha | 1 | 0.75 | 15 | beta-Sitosterol |
| Oxysterols receptor LXR-beta | 1 | 0.75 | 3 | beta-Sitosterol |
| Prothrombin | 1 | 1 | 1 | beta-Sitosterol |
| Sex hormone-binding globulin | 1 | 0.97 | 11 | beta-Sitosterol |
| Steroid 17-alpha-hydroxylase/17,20 lyase | 1 | 0.8 | 13 | beta-Sitosterol |
| Sterol regulatory element-binding protein 2 | 1 | 0.81 | 9 | beta-Sitosterol |
| Tyrosine-protein phosphatase non-receptor type 1 | 1 | 0.71 | 4 | beta-Sitosterol |
| Tyrosine-protein phosphatase non-receptor type 1 [1-405] | 1 | 0.74 | 1 | beta-Sitosterol |
| Tyrosine-protein phosphatase non-receptor type 2 | 1 | 0.71 | 4 | beta-Sitosterol |
| Vitamin D3 receptor | 1 | 0.7 | 2 | beta-Sitosterol |
| Aldo-keto reductase family 1 member B1 | 1 | 1 | 1 | Ellagic acid |
| Amyloid-beta precursor protein | 1 | 1 | 1 | Ellagic acid |
| Angiopoietin-1 receptor | 1 | 1 | 1 | Ellagic acid |
| Carbonic anhydrase 1 | 1 | 0.7 | 1 | Ellagic acid |
| Carbonic anhydrase 12 | 1 | 0.7 | 3 | Ellagic acid |
| Carbonic anhydrase 9 | 1 | 0.7 | 7 | Ellagic acid |
| Casein kinase II subunit alpha | 1 | 1 | 1 | Ellagic acid |
| D-amino-acid oxidase | 1 | 0.7 | 1 | Ellagic acid |
| DNA polymerase eta | 1 | 1 | 1 | Ellagic acid |
| DNA polymerase iota | 1 | 1 | 1 | Ellagic acid |
| DNA-(apurinic or apyrimidinic site) endonuclease | 1 | 0.77 | 1 | Ellagic acid |
| Dual specificity protein phosphatase 3 | 1 | 1 | 1 | Ellagic acid |
| ELAV-like protein 3 | 1 | 1 | 1 | Ellagic acid |
| Epidermal growth factor receptor | 1 | 1 | 1 | Ellagic acid |
| Estrogen receptor | 1 | 0.7 | 1 | Ellagic acid |
| Estrogen receptor beta | 1 | 0.7 | 1 | Ellagic acid |
| G-protein coupled receptor 35 | 1 | 1 | 1 | Ellagic acid |
| Heat shock 70 kDa protein 1A | 1 | 1 | 1 | Ellagic acid |
| Hepatocyte growth factor receptor | 1 | 1 | 1 | Ellagic acid |
| Insulin receptor | 1 | 1 | 1 | Ellagic acid |
| Insulin-like growth factor 1 receptor | 1 | 1 | 1 | Ellagic acid |
| Mothers against decapentaplegic homolog 3 | 1 | 1 | 1 | Ellagic acid |
| NUAK family SNF1-like kinase 1 | 1 | 1 | 1 | Ellagic acid |
| PC4 and SFRS1-interacting protein | 1 | 0.77 | 1 | Ellagic acid |
| Polypeptide N-acetylgalactosaminyltransferase 10 | 1 | 0.88 | 1 | Ellagic acid |
| Polypeptide N-acetylgalactosaminyltransferase 14 | 1 | 0.88 | 1 | Ellagic acid |
| Polypeptide N-acetylgalactosaminyltransferase 2 | 1 | 1 | 2 | Ellagic acid |
| Proto-oncogene tyrosine-protein kinase Src | 1 | 1 | 1 | Ellagic acid |
| Solute carrier family 22 member 6 | 1 | 1 | 1 | Ellagic acid |
| Sphingosine kinase 1 | 1 | 1 | 1 | Ellagic acid |
| Vascular endothelial growth factor receptor 2 | 1 | 1 | 1 | Ellagic acid |
| Aldo-keto reductase family 1 member B1 | 1 | 0.99 | 4 | Chlorogenic acid |
| Aldo-keto reductase family 1 member B10 | 1 | 0.99 | 2 | Chlorogenic acid |
| Amyloid-beta precursor protein | 1 | 0.96 | 2 | Chlorogenic acid |
| Heat shock protein HSP 90-beta | 1 | 0.82 | 1 | Chlorogenic acid |
| Histone deacetylase | 1 | 1 | 1 | Chlorogenic acid |
| Methionine aminopeptidase 2 | 1 | 0.7 | 1 | Chlorogenic acid |
| Neutrophil elastase | 1 | 0.73 | 1 | Chlorogenic acid |
| Protein kinase C alpha type | 1 | 0.7 | 3 | Chlorogenic acid |
| Short transient receptor potential channel 1/4 | 1 | 0.7 | 1 | Chlorogenic acid |
| Short transient receptor potential channel 5 | 1 | 0.7 | 2 | Chlorogenic acid |
| Tyrosine-protein phosphatase non-receptor type 1 | 1 | 1 | 1 | Chlorogenic acid |
| 72 kDa type IV collagenase | 1 | 1 | 1 | Caffeic Acid |
| Aldo-keto reductase family 1 member B1 | 1 | 0.84 | 3 | Caffeic Acid |
| Aldo-keto reductase family 1 member B10 | 1 | 0.7 | 3 | Caffeic Acid |
| Alpha-synuclein | 1 | 0.88 | 1 | Caffeic Acid |
| Carbonic anhydrase 1 | 1 | 0.76 | 3 | Caffeic Acid |
| Carbonic anhydrase 12 | 1 | 0.76 | 3 | Caffeic Acid |
| Carbonic anhydrase 14 | 1 | 0.76 | 3 | Caffeic Acid |
| Carbonic anhydrase 2 | 1 | 0.84 | 5 | Caffeic Acid |
| Carbonic anhydrase 7 | 1 | 0.76 | 3 | Caffeic Acid |
| Carbonic anhydrase 9 | 1 | 0.76 | 3 | Caffeic Acid |
| Caspase-1 | 1 | 0.7 | 1 | Caffeic Acid |
| Histone deacetylase | 1 | 0.7 | 1 | Caffeic Acid |
| Interstitial collagenase | 1 | 1 | 1 | Caffeic Acid |
| Lactoylglutathione lyase | 1 | 0.7 | 1 | Caffeic Acid |
| Matrix metalloproteinase-9 | 1 | 1 | 1 | Caffeic Acid |
| Poly [ADP-ribose] polymerase tankyrase-1/tankyrase-2 | 1 | 0.7 | 1 | Caffeic Acid |
| Polyphenol oxidase 2 | 1 | 0.7 | 2 | Caffeic Acid |
| Polyunsaturated fatty acid 5-lipoxygenase | 1 | 0.7 | 3 | Caffeic Acid |
| Polyunsaturated fatty acid lipoxygenase ALOX15 | 1 | 0.7 | 1 | Caffeic Acid |
| Type-1 angiotensin II receptor | 1 | 1 | 1 | Caffeic Acid |
| 17-beta-hydroxysteroid dehydrogenase type 1 | 1 | 0.83 | 1 | Morin |
| 17-beta-hydroxysteroid dehydrogenase type 2 | 1 | 0.94 | 1 | Morin |
| Acetylcholinesterase | 1 | 0.76 | 10 | Morin |
| Adenosine receptor A2a | 1 | 0.92 | 1 | Morin |
| Adenosine receptor A3 | 1 | 0.7 | 6 | Morin |
| Aldo-keto reductase family 1 member B1 | 1 | 0.75 | 32 | Morin |
| Aldo-keto reductase family 1 member C3 | 1 | 0.79 | 1 | Morin |
| Alpha-synuclein | 1 | 0.9 | 1 | Morin |
| Amine oxidase [flavin-containing] A | 1 | 0.76 | 8 | Morin |
| Amine oxidase [flavin-containing] B | 1 | 0.78 | 4 | Morin |
| Androgen receptor | 1 | 0.79 | 6 | Morin |
| Aromatase | 1 | 0.73 | 12 | Morin |
| ATP-dependent translocase ABCB1 | 1 | 0.7 | 19 | Morin |
| Aurora kinase B | 1 | 0.9 | 1 | Morin |
| BDNF/NT-3 growth factors receptor | 1 | 0.79 | 1 | Morin |
| Beta-secretase 1 | 1 | 0.9 | 5 | Morin |
| Broad substrate specificity ATP-binding cassette transporter ABCG2 | 1 | 0.7 | 9 | Morin |
| Bromodomain-containing protein 4 | 1 | 0.8 | 1 | Morin |
| Carbonic anhydrase 12 | 1 | 0.85 | 5 | Morin |
| Carbonic anhydrase 2 | 1 | 0.85 | 2 | Morin |
| Carbonic anhydrase 4 | 1 | 0.81 | 4 | Morin |
| Carbonic anhydrase 7 | 1 | 0.85 | 5 | Morin |
| Carbonyl reductase [NADPH] 1 | 1 | 0.8 | 1 | Morin |
| Casein kinase II subunit alpha | 1 | 0.86 | 2 | Morin |
| Casein kinase II subunit alpha 3 | 1 | 0.81 | 6 | Morin |
| Cathepsin L2 | 1 | 0.7 | 1 | Morin |
| CDGSH iron-sulfur domain-containing protein 1 | 1 | 0.94 | 1 | Morin |
| Cyclin homolog/dependent kinase 6 | 1 | 0.86 | 1 | Morin |
| Cyclin-C | 1 | 0.7 | 5 | Morin |
| Cyclin-dependent kinase 1 | 1 | 0.9 | 1 | Morin |
| Cyclin-dependent kinase 2 | 1 | 0.71 | 1 | Morin |
| Cyclin-dependent kinase 5 activator 1 [99-307] | 1 | 0.86 | 1 | Morin |
| Cyclin-dependent kinase 6 | 1 | 0.86 | 1 | Morin |
| Cyclin-dependent kinase 9 | 1 | 0.77 | 1 | Morin |
| Cyclin-T1/Cyclin-dependent kinase 9 | 1 | 0.77 | 1 | Morin |
| Cytochrome P450 1A1 | 1 | 0.7 | 43 | Morin |
| Cytochrome P450 1A2 | 1 | 0.7 | 36 | Morin |
| Cytochrome P450 1B1 | 1 | 0.7 | 49 | Morin |
| Cytochrome P450 2D6 | 1 | 0.74 | 1 | Morin |
| Delta-type opioid receptor | 1 | 0.7 | 1 | Morin |
| Dipeptidyl peptidase 4 | 1 | 0.7 | 6 | Morin |
| DNA topoisomerase 2-alpha | 1 | 0.9 | 1 | Morin |
| DNA-(apurinic or apyrimidinic site) endonuclease | 1 | 0.9 | 1 | Morin |
| ELAV-like protein 3 | 1 | 0.9 | 1 | Morin |
| Enoyl-acyl-carrier protein reductase | 1 | 0.86 | 3 | Morin |
| Epidermal growth factor receptor | 1 | 0.83 | 2 | Morin |
| Estrogen receptor | 1 | 0.7 | 1 | Morin |
| Estrogen receptor beta | 1 | 0.7 | 3 | Morin |
| Glycogen synthase kinase-3 beta | 1 | 0.81 | 2 | Morin |
| Hepatocyte growth factor receptor | 1 | 0.9 | 1 | Morin |
| Inositol hexakisphosphate kinase 2 | 1 | 0.9 | 4 | Morin |
| Insulin-like growth factor 1 receptor | 1 | 0.9 | 1 | Morin |
| Lactoylglutathione lyase | 1 | 0.9 | 1 | Morin |
| Macrophage metalloelastase | 1 | 0.81 | 1 | Morin |
| MAP kinase-interacting serine/threonine-protein kinase 1 | 1 | 0.81 | 1 | Morin |
| MAP kinase-interacting serine/threonine-protein kinase 2 | 1 | 0.81 | 4 | Morin |
| Mast/stem cell growth factor receptor Kit | 1 | 0.8 | 2 | Morin |
| Matrix metalloproteinase-9 | 1 | 0.9 | 1 | Morin |
| Mu-type opioid receptor | 1 | 0.77 | 1 | Morin |
| Muscleblind-like protein 1 | 1 | 0.9 | 1 | Morin |
| NADPH oxidase 4 | 1 | 0.9 | 3 | Morin |
| Nuclear receptor subfamily 0 group B member 1 | 1 | 0.81 | 1 | Morin |
| PC4 and SFRS1-interacting protein | 1 | 0.9 | 2 | Morin |
| Peroxisome proliferator-activated receptor gamma | 1 | 0.7 | 1 | Morin |
| Phosphatidylinositol 4,5-bisphosphate 3-kinase catalytic subunit gamma isoform | 1 | 0.9 | 2 | Morin |
| Placenta growth factor | 1 | 0.7 | 1 | Morin |
| Poly [ADP-ribose] polymerase 1 | 1 | 0.7 | 10 | Morin |
| Poly [ADP-ribose] polymerase tankyrase-1 | 1 | 0.7 | 7 | Morin |
| Poly [ADP-ribose] polymerase tankyrase-2 | 1 | 0.7 | 6 | Morin |
| Polyphenol oxidase 2 | 1 | 0.8 | 2 | Morin |
| Polyunsaturated fatty acid 5-lipoxygenase | 1 | 0.9 | 32 | Morin |
| Polyunsaturated fatty acid lipoxygenase ALOX12 | 1 | 0.86 | 3 | Morin |
| Polyunsaturated fatty acid lipoxygenase ALOX15 | 1 | 0.82 | 2 | Morin |
| Polyunsaturated fatty acid lipoxygenase ALOX15B | 1 | 0.82 | 1 | Morin |
| Potassium voltage-gated channel subfamily A member 3 | 1 | 0.7 | 1 | Morin |
| Proteasome subunit beta type-5 | 1 | 0.83 | 1 | Morin |
| Prothrombin | 1 | 0.81 | 2 | Morin |
| Pyruvate kinase PKM | 1 | 0.83 | 3 | Morin |
| Receptor-type tyrosine-protein kinase FLT3 | 1 | 0.81 | 6 | Morin |
| Receptor-type tyrosine-protein phosphatase S | 1 | 0.7 | 3 | Morin |
| Replicase polyprotein 1ab | 1 | 0.82 | 3 | Morin |
| Serine/threonine-protein kinase pim-1 | 1 | 0.88 | 8 | Morin |
| Short transient receptor potential channel 5 | 1 | 0.92 | 2 | Morin |
| Solute carrier organic anion transporter family member 2B1 | 1 | 0.85 | 1 | Morin |
| Telomerase reverse transcriptase | 1 | 0.8 | 1 | Morin |
| Thiosulfate sulfurtransferase | 1 | 0.87 | 1 | Morin |
| Transthyretin | 1 | 0.83 | 2 | Morin |
| Tyrosinase | 1 | 0.81 | 3 | Morin |
| Tyrosine-protein kinase Lck | 1 | 0.7 | 1 | Morin |
| Tyrosine-protein kinase receptor UFO | 1 | 0.9 | 1 | Morin |
| Tyrosine-protein kinase Yes | 1 | 0.9 | 1 | Morin |
| Tyrosine-protein phosphatase non-receptor type 1 | 1 | 0.7 | 1 | Morin |
| Vascular endothelial growth factor A | 1 | 0.7 | 1 | Morin |
| Vascular endothelial growth factor receptor 2 | 1 | 0.83 | 1 | Morin |
| Xanthine dehydrogenase/oxidase | 1 | 0.9 | 14 | Morin |
| 17-beta-hydroxysteroid dehydrogenase type 1 | 1 | 0.92 | 1 | Luteolin |
| 17-beta-hydroxysteroid dehydrogenase type 2 | 1 | 0.86 | 1 | Luteolin |
| 3-oxoacyl-acyl-carrier protein reductase | 1 | 1 | 1 | Luteolin |
| 5'-nucleotidase | 1 | 0.9 | 1 | Luteolin |
| 5-hydroxytryptamine receptor 2A | 1 | 0.7 | 1 | Luteolin |
| Acetylcholinesterase | 1 | 0.74 | 18 | Luteolin |
| Adenosine receptor A1 | 1 | 0.74 | 3 | Luteolin |
| Adenosine receptor A2a | 1 | 0.84 | 1 | Luteolin |
| Adenosine receptor A3 | 1 | 0.73 | 2 | Luteolin |
| Aldo-keto reductase family 1 member B1 | 1 | 0.81 | 33 | Luteolin |
| Aldo-keto reductase family 1 member B1 [K65Q] | 1 | 0.71 | 10 | Luteolin |
| Aldo-keto reductase family 1 member C3 | 1 | 0.74 | 1 | Luteolin |
| Alpha-synuclein | 1 | 0.9 | 1 | Luteolin |
| Amine oxidase [flavin-containing] A | 1 | 0.93 | 10 | Luteolin |
| Amine oxidase [flavin-containing] B | 1 | 0.87 | 11 | Luteolin |
| Amyloid-beta precursor protein | 1 | 0.7 | 3 | Luteolin |
| Androgen receptor | 1 | 0.74 | 10 | Luteolin |
| Aromatase | 1 | 0.82 | 12 | Luteolin |
| Aryl hydrocarbon receptor | 1 | 0.74 | 5 | Luteolin |
| ATP-dependent translocase ABCB1 | 1 | 0.71 | 21 | Luteolin |
| Aurora kinase B | 1 | 0.9 | 1 | Luteolin |
| BDNF/NT-3 growth factors receptor | 1 | 0.79 | 1 | Luteolin |
| Beta-secretase 1 | 1 | 0.9 | 3 | Luteolin |
| Broad substrate specificity ATP-binding cassette transporter ABCG2 | 1 | 0.74 | 17 | Luteolin |
| Bromodomain-containing protein 4 | 1 | 0.88 | 1 | Luteolin |
| cAMP-specific 3',5'-cyclic phosphodiesterase 4D | 1 | 0.71 | 1 | Luteolin |
| Carbonic anhydrase 12 | 1 | 0.85 | 5 | Luteolin |
| Carbonic anhydrase 2 | 1 | 0.85 | 2 | Luteolin |
| Carbonic anhydrase 4 | 1 | 1 | 4 | Luteolin |
| Carbonic anhydrase 7 | 1 | 0.85 | 5 | Luteolin |
| Carbonyl reductase [NADPH] 1 | 1 | 0.89 | 1 | Luteolin |
| Carboxylic ester hydrolase | 1 | 0.71 | 11 | Luteolin |
| Casein kinase II subunit alpha | 1 | 0.88 | 2 | Luteolin |
| Casein kinase II subunit alpha 3 | 1 | 1 | 6 | Luteolin |
| Cathepsin L2 | 1 | 0.73 | 1 | Luteolin |
| CDGSH iron-sulfur domain-containing protein 1 | 1 | 0.86 | 1 | Luteolin |
| cGMP-specific 3',5'-cyclic phosphodiesterase | 1 | 0.71 | 1 | Luteolin |
| Cholinesterase | 1 | 0.74 | 3 | Luteolin |
| Co-chaperonin GroES | 1 | 0.85 | 1 | Luteolin |
| Cyclin homolog/dependent kinase 6 | 1 | 0.88 | 1 | Luteolin |
| Cyclin-C | 1 | 0.7 | 8 | Luteolin |
| Cyclin-dependent kinase 1 | 1 | 0.9 | 1 | Luteolin |
| Cyclin-dependent kinase 1/G2/mitotic-specific cyclin-B | 1 | 0.88 | 1 | Luteolin |
| Cyclin-dependent kinase 2 | 1 | 0.7 | 1 | Luteolin |
| Cyclin-dependent kinase 5 activator 1 [99-307] | 1 | 0.88 | 1 | Luteolin |
| Cyclin-dependent kinase 6 | 1 | 0.88 | 1 | Luteolin |
| Cyclin-dependent kinase 9 | 1 | 0.76 | 1 | Luteolin |
| Cyclin-T1/Cyclin-dependent kinase 9 | 1 | 0.76 | 1 | Luteolin |
| Cytochrome P450 1A1 | 1 | 0.74 | 57 | Luteolin |
| Cytochrome P450 1A2 | 1 | 0.74 | 49 | Luteolin |
| Cytochrome P450 1B1 | 1 | 0.74 | 58 | Luteolin |
| Cytochrome P450 2D6 | 1 | 0.74 | 1 | Luteolin |
| Cytochrome P450 4F2 | 1 | 0.7 | 1 | Luteolin |
| Delta-type opioid receptor | 1 | 0.7 | 1 | Luteolin |
| Dipeptidyl peptidase 4 | 1 | 0.77 | 6 | Luteolin |
| Discoidin domain-containing receptor 2 | 1 | 0.7 | 1 | Luteolin |
| DNA topoisomerase 1 | 1 | 1 | 1 | Luteolin |
| DNA topoisomerase 2-alpha | 1 | 0.9 | 1 | Luteolin |
| DNA-(apurinic or apyrimidinic site) endonuclease | 1 | 0.9 | 1 | Luteolin |
| Dual specificity mitogen-activated protein kinase kinase 2 | 1 | 0.7 | 1 | Luteolin |
| Dual specificity tyrosine-phosphorylation-regulated kinase 1A | 1 | 0.7 | 1 | Luteolin |
| ELAV-like protein 3 | 1 | 0.9 | 1 | Luteolin |
| Enoyl-acyl-carrier protein reductase | 1 | 0.88 | 3 | Luteolin |
| Epidermal growth factor receptor | 1 | 0.84 | 2 | Luteolin |
| Estrogen receptor | 1 | 0.71 | 1 | Luteolin |
| Estrogen receptor beta | 1 | 0.82 | 3 | Luteolin |
| Gamma-aminobutyric acid receptor subunit alpha-1 | 1 | 0.7 | 3 | Luteolin |
| Gamma-aminobutyric acid receptor subunit alpha-2 | 1 | 0.7 | 1 | Luteolin |
| Gamma-aminobutyric acid receptor subunit alpha-3 | 1 | 0.7 | 1 | Luteolin |
| Gamma-aminobutyric acid receptor subunit alpha-5 | 1 | 0.7 | 1 | Luteolin |
| Genome polyprotein | 1 | 0.7 | 2 | Luteolin |
| Glycogen synthase kinase-3 beta | 1 | 1 | 2 | Luteolin |
| Hepatocyte growth factor receptor | 1 | 0.9 | 1 | Luteolin |
| Inositol hexakisphosphate kinase 2 | 1 | 0.9 | 4 | Luteolin |
| Insulin-like growth factor 1 receptor | 1 | 0.9 | 1 | Luteolin |
| Integrase | 1 | 0.88 | 6 | Luteolin |
| Intestinal-type alkaline phosphatase | 1 | 0.73 | 1 | Luteolin |
| Lactoylglutathione lyase | 1 | 0.9 | 1 | Luteolin |
| Luciferin 4-monooxygenase | 1 | 0.72 | 1 | Luteolin |
| M18 aspartyl aminopeptidase | 1 | 0.9 | 1 | Luteolin |
| Macrophage metalloelastase | 1 | 1 | 1 | Luteolin |
| MAP kinase-interacting serine/threonine-protein kinase 1 | 1 | 1 | 1 | Luteolin |
| MAP kinase-interacting serine/threonine-protein kinase 2 | 1 | 1 | 4 | Luteolin |
| Mast/stem cell growth factor receptor Kit | 1 | 0.84 | 2 | Luteolin |
| Matrix metalloproteinase-9 | 1 | 0.9 | 1 | Luteolin |
| Mu-type opioid receptor | 1 | 0.74 | 1 | Luteolin |
| Multidrug resistance-associated protein 1 | 1 | 0.71 | 1 | Luteolin |
| Muscleblind-like protein 1 | 1 | 0.9 | 1 | Luteolin |
| NADPH oxidase 4 | 1 | 0.9 | 3 | Luteolin |
| Nonstructural protein 3 | 1 | 0.9 | 1 | Luteolin |
| Nuclear receptor subfamily 0 group B member 1 | 1 | 0.84 | 1 | Luteolin |
| PC4 and SFRS1-interacting protein | 1 | 0.9 | 2 | Luteolin |
| Peroxisome proliferator-activated receptor alpha | 1 | 0.7 | 1 | Luteolin |
| Peroxisome proliferator-activated receptor gamma | 1 | 0.71 | 3 | Luteolin |
| Phosphatidylinositol 4,5-bisphosphate 3-kinase catalytic subunit gamma isoform | 1 | 0.9 | 2 | Luteolin |
| Placenta growth factor | 1 | 0.7 | 1 | Luteolin |
| Poly [ADP-ribose] polymerase 1 | 1 | 0.7 | 8 | Luteolin |
| Poly [ADP-ribose] polymerase tankyrase-1 | 1 | 0.77 | 8 | Luteolin |
| Poly [ADP-ribose] polymerase tankyrase-2 | 1 | 0.77 | 6 | Luteolin |
| Polyphenol oxidase 2 | 1 | 0.88 | 2 | Luteolin |
| Polyunsaturated fatty acid 5-lipoxygenase | 1 | 0.9 | 41 | Luteolin |
| Polyunsaturated fatty acid lipoxygenase ALOX12 | 1 | 0.88 | 3 | Luteolin |
| Polyunsaturated fatty acid lipoxygenase ALOX15 | 1 | 0.86 | 2 | Luteolin |
| Polyunsaturated fatty acid lipoxygenase ALOX15B | 1 | 0.86 | 1 | Luteolin |
| Prostaglandin G/H synthase 1 | 1 | 0.7 | 2 | Luteolin |
| Proteasome subunit beta type-5 | 1 | 0.92 | 1 | Luteolin |
| Protein E6 | 1 | 0.9 | 1 | Luteolin |
| Prothrombin | 1 | 0.82 | 2 | Luteolin |
| Pyruvate kinase PKM | 1 | 0.92 | 3 | Luteolin |
| Receptor-type tyrosine-protein kinase FLT3 | 1 | 0.9 | 6 | Luteolin |
| Receptor-type tyrosine-protein phosphatase S | 1 | 0.72 | 3 | Luteolin |
| Replicase polyprotein 1ab | 1 | 0.86 | 3 | Luteolin |
| Serine/threonine-protein kinase pim-1 | 1 | 0.88 | 9 | Luteolin |
| Short transient receptor potential channel 5 | 1 | 0.84 | 2 | Luteolin |
| Sialidase | 1 | 0.85 | 1 | Luteolin |
| Sigma non-opioid intracellular receptor 1 | 1 | 0.72 | 1 | Luteolin |
| Sodium-dependent dopamine transporter | 1 | 0.76 | 3 | Luteolin |
| Solute carrier organic anion transporter family member 2B1 | 1 | 0.89 | 1 | Luteolin |
| Sucrase-isomaltase, intestinal | 1 | 0.8 | 1 | Luteolin |
| Sulfotransferase 1A1 | 1 | 0.9 | 2 | Luteolin |
| Synapsin-1 | 1 | 0.88 | 1 | Luteolin |
| Telomerase reverse transcriptase | 1 | 0.76 | 6 | Luteolin |
| Thiosulfate sulfurtransferase | 1 | 0.85 | 1 | Luteolin |
| Transthyretin | 1 | 0.92 | 2 | Luteolin |
| Tyrosinase | 1 | 1 | 3 | Luteolin |
| Tyrosine-protein kinase Lck | 1 | 0.71 | 1 | Luteolin |
| Tyrosine-protein kinase receptor UFO | 1 | 0.9 | 1 | Luteolin |
| Tyrosine-protein kinase Yes | 1 | 0.9 | 1 | Luteolin |
| Tyrosine-protein phosphatase non-receptor type 1 | 1 | 0.71 | 1 | Luteolin |
| Vascular endothelial growth factor A | 1 | 0.7 | 1 | Luteolin |
| Vascular endothelial growth factor receptor 2 | 1 | 0.84 | 1 | Luteolin |
| Xanthine dehydrogenase/oxidase | 1 | 0.9 | 14 | Luteolin |
| 17-beta-hydroxysteroid dehydrogenase type 1 | 1 | 0.83 | 1 | Rhamnetin |
| 17-beta-hydroxysteroid dehydrogenase type 2 | 1 | 0.94 | 1 | Rhamnetin |
| 3-oxoacyl-acyl-carrier protein reductase | 1 | 0.9 | 1 | Rhamnetin |
| 5'-nucleotidase | 1 | 1 | 1 | Rhamnetin |
| Acetylcholinesterase | 1 | 0.77 | 11 | Rhamnetin |
| Adenosine receptor A1 | 1 | 0.7 | 4 | Rhamnetin |
| Adenosine receptor A2a | 1 | 0.92 | 1 | Rhamnetin |
| Adenosine receptor A3 | 1 | 0.7 | 6 | Rhamnetin |
| Aldo-keto reductase family 1 member B1 | 1 | 0.8 | 33 | Rhamnetin |
| Aldo-keto reductase family 1 member B1 [K65Q] | 1 | 0.7 | 9 | Rhamnetin |
| Aldo-keto reductase family 1 member C3 | 1 | 0.71 | 1 | Rhamnetin |
| Alpha-synuclein | 1 | 1 | 1 | Rhamnetin |
| Amine oxidase [flavin-containing] A | 1 | 0.84 | 8 | Rhamnetin |
| Amine oxidase [flavin-containing] B | 1 | 0.79 | 6 | Rhamnetin |
| Androgen receptor | 1 | 0.71 | 8 | Rhamnetin |
| Aromatase | 1 | 0.74 | 12 | Rhamnetin |
| Aryl hydrocarbon receptor | 1 | 0.7 | 4 | Rhamnetin |
| ATP-dependent translocase ABCB1 | 1 | 0.7 | 18 | Rhamnetin |
| Aurora kinase B | 1 | 1 | 1 | Rhamnetin |
| BDNF/NT-3 growth factors receptor | 1 | 0.77 | 1 | Rhamnetin |
| Beta-secretase 1 | 1 | 1 | 5 | Rhamnetin |
| Broad substrate specificity ATP-binding cassette transporter ABCG2 | 1 | 0.7 | 8 | Rhamnetin |
| Bromodomain-containing protein 4 | 1 | 0.86 | 1 | Rhamnetin |
| cAMP-specific 3',5'-cyclic phosphodiesterase 4D | 1 | 0.7 | 1 | Rhamnetin |
| Carbonic anhydrase 12 | 1 | 0.94 | 5 | Rhamnetin |
| Carbonic anhydrase 2 | 1 | 0.94 | 2 | Rhamnetin |
| Carbonic anhydrase 4 | 1 | 0.9 | 4 | Rhamnetin |
| Carbonic anhydrase 7 | 1 | 0.94 | 5 | Rhamnetin |
| Carbonyl reductase [NADPH] 1 | 1 | 0.8 | 1 | Rhamnetin |
| Carboxylic ester hydrolase | 1 | 0.7 | 1 | Rhamnetin |
| Casein kinase II subunit alpha | 1 | 0.97 | 2 | Rhamnetin |
| Casein kinase II subunit alpha 3 | 1 | 0.9 | 6 | Rhamnetin |
| CDGSH iron-sulfur domain-containing protein 1 | 1 | 0.94 | 1 | Rhamnetin |
| cGMP-specific 3',5'-cyclic phosphodiesterase | 1 | 0.72 | 2 | Rhamnetin |
| Co-chaperonin GroES | 1 | 0.94 | 1 | Rhamnetin |
| Cyclin homolog/dependent kinase 6 | 1 | 0.97 | 1 | Rhamnetin |
| Cyclin-C | 1 | 0.7 | 5 | Rhamnetin |
| Cyclin-dependent kinase 1 | 1 | 1 | 1 | Rhamnetin |
| Cyclin-dependent kinase 1/G2/mitotic-specific cyclin-B | 1 | 0.97 | 1 | Rhamnetin |
| Cyclin-dependent kinase 2 | 1 | 0.7 | 2 | Rhamnetin |
| Cyclin-dependent kinase 5 activator 1 [99-307] | 1 | 0.97 | 1 | Rhamnetin |
| Cyclin-dependent kinase 6 | 1 | 0.97 | 1 | Rhamnetin |
| Cyclin-dependent kinase 9 | 1 | 0.75 | 1 | Rhamnetin |
| Cyclin-T1/Cyclin-dependent kinase 9 | 1 | 0.75 | 1 | Rhamnetin |
| Cytochrome P450 1A1 | 1 | 0.7 | 46 | Rhamnetin |
| Cytochrome P450 1A2 | 1 | 0.7 | 37 | Rhamnetin |
| Cytochrome P450 1B1 | 1 | 0.7 | 49 | Rhamnetin |
| Cytochrome P450 2D6 | 1 | 0.81 | 1 | Rhamnetin |
| Delta-type opioid receptor | 1 | 0.7 | 1 | Rhamnetin |
| Dipeptidyl peptidase 4 | 1 | 0.7 | 6 | Rhamnetin |
| DNA topoisomerase 1 | 1 | 0.9 | 1 | Rhamnetin |
| DNA topoisomerase 2-alpha | 1 | 1 | 1 | Rhamnetin |
| DNA-(apurinic or apyrimidinic site) endonuclease | 1 | 1 | 1 | Rhamnetin |
| Dual specificity tyrosine-phosphorylation-regulated kinase 1A | 1 | 0.7 | 1 | Rhamnetin |
| ELAV-like protein 3 | 1 | 1 | 1 | Rhamnetin |
| Enoyl-acyl-carrier protein reductase | 1 | 0.97 | 3 | Rhamnetin |
| Epidermal growth factor receptor | 1 | 0.92 | 2 | Rhamnetin |
| Estrogen receptor | 1 | 0.7 | 1 | Rhamnetin |
| Estrogen receptor beta | 1 | 0.77 | 3 | Rhamnetin |
| Gamma-aminobutyric acid receptor subunit alpha-1 | 1 | 0.7 | 2 | Rhamnetin |
| Gamma-aminobutyric acid receptor subunit alpha-2 | 1 | 0.7 | 1 | Rhamnetin |
| Gamma-aminobutyric acid receptor subunit alpha-3 | 1 | 0.7 | 1 | Rhamnetin |
| Gamma-aminobutyric acid receptor subunit alpha-5 | 1 | 0.7 | 1 | Rhamnetin |
| Genome polyprotein | 1 | 0.7 | 1 | Rhamnetin |
| Glycogen synthase kinase-3 beta | 1 | 0.9 | 2 | Rhamnetin |
| Hepatocyte growth factor receptor | 1 | 1 | 1 | Rhamnetin |
| Inositol hexakisphosphate kinase 2 | 1 | 1 | 4 | Rhamnetin |
| Insulin-like growth factor 1 receptor | 1 | 1 | 1 | Rhamnetin |
| Integrase | 1 | 0.98 | 8 | Rhamnetin |
| Lactoylglutathione lyase | 1 | 1 | 1 | Rhamnetin |
| Low molecular weight phosphotyrosine protein phosphatase | 1 | 0.7 | 1 | Rhamnetin |
| Luciferin 4-monooxygenase | 1 | 0.7 | 1 | Rhamnetin |
| M18 aspartyl aminopeptidase | 1 | 1 | 1 | Rhamnetin |
| Macrophage metalloelastase | 1 | 0.9 | 1 | Rhamnetin |
| MAP kinase-interacting serine/threonine-protein kinase 1 | 1 | 0.9 | 1 | Rhamnetin |
| MAP kinase-interacting serine/threonine-protein kinase 2 | 1 | 0.9 | 4 | Rhamnetin |
| Mast/stem cell growth factor receptor Kit | 1 | 0.79 | 2 | Rhamnetin |
| Matrix metalloproteinase-9 | 1 | 1 | 1 | Rhamnetin |
| Mu-type opioid receptor | 1 | 0.75 | 1 | Rhamnetin |
| Muscleblind-like protein 1 | 1 | 1 | 1 | Rhamnetin |
| NADPH oxidase 4 | 1 | 1 | 3 | Rhamnetin |
| Nonstructural protein 3 | 1 | 1 | 1 | Rhamnetin |
| Nuclear receptor subfamily 0 group B member 1 | 1 | 0.8 | 1 | Rhamnetin |
| PC4 and SFRS1-interacting protein | 1 | 1 | 2 | Rhamnetin |
| Peroxisome proliferator-activated receptor alpha | 1 | 0.7 | 1 | Rhamnetin |
| Peroxisome proliferator-activated receptor gamma | 1 | 0.7 | 1 | Rhamnetin |
| Phosphatidylinositol 4,5-bisphosphate 3-kinase catalytic subunit gamma isoform | 1 | 1 | 2 | Rhamnetin |
| Placenta growth factor | 1 | 0.7 | 1 | Rhamnetin |
| Poly [ADP-ribose] polymerase 1 | 1 | 0.7 | 11 | Rhamnetin |
| Poly [ADP-ribose] polymerase tankyrase-1 | 1 | 0.7 | 7 | Rhamnetin |
| Poly [ADP-ribose] polymerase tankyrase-2 | 1 | 0.7 | 6 | Rhamnetin |
| Polyphenol oxidase 2 | 1 | 0.86 | 2 | Rhamnetin |
| Polyunsaturated fatty acid 5-lipoxygenase | 1 | 1 | 50 | Rhamnetin |
| Polyunsaturated fatty acid lipoxygenase ALOX12 | 1 | 0.97 | 3 | Rhamnetin |
| Polyunsaturated fatty acid lipoxygenase ALOX15 | 1 | 0.81 | 2 | Rhamnetin |
| Polyunsaturated fatty acid lipoxygenase ALOX15B | 1 | 0.81 | 1 | Rhamnetin |
| Prostaglandin G/H synthase 1 | 1 | 0.7 | 1 | Rhamnetin |
| Proteasome subunit beta type-5 | 1 | 0.83 | 1 | Rhamnetin |
| Protein E6 | 1 | 1 | 1 | Rhamnetin |
| Prothrombin | 1 | 0.9 | 2 | Rhamnetin |
| Pyruvate kinase PKM | 1 | 0.83 | 3 | Rhamnetin |
| Receptor-type tyrosine-protein kinase FLT3 | 1 | 0.81 | 6 | Rhamnetin |
| Receptor-type tyrosine-protein phosphatase S | 1 | 0.7 | 3 | Rhamnetin |
| Replicase polyprotein 1ab | 1 | 0.81 | 3 | Rhamnetin |
| Serine/threonine-protein kinase pim-1 | 1 | 0.98 | 8 | Rhamnetin |
| Short transient receptor potential channel 5 | 1 | 0.92 | 2 | Rhamnetin |
| Sialidase | 1 | 0.94 | 1 | Rhamnetin |
| Sodium-dependent dopamine transporter | 1 | 0.71 | 3 | Rhamnetin |
| Solute carrier organic anion transporter family member 2B1 | 1 | 0.84 | 1 | Rhamnetin |
| Sucrase-isomaltase, intestinal | 1 | 0.73 | 1 | Rhamnetin |
| Sulfotransferase 1A1 | 1 | 1 | 2 | Rhamnetin |
| Synapsin-1 | 1 | 0.98 | 1 | Rhamnetin |
| Telomerase reverse transcriptase | 1 | 0.75 | 6 | Rhamnetin |
| Thiosulfate sulfurtransferase | 1 | 0.94 | 1 | Rhamnetin |
| Transthyretin | 1 | 0.83 | 2 | Rhamnetin |
| Tyrosinase | 1 | 0.9 | 3 | Rhamnetin |
| Tyrosine-protein kinase Lck | 1 | 0.7 | 1 | Rhamnetin |
| Tyrosine-protein kinase receptor UFO | 1 | 1 | 1 | Rhamnetin |
| Tyrosine-protein kinase Yes | 1 | 1 | 1 | Rhamnetin |
| Tyrosine-protein phosphatase non-receptor type 1 | 1 | 0.7 | 1 | Rhamnetin |
| Vascular endothelial growth factor A | 1 | 0.7 | 1 | Rhamnetin |
| Vascular endothelial growth factor receptor 2 | 1 | 0.92 | 1 | Rhamnetin |
| Xanthine dehydrogenase/oxidase | 1 | 1 | 14 | Rhamnetin |
| 17-beta-hydroxysteroid dehydrogenase type 3 | 1 | 0.78 | 3 | Umbelliferone |
| Aldo-keto reductase family 1 member B1 | 1 | 0.7 | 1 | Umbelliferone |
| Amine oxidase [flavin-containing] A | 1 | 0.7 | 2 | Umbelliferone |
| Amine oxidase [flavin-containing] B | 1 | 0.72 | 2 | Umbelliferone |
| Amyloid-beta precursor protein | 1 | 0.7 | 1 | Umbelliferone |
| Angiopoietin-1 receptor | 1 | 0.7 | 1 | Umbelliferone |
| Carbonic anhydrase 12 | 1 | 0.7 | 12 | Umbelliferone |
| Carbonic anhydrase 14 | 1 | 0.77 | 1 | Umbelliferone |
| Carbonic anhydrase 4 | 1 | 0.7 | 2 | Umbelliferone |
| Carbonic anhydrase 6 | 1 | 0.7 | 1 | Umbelliferone |
| Carbonic anhydrase 9 | 1 | 1 | 19 | Umbelliferone |
| Casein kinase II subunit alpha | 1 | 0.7 | 2 | Umbelliferone |
| Casein kinase II subunit alpha 3 | 1 | 0.71 | 1 | Umbelliferone |
| Cytochrome P450 2A6 | 1 | 0.75 | 6 | Umbelliferone |
| D-amino-acid oxidase | 1 | 0.76 | 1 | Umbelliferone |
| DNA polymerase eta | 1 | 0.7 | 1 | Umbelliferone |
| DNA polymerase iota | 1 | 0.7 | 1 | Umbelliferone |
| Dual specificity protein phosphatase 3 | 1 | 0.7 | 1 | Umbelliferone |
| Dual specificity tyrosine-phosphorylation-regulated kinase 1A | 1 | 0.7 | 1 | Umbelliferone |
| ELAV-like protein 3 | 1 | 0.7 | 1 | Umbelliferone |
| Epidermal growth factor receptor | 1 | 0.7 | 1 | Umbelliferone |
| Estrogen receptor | 1 | 0.71 | 2 | Umbelliferone |
| Estrogen receptor beta | 1 | 0.71 | 2 | Umbelliferone |
| G-protein coupled receptor 35 | 1 | 0.7 | 1 | Umbelliferone |
| Heat shock 70 kDa protein 1A | 1 | 0.7 | 1 | Umbelliferone |
| Hepatocyte growth factor receptor | 1 | 0.7 | 1 | Umbelliferone |
| Insulin receptor | 1 | 0.7 | 1 | Umbelliferone |
| Insulin-like growth factor 1 receptor | 1 | 0.7 | 1 | Umbelliferone |
| Melatonin receptor type 1A | 1 | 0.7 | 1 | Umbelliferone |
| Mothers against decapentaplegic homolog 3 | 1 | 0.7 | 1 | Umbelliferone |
| NUAK family SNF1-like kinase 1 | 1 | 0.7 | 1 | Umbelliferone |
| Polypeptide N-acetylgalactosaminyltransferase 10 | 1 | 0.74 | 1 | Umbelliferone |
| Polypeptide N-acetylgalactosaminyltransferase 14 | 1 | 0.74 | 1 | Umbelliferone |
| Polypeptide N-acetylgalactosaminyltransferase 2 | 1 | 0.7 | 2 | Umbelliferone |
| Potassium voltage-gated channel subfamily A member 3 | 1 | 0.7 | 1 | Umbelliferone |
| Proto-oncogene tyrosine-protein kinase Src | 1 | 0.7 | 1 | Umbelliferone |
| Solute carrier family 22 member 6 | 1 | 0.7 | 1 | Umbelliferone |
| Sphingosine kinase 1 | 1 | 0.7 | 1 | Umbelliferone |
| Vascular endothelial growth factor receptor 2 | 1 | 0.7 | 1 | Umbelliferone |
| 6-phosphogluconate dehydrogenase, decarboxylating | 1 | 0.78 | 2 | EPICATCHIN |
| 72 kDa type IV collagenase | 1 | 0.82 | 1 | EPICATCHIN |
| A disintegrin and metalloproteinase with thrombospondin motifs 4 | 1 | 0.78 | 2 | EPICATCHIN |
| A disintegrin and metalloproteinase with thrombospondin motifs 5 | 1 | 0.78 | 2 | EPICATCHIN |
| Alkaline phosphatase, tissue-nonspecific isozyme | 1 | 1 | 1 | EPICATCHIN |
| Alpha-(1,3)-fucosyltransferase 7 | 1 | 0.78 | 1 | EPICATCHIN |
| Alpha-synuclein | 1 | 1 | 1 | EPICATCHIN |
| Amyloid-beta precursor protein | 1 | 0.78 | 1 | EPICATCHIN |
| Apoptosis regulator Bcl-2 | 1 | 0.8 | 6 | EPICATCHIN |
| Aromatase | 1 | 0.7 | 3 | EPICATCHIN |
| ATP-dependent translocase ABCB1 | 1 | 0.72 | 32 | EPICATCHIN |
| Beta-secretase 1 | 1 | 0.78 | 3 | EPICATCHIN |
| Broad substrate specificity ATP-binding cassette transporter ABCG2 | 1 | 0.7 | 1 | EPICATCHIN |
| Carbonic anhydrase 1 | 1 | 0.7 | 1 | EPICATCHIN |
| Carbonic anhydrase 12 | 1 | 0.73 | 4 | EPICATCHIN |
| Carbonic anhydrase 2 | 1 | 0.7 | 1 | EPICATCHIN |
| Carbonic anhydrase 4 | 1 | 0.72 | 3 | EPICATCHIN |
| Carbonic anhydrase 7 | 1 | 1 | 5 | EPICATCHIN |
| Casein kinase I isoform epsilon | 1 | 0.78 | 1 | EPICATCHIN |
| Collagenase 3 | 1 | 0.71 | 1 | EPICATCHIN |
| Cytochrome P450 1B1 | 1 | 0.71 | 2 | EPICATCHIN |
| Dipeptidyl peptidase 4 | 1 | 0.72 | 2 | EPICATCHIN |
| DNA (cytosine-5)-methyltransferase 1 | 1 | 0.78 | 1 | EPICATCHIN |
| DNA repair protein RAD52 homolog | 1 | 0.78 | 1 | EPICATCHIN |
| Dual specificity protein kinase CLK1 | 1 | 0.78 | 1 | EPICATCHIN |
| Dual specificity protein kinase CLK4 | 1 | 0.78 | 1 | EPICATCHIN |
| Dual specificity tyrosine-phosphorylation-regulated kinase 1A | 1 | 0.78 | 22 | EPICATCHIN |
| Dual specificity tyrosine-phosphorylation-regulated kinase 1B | 1 | 0.78 | 1 | EPICATCHIN |
| ELAV-like protein 3 | 1 | 0.78 | 2 | EPICATCHIN |
| Estrogen receptor | 1 | 0.7 | 6 | EPICATCHIN |
| Estrogen receptor beta | 1 | 0.7 | 7 | EPICATCHIN |
| Fibroblast growth factor receptor 1 | 1 | 0.76 | 1 | EPICATCHIN |
| Heat shock protein HSP 90-beta | 1 | 0.73 | 1 | EPICATCHIN |
| Hepatocyte growth factor receptor | 1 | 0.76 | 1 | EPICATCHIN |
| MAP kinase-activated protein kinase 5 | 1 | 0.78 | 1 | EPICATCHIN |
| Mast/stem cell growth factor receptor Kit | 1 | 0.76 | 1 | EPICATCHIN |
| Multidrug resistance-associated protein 1 | 1 | 0.7 | 1 | EPICATCHIN |
| NAD kinase | 1 | 0.78 | 1 | EPICATCHIN |
| Phosphatidylinositol 4,5-bisphosphate 3-kinase catalytic subunit alpha isoform | 1 | 0.78 | 1 | EPICATCHIN |
| Phosphoglycerate mutase 1 | 1 | 0.75 | 2 | EPICATCHIN |
| Placenta growth factor | 1 | 0.76 | 3 | EPICATCHIN |
| Plasminogen activator inhibitor 1 | 1 | 0.74 | 2 | EPICATCHIN |
| Proteasome subunit beta type-5 | 1 | 0.78 | 4 | EPICATCHIN |
| Prothrombin | 1 | 0.7 | 1 | EPICATCHIN |
| Proto-oncogene tyrosine-protein kinase Src | 1 | 0.76 | 1 | EPICATCHIN |
| Pyruvate kinase PKM | 1 | 0.78 | 2 | EPICATCHIN |
| RAC-alpha serine/threonine-protein kinase | 1 | 0.7 | 1 | EPICATCHIN |
| Serine/threonine-protein kinase mTOR | 1 | 0.78 | 1 | EPICATCHIN |
| Sex hormone-binding globulin | 1 | 0.7 | 1 | EPICATCHIN |
| Signal transducer and activator of transcription 1-alpha/beta | 1 | 0.78 | 1 | EPICATCHIN |
| Solute carrier organic anion transporter family member 2B1 | 1 | 0.71 | 1 | EPICATCHIN |
| Telomerase reverse transcriptase | 1 | 0.78 | 1 | EPICATCHIN |
| Transthyretin | 1 | 0.78 | 1 | EPICATCHIN |
| Tyrosine-protein kinase receptor UFO | 1 | 0.7 | 1 | EPICATCHIN |
| Vascular endothelial growth factor A | 1 | 0.76 | 3 | EPICATCHIN |
| 11-beta-hydroxysteroid dehydrogenase 1 | 1 | 0.7 | 1 | Coumaric acid |
| 72 kDa type IV collagenase | 1 | 0.84 | 1 | Coumaric acid |
| Aldo-keto reductase family 1 member B1 | 1 | 1 | 1 | Coumaric acid |
| Aldo-keto reductase family 1 member B10 | 1 | 0.7 | 2 | Coumaric acid |
| Alpha-synuclein | 1 | 0.73 | 1 | Coumaric acid |
| Carbonic anhydrase 1 | 1 | 0.89 | 3 | Coumaric acid |
| Carbonic anhydrase 12 | 1 | 0.89 | 3 | Coumaric acid |
| Carbonic anhydrase 14 | 1 | 0.89 | 4 | Coumaric acid |
| Carbonic anhydrase 2 | 1 | 1 | 5 | Coumaric acid |
| Carbonic anhydrase 5A, mitochondrial | 1 | 0.7 | 1 | Coumaric acid |
| Carbonic anhydrase 5B, mitochondrial | 1 | 0.7 | 1 | Coumaric acid |
| Carbonic anhydrase 6 | 1 | 0.7 | 1 | Coumaric acid |
| Carbonic anhydrase 7 | 1 | 0.89 | 4 | Coumaric acid |
| Histone deacetylase | 1 | 0.78 | 1 | Coumaric acid |
| Interstitial collagenase | 1 | 0.84 | 1 | Coumaric acid |
| Matrix metalloproteinase-9 | 1 | 0.84 | 1 | Coumaric acid |
| Poly [ADP-ribose] polymerase tankyrase-1/tankyrase-2 | 1 | 0.78 | 1 | Coumaric acid |
| Tissue factor | 1 | 0.7 | 1 | Coumaric acid |
| Type-1 angiotensin II receptor | 1 | 0.84 | 1 | Coumaric acid |
| 11-beta-hydroxysteroid dehydrogenase 1 | 1 | 0.7 | 1 | Coumaric acid |
| 72 kDa type IV collagenase | 1 | 0.84 | 1 | Coumaric acid |
| Aldo-keto reductase family 1 member B1 | 1 | 1 | 1 | Coumaric acid |
| Aldo-keto reductase family 1 member B10 | 1 | 0.7 | 2 | Coumaric acid |
| Alpha-synuclein | 1 | 0.73 | 1 | Coumaric acid |
| Carbonic anhydrase 1 | 1 | 0.89 | 3 | Coumaric acid |
| Carbonic anhydrase 12 | 1 | 0.89 | 3 | Coumaric acid |
| Carbonic anhydrase 14 | 1 | 0.89 | 4 | Coumaric acid |
| Carbonic anhydrase 2 | 1 | 1 | 5 | Coumaric acid |
| Carbonic anhydrase 5A, mitochondrial | 1 | 0.7 | 1 | Coumaric acid |
| Carbonic anhydrase 5B, mitochondrial | 1 | 0.7 | 1 | Coumaric acid |
| Carbonic anhydrase 6 | 1 | 0.7 | 1 | Coumaric acid |
| Carbonic anhydrase 7 | 1 | 0.89 | 4 | Coumaric acid |
| Histone deacetylase | 1 | 0.78 | 1 | Coumaric acid |
| Interstitial collagenase | 1 | 0.84 | 1 | Coumaric acid |
| Matrix metalloproteinase-9 | 1 | 0.84 | 1 | Coumaric acid |
| Poly [ADP-ribose] polymerase tankyrase-1/tankyrase-2 | 1 | 0.78 | 1 | Coumaric acid |
| Tissue factor | 1 | 0.7 | 1 | Coumaric acid |
| Type-1 angiotensin II receptor | 1 | 0.84 | 1 | Coumaric acid |
| Beta-secretase 1 | 1 | 0.7 | 1 | kino tannic acid |
| DNA repair protein RAD51 homolog 1 | 1 | 0.7 | 1 | kino tannic acid |
| Carboxypeptidase B2 | 1 | 0.78 | 1 | Histidine |
| 17-beta-hydroxysteroid dehydrogenase type 2 | 1 | 0.8 | 1 | Cyanidin |
| Acetylcholinesterase | 1 | 0.77 | 3 | Cyanidin |
| Adenosine receptor A2a | 1 | 0.77 | 1 | Cyanidin |
| Adenosine receptor A3 | 1 | 0.77 | 1 | Cyanidin |
| Aldo-keto reductase family 1 member B1 | 1 | 0.75 | 11 | Cyanidin |
| Alpha-synuclein | 1 | 0.85 | 1 | Cyanidin |
| Amine oxidase [flavin-containing] A | 1 | 0.78 | 4 | Cyanidin |
| Amyloid-beta precursor protein | 1 | 0.7 | 1 | Cyanidin |
| Aromatase | 1 | 0.85 | 1 | Cyanidin |
| Aryl hydrocarbon receptor | 1 | 0.8 | 1 | Cyanidin |
| Aurora kinase B | 1 | 0.85 | 1 | Cyanidin |
| Beta-secretase 1 | 1 | 0.85 | 2 | Cyanidin |
| Broad substrate specificity ATP-binding cassette transporter ABCG2 | 1 | 0.85 | 4 | Cyanidin |
| Bromodomain-containing protein 4 | 1 | 0.74 | 1 | Cyanidin |
| Carbonic anhydrase 12 | 1 | 0.8 | 4 | Cyanidin |
| Carbonic anhydrase 2 | 1 | 0.8 | 2 | Cyanidin |
| Carbonic anhydrase 4 | 1 | 0.76 | 3 | Cyanidin |
| Carbonic anhydrase 7 | 1 | 0.8 | 4 | Cyanidin |
| Casein kinase II subunit alpha | 1 | 0.83 | 1 | Cyanidin |
| Casein kinase II subunit alpha 3 | 1 | 0.76 | 5 | Cyanidin |
| CDGSH iron-sulfur domain-containing protein 1 | 1 | 0.8 | 1 | Cyanidin |
| Cyclin homolog/dependent kinase 6 | 1 | 0.83 | 1 | Cyanidin |
| Cyclin-dependent kinase 1 | 1 | 0.85 | 1 | Cyanidin |
| Cyclin-dependent kinase 2 | 1 | 0.7 | 1 | Cyanidin |
| Cyclin-dependent kinase 5 activator 1 [99-307] | 1 | 0.83 | 1 | Cyanidin |
| Cyclin-dependent kinase 6 | 1 | 0.83 | 1 | Cyanidin |
| Cytochrome P450 1A1 | 1 | 0.78 | 9 | Cyanidin |
| Cytochrome P450 1A2 | 1 | 0.77 | 3 | Cyanidin |
| Cytochrome P450 1B1 | 1 | 0.85 | 11 | Cyanidin |
| Dipeptidyl peptidase 4 | 1 | 0.76 | 2 | Cyanidin |
| DNA topoisomerase 2-alpha | 1 | 0.85 | 1 | Cyanidin |
| DNA-(apurinic or apyrimidinic site) endonuclease | 1 | 0.85 | 1 | Cyanidin |
| ELAV-like protein 3 | 1 | 0.85 | 1 | Cyanidin |
| Epidermal growth factor receptor | 1 | 0.85 | 2 | Cyanidin |
| Estrogen receptor | 1 | 0.7 | 3 | Cyanidin |
| Estrogen receptor beta | 1 | 0.7 | 2 | Cyanidin |
| Glycogen synthase kinase-3 beta | 1 | 0.76 | 2 | Cyanidin |
| Hepatocyte growth factor receptor | 1 | 0.85 | 1 | Cyanidin |
| Inositol hexakisphosphate kinase 2 | 1 | 0.85 | 4 | Cyanidin |
| Insulin-like growth factor 1 receptor | 1 | 0.85 | 1 | Cyanidin |
| Integrase | 1 | 0.83 | 4 | Cyanidin |
| Lactoylglutathione lyase | 1 | 0.85 | 1 | Cyanidin |
| Macrophage metalloelastase | 1 | 0.76 | 1 | Cyanidin |
| MAP kinase-interacting serine/threonine-protein kinase 1 | 1 | 0.76 | 1 | Cyanidin |
| MAP kinase-interacting serine/threonine-protein kinase 2 | 1 | 0.76 | 1 | Cyanidin |
| Matrix metalloproteinase-9 | 1 | 0.85 | 1 | Cyanidin |
| Muscleblind-like protein 1 | 1 | 0.85 | 1 | Cyanidin |
| NADPH oxidase 4 | 1 | 0.85 | 3 | Cyanidin |
| PC4 and SFRS1-interacting protein | 1 | 0.85 | 2 | Cyanidin |
| Phosphatidylinositol 4,5-bisphosphate 3-kinase catalytic subunit gamma isoform | 1 | 0.85 | 2 | Cyanidin |
| Poly [ADP-ribose] polymerase tankyrase-1 | 1 | 0.71 | 3 | Cyanidin |
| Poly [ADP-ribose] polymerase tankyrase-2 | 1 | 0.71 | 3 | Cyanidin |
| Polyunsaturated fatty acid 5-lipoxygenase | 1 | 0.85 | 1 | Cyanidin |
| Polyunsaturated fatty acid lipoxygenase ALOX12 | 1 | 0.83 | 2 | Cyanidin |
| Polyunsaturated fatty acid lipoxygenase ALOX15 | 1 | 0.85 | 1 | Cyanidin |
| Prostaglandin G/H synthase 1 | 1 | 0.73 | 1 | Cyanidin |
| Prothrombin | 1 | 0.84 | 2 | Cyanidin |
| Pyruvate kinase PKM | 1 | 0.83 | 2 | Cyanidin |
| Receptor-type tyrosine-protein kinase FLT3 | 1 | 0.76 | 2 | Cyanidin |
| Receptor-type tyrosine-protein phosphatase S | 1 | 0.71 | 1 | Cyanidin |
| Serine/threonine-protein kinase pim-1 | 1 | 0.83 | 7 | Cyanidin |
| Sex hormone-binding globulin | 1 | 0.7 | 1 | Cyanidin |
| Short transient receptor potential channel 5 | 1 | 0.77 | 1 | Cyanidin |
| Solute carrier organic anion transporter family member 2B1 | 1 | 0.71 | 1 | Cyanidin |
| Telomerase reverse transcriptase | 1 | 0.74 | 1 | Cyanidin |
| Thiosulfate sulfurtransferase | 1 | 0.8 | 1 | Cyanidin |
| Transthyretin | 1 | 0.76 | 1 | Cyanidin |
| Tyrosinase | 1 | 0.76 | 2 | Cyanidin |
| Tyrosine-protein kinase receptor UFO | 1 | 0.85 | 1 | Cyanidin |
| Tyrosine-protein kinase Yes | 1 | 0.85 | 1 | Cyanidin |
| Vascular endothelial growth factor receptor 2 | 1 | 0.85 | 1 | Cyanidin |
| Xanthine dehydrogenase/oxidase | 1 | 0.85 | 3 | Cyanidin |
| 11-beta-hydroxysteroid dehydrogenase 1 | 1 | 0.73 | 3 | SHELLOLOIC ACID |
| Tumor necrosis factor | 1 | 0.7 | 5 | SHELLOLOIC ACID |
| Acetylcholinesterase | 1 | 0.79 | 10 | Dimethyl Quercetin |
| Aldehyde dehydrogenase, mitochondrial | 1 | 0.95 | 28 | Dimethyl Quercetin |
| Aldehyde oxidase | 1 | 0.91 | 1 | Dimethyl Quercetin |
| Amine oxidase [flavin-containing] A | 1 | 0.7 | 5 | Dimethyl Quercetin |
| Amine oxidase [flavin-containing] B | 1 | 0.91 | 16 | Dimethyl Quercetin |
| Aromatase | 1 | 0.92 | 1 | Dimethyl Quercetin |
| Aryl hydrocarbon receptor | 1 | 0.78 | 1 | Dimethyl Quercetin |
| ATP-dependent translocase ABCB1 | 1 | 0.78 | 3 | Dimethyl Quercetin |
| Carbonic anhydrase 12 | 1 | 0.92 | 2 | Dimethyl Quercetin |
| Carbonic anhydrase 4 | 1 | 0.94 | 1 | Dimethyl Quercetin |
| Carbonic anhydrase 7 | 1 | 0.92 | 2 | Dimethyl Quercetin |
| Cholinesterase | 1 | 0.7 | 3 | Dimethyl Quercetin |
| D(1B) dopamine receptor | 1 | 0.92 | 1 | Dimethyl Quercetin |
| Dipeptidyl peptidase 4 | 1 | 0.91 | 1 | Dimethyl Quercetin |
| Dual specificity mitogen-activated protein kinase kinase 4 | 1 | 0.91 | 1 | Dimethyl Quercetin |
| Epidermal growth factor receptor | 1 | 0.91 | 4 | Dimethyl Quercetin |
| Estrogen receptor | 1 | 0.91 | 2 | Dimethyl Quercetin |
| Estrogen receptor beta | 1 | 0.91 | 5 | Dimethyl Quercetin |
| Histamine H3 receptor | 1 | 0.82 | 5 | Dimethyl Quercetin |
| Isoform 1 of Steroid hormone receptor ERR2 (ERRbeta2-delta10) | 1 | 0.91 | 1 | Dimethyl Quercetin |
| Macrophage migration inhibitory factor | 1 | 0.77 | 6 | Dimethyl Quercetin |
| Polyunsaturated fatty acid 5-lipoxygenase | 1 | 0.75 | 7 | Dimethyl Quercetin |
| Polyunsaturated fatty acid lipoxygenase ALOX12 | 1 | 0.73 | 4 | Dimethyl Quercetin |
| Polyunsaturated fatty acid lipoxygenase ALOX15 | 1 | 0.76 | 1 | Dimethyl Quercetin |
| Receptor-type tyrosine-protein phosphatase S | 1 | 0.8 | 1 | Dimethyl Quercetin |
| Smoothened homolog | 1 | 0.73 | 1 | Dimethyl Quercetin |
| Sphingosine 1-phosphate receptor 1 | 1 | 0.87 | 1 | Dimethyl Quercetin |
| Steroid hormone receptor ERR1 | 1 | 0.91 | 1 | Dimethyl Quercetin |
| Steroidogenic factor 1 | 1 | 0.77 | 1 | Dimethyl Quercetin |
| Steryl-sulfatase | 1 | 0.73 | 1 | Dimethyl Quercetin |
| Sulfotransferase 1A1 | 1 | 0.91 | 2 | Dimethyl Quercetin |
| Fatty acid-binding protein 5 | 1 | 0.81 | 1 | butolic acid |
| Fatty acid-binding protein 5 [M35A,L60A] | 1 | 0.81 | 1 | butolic acid |
| Fatty acid-binding protein, adipocyte | 1 | 0.81 | 1 | butolic acid |
| Fatty acid-binding protein, liver | 1 | 0.7 | 1 | butolic acid |
| Free fatty acid receptor 1 | 1 | 0.81 | 1 | butolic acid |
| G-protein coupled receptor 84 | 1 | 0.85 | 5 | butolic acid |
| Solute carrier family 22 member 6 | 1 | 0.73 | 1 | butolic acid |
| Fatty acid-binding protein 5 | 1 | 0.81 | 1 | butolic acid |
| Fatty acid-binding protein 5 [M35A,L60A] | 1 | 0.81 | 1 | butolic acid |
| Fatty acid-binding protein, adipocyte | 1 | 0.81 | 1 | butolic acid |
| Fatty acid-binding protein, liver | 1 | 0.7 | 1 | butolic acid |
| Free fatty acid receptor 1 | 1 | 0.81 | 1 | butolic acid |
| G-protein coupled receptor 84 | 1 | 0.85 | 5 | butolic acid |
| Solute carrier family 22 member 6 | 1 | 0.73 | 1 | butolic acid |

**Supplementary Table S7. Results of the Swiss Target Prediction.**

| **Target** | **Common name** | **Uniprot ID** | **ChEMBL ID** | **Target Class** | **Probability*** | **Known actives (3D/2D)** | **Phytoconstituents** |
| --- | --- | --- | --- | --- | --- | --- | --- |
| Gamma-amino-N-butyrate transaminase (by homology) | ABAT | P80404 | CHEMBL2044 | Transferase | 0.03397069 | 2 / 0 | Alanine |
| Thymidylate synthase (by homology) | TYMS | P04818 | CHEMBL1952 | Transferase | 0.03397069 | 1 / 0 | Allophonic acid |
| Amine oxidase, copper containing | AOC3 | Q16853 | CHEMBL3437 | Enzyme | 0.023832743 | 2 / 0 | Allophonic acid |
| HMG-CoA reductase | HMGCR | P04035 | CHEMBL402 | Oxidoreductase | 0.680955494 | 54 / 7 | Bita sitosterol |
| Cytochrome P450 51 (by homology) | CYP51A1 | Q16850 | CHEMBL3849 | Cytochrome P450 | 0.680955494 | 3 / 2 | Bita sitosterol |
| Androgen Receptor | AR | P10275 | CHEMBL1871 | Nuclear receptor | 0.656003572 | 26 / 102 | Bita sitosterol |
| Niemann-Pick C1-like protein 1 | NPC1L1 | Q9UHC9 | CHEMBL2027 | Other membrane protein | 0.647646922 | 9 / 13 | Bita sitosterol |
| LXR-alpha | NR1H3 | Q13133 | CHEMBL2808 | Nuclear receptor | 0.622655937 | 19 / 20 | Bita sitosterol |
| Cytochrome P450 17A1 | CYP17A1 | P05093 | CHEMBL3522 | Cytochrome P450 | 0.506040802 | 48 / 50 | Bita sitosterol |
| Nuclear receptor ROR-gamma | RORC | P51449 | CHEMBL1741186 | Nuclear receptor | 0.431038563 | 11 / 9 | Bita sitosterol |
| Cytochrome P450 19A1 | CYP19A1 | P11511 | CHEMBL1978 | Cytochrome P450 | 0.314405075 | 10 / 161 | Bita sitosterol |
| Estrogen receptor beta | ESR2 | Q92731 | CHEMBL242 | Nuclear receptor | 0.306043655 | 5 / 47 | Bita sitosterol |
| Estrogen receptor alpha | ESR1 | P03372 | CHEMBL206 | Nuclear receptor | 0.222798333 | 6 / 39 | Bita sitosterol |
| Testis-specific androgen-binding protein | SHBG | P04278 | CHEMBL3305 | Secreted protein | 0.189458322 | 1 / 48 | Bita sitosterol |
| Sterol regulatory element-binding protein 2 | SREBF2 | Q12772 | CHEMBL1795166 | Unclassified protein | 0.172809876 | 1 / 1 | Bita sitosterol |
| Cytochrome P450 2C19 | CYP2C19 | P33261 | CHEMBL3622 | Cytochrome P450 | 0.131155749 | 0 / 3 | Bita sitosterol |
| Norepinephrine transporter | SLC6A2 | P23975 | CHEMBL222 | Electrochemical transporter | 0.12282732 | 2 / 2 | Bita sitosterol |
| Butyrylcholinesterase | BCHE | P06276 | CHEMBL1914 | Hydrolase | 0.12282732 | 3 / 2 | Bita sitosterol |
| Nuclear receptor ROR-alpha | RORA | P35398 | CHEMBL5868 | Nuclear receptor | 0.12282732 | 3 / 3 | Bita sitosterol |
| Protein-tyrosine phosphatase 1B | PTPN1 | P18031 | CHEMBL335 | Phosphatase | 0.12282732 | 14 / 47 | Bita sitosterol |
| Corticosteroid binding globulin | SERPINA6 | P08185 | CHEMBL2421 | Secreted protein | 0.11449479 | 0 / 18 | Bita sitosterol |
| Serotonin transporter | SLC6A4 | P31645 | CHEMBL228 | Electrochemical transporter | 0.106165761 | 12 / 5 | Bita sitosterol |
| Glucose-6-phosphate 1-dehydrogenase | G6PD | P11413 | CHEMBL5347 | Enzyme | 0.106165761 | 4 / 2 | Bita sitosterol |
| Nuclear receptor subfamily 1 group I member 3 (by homology) | NR1I3 | Q14994 | CHEMBL5503 | Nuclear receptor | 0.106165761 | 0 / 3 | Bita sitosterol |
| Muscarinic acetylcholine receptor M2 | CHRM2 | P08172 | CHEMBL211 | Family A G protein-coupled receptor | 0.106165761 | 1 / 1 | Bita sitosterol |
| Acetylcholinesterase | ACHE | P22303 | CHEMBL220 | Hydrolase | 0.106165761 | 3 / 1 | Bita sitosterol |
| Vitamin D receptor | VDR | P11473 | CHEMBL1977 | Nuclear receptor | 0.106165761 | 17 / 49 | Bita sitosterol |
| Carboxylesterase 2 | CES2 | O00748 | CHEMBL3180 | Enzyme | 0.106165761 | 6 / 10 | Bita sitosterol |
| LXR-beta | NR1H2 | P55055 | CHEMBL4093 | Nuclear receptor | 0.106165761 | 6 / 1 | Bita sitosterol |
| Dual specificity phosphatase Cdc25A | CDC25A | P30304 | CHEMBL3775 | Phosphatase | 0.106165761 | 5 / 11 | Bita sitosterol |
| Prostanoid EP1 receptor (by homology) | PTGER1 | P34995 | CHEMBL1811 | Family A G protein-coupled receptor | 0.106165761 | 0 / 9 | Bita sitosterol |
| Prostanoid EP2 receptor (by homology) | PTGER2 | P43116 | CHEMBL1881 | Family A G protein-coupled receptor | 0.106165761 | 0 / 20 | Bita sitosterol |
| 11-beta-hydroxysteroid dehydrogenase 1 | HSD11B1 | P28845 | CHEMBL4235 | Enzyme | 0.106165761 | 61 / 23 | Bita sitosterol |
| Anti-estrogen binding site (AEBS) (by homology) | DHCR7 | Q9UBM7 | CHEMBL2169735 | Enzyme | 0.106165761 | 3 / 5 | Bita sitosterol |
| Glycine receptor subunit alpha-1 | GLRA1 | P23415 | CHEMBL5845 | Ligand-gated ion channel | 0.106165761 | 1 / 1 | Bita sitosterol |
| Peroxisome proliferator-activated receptor delta | PPARD | Q03181 | CHEMBL3979 | Nuclear receptor | 0.106165761 | 0 / 8 | Bita sitosterol |
| Squalene monooxygenase | SQLE | Q14534 | CHEMBL3592 | Enzyme | 0.106165761 | 0 / 4 | Bita sitosterol |
| Protein-tyrosine phosphatase 1C | PTPN6 | P29350 | CHEMBL3166 | Phosphatase | 0.106165761 | 0 / 1 | Bita sitosterol |
| Squalene synthetase (by homology) | FDFT1 | P37268 | CHEMBL3338 | Enzyme | 0.106165761 | 0 / 1 | Bita sitosterol |
| Nitric oxide synthase, inducible (by homology) | NOS2 | P35228 | CHEMBL4481 | Enzyme | 0.106165761 | 2 / 16 | Bita sitosterol |
| Glucocorticoid receptor | NR3C1 | P04150 | CHEMBL2034 | Nuclear receptor | 0.106165761 | 15 / 25 | Bita sitosterol |
| Dual specificity phosphatase Cdc25B | CDC25B | P30305 | CHEMBL4804 | Phosphatase | 0.106165761 | 5 / 7 | Bita sitosterol |
| Sonic hedgehog protein (by homology) | SHH | Q15465 | CHEMBL5602 | Unclassified protein | 0.106165761 | 0 / 1 | Bita sitosterol |
| UDP-glucuronosyltransferase 2B7 | UGT2B7 | P16662 | CHEMBL4370 | Enzyme | 0.106165761 | 0 / 8 | Bita sitosterol |
| 11-beta-hydroxysteroid dehydrogenase 2 | HSD11B2 | P80365 | CHEMBL3746 | Enzyme | 0.106165761 | 8 / 8 | Bita sitosterol |
| Dopamine D2 receptor | DRD2 | P14416 | CHEMBL217 | Family A G protein-coupled receptor | 0.106165761 | 16 / 0 | Bita sitosterol |
| DNA polymerase beta (by homology) | POLB | P06746 | CHEMBL2392 | Enzyme | 0.106165761 | 0 / 6 | Bita sitosterol |
| HMG-CoA reductase | HMGCR | P04035 | CHEMBL402 | Oxidoreductase | 0.680955494 | 54 / 7 | Bita sitosterol |
| Cytochrome P450 51 (by homology) | CYP51A1 | Q16850 | CHEMBL3849 | Cytochrome P450 | 0.680955494 | 3 / 2 | Bita sitosterol |
| Androgen Receptor | AR | P10275 | CHEMBL1871 | Nuclear receptor | 0.656003572 | 26 / 102 | Bita sitosterol |
| Niemann-Pick C1-like protein 1 | NPC1L1 | Q9UHC9 | CHEMBL2027 | Other membrane protein | 0.647646922 | 9 / 13 | Bita sitosterol |
| LXR-alpha | NR1H3 | Q13133 | CHEMBL2808 | Nuclear receptor | 0.622655937 | 19 / 20 | Bita sitosterol |
| Cytochrome P450 17A1 | CYP17A1 | P05093 | CHEMBL3522 | Cytochrome P450 | 0.506040802 | 48 / 50 | Bita sitosterol |
| Nuclear receptor ROR-gamma | RORC | P51449 | CHEMBL1741186 | Nuclear receptor | 0.431038563 | 11 / 9 | Bita sitosterol |
| Cytochrome P450 19A1 | CYP19A1 | P11511 | CHEMBL1978 | Cytochrome P450 | 0.314405075 | 10 / 161 | Bita sitosterol |
| Estrogen receptor beta | ESR2 | Q92731 | CHEMBL242 | Nuclear receptor | 0.306043655 | 5 / 47 | Bita sitosterol |
| Estrogen receptor alpha | ESR1 | P03372 | CHEMBL206 | Nuclear receptor | 0.222798333 | 6 / 39 | Bita sitosterol |
| Testis-specific androgen-binding protein | SHBG | P04278 | CHEMBL3305 | Secreted protein | 0.189458322 | 1 / 48 | Bita sitosterol |
| Sterol regulatory element-binding protein 2 | SREBF2 | Q12772 | CHEMBL1795166 | Unclassified protein | 0.172809876 | 1 / 1 | Bita sitosterol |
| Cytochrome P450 2C19 | CYP2C19 | P33261 | CHEMBL3622 | Cytochrome P450 | 0.131155749 | 0 / 3 | Bita sitosterol |
| Norepinephrine transporter | SLC6A2 | P23975 | CHEMBL222 | Electrochemical transporter | 0.12282732 | 2 / 2 | Bita sitosterol |
| Butyrylcholinesterase | BCHE | P06276 | CHEMBL1914 | Hydrolase | 0.12282732 | 3 / 2 | Bita sitosterol |
| Nuclear receptor ROR-alpha | RORA | P35398 | CHEMBL5868 | Nuclear receptor | 0.12282732 | 3 / 3 | Bita sitosterol |
| Protein-tyrosine phosphatase 1B | PTPN1 | P18031 | CHEMBL335 | Phosphatase | 0.12282732 | 14 / 47 | Bita sitosterol |
| Corticosteroid binding globulin | SERPINA6 | P08185 | CHEMBL2421 | Secreted protein | 0.11449479 | 0 / 18 | Bita sitosterol |
| Serotonin transporter | SLC6A4 | P31645 | CHEMBL228 | Electrochemical transporter | 0.106165761 | 12 / 5 | Bita sitosterol |
| Glucose-6-phosphate 1-dehydrogenase | G6PD | P11413 | CHEMBL5347 | Enzyme | 0.106165761 | 4 / 2 | Bita sitosterol |
| Nuclear receptor subfamily 1 group I member 3 (by homology) | NR1I3 | Q14994 | CHEMBL5503 | Nuclear receptor | 0.106165761 | 0 / 3 | Bita sitosterol |
| Muscarinic acetylcholine receptor M2 | CHRM2 | P08172 | CHEMBL211 | Family A G protein-coupled receptor | 0.106165761 | 1 / 1 | Bita sitosterol |
| Acetylcholinesterase | ACHE | P22303 | CHEMBL220 | Hydrolase | 0.106165761 | 3 / 1 | Bita sitosterol |
| Vitamin D receptor | VDR | P11473 | CHEMBL1977 | Nuclear receptor | 0.106165761 | 17 / 49 | Bita sitosterol |
| Carboxylesterase 2 | CES2 | O00748 | CHEMBL3180 | Enzyme | 0.106165761 | 6 / 10 | Bita sitosterol |
| LXR-beta | NR1H2 | P55055 | CHEMBL4093 | Nuclear receptor | 0.106165761 | 6 / 1 | Bita sitosterol |
| Dual specificity phosphatase Cdc25A | CDC25A | P30304 | CHEMBL3775 | Phosphatase | 0.106165761 | 5 / 11 | Bita sitosterol |
| Prostanoid EP1 receptor (by homology) | PTGER1 | P34995 | CHEMBL1811 | Family A G protein-coupled receptor | 0.106165761 | 0 / 9 | Bita sitosterol |
| Prostanoid EP2 receptor (by homology) | PTGER2 | P43116 | CHEMBL1881 | Family A G protein-coupled receptor | 0.106165761 | 0 / 20 | Bita sitosterol |
| 11-beta-hydroxysteroid dehydrogenase 1 | HSD11B1 | P28845 | CHEMBL4235 | Enzyme | 0.106165761 | 61 / 23 | Bita sitosterol |
| Anti-estrogen binding site (AEBS) (by homology) | DHCR7 | Q9UBM7 | CHEMBL2169735 | Enzyme | 0.106165761 | 3 / 5 | Bita sitosterol |
| Glycine receptor subunit alpha-1 | GLRA1 | P23415 | CHEMBL5845 | Ligand-gated ion channel | 0.106165761 | 1 / 1 | Bita sitosterol |
| Peroxisome proliferator-activated receptor delta | PPARD | Q03181 | CHEMBL3979 | Nuclear receptor | 0.106165761 | 0 / 8 | Bita sitosterol |
| Squalene monooxygenase | SQLE | Q14534 | CHEMBL3592 | Enzyme | 0.106165761 | 0 / 4 | Bita sitosterol |
| Protein-tyrosine phosphatase 1C | PTPN6 | P29350 | CHEMBL3166 | Phosphatase | 0.106165761 | 0 / 1 | Bita sitosterol |
| Squalene synthetase (by homology) | FDFT1 | P37268 | CHEMBL3338 | Enzyme | 0.106165761 | 0 / 1 | Bita sitosterol |
| Nitric oxide synthase, inducible (by homology) | NOS2 | P35228 | CHEMBL4481 | Enzyme | 0.106165761 | 2 / 16 | Bita sitosterol |
| Glucocorticoid receptor | NR3C1 | P04150 | CHEMBL2034 | Nuclear receptor | 0.106165761 | 15 / 25 | Bita sitosterol |
| Dual specificity phosphatase Cdc25B | CDC25B | P30305 | CHEMBL4804 | Phosphatase | 0.106165761 | 5 / 7 | Bita sitosterol |
| Sonic hedgehog protein (by homology) | SHH | Q15465 | CHEMBL5602 | Unclassified protein | 0.106165761 | 0 / 1 | Bita sitosterol |
| UDP-glucuronosyltransferase 2B7 | UGT2B7 | P16662 | CHEMBL4370 | Enzyme | 0.106165761 | 0 / 8 | Bita sitosterol |
| 11-beta-hydroxysteroid dehydrogenase 2 | HSD11B2 | P80365 | CHEMBL3746 | Enzyme | 0.106165761 | 8 / 8 | Bita sitosterol |
| Dopamine D2 receptor | DRD2 | P14416 | CHEMBL217 | Family A G protein-coupled receptor | 0.106165761 | 16 / 0 | Bita sitosterol |
| DNA polymerase beta (by homology) | POLB | P06746 | CHEMBL2392 | Enzyme | 0.106165761 | 0 / 6 | Bita sitosterol |
| HMG-CoA reductase | HMGCR | P04035 | CHEMBL402 | Oxidoreductase | 0.680955494 | 54 / 7 | Bita sitosterol |
| Cytochrome P450 19A1 | CYP19A1 | P11511 | CHEMBL1978 | Cytochrome P450 | 1 | 23 / 51 | Butin |
| Carbonic anhydrase VII | CA7 | P43166 | CHEMBL2326 | Lyase | 0.559044784 | 15 / 8 | Butin |
| Carbonic anhydrase XII | CA12 | O43570 | CHEMBL3242 | Lyase | 0.559044784 | 30 / 7 | Butin |
| Carbonic anhydrase IV | CA4 | P22748 | CHEMBL3729 | Lyase | 0.559044784 | 10 / 8 | Butin |
| Cytochrome P450 1B1 | CYP1B1 | Q16678 | CHEMBL4878 | Cytochrome P450 | 0.559044784 | 6 / 6 | Butin |
| Estrogen receptor alpha | ESR1 | P03372 | CHEMBL206 | Nuclear receptor | 0.329868485 | 254 / 85 | Butin |
| Estrogen receptor beta | ESR2 | Q92731 | CHEMBL242 | Nuclear receptor | 0.329868485 | 252 / 77 | Butin |
| Multidrug resistance-associated protein 1 | ABCC1 | P33527 | CHEMBL3004 | Primary active transporter | 0.29705997 | 2 / 6 | Butin |
| Estradiol 17-beta-dehydrogenase 1 | HSD17B1 | P14061 | CHEMBL3181 | Enzyme | 0.29705997 | 97 / 2 | Butin |
| Testis-specific androgen-binding protein | SHBG | P04278 | CHEMBL3305 | Secreted protein | 0.29705997 | 2 / 3 | Butin |
| Carbonyl reductase [NADPH] 1 | CBR1 | P16152 | CHEMBL5586 | Enzyme | 0.29705997 | 1 / 1 | Butin |
| Monoamine oxidase B | MAOB | P27338 | CHEMBL2039 | Oxidoreductase | 0.182484829 | 12 / 123 | Butin |
| Cyclooxygenase-1 | PTGS1 | P23219 | CHEMBL221 | Oxidoreductase | 0.149732594 | 8 / 13 | Butin |
| ATP-binding cassette sub-family G member 2 | ABCG2 | Q9UNQ0 | CHEMBL5393 | Primary active transporter | 0.116965063 | 4 / 11 | Butin |
| Taste receptor type 2 member 31 | TAS2R31 | P59538 | CHEMBL2034804 | Taste family G protein-coupled receptor | 0.108770969 | 1 / 3 | Butin |
| Adenosine A1 receptor (by homology) | ADORA1 | P30542 | CHEMBL226 | Family A G protein-coupled receptor | 0.108770969 | 6 / 3 | Butin |
| Adenosine A3 receptor | ADORA3 | P0DMS8 | CHEMBL256 | Family A G protein-coupled receptor | 0.108770969 | 3 / 3 | Butin |
| Aldo-keto-reductase family 1 member C3 | AKR1C3 | P42330 | CHEMBL4681 | Enzyme | 0.108770969 | 0 / 11 | Butin |
| Matrix metalloproteinase 13 | MMP13 | P45452 | CHEMBL280 | Protease | 0.108770969 | 12 / 1 | Butin |
| Matrix metalloproteinase 12 | MMP12 | P39900 | CHEMBL4393 | Protease | 0.108770969 | 7 / 3 | Butin |
| Metabotropic glutamate receptor 5 | GRM5 | P41594 | CHEMBL3227 | Family C G protein-coupled receptor | 0.100578902 | 0 / 10 | Butin |
| Phospholipase A2 group 1B | PLA2G1B | P04054 | CHEMBL4426 | Enzyme | 0.100578902 | 0 / 1 | Butin |
| Acetylcholinesterase | ACHE | P22303 | CHEMBL220 | Hydrolase | 0.100578902 | 0 / 40 | Butin |
| Kallikrein 1 | KLK1 | P06870 | CHEMBL2319 | Protease | 0.100578902 | 0 / 1 | Butin |
| Kallikrein 2 | KLK2 | P20151 | CHEMBL2442 | Protease | 0.100578902 | 0 / 1 | Butin |
| Beta-secretase 1 | BACE1 | P56817 | CHEMBL4822 | Protease | 0.100578902 | 14 / 15 | Butin |
| Acyl coenzyme A:cholesterol acyltransferase | CES1 | P23141 | CHEMBL2265 | Enzyme | 0.100578902 | 0 / 1 | Butin |
| Peroxisome proliferator-activated receptor gamma | PPARG | P37231 | CHEMBL235 | Nuclear receptor | 0.100578902 | 0 / 115 | Butin |
| Carboxylesterase 2 | CES2 | O00748 | CHEMBL3180 | Enzyme | 0.100578902 | 0 / 1 | Butin |
| Stem cell growth factor receptor | KIT | P10721 | CHEMBL1936 | Kinase | 0.100578902 | 2 / 3 | Butin |
| Tyrosine-protein kinase SRC | SRC | P12931 | CHEMBL267 | Kinase | 0.100578902 | 32 / 4 | Butin |
| Vascular endothelial growth factor receptor 2 | KDR | P35968 | CHEMBL279 | Kinase | 0.100578902 | 7 / 12 | Butin |
| Fibroblast growth factor receptor 1 | FGFR1 | P11362 | CHEMBL3650 | Kinase | 0.100578902 | 1 / 3 | Butin |
| Hepatocyte growth factor receptor | MET | P08581 | CHEMBL3717 | Kinase | 0.100578902 | 10 / 5 | Butin |
| Carbonic anhydrase II | CA2 | P00918 | CHEMBL205 | Lyase | 0.100578902 | 66 / 4 | Butin |
| Carbonic anhydrase I | CA1 | P00915 | CHEMBL261 | Lyase | 0.100578902 | 54 / 3 | Butin |
| Sodium/glucose cotransporter 2 | SLC5A2 | P31639 | CHEMBL3884 | Electrochemical transporter | 0.100578902 | 0 / 3 | Butin |
| Arachidonate 12-lipoxygenase | ALOX12 | P18054 | CHEMBL3687 | Enzyme | 0.100578902 | 9 / 4 | Butin |
| DNA polymerase beta (by homology) | POLB | P06746 | CHEMBL2392 | Enzyme | 0.100578902 | 0 / 3 | Butin |
| Neuronal acetylcholine receptor protein alpha-7 subunit | CHRNA7 | P36544 | CHEMBL2492 | Ligand-gated ion channel | 0.100578902 | 9 / 0 | Butin |
| Endothelin receptor ET-A (by homology) | EDNRA | P25101 | CHEMBL252 | Family A G protein-coupled receptor | 0.100578902 | 0 / 75 | Butin |
| Phospholipase A2 group IIA | PLA2G2A | P14555 | CHEMBL3474 | Enzyme | 0.100578902 | 0 / 8 | Butin |
| Phospholipase A2 group V | PLA2G5 | P39877 | CHEMBL4323 | Enzyme | 0.100578902 | 0 / 3 | Butin |
| Group X secretory phospholipase A2 | PLA2G10 | O15496 | CHEMBL4342 | Enzyme | 0.100578902 | 0 / 1 | Butin |
| Carbonic anhydrase III | CA3 | P07451 | CHEMBL2885 | Lyase | 0.100578902 | 2 / 2 | Butin |
| Carbonic anhydrase VI | CA6 | P23280 | CHEMBL3025 | Lyase | 0.100578902 | 4 / 2 | Butin |
| Carbonic anhydrase XIII | CA13 | Q8N1Q1 | CHEMBL3912 | Lyase | 0.100578902 | 3 / 2 | Butin |
| Carbonic anhydrase VB | CA5B | Q9Y2D0 | CHEMBL3969 | Lyase | 0.100578902 | 5 / 2 | Butin |
| Carbonic anhydrase VA | CA5A | P35218 | CHEMBL4789 | Lyase | 0.100578902 | 6 / 3 | Butin |
| Plasminogen activator inhibitor-1 | SERPINE1 | P05121 | CHEMBL3475 | Secreted protein | 0.100578902 | 5 / 5 | Butin |
| Matrix metalloproteinase 2 | MMP2 | P08253 | CHEMBL333 | Protease | 0.100578902 | 54 / 7 | Butin |
| Dual-specificity tyrosine-phosphorylation regulated kinase 1A | DYRK1A | Q13627 | CHEMBL2292 | Kinase | 0.100578902 | 24 / 1 | Butin |
| Beta amyloid A4 protein | APP | P05067 | CHEMBL2487 | Membrane receptor | 0.100578902 | 11 / 3 | Butin |
| Carbonic anhydrase IX | CA9 | Q16790 | CHEMBL3594 | Lyase | 0.100578902 | 36 / 1 | Butin |
| Matrix metalloproteinase 9 | MMP9 | P14780 | CHEMBL321 | Protease | 0.100578902 | 56 / 1 | Butin |
| Microtubule-associated protein tau | MAPT | P10636 | CHEMBL1293224 | Unclassified protein | 0.100578902 | 3 / 1 | Butin |
| Retinoid X receptor alpha | RXRA | P19793 | CHEMBL2061 | Nuclear receptor | 0.100578902 | 0 / 17 | Butin |
| NADPH oxidase 4 | NOX4 | Q9NPH5 | CHEMBL1250375 | Enzyme | 0.100578902 | 10 / 0 | Butin |
| Estrogen-related receptor alpha | ESRRA | P11474 | CHEMBL3429 | Nuclear receptor | 0.100578902 | 18 / 0 | Butin |
| 17-beta-hydroxysteroid dehydrogenase 14 | HSD17B14 | Q9BPX1 | CHEMBL3712868 | Enzyme | 0.100578902 | 1 / 0 | Butin |
| Estrogen-related receptor beta | ESRRB | O95718 | CHEMBL3751 | Nuclear receptor | 0.100578902 | 18 / 0 | Butin |
| Apoptosis regulator Bcl-2 | BCL2 | P10415 | CHEMBL4860 | Other ion channel | 0.100578902 | 7 / 10 | Butin |
| Dual specificty protein kinase CLK1 | CLK1 | P49759 | CHEMBL4224 | Kinase | 0.100578902 | 14 / 0 | Butin |
| Metabotropic glutamate receptor 2 (by homology) | GRM2 | Q14416 | CHEMBL5137 | Family C G protein-coupled receptor | 0.100578902 | 0 / 72 | Butin |
| DNA (cytosine-5)-methyltransferase 1 | DNMT1 | P26358 | CHEMBL1993 | Writer | 0.100578902 | 0 / 1 | Butin |
| CMP-N-acetylneuraminate-beta-1,4-galactoside alpha-2,3-sialyltransferase | ST3GAL3 | Q11203 | CHEMBL3596076 | Transferase | 0.100578902 | 0 / 1 | Butin |
| Alpha-(1,3)-fucosyltransferase 7 | FUT7 | Q11130 | CHEMBL3596077 | Transferase | 0.100578902 | 0 / 1 | Butin |
| Fucosyltransferase 4 | FUT4 | P22083 | CHEMBL4996 | Enzyme | 0.100578902 | 0 / 1 | Butin |
| Signal transducer and activator of transcription 1-alpha/beta | STAT1 | P42224 | CHEMBL6101 | Transcription factor | 0.100578902 | 0 / 1 | Butin |
| Squalene monooxygenase (by homology) | SQLE | Q14534 | CHEMBL3592 | Enzyme | 0.100578902 | 0 / 6 | Butin |
| Quinone reductase 2 | NQO2 | P16083 | CHEMBL3959 | Enzyme | 0.100578902 | 6 / 0 | Butin |
| Estradiol 17-beta-dehydrogenase 2 | HSD17B2 | P37059 | CHEMBL2789 | Enzyme | 0.100578902 | 91 / 0 | Butin |
| Dual specificity tyrosine-phosphorylation-regulated kinase 1B | DYRK1B | Q9Y463 | CHEMBL5543 | Kinase | 0.100578902 | 13 / 0 | Butin |
| Insulin-like growth factor I receptor | IGF1R | P08069 | CHEMBL1957 | Kinase | 0.100578902 | 23 / 0 | Butin |
| Insulin receptor | INSR | P06213 | CHEMBL1981 | Kinase | 0.100578902 | 5 / 0 | Butin |
| Insulin-like growth factor binding protein 3 | IGFBP3 | P17936 | CHEMBL3997 | Secreted protein | 0.100578902 | 1 / 0 | Butin |
| 14-3-3 protein gamma | YWHAG | P61981 | CHEMBL1293296 | Unclassified protein | 0.100578902 | 1 / 0 | Butin |
| Glycogen synthase kinase-3 beta | GSK3B | P49841 | CHEMBL262 | Kinase | 0.100578902 | 22 / 0 | Butin |
| Tyrosine-protein kinase LCK | LCK | P06239 | CHEMBL258 | Kinase | 0.100578902 | 12 / 0 | Butin |
| Tyrosine-protein kinase SYK | SYK | P43405 | CHEMBL2599 | Kinase | 0.100578902 | 6 / 0 | Butin |
| Steryl-sulfatase | STS | P08842 | CHEMBL3559 | Enzyme | 0.100578902 | 3 / 7 | Butin |
| Free fatty acid receptor 1 | FFAR1 | O14842 | CHEMBL4422 | Family A G protein-coupled receptor | 0.100578902 | 0 / 126 | Butin |
| Hypoxia-inducible factor 1 alpha | HIF1A | Q16665 | CHEMBL4261 | Transcription factor | 0.100578902 | 0 / 4 | Butin |
| Cyclin-dependent kinase 5/CDK5 activator 1 | CDK5R1 CDK5 | Q15078 Q00535 | CHEMBL1907600 | Kinase | 0.100578902 | 6 / 0 | Butin |
| Alpha-synuclein | SNCA | P37840 | CHEMBL6152 | Unclassified protein | 0.100578902 | 9 / 0 | Butin |
| Serine/threonine-protein kinase AKT | AKT1 | P31749 | CHEMBL4282 | Kinase | 0.100578902 | 0 / 16 | Butin |
| Serine/threonine-protein kinase/endoribonuclease IRE1 | ERN1 | O75460 | CHEMBL1163101 | Enzyme | 0.100578902 | 22 / 0 | Butin |
| P-glycoprotein 1 | ABCB1 | P08183 | CHEMBL4302 | Primary active transporter | 0.100578902 | 1 / 207 | Butin |
| Coagulation factor VII/tissue factor | F3 | P13726 | CHEMBL4081 | Surface antigen | 0.100578902 | 7 / 0 | Butin |
| Cathepsin (B and K) | CTSB | P07858 | CHEMBL4072 | Protease | 0.100578902 | 0 / 1 | Butin |
| Transitional endoplasmic reticulum ATPase | VCP | P55072 | CHEMBL1075145 | Primary active transporter | 0.100578902 | 4 / 0 | Butin |
| Aldose reductase | AKR1B1 | P15121 | CHEMBL1900 | Enzyme | 0.100578902 | 39 / 43 | Butin |
| Serine/threonine-protein kinase WEE1 | WEE1 | P30291 | CHEMBL5491 | Kinase | 0.100578902 | 43 / 0 | Butin |
| Ornithine decarboxylase | ODC1 | P11926 | CHEMBL1869 | Lyase | 0.100578902 | 0 / 5 | Butin |
| Free fatty acid receptor 1 | FFAR1 | O14842 | CHEMBL4422 | Family A G protein-coupled receptor | 0.346092208 | 163 / 3 | Butolic Acid |
| Fatty acid binding protein intestinal | FABP2 | P12104 | CHEMBL4879 | Fatty acid binding protein family | 0.33721581 | 0 / 1 | Butolic Acid |
| Peroxisome proliferator-activated receptor alpha | PPARA | Q07869 | CHEMBL239 | Nuclear receptor | 0.300846746 | 140 / 9 | Butolic Acid |
| Peroxisome proliferator-activated receptor delta | PPARD | Q03181 | CHEMBL3979 | Nuclear receptor | 0.300846746 | 107 / 7 | Butolic Acid |
| Fatty acid binding protein adipocyte | FABP4 | P15090 | CHEMBL2083 | Fatty acid binding protein family | 0.256136198 | 25 / 3 | Butolic Acid |
| Fatty acid binding protein muscle | FABP3 | P05413 | CHEMBL3344 | Fatty acid binding protein family | 0.256136198 | 11 / 5 | Butolic Acid |
| Fatty acid binding protein epidermal | FABP5 | Q01469 | CHEMBL3674 | Fatty acid binding protein family | 0.193083813 | 2 / 2 | Butolic Acid |
| 11-beta-hydroxysteroid dehydrogenase 1 | HSD11B1 | P28845 | CHEMBL4235 | Enzyme | 0.166091486 | 59 / 22 | Butolic Acid |
| Solute carrier family 22 member 6 (by homology) | SLC22A6 | Q4U2R8 | CHEMBL1641347 | Electrochemical transporter | 0.157080105 | 1 / 2 | Butolic Acid |
| Lysine-specific demethylase 2A | KDM2A | Q9Y2K7 | CHEMBL1938210 | Eraser | 0.13892441 | 25 / 1 | Butolic Acid |
| Histone lysine demethylase PHF8 | PHF8 | Q9UPP1 | CHEMBL1938212 | Eraser | 0.13892441 | 2 / 1 | Butolic Acid |
| Bile acid receptor FXR | NR1H4 | Q96RI1 | CHEMBL2047 | Nuclear receptor | 0.112023618 | 95 / 11 | Butolic Acid |
| Vitamin D receptor | VDR | P11473 | CHEMBL1977 | Nuclear receptor | 0.084997247 | 6 / 2 | Butolic Acid |
| G-protein coupled bile acid receptor 1 | GPBAR1 | Q8TDU6 | CHEMBL5409 | Family A G protein-coupled receptor | 0.084997247 | 25 / 15 | Butolic Acid |
| Prostanoid EP2 receptor | PTGER2 | P43116 | CHEMBL1881 | Family A G protein-coupled receptor | 0.075973634 | 213 / 5 | Butolic Acid |
| Corticosteroid binding globulin | SERPINA6 | P08185 | CHEMBL2421 | Secreted protein | 0.075973634 | 0 / 6 | Butolic Acid |
| Testis-specific androgen-binding protein | SHBG | P04278 | CHEMBL3305 | Secreted protein | 0.075973634 | 0 / 26 | Butolic Acid |
| Estradiol 17-beta-dehydrogenase 3 | HSD17B3 | P37058 | CHEMBL4234 | Enzyme | 0.075973634 | 0 / 11 | Butolic Acid |
| Glucose-6-phosphate 1-dehydrogenase | G6PD | P11413 | CHEMBL5347 | Enzyme | 0.075973634 | 0 / 9 | Butolic Acid |
| GABA-B receptor (by homology) | GABBR1 | Q9UBS5 | CHEMBL2064 | Family C G protein-coupled receptor | 0.075973634 | 0 / 1 | Butolic Acid |
| Dual specificity phosphatase Cdc25A | CDC25A | P30304 | CHEMBL3775 | Phosphatase | 0.075973634 | 7 / 14 | Butolic Acid |
| Cytochrome P450 19A1 | CYP19A1 | P11511 | CHEMBL1978 | Cytochrome P450 | 0.066975016 | 2 / 22 | Butolic Acid |
| Aldo-keto reductase family 1 member B10 | AKR1B10 | O60218 | CHEMBL5983 | Enzyme | 0.066975016 | 3 / 7 | Butolic Acid |
| Prostanoid FP receptor | PTGFR | P43088 | CHEMBL1987 | Family A G protein-coupled receptor | 0.066975016 | 31 / 1 | Butolic Acid |
| Lysine-specific demethylase 5C | KDM5C | P41229 | CHEMBL2163176 | Eraser | 0.066975016 | 25 / 1 | Butolic Acid |
| Protein farnesyltransferase | FNTA FNTB | P49354 P49356 | CHEMBL2094108 | Enzyme | 0.057956876 | 97 / 8 | Butolic Acid |
| Niemann-Pick C1-like protein 1 | NPC1L1 | Q9UHC9 | CHEMBL2027 | Other membrane protein | 0.057956876 | 0 / 6 | Butolic Acid |
| GABA A receptor alpha-2/beta-2/gamma-2 | GABRA2 GABRB2 GABRG2 | P47869 P47870 P18507 | CHEMBL2111413 | Ligand-gated ion channel | 0.057956876 | 0 / 2 | Butolic Acid |
| Androgen Receptor | AR | P10275 | CHEMBL1871 | Nuclear receptor | 0.057956876 | 5 / 33 | Butolic Acid |
| UDP-glucuronosyltransferase 2B7 | UGT2B7 | P16662 | CHEMBL4370 | Enzyme | 0.057956876 | 2 / 20 | Butolic Acid |
| DNA polymerase beta | POLB | P06746 | CHEMBL2392 | Enzyme | 0.057956876 | 1 / 5 | Butolic Acid |
| 11-beta-hydroxysteroid dehydrogenase 2 | HSD11B2 | P80365 | CHEMBL3746 | Enzyme | 0.048952898 | 9 / 3 | Butolic Acid |
| Carbonic anhydrase II | CA2 | P00918 | CHEMBL205 | Lyase | 0.048952898 | 66 / 5 | Butolic Acid |
| Carbonic anhydrase I | CA1 | P00915 | CHEMBL261 | Lyase | 0.048952898 | 60 / 7 | Butolic Acid |
| Inosine-5'-monophosphate dehydrogenase 2 | IMPDH2 | P12268 | CHEMBL2002 | Oxidoreductase | 0.048952898 | 57 / 0 | Butolic Acid |
| Glutathione S-transferase kappa 1 | GSTK1 | Q9Y2Q3 | CHEMBL4491 | Enzyme | 0.048952898 | 0 / 1 | Butolic Acid |
| Hydroxyacid oxidase 1 | HAO1 | Q9UJM8 | CHEMBL4229 | Enzyme | 0.048952898 | 4 / 1 | Butolic Acid |
| Protein-tyrosine phosphatase 1B | PTPN1 | P18031 | CHEMBL335 | Phosphatase | 0.048952898 | 183 / 27 | Butolic Acid |
| Progesterone receptor | PGR | P06401 | CHEMBL208 | Nuclear receptor | 0.048952898 | 18 / 0 | Butolic Acid |
| Leukotriene A4 hydrolase | LTA4H | P09960 | CHEMBL4618 | Protease | 0.048952898 | 75 / 1 | Butolic Acid |
| Prostanoid EP4 receptor | PTGER4 | P35408 | CHEMBL1836 | Family A G protein-coupled receptor | 0.048952898 | 280 / 0 | Butolic Acid |
| Lysine-specific demethylase 7A/7B | KDM7A | Q6ZMT4 | CHEMBL2163177 | Eraser | 0.048952898 | 2 / 0 | Butolic Acid |
| CDC45-related protein | CDC45 | O75419 | CHEMBL3040 | Other nuclear protein | 0.048952898 | 0 / 1 | Butolic Acid |
| Squalene synthetase (by homology) | FDFT1 | P37268 | CHEMBL3338 | Enzyme | 0.048952898 | 53 / 1 | Butolic Acid |
| Inosine-5'-monophosphate dehydrogenase 1 | IMPDH1 | P20839 | CHEMBL1822 | Oxidoreductase | 0.048952898 | 19 / 0 | Butolic Acid |
| HMG-CoA reductase | HMGCR | P04035 | CHEMBL402 | Oxidoreductase | 0.048952898 | 440 / 21 | Butolic Acid |
| Type-1 angiotensin II receptor (by homology) | AGTR1 | P30556 | CHEMBL227 | Family A G protein-coupled receptor | 0.048952898 | 29 / 0 | Butolic Acid |
| Thromboxane A2 receptor | TBXA2R | P21731 | CHEMBL2069 | Family A G protein-coupled receptor | 0.048952898 | 197 / 0 | Butolic Acid |
| Serine/threonine-protein kinase Chk1 | CHEK1 | O14757 | CHEMBL4630 | Kinase | 0.048952898 | 13 / 0 | Butolic Acid |
| Plasminogen | PLG | P00747 | CHEMBL1801 | Protease | 0.048952898 | 3 / 1 | Butolic Acid |
| Thyroid hormone receptor beta-1 | THRB | P10828 | CHEMBL1947 | Nuclear receptor | 0.048952898 | 162 / 0 | Butolic Acid |
| Matrix metalloproteinase 12 | MMP12 | P39900 | CHEMBL4393 | Protease | 0.048952898 | 45 / 0 | Butolic Acid |
| Matrix metalloproteinase 13 | MMP13 | P45452 | CHEMBL280 | Protease | 0.048952898 | 110 / 0 | Butolic Acid |
| Thyroid hormone receptor alpha | THRA | P10827 | CHEMBL1860 | Nuclear receptor | 0.048952898 | 149 / 0 | Butolic Acid |
| Adenosine A2a receptor | ADORA2A | P29274 | CHEMBL251 | Family A G protein-coupled receptor | 0.048952898 | 33 / 0 | Butolic Acid |
| Adenosine A2b receptor | ADORA2B | P29275 | CHEMBL255 | Family A G protein-coupled receptor | 0.048952898 | 28 / 0 | Butolic Acid |
| Prostanoid EP3 receptor | PTGER3 | P43115 | CHEMBL3710 | Family A G protein-coupled receptor | 0.048952898 | 48 / 0 | Butolic Acid |
| Peroxisome proliferator-activated receptor gamma | PPARG | P37231 | CHEMBL235 | Nuclear receptor | 0.048952898 | 191 / 8 | Butolic Acid |
| Neuronal acetylcholine receptor protein alpha-7 subunit (by homology) | CHRNA7 | P36544 | CHEMBL2492 | Ligand-gated ion channel | 0.048952898 | 0 / 2 | Butolic Acid |
| Hepatocyte nuclear factor 4-alpha | HNF4A | P41235 | CHEMBL5398 | Unclassified protein | 0.048952898 | 9 / 0 | Butolic Acid |
| G protein-coupled receptor kinase 6 | GRK6 | P43250 | CHEMBL6144 | Kinase | 0.048952898 | 24 / 0 | Butolic Acid |
| AMP deaminase 1 | AMPD1 | P23109 | CHEMBL2869 | Enzyme | 0.048952898 | 2 / 0 | Butolic Acid |
| Sphingosine 1-phosphate receptor Edg-8 | S1PR5 | Q9H228 | CHEMBL2274 | Family A G protein-coupled receptor | 0.048952898 | 43 / 0 | Butolic Acid |
| Cytochrome P450 2C9 | CYP2C9 | P11712 | CHEMBL3397 | Cytochrome P450 | 0.048952898 | 2 / 0 | Butolic Acid |
| MAP kinase ERK2 | MAPK1 | P28482 | CHEMBL4040 | Kinase | 0.048952898 | 7 / 0 | Butolic Acid |
| Estradiol 17-beta-dehydrogenase 2 | HSD17B2 | P37059 | CHEMBL2789 | Enzyme | 0.048952898 | 2 / 0 | Butolic Acid |
| Cytochrome P450 26B1 | CYP26B1 | Q9NR63 | CHEMBL3713687 | Cytochrome P450 | 0.048952898 | 4 / 0 | Butolic Acid |
| Cytochrome P450 26A1 | CYP26A1 | O43174 | CHEMBL5141 | Cytochrome P450 | 0.048952898 | 15 / 0 | Butolic Acid |
| Poly [ADP-ribose] polymerase-1 | PARP1 | P09874 | CHEMBL3105 | Enzyme | 0.048952898 | 11 / 0 | Butolic Acid |
| Histone deacetylase 1 | HDAC1 | Q13547 | CHEMBL325 | Eraser | 0.048952898 | 12 / 0 | Butolic Acid |
| Induced myeloid leukemia cell differentiation protein Mcl-1 | MCL1 | Q07820 | CHEMBL4361 | Other cytosolic protein | 0.048952898 | 40 / 0 | Butolic Acid |
| Epoxide hydratase | EPHX2 | P34913 | CHEMBL2409 | Protease | 0.048952898 | 54 / 0 | Butolic Acid |
| Vascular endothelial growth factor receptor 2 | KDR | P35968 | CHEMBL279 | Kinase | 0.048952898 | 67 / 0 | Butolic Acid |
| Metabotropic glutamate receptor 5 | GRM5 | P41594 | CHEMBL3227 | Family C G protein-coupled receptor | 0.048952898 | 0 / 4 | Butolic Acid |
| Sphingosine 1-phosphate receptor Edg-1 | S1PR1 | P21453 | CHEMBL4333 | Family A G protein-coupled receptor | 0.048952898 | 88 / 0 | Butolic Acid |
| Delta opioid receptor | OPRD1 | P41143 | CHEMBL236 | Family A G protein-coupled receptor | 0.048952898 | 100 / 0 | Butolic Acid |
| Dual specificity protein phosphatase 3 | DUSP3 | P51452 | CHEMBL2635 | Phosphatase | 0.048952898 | 28 / 0 | Butolic Acid |
| Methionyl-tRNA synthetase | MARS | P56192 | CHEMBL2870 | Enzyme | 0.048952898 | 1 / 0 | Butolic Acid |
| Indoleamine 2,3-dioxygenase | IDO1 | P14902 | CHEMBL4685 | Enzyme | 0.048952898 | 6 / 0 | Butolic Acid |
| Carbonic anhydrase XII | CA12 | O43570 | CHEMBL3242 | Lyase | 0.048952898 | 35 / 0 | Butolic Acid |
| Aldo-keto-reductase family 1 member C3 | AKR1C3 | P42330 | CHEMBL4681 | Enzyme | 0.048952898 | 75 / 0 | Butolic Acid |
| Carbonic anhydrase II | CA2 | P00918 | CHEMBL205 | Lyase | 0.729303188 | 33 / 13 | Caffeic acid |
| Arachidonate 5-lipoxygenase | ALOX5 | P09917 | CHEMBL215 | Oxidoreductase | 0.729303188 | 1 / 29 | Caffeic acid |
| Carbonic anhydrase VII | CA7 | P43166 | CHEMBL2326 | Lyase | 0.729303188 | 11 / 19 | Caffeic acid |
| Carbonic anhydrase I | CA1 | P00915 | CHEMBL261 | Lyase | 0.729303188 | 28 / 16 | Caffeic acid |
| Carbonic anhydrase VI | CA6 | P23280 | CHEMBL3025 | Lyase | 0.729303188 | 8 / 15 | Caffeic acid |
| Matrix metalloproteinase 9 | MMP9 | P14780 | CHEMBL321 | Protease | 0.729303188 | 2 / 35 | Caffeic acid |
| Carbonic anhydrase XII | CA12 | O43570 | CHEMBL3242 | Lyase | 0.729303188 | 19 / 14 | Caffeic acid |
| Matrix metalloproteinase 1 | MMP1 | P03956 | CHEMBL332 | Protease | 0.729303188 | 2 / 34 | Caffeic acid |
| Matrix metalloproteinase 2 | MMP2 | P08253 | CHEMBL333 | Protease | 0.729303188 | 2 / 36 | Caffeic acid |
| Protein-tyrosine phosphatase 1B | PTPN1 | P18031 | CHEMBL335 | Phosphatase | 0.729303188 | 18 / 2 | Caffeic acid |
| Carbonic anhydrase XIV | CA14 | Q9ULX7 | CHEMBL3510 | Lyase | 0.729303188 | 10 / 18 | Caffeic acid |
| Carbonic anhydrase IX | CA9 | Q16790 | CHEMBL3594 | Lyase | 0.729303188 | 15 / 18 | Caffeic acid |
| Carbonic anhydrase VB | CA5B | Q9Y2D0 | CHEMBL3969 | Lyase | 0.729303188 | 5 / 13 | Caffeic acid |
| Carbonic anhydrase VA | CA5A | P35218 | CHEMBL4789 | Lyase | 0.729303188 | 6 / 14 | Caffeic acid |
| Carbonic anhydrase III | CA3 | P07451 | CHEMBL2885 | Lyase | 0.212408696 | 4 / 3 | Caffeic acid |
| Aldose reductase | AKR1B1 | P15121 | CHEMBL1900 | Enzyme | 0.150923845 | 11 / 35 | Caffeic acid |
| Estrogen receptor beta | ESR2 | Q92731 | CHEMBL242 | Nuclear receptor | 0.150923845 | 1 / 10 | Caffeic acid |
| Carbonic anhydrase IV | CA4 | P22748 | CHEMBL3729 | Lyase | 0.150923845 | 3 / 8 | Caffeic acid |
| Aldo-keto reductase family 1 member B10 | AKR1B10 | O60218 | CHEMBL5983 | Enzyme | 0.080569968 | 0 / 18 | Caffeic acid |
| Hydroxycarboxylic acid receptor 2 | HCAR2 | Q8TDS4 | CHEMBL3785 | Family A G protein-coupled receptor | 0.080569968 | 0 / 1 | Caffeic acid |
| Carbonic anhydrase XIII | CA13 | Q8N1Q1 | CHEMBL3912 | Lyase | 0.080569968 | 4 / 10 | Caffeic acid |
| Macrophage migration inhibitory factor | MIF | P14174 | CHEMBL2085 | Enzyme | 0.080569968 | 1 / 1 | Caffeic acid |
| Quinone reductase 2 | NQO2 | P16083 | CHEMBL3959 | Enzyme | 0.071787163 | 0 / 17 | Caffeic acid |
| Toll-like receptor 4 (by homology) | TLR4 | O00206 | CHEMBL5255 | Toll-like and Il-1 receptors | 0.071787163 | 0 / 20 | Caffeic acid |
| Receptor protein-tyrosine kinase erbB-2 | ERBB2 | P04626 | CHEMBL1824 | Kinase | 0.071787163 | 0 / 3 | Caffeic acid |
| Estrogen receptor alpha | ESR1 | P03372 | CHEMBL206 | Nuclear receptor | 0.071787163 | 0 / 22 | Caffeic acid |
| Norepinephrine transporter | SLC6A2 | P23975 | CHEMBL222 | Electrochemical transporter | 0.071787163 | 0 / 3 | Caffeic acid |
| MAP kinase ERK2 | MAPK1 | P28482 | CHEMBL4040 | Kinase | 0.071787163 | 0 / 1 | Caffeic acid |
| Aldo-keto reductase family 1 member C4 | AKR1C4 | P17516 | CHEMBL4999 | Enzyme | 0.071787163 | 0 / 1 | Caffeic acid |
| Tyrosine-protein kinase SYK | SYK | P43405 | CHEMBL2599 | Kinase | 0.071787163 | 0 / 1 | Caffeic acid |
| Beta amyloid A4 protein | APP | P05067 | CHEMBL2487 | Membrane receptor | 0.071787163 | 0 / 44 | Caffeic acid |
| Tyrosine-protein kinase FYN | FYN | P06241 | CHEMBL1841 | Kinase | 0.071787163 | 3 / 2 | Caffeic acid |
| Tyrosine-protein kinase LCK | LCK | P06239 | CHEMBL258 | Kinase | 0.071787163 | 2 / 6 | Caffeic acid |
| Low affinity neurotrophin receptor p75NTR | NGFR | P08138 | CHEMBL4762 | Membrane receptor | 0.071787163 | 2 / 0 | Caffeic acid |
| Epidermal growth factor receptor erbB1 | EGFR | P00533 | CHEMBL203 | Kinase | 0.071787163 | 4 / 38 | Caffeic acid |
| Thiopurine S-methyltransferase | TPMT | P51580 | CHEMBL2500 | Enzyme | 0.071787163 | 4 / 0 | Caffeic acid |
| C-terminal-binding protein 2 | CTBP2 | P56545 | CHEMBL3797016 | Unclassified protein | 0.071787163 | 10 / 0 | Caffeic acid |
| Monoamine oxidase B | MAOB | P27338 | CHEMBL2039 | Oxidoreductase | 0.071787163 | 2 / 17 | Caffeic acid |
| PI3-kinase p110-beta subunit | PIK3CB | P42338 | CHEMBL3145 | Enzyme | 0.071787163 | 0 / 1 | Caffeic acid |
| Cytochrome P450 1A2 | CYP1A2 | P05177 | CHEMBL3356 | Cytochrome P450 | 0.071787163 | 0 / 5 | Caffeic acid |
| Cytochrome P450 2C9 | CYP2C9 | P11712 | CHEMBL3397 | Cytochrome P450 | 0.071787163 | 0 / 3 | Caffeic acid |
| Cytochrome P450 3A4 | CYP3A4 | P08684 | CHEMBL340 | Cytochrome P450 | 0.071787163 | 0 / 3 | Caffeic acid |
| Cytochrome P450 2C19 | CYP2C19 | P33261 | CHEMBL3622 | Cytochrome P450 | 0.071787163 | 0 / 3 | Caffeic acid |
| PI3-kinase p110-alpha subunit | PIK3CA | P42336 | CHEMBL4005 | Enzyme | 0.071787163 | 0 / 1 | Caffeic acid |
| Cyclooxygenase-1 | PTGS1 | P23219 | CHEMBL221 | Oxidoreductase | 0.071787163 | 1 / 20 | Caffeic acid |
| Leukocyte elastase | ELANE | P08246 | CHEMBL248 | Protease | 0.071787163 | 0 / 2 | Caffeic acid |
| Coagulation factor VII/tissue factor | F3 | P13726 | CHEMBL4081 | Surface antigen | 0.071787163 | 0 / 9 | Caffeic acid |
| 11-beta-hydroxysteroid dehydrogenase 1 | HSD11B1 | P28845 | CHEMBL4235 | Enzyme | 0.071787163 | 0 / 6 | Caffeic acid |
| Lysine-specific demethylase 4D-like | KDM4E | B2RXH2 | CHEMBL1293226 | Eraser | 0.071787163 | 4 / 0 | Caffeic acid |
| Lysine-specific demethylase 4A | KDM4A | O75164 | CHEMBL5896 | Eraser | 0.071787163 | 4 / 0 | Caffeic acid |
| Nuclear factor erythroid 2-related factor 2 | NFE2L2 | Q16236 | CHEMBL1075094 | Unclassified protein | 0.071787163 | 0 / 4 | Caffeic acid |
| Signal transducer and activator of transcription 3 | STAT3 | P40763 | CHEMBL4026 | Transcription factor | 0.071787163 | 0 / 1 | Caffeic acid |
| Carbonyl reductase [NADPH] 1 | CBR1 | P16152 | CHEMBL5586 | Enzyme | 0.237888614 | 2 / 3 | Cajanin |
| Estrogen receptor beta | ESR2 | Q92731 | CHEMBL242 | Nuclear receptor | 0.214178827 | 35 / 47 | Cajanin |
| Adenosine A1 receptor (by homology) | ADORA1 | P30542 | CHEMBL226 | Family A G protein-coupled receptor | 0.174646372 | 4 / 21 | Cajanin |
| Adenosine A2a receptor | ADORA2A | P29274 | CHEMBL251 | Family A G protein-coupled receptor | 0.174646372 | 3 / 14 | Cajanin |
| Aldehyde dehydrogenase | ALDH2 | P05091 | CHEMBL1935 | Oxidoreductase | 0.174646372 | 0 / 39 | Cajanin |
| Estrogen receptor alpha | ESR1 | P03372 | CHEMBL206 | Nuclear receptor | 0.143102156 | 20 / 57 | Cajanin |
| Estradiol 17-beta-dehydrogenase 2 | HSD17B2 | P37059 | CHEMBL2789 | Enzyme | 0.143102156 | 6 / 4 | Cajanin |
| Epidermal growth factor receptor erbB1 | EGFR | P00533 | CHEMBL203 | Kinase | 0.135202128 | 5 / 26 | Cajanin |
| Interleukin-2 | IL2 | P60568 | CHEMBL5880 | Secreted protein | 0.12730257 | 0 / 4 | Cajanin |
| Estradiol 17-beta-dehydrogenase 1 | HSD17B1 | P14061 | CHEMBL3181 | Enzyme | 0.119403562 | 6 / 5 | Cajanin |
| Thromboxane-A synthase | TBXAS1 | P24557 | CHEMBL1835 | Cytochrome P450 | 0.119403562 | 0 / 1 | Cajanin |
| Maltase-glucoamylase | MGAM | O43451 | CHEMBL2074 | Hydrolase | 0.119403562 | 0 / 1 | Cajanin |
| Serotonin 2a (5-HT2a) receptor | HTR2A | P28223 | CHEMBL224 | Family A G protein-coupled receptor | 0.119403562 | 0 / 2 | Cajanin |
| Serotonin 2c (5-HT2c) receptor | HTR2C | P28335 | CHEMBL225 | Family A G protein-coupled receptor | 0.119403562 | 0 / 2 | Cajanin |
| Estrogen-related receptor beta | ESRRB | O95718 | CHEMBL3751 | Nuclear receptor | 0.119403562 | 0 / 1 | Cajanin |
| Carbonic anhydrase XII | CA12 | O43570 | CHEMBL3242 | Lyase | 0.119403562 | 7 / 24 | Cajanin |
| Cyclooxygenase-1 | PTGS1 | P23219 | CHEMBL221 | Oxidoreductase | 0.119403562 | 0 / 1 | Cajanin |
| Norepinephrine transporter | SLC6A2 | P23975 | CHEMBL222 | Electrochemical transporter | 0.119403562 | 0 / 1 | Cajanin |
| Cytochrome P450 19A1 | CYP19A1 | P11511 | CHEMBL1978 | Cytochrome P450 | 0.111501865 | 4 / 24 | Cajanin |
| Carbonic anhydrase VII | CA7 | P43166 | CHEMBL2326 | Lyase | 0.111501865 | 5 / 18 | Cajanin |
| Carbonic anhydrase IV | CA4 | P22748 | CHEMBL3729 | Lyase | 0.111501865 | 5 / 13 | Cajanin |
| Macrophage migration inhibitory factor | MIF | P14174 | CHEMBL2085 | Enzyme | 0.111501865 | 0 / 5 | Cajanin |
| Estrogen-related receptor alpha | ESRRA | P11474 | CHEMBL3429 | Nuclear receptor | 0.111501865 | 1 / 2 | Cajanin |
| ATP-binding cassette sub-family G member 2 | ABCG2 | Q9UNQ0 | CHEMBL5393 | Primary active transporter | 0.111501865 | 5 / 40 | Cajanin |
| NADPH oxidase 4 | NOX4 | Q9NPH5 | CHEMBL1250375 | Enzyme | 0.111501865 | 7 / 7 | Cajanin |
| Monoamine oxidase B | MAOB | P27338 | CHEMBL2039 | Oxidoreductase | 0.111501865 | 0 / 102 | Cajanin |
| Arachidonate 5-lipoxygenase | ALOX5 | P09917 | CHEMBL215 | Oxidoreductase | 0.111501865 | 4 / 40 | Cajanin |
| 6-phosphofructo-2-kinase/fructose-2,6-bisphosphatase 3 | PFKFB3 | Q16875 | CHEMBL2331053 | Enzyme | 0.111501865 | 2 / 2 | Cajanin |
| Monoamine oxidase A | MAOA | P21397 | CHEMBL1951 | Oxidoreductase | 0.111501865 | 3 / 43 | Cajanin |
| P-glycoprotein 1 | ABCB1 | P08183 | CHEMBL4302 | Primary active transporter | 0.111501865 | 7 / 38 | Cajanin |
| Arachidonate 12-lipoxygenase | ALOX12 | P18054 | CHEMBL3687 | Enzyme | 0.111501865 | 7 / 12 | Cajanin |
| Receptor-type tyrosine-protein phosphatase S | PTPRS | Q13332 | CHEMBL2396508 | Phosphatase | 0.111501865 | 3 / 7 | Cajanin |
| Peroxisome proliferator-activated receptor alpha | PPARA | Q07869 | CHEMBL239 | Nuclear receptor | 0.111501865 | 0 / 1 | Cajanin |
| Carbonic anhydrase II | CA2 | P00918 | CHEMBL205 | Lyase | 0.111501865 | 8 / 25 | Cajanin |
| Carbonic anhydrase I | CA1 | P00915 | CHEMBL261 | Lyase | 0.111501865 | 4 / 22 | Cajanin |
| Acetylcholinesterase | ACHE | P22303 | CHEMBL220 | Hydrolase | 0.111501865 | 2 / 61 | Cajanin |
| Xanthine dehydrogenase | XDH | P47989 | CHEMBL1929 | Oxidoreductase | 0.111501865 | 11 / 19 | Cajanin |
| Toll-like receptor (TLR7/TLR9) | TLR9 | Q9NR96 | CHEMBL5804 | Toll-like and Il-1 receptors | 0.111501865 | 0 / 1 | Cajanin |
| Protein-tyrosine phosphatase 1B | PTPN1 | P18031 | CHEMBL335 | Phosphatase | 0.111501865 | 3 / 15 | Cajanin |
| Tyrosinase | TYR | P14679 | CHEMBL1973 | Oxidoreductase | 0.111501865 | 1 / 2 | Cajanin |
| Trypsin I | PRSS1 | P07477 | CHEMBL209 | Protease | 0.111501865 | 2 / 0 | Cajanin |
| Thrombin and coagulation factor X | F10 | P00742 | CHEMBL244 | Protease | 0.111501865 | 9 / 0 | Cajanin |
| Urokinase-type plasminogen activator | PLAU | P00749 | CHEMBL3286 | Protease | 0.111501865 | 9 / 0 | Cajanin |
| Calcium-activated potassium channel subunit alpha-1 | KCNMA1 | Q12791 | CHEMBL4304 | Voltage-gated ion channel | 0.111501865 | 5 / 0 | Cajanin |
| Serum paraoxonase/arylesterase 1 | PON1 | P27169 | CHEMBL3167 | Enzyme | 0.111501865 | 0 / 1 | Cajanin |
| Arachidonate 15-lipoxygenase | ALOX15 | P16050 | CHEMBL2903 | Enzyme | 0.111501865 | 5 / 9 | Cajanin |
| Aldose reductase | AKR1B1 | P15121 | CHEMBL1900 | Enzyme | 0.872151955 | 67 / 24 | Cladrin |
| Aldo-keto reductase family 1 member B10 | AKR1B10 | O60218 | CHEMBL5983 | Enzyme | 0.74994842 | 2 / 18 | Cladrin |
| Matrix metalloproteinase 13 | MMP13 | P45452 | CHEMBL280 | Protease | 0.256161866 | 1 / 3 | Cladrin |
| Matrix metalloproteinase 2 | MMP2 | P08253 | CHEMBL333 | Protease | 0.256161866 | 2 / 6 | Cladrin |
| Beta amyloid A4 protein | APP | P05067 | CHEMBL2487 | Membrane receptor | 0.130706653 | 1 / 16 | Cladrin |
| Matrix metalloproteinase 12 | MMP12 | P39900 | CHEMBL4393 | Protease | 0.122339194 | 1 / 5 | Cladrin |
| Glucose-6-phosphate translocase | SLC37A4 | O43826 | CHEMBL3217398 | Electrochemical transporter | 0.097239989 | 0 / 2 | Cladrin |
| Carbonic anhydrase II | CA2 | P00918 | CHEMBL205 | Lyase | 0.097239989 | 121 / 24 | Cladrin |
| Carbonic anhydrase I | CA1 | P00915 | CHEMBL261 | Lyase | 0.097239989 | 93 / 33 | Cladrin |
| Carbonic anhydrase XII | CA12 | O43570 | CHEMBL3242 | Lyase | 0.097239989 | 30 / 32 | Cladrin |
| Carbonic anhydrase IX | CA9 | Q16790 | CHEMBL3594 | Lyase | 0.097239989 | 30 / 36 | Cladrin |
| Liver glycogen phosphorylase | PYGL | P06737 | CHEMBL2568 | Enzyme | 0.097239989 | 27 / 1 | Cladrin |
| Protein kinase C delta (by homology) | PRKCD | Q05655 | CHEMBL2996 | Kinase | 0.097239989 | 0 / 51 | Cladrin |
| Protein kinase C alpha | PRKCA | P17252 | CHEMBL299 | Kinase | 0.097239989 | 0 / 243 | Cladrin |
| Sialidase 4 | NEU4 | Q8WWR8 | CHEMBL4174 | Enzyme | 0.097239989 | 7 / 0 | Cladrin |
| Beta-secretase 1 | BACE1 | P56817 | CHEMBL4822 | Protease | 0.097239989 | 0 / 3 | Cladrin |
| Caspase-3 | CASP3 | P42574 | CHEMBL2334 | Protease | 0.097239989 | 27 / 0 | Cladrin |
| Phosphodiesterase 4D | PDE4D | Q08499 | CHEMBL288 | Phosphodiesterase | 0.097239989 | 0 / 3 | Cladrin |
| Phosphodiesterase 9A | PDE9A | O76083 | CHEMBL3535 | Phosphodiesterase | 0.097239989 | 0 / 1 | Cladrin |
| Phosphodiesterase 1B | PDE1B | Q01064 | CHEMBL4425 | Phosphodiesterase | 0.097239989 | 0 / 1 | Cladrin |
| Vascular endothelial growth factor receptor 2 | KDR | P35968 | CHEMBL279 | Kinase | 0.097239989 | 5 / 0 | Cladrin |
| Endo-beta-N-acetylglucosaminidase | ENGASE | Q8NFI3 | CHEMBL5172 | Enzyme | 0.097239989 | 1 / 0 | Cladrin |
| Carbonic anhydrase VB | CA5B | Q9Y2D0 | CHEMBL3969 | Lyase | 0.097239989 | 0 / 14 | Cladrin |
| P-glycoprotein 1 | ABCB1 | P08183 | CHEMBL4302 | Primary active transporter | 0.097239989 | 0 / 72 | Cladrin |
| Sialidase 3 | NEU3 | Q9UQ49 | CHEMBL3046 | Enzyme | 0.097239989 | 7 / 0 | Cladrin |
| Sialidase 2 | NEU2 | Q9Y3R4 | CHEMBL3200 | Enzyme | 0.097239989 | 4 / 0 | Cladrin |
| Leukocyte elastase | ELANE | P08246 | CHEMBL248 | Protease | 0.097239989 | 1 / 2 | Cladrin |
| Bifunctional protein NCOAT | OGA | O60502 | CHEMBL5921 | Enzyme | 0.097239989 | 9 / 0 | Cladrin |
| Trehalase | TREH | O43280 | CHEMBL3087 | Enzyme | 0.097239989 | 1 / 0 | Cladrin |
| Caspase-6 | CASP6 | P55212 | CHEMBL3308 | Protease | 0.097239989 | 6 / 0 | Cladrin |
| Caspase-7 | CASP7 | P55210 | CHEMBL3468 | Protease | 0.097239989 | 7 / 0 | Cladrin |
| Caspase-8 | CASP8 | Q14790 | CHEMBL3776 | Protease | 0.097239989 | 7 / 0 | Cladrin |
| Caspase-1 | CASP1 | P29466 | CHEMBL4801 | Protease | 0.097239989 | 7 / 0 | Cladrin |
| Caspase-2 | CASP2 | P42575 | CHEMBL4884 | Protease | 0.097239989 | 3 / 0 | Cladrin |
| Endothelin-converting enzyme 1 | ECE1 | P42892 | CHEMBL4791 | Protease | 0.097239989 | 9 / 0 | Cladrin |
| Egl nine homolog 1 | EGLN1 | Q9GZT9 | CHEMBL5697 | Oxidoreductase | 0.097239989 | 10 / 0 | Cladrin |
| ADAMTS5 | ADAMTS5 | Q9UNA0 | CHEMBL2285 | Protease | 0.097239989 | 1 / 0 | Cladrin |
| Alpha-ketoglutarate-dependent dioxygenase FTO | FTO | Q9C0B1 | CHEMBL2331065 | Oxidoreductase | 0.097239989 | 1 / 0 | Cladrin |
| TNF-alpha | TNF | P01375 | CHEMBL1825 | Secreted protein | 0.578270841 | 1 / 3 | Cladrin |
| Interleukin-2 | IL2 | P60568 | CHEMBL5880 | Secreted protein | 0.578270841 | 3 / 5 | Cladrin |
| Aldehyde dehydrogenase | ALDH2 | P05091 | CHEMBL1935 | Oxidoreductase | 0.134155403 | 1 / 37 | Cladrin |
| Adenosine A1 receptor | ADORA1 | P30542 | CHEMBL226 | Family A G protein-coupled receptor | 0.100634432 | 240 / 3 | Cladrin |
| Carbonic anhydrase I | CA1 | P00915 | CHEMBL261 | Lyase | 0.100634432 | 60 / 14 | Cladrin |
| Carbonic anhydrase XII | CA12 | O43570 | CHEMBL3242 | Lyase | 0.100634432 | 49 / 19 | Cladrin |
| Carbonic anhydrase IX | CA9 | Q16790 | CHEMBL3594 | Lyase | 0.100634432 | 54 / 10 | Cladrin |
| Carbonic anhydrase II | CA2 | P00918 | CHEMBL205 | Lyase | 0.100634432 | 66 / 14 | Cladrin |
| Adenosine A3 receptor | ADORA3 | P0DMS8 | CHEMBL256 | Family A G protein-coupled receptor | 0.100634432 | 144 / 0 | Cladrin |
| Tyrosine-protein kinase ABL | ABL1 | P00519 | CHEMBL1862 | Kinase | 0.100634432 | 4 / 0 | Cladrin |
| Stem cell growth factor receptor | KIT | P10721 | CHEMBL1936 | Kinase | 0.100634432 | 3 / 0 | Cladrin |
| Platelet-derived growth factor receptor | PDGFRA PDGFRB | P16234 P09619 | CHEMBL2095189 | Kinase | 0.100634432 | 3 / 0 | Cladrin |
| Beta-glucocerebrosidase | GBA | P04062 | CHEMBL2179 | Enzyme | 0.100634432 | 48 / 0 | Cladrin |
| MAP kinase p38 alpha | MAPK14 | Q16539 | CHEMBL260 | Kinase | 0.100634432 | 7 / 0 | Cladrin |
| Tyrosine-protein kinase SRC | SRC | P12931 | CHEMBL267 | Kinase | 0.100634432 | 8 / 0 | Cladrin |
| Equilibrative nucleoside transporter 1 | SLC29A1 | Q99808 | CHEMBL1997 | Electrochemical transporter | 0.100634432 | 25 / 0 | Cladrin |
| Carbonic anhydrase XIII | CA13 | Q8N1Q1 | CHEMBL3912 | Lyase | 0.100634432 | 0 / 3 | Cladrin |
| Xanthine dehydrogenase | XDH | P47989 | CHEMBL1929 | Oxidoreductase | 0.100634432 | 0 / 9 | Cladrin |
| Beta-glucosidase | GBA2 | Q9HCG7 | CHEMBL3761 | Enzyme | 0.100634432 | 42 / 0 | Cladrin |
| Aldose reductase | AKR1B1 | P15121 | CHEMBL1900 | Enzyme | 0.100634432 | 2 / 16 | Cladrin |
| Poly [ADP-ribose] polymerase-1 | PARP1 | P09874 | CHEMBL3105 | Enzyme | 0.100634432 | 4 / 0 | Cladrin |
| Adenosine A2b receptor | ADORA2B | P29275 | CHEMBL255 | Family A G protein-coupled receptor | 0.100634432 | 8 / 0 | Cladrin |
| Aldose reductase | AKR1B1 | P15121 | CHEMBL1900 | Enzyme | 1 | 28 / 38 | Coumaric acid |
| Carbonic anhydrase II | CA2 | P00918 | CHEMBL205 | Lyase | 1 | 20 / 14 | Coumaric acid |
| Carbonic anhydrase VII | CA7 | P43166 | CHEMBL2326 | Lyase | 1 | 6 / 18 | Coumaric acid |
| Estrogen receptor beta | ESR2 | Q92731 | CHEMBL242 | Nuclear receptor | 1 | 1 / 12 | Coumaric acid |
| Carbonic anhydrase I | CA1 | P00915 | CHEMBL261 | Lyase | 1 | 17 / 18 | Coumaric acid |
| Carbonic anhydrase III | CA3 | P07451 | CHEMBL2885 | Lyase | 1 | 4 / 4 | Coumaric acid |
| Carbonic anhydrase VI | CA6 | P23280 | CHEMBL3025 | Lyase | 1 | 7 / 15 | Coumaric acid |
| Carbonic anhydrase XII | CA12 | O43570 | CHEMBL3242 | Lyase | 1 | 11 / 14 | Coumaric acid |
| Carbonic anhydrase XIV | CA14 | Q9ULX7 | CHEMBL3510 | Lyase | 1 | 7 / 18 | Coumaric acid |
| Carbonic anhydrase IX | CA9 | Q16790 | CHEMBL3594 | Lyase | 1 | 10 / 19 | Coumaric acid |
| Carbonic anhydrase IV | CA4 | P22748 | CHEMBL3729 | Lyase | 1 | 2 / 7 | Coumaric acid |
| Carbonic anhydrase VB | CA5B | Q9Y2D0 | CHEMBL3969 | Lyase | 1 | 5 / 14 | Coumaric acid |
| Carbonic anhydrase VA | CA5A | P35218 | CHEMBL4789 | Lyase | 1 | 6 / 15 | Coumaric acid |
| Macrophage migration inhibitory factor | MIF | P14174 | CHEMBL2085 | Enzyme | 0.249992358 | 1 / 1 | Coumaric acid |
| Arachidonate 5-lipoxygenase | ALOX5 | P09917 | CHEMBL215 | Oxidoreductase | 0.208479015 | 1 / 21 | Coumaric acid |
| Matrix metalloproteinase 9 | MMP9 | P14780 | CHEMBL321 | Protease | 0.208479015 | 2 / 19 | Coumaric acid |
| Matrix metalloproteinase 1 | MMP1 | P03956 | CHEMBL332 | Protease | 0.208479015 | 2 / 18 | Coumaric acid |
| Matrix metalloproteinase 2 | MMP2 | P08253 | CHEMBL333 | Protease | 0.208479015 | 2 / 20 | Coumaric acid |
| Protein-tyrosine phosphatase 1B | PTPN1 | P18031 | CHEMBL335 | Phosphatase | 0.208479015 | 13 / 2 | Coumaric acid |
| Aldo-keto reductase family 1 member B10 | AKR1B10 | O60218 | CHEMBL5983 | Enzyme | 0.158397607 | 0 / 18 | Coumaric acid |
| Toll-like receptor 4 (by homology) | TLR4 | O00206 | CHEMBL5255 | Toll-like and Il-1 receptors | 0.133391038 | 0 / 22 | Coumaric acid |
| Carbonic anhydrase XIII (by homology) | CA13 | Q8N1Q1 | CHEMBL3912 | Lyase | 0.133391038 | 2 / 11 | Coumaric acid |
| Lysine-specific demethylase 4D-like | KDM4E | B2RXH2 | CHEMBL1293226 | Eraser | 0.133391038 | 3 / 0 | Coumaric acid |
| Lysine-specific demethylase 4A | KDM4A | O75164 | CHEMBL5896 | Eraser | 0.133391038 | 4 / 0 | Coumaric acid |
| Lysine-specific demethylase 3A | KDM3A | Q9Y4C1 | CHEMBL1938209 | Eraser | 0.12507596 | 2 / 0 | Coumaric acid |
| Lysine-specific demethylase 6B | KDM6B | O15054 | CHEMBL1938211 | Eraser | 0.12507596 | 2 / 0 | Coumaric acid |
| Alpha-ketoglutarate-dependent dioxygenase FTO | FTO | Q9C0B1 | CHEMBL2331065 | Oxidoreductase | 0.12507596 | 2 / 0 | Coumaric acid |
| Lysine-specific demethylase 4C | KDM4C | Q9H3R0 | CHEMBL6175 | Eraser | 0.12507596 | 3 / 0 | Coumaric acid |
| Coagulation factor VII/tissue factor | F3 | P13726 | CHEMBL4081 | Surface antigen | 0.12507596 | 0 / 10 | Coumaric acid |
| 11-beta-hydroxysteroid dehydrogenase 1 | HSD11B1 | P28845 | CHEMBL4235 | Enzyme | 0.12507596 | 0 / 6 | Coumaric acid |
| Hydroxycarboxylic acid receptor 2 | HCAR2 | Q8TDS4 | CHEMBL3785 | Family A G protein-coupled receptor | 0.12507596 | 7 / 1 | Coumaric acid |
| Thiopurine S-methyltransferase | TPMT | P51580 | CHEMBL2500 | Enzyme | 0.12507596 | 4 / 0 | Coumaric acid |
| D-amino-acid oxidase | DAO | P14920 | CHEMBL5485 | Enzyme | 0.116739032 | 5 / 0 | Coumaric acid |
| Transient receptor potential cation channel subfamily A member 1 | TRPA1 | O75762 | CHEMBL6007 | Voltage-gated ion channel | 0.116739032 | 0 / 1 | Coumaric acid |
| Estrogen receptor alpha | ESR1 | P03372 | CHEMBL206 | Nuclear receptor | 0.116739032 | 0 / 25 | Coumaric acid |
| Progesterone receptor | PGR | P06401 | CHEMBL208 | Nuclear receptor | 0.116739032 | 0 / 2 | Coumaric acid |
| Tyrosine-protein kinase FYN | FYN | P06241 | CHEMBL1841 | Kinase | 0.116739032 | 2 / 2 | Coumaric acid |
| Tyrosine-protein kinase LCK | LCK | P06239 | CHEMBL258 | Kinase | 0.116739032 | 2 / 6 | Coumaric acid |
| Low affinity neurotrophin receptor p75NTR | NGFR | P08138 | CHEMBL4762 | Membrane receptor | 0.116739032 | 1 / 0 | Coumaric acid |
| Thrombin | F2 | P00734 | CHEMBL204 | Protease | 0.768469201 | 1 / 3 | Cynidin |
| LXR-alpha | NR1H3 | Q13133 | CHEMBL2808 | Nuclear receptor | 0.768469201 | 1 / 1 | Cynidin |
| Glyoxalase I | GLO1 | Q04760 | CHEMBL2424 | Enzyme | 0.320218721 | 0 / 4 | Cynidin |
| Lymphocyte differentiation antigen CD38 | CD38 | P28907 | CHEMBL4660 | Enzyme | 0.122581769 | 0 / 3 | Cynidin |
| NADPH oxidase 4 | NOX4 | Q9NPH5 | CHEMBL1250375 | Enzyme | 0.106099949 | 0 / 8 | Cynidin |
| Microtubule-associated protein tau | MAPT | P10636 | CHEMBL1293224 | Unclassified protein | 0.106099949 | 0 / 1 | Cynidin |
| Lysine-specific demethylase 4D-like | KDM4E | B2RXH2 | CHEMBL1293226 | Eraser | 0.106099949 | 0 / 2 | Cynidin |
| G-protein coupled receptor 35 | GPR35 | Q9HC97 | CHEMBL1293267 | Family A G protein-coupled receptor | 0.106099949 | 0 / 2 | Cynidin |
| Vasopressin V2 receptor | AVPR2 | P30518 | CHEMBL1790 | Family A G protein-coupled receptor | 0.106099949 | 0 / 1 | Cynidin |
| DNA topoisomerase II alpha | TOP2A | P11388 | CHEMBL1806 | Isomerase | 0.106099949 | 0 / 1 | Cynidin |
| Aldose reductase | AKR1B1 | P15121 | CHEMBL1900 | Enzyme | 0.106099949 | 0 / 62 | Cynidin |
| Xanthine dehydrogenase | XDH | P47989 | CHEMBL1929 | Oxidoreductase | 0.106099949 | 0 / 20 | Cynidin |
| Monoamine oxidase A | MAOA | P21397 | CHEMBL1951 | Oxidoreductase | 0.106099949 | 0 / 3 | Cynidin |
| Insulin-like growth factor I receptor | IGF1R | P08069 | CHEMBL1957 | Kinase | 0.106099949 | 0 / 3 | Cynidin |
| Tyrosine-protein kinase receptor FLT3 | FLT3 | P36888 | CHEMBL1974 | Kinase | 0.106099949 | 0 / 6 | Cynidin |
| Cytochrome P450 19A1 | CYP19A1 | P11511 | CHEMBL1978 | Cytochrome P450 | 0.106099949 | 0 / 12 | Cynidin |
| Insulin receptor | INSR | P06213 | CHEMBL1981 | Kinase | 0.106099949 | 0 / 1 | Cynidin |
| Epidermal growth factor receptor erbB1 | EGFR | P00533 | CHEMBL203 | Kinase | 0.106099949 | 0 / 13 | Cynidin |
| Carbonic anhydrase II | CA2 | P00918 | CHEMBL205 | Lyase | 0.106099949 | 0 / 11 | Cynidin |
| Serine/threonine-protein kinase PIM1 | PIM1 | P11309 | CHEMBL2147 | Kinase | 0.106099949 | 0 / 6 | Cynidin |
| Arachidonate 5-lipoxygenase | ALOX5 | P09917 | CHEMBL215 | Oxidoreductase | 0.106099949 | 0 / 48 | Cynidin |
| Serine/threonine-protein kinase Aurora-B | AURKB | Q96GD4 | CHEMBL2185 | Kinase | 0.106099949 | 0 / 4 | Cynidin |
| Dopamine D4 receptor | DRD4 | P21917 | CHEMBL219 | Family A G protein-coupled receptor | 0.106099949 | 0 / 1 | Cynidin |
| Acetylcholinesterase | ACHE | P22303 | CHEMBL220 | Hydrolase | 0.106099949 | 0 / 22 | Cynidin |
| Adenosine A1 receptor (by homology) | ADORA1 | P30542 | CHEMBL226 | Family A G protein-coupled receptor | 0.106099949 | 0 / 20 | Cynidin |
| Carbonic anhydrase VII | CA7 | P43166 | CHEMBL2326 | Lyase | 0.106099949 | 0 / 8 | Cynidin |
| Myosin light chain kinase, smooth muscle | MYLK | Q15746 | CHEMBL2428 | Kinase | 0.106099949 | 0 / 1 | Cynidin |
| Myeloperoxidase | MPO | P05164 | CHEMBL2439 | Enzyme | 0.106099949 | 0 / 1 | Cynidin |
| PI3-kinase p85-alpha subunit | PIK3R1 | P27986 | CHEMBL2506 | Enzyme | 0.106099949 | 0 / 1 | Cynidin |
| Death-associated protein kinase 1 | DAPK1 | P53355 | CHEMBL2558 | Kinase | 0.106099949 | 0 / 2 | Cynidin |
| Liver glycogen phosphorylase | PYGL | P06737 | CHEMBL2568 | Enzyme | 0.106099949 | 0 / 1 | Cynidin |
| Tyrosine-protein kinase SYK | SYK | P43405 | CHEMBL2599 | Kinase | 0.106099949 | 0 / 3 | Cynidin |
| Carbonic anhydrase I | CA1 | P00915 | CHEMBL261 | Lyase | 0.106099949 | 0 / 6 | Cynidin |
| Glycogen synthase kinase-3 beta | GSK3B | P49841 | CHEMBL262 | Kinase | 0.106099949 | 0 / 6 | Cynidin |
| Tyrosine-protein kinase SRC | SRC | P12931 | CHEMBL267 | Kinase | 0.106099949 | 0 / 9 | Cynidin |
| Focal adhesion kinase 1 | PTK2 | Q05397 | CHEMBL2695 | Kinase | 0.106099949 | 0 / 2 | Cynidin |
| Estradiol 17-beta-dehydrogenase 2 | HSD17B2 | P37059 | CHEMBL2789 | Enzyme | 0.106099949 | 0 / 2 | Cynidin |
| Vascular endothelial growth factor receptor 2 | KDR | P35968 | CHEMBL279 | Kinase | 0.106099949 | 0 / 3 | Cynidin |
| Matrix metalloproteinase 13 | MMP13 | P45452 | CHEMBL280 | Protease | 0.106099949 | 0 / 1 | Cynidin |
| Matrix metalloproteinase 3 | MMP3 | P08254 | CHEMBL283 | Protease | 0.106099949 | 0 / 1 | Cynidin |
| Carbonic anhydrase III | CA3 | P07451 | CHEMBL2885 | Lyase | 0.106099949 | 0 / 1 | Cynidin |
| Arachidonate 15-lipoxygenase | ALOX15 | P16050 | CHEMBL2903 | Enzyme | 0.106099949 | 0 / 5 | Cynidin |
| Multidrug resistance-associated protein 1 | ABCC1 | P33527 | CHEMBL3004 | Primary active transporter | 0.106099949 | 0 / 11 | Cynidin |
| Serine/threonine-protein kinase PLK1 | PLK1 | P53350 | CHEMBL3024 | Kinase | 0.106099949 | 0 / 3 | Cynidin |
| Carbonic anhydrase VI | CA6 | P23280 | CHEMBL3025 | Lyase | 0.106099949 | 0 / 1 | Cynidin |
| Cyclin-dependent kinase 1 | CDK1 | P06493 | CHEMBL308 | Kinase | 0.106099949 | 0 / 8 | Cynidin |
| Matrix metalloproteinase 9 | MMP9 | P14780 | CHEMBL321 | Protease | 0.106099949 | 0 / 2 | Cynidin |
| Carbonic anhydrase XII | CA12 | O43570 | CHEMBL3242 | Lyase | 0.106099949 | 0 / 11 | Cynidin |
| PI3-kinase p110-gamma subunit | PIK3CG | P48736 | CHEMBL3267 | Enzyme | 0.106099949 | 0 / 1 | Cynidin |
| Matrix metalloproteinase 2 | MMP2 | P08253 | CHEMBL333 | Protease | 0.106099949 | 0 / 2 | Cynidin |
| Protein kinase N1 | PKN1 | Q16512 | CHEMBL3384 | Kinase | 0.106099949 | 0 / 2 | Cynidin |
| Carbonic anhydrase XIV | CA14 | Q9ULX7 | CHEMBL3510 | Lyase | 0.106099949 | 0 / 1 | Cynidin |
| Carbonic anhydrase IX | CA9 | Q16790 | CHEMBL3594 | Lyase | 0.106099949 | 0 / 6 | Cynidin |
| Casein kinase II alpha | CSNK2A1 | P68400 | CHEMBL3629 | Kinase | 0.106099949 | 0 / 2 | Cynidin |
| Arachidonate 12-lipoxygenase | ALOX12 | P18054 | CHEMBL3687 | Enzyme | 0.106099949 | 0 / 6 | Cynidin |
| Hepatocyte growth factor receptor | MET | P08581 | CHEMBL3717 | Kinase | 0.106099949 | 0 / 4 | Cynidin |
| Carbonic anhydrase IV | CA4 | P22748 | CHEMBL3729 | Lyase | 0.106099949 | 0 / 7 | Cynidin |
| Serine/threonine-protein kinase NEK2 | NEK2 | P51955 | CHEMBL3835 | Kinase | 0.106099949 | 0 / 2 | Cynidin |
| Interleukin-8 receptor A | CXCR1 | P25024 | CHEMBL4029 | Family A G protein-coupled receptor | 0.106099949 | 0 / 1 | Cynidin |
| CaM kinase II beta | CAMK2B | Q13554 | CHEMBL4121 | Kinase | 0.106099949 | 0 / 1 | Cynidin |
| ALK tyrosine kinase receptor | ALK | Q9UM73 | CHEMBL4247 | Kinase | 0.106099949 | 0 / 3 | Cynidin |
| Serine/threonine-protein kinase AKT | AKT1 | P31749 | CHEMBL4282 | Kinase | 0.106099949 | 0 / 4 | Cynidin |
| P-glycoprotein 1 | ABCB1 | P08183 | CHEMBL4302 | Primary active transporter | 0.106099949 | 0 / 43 | Cynidin |
| Serine/threonine-protein kinase NEK6 | NEK6 | Q9HC98 | CHEMBL4309 | Kinase | 0.106099949 | 0 / 2 | Cynidin |
| Phospholipase A2 group 1B | PLA2G1B | P04054 | CHEMBL4426 | Enzyme | 0.106099949 | 0 / 1 | Cynidin |
| Carbonic anhydrase VA | CA5A | P35218 | CHEMBL4789 | Lyase | 0.106099949 | 0 / 1 | Cynidin |
| Beta-secretase 1 | BACE1 | P56817 | CHEMBL4822 | Protease | 0.106099949 | 0 / 12 | Cynidin |
| Cytochrome P450 1B1 | CYP1B1 | Q16678 | CHEMBL4878 | Cytochrome P450 | 0.106099949 | 0 / 43 | Cynidin |
| Tyrosine-protein kinase receptor UFO | AXL | P30530 | CHEMBL4895 | Kinase | 0.106099949 | 0 / 3 | Cynidin |
| ATP-binding cassette sub-family G member 2 | ABCG2 | Q9UNQ0 | CHEMBL5393 | Primary active transporter | 0.106099949 | 0 / 44 | Cynidin |
| DNA-(apurinic or apyrimidinic site) lyase | APEX1 | P27695 | CHEMBL5619 | Enzyme | 0.106099949 | 0 / 1 | Cynidin |
| NUAK family SNF1-like kinase 1 | NUAK1 | O60285 | CHEMBL5784 | Kinase | 0.106099949 | 0 / 2 | Cynidin |
| Aldo-keto reductase family 1 member C2 (by homology) | AKR1C2 | P52895 | CHEMBL5847 | Enzyme | 0.106099949 | 0 / 1 | Cynidin |
| Aldo-keto reductase family 1 member C1 (by homology) | AKR1C1 | Q04828 | CHEMBL5905 | Enzyme | 0.106099949 | 0 / 1 | Cynidin |
| Aldo-keto-reductase family 1 member C3 (by homology) | AKR1C3 | P42330 | CHEMBL4681 | Enzyme | 0.106099949 | 0 / 1 | Cynidin |
| Aldo-keto reductase family 1 member C4 (by homology) | AKR1C4 | P17516 | CHEMBL4999 | Enzyme | 0.106099949 | 0 / 1 | Cynidin |
| Carbonic anhydrase XIII (by homology) | CA13 | Q8N1Q1 | CHEMBL3912 | Lyase | 0.106099949 | 0 / 1 | Cynidin |
| Adenosine A2a receptor (by homology) | ADORA2A | P29274 | CHEMBL251 | Family A G protein-coupled receptor | 0.106099949 | 0 / 10 | Cynidin |
| Aldehyde reductase (by homology) | AKR1A1 | P14550 | CHEMBL2246 | Enzyme | 0.106099949 | 0 / 1 | Cynidin |
| NAD-dependent deacetylase sirtuin 1 | SIRT1 | Q96EB6 | CHEMBL4506 | Eraser | 0.106099949 | 0 / 1 | Cynidin |
| Cyclin-dependent kinase 5/CDK5 activator 1 | CDK5R1 CDK5 | Q15078 Q00535 | CHEMBL1907600 | Kinase | 0.106099949 | 0 / 5 | Cynidin |
| Cyclin-dependent kinase 1/cyclin B | CCNB3 CDK1 CCNB1 CCNB2 | Q8WWL7 P06493 P14635 O95067 | CHEMBL2094127 | Other cytosolic protein | 0.106099949 | 0 / 4 | Cynidin |
| Cyclin-dependent kinase 6 | CDK6 | Q00534 | CHEMBL2508 | Kinase | 0.106099949 | 0 / 3 | Cynidin |
| Cyclin-dependent kinase 2 | CDK2 | P24941 | CHEMBL301 | Kinase | 0.106099949 | 0 / 10 | Cynidin |
| Arginase-1 (by homology) | ARG1 | P05089 | CHEMBL1075097 | Enzyme | 0.106099949 | 0 / 2 | Cynidin |
| Beta-galactoside alpha-2,6-sialyltransferase 1 | ST6GAL1 | P15907 | CHEMBL3596075 | Transferase | 0.097874534 | 0 / 2 | Cynidin |
| Cyclooxygenase-1 | PTGS1 | P23219 | CHEMBL221 | Oxidoreductase | 0.097874534 | 0 / 3 | Cynidin |
| Phosphodiesterase 4B | PDE4B | Q07343 | CHEMBL275 | Phosphodiesterase | 0.097874534 | 0 / 1 | Cynidin |
| Phosphodiesterase 4D | PDE4D | Q08499 | CHEMBL288 | Phosphodiesterase | 0.097874534 | 0 / 12 | Cynidin |
| Tyrosinase | TYR | P14679 | CHEMBL1973 | Oxidoreductase | 0.097874534 | 0 / 2 | Cynidin |
| Estradiol 17-beta-dehydrogenase 1 | HSD17B1 | P14061 | CHEMBL3181 | Enzyme | 0.097874534 | 0 / 3 | Cynidin |
| Aryl hydrocarbon receptor | AHR | P35869 | CHEMBL3201 | Transcription factor | 0.097874534 | 0 / 1 | Cynidin |
| Estrogen-related receptor alpha | ESRRA | P11474 | CHEMBL3429 | Nuclear receptor | 0.097874534 | 0 / 1 | Cynidin |
| Adenosine A3 receptor | ADORA3 | P0DMS8 | CHEMBL256 | Family A G protein-coupled receptor | 0.097874534 | 0 / 16 | Cynidin |
| Receptor-type tyrosine-protein phosphatase S | PTPRS | Q13332 | CHEMBL2396508 | Phosphatase | 0.097874534 | 0 / 8 | Cynidin |
| Telomerase reverse transcriptase | TERT | O14746 | CHEMBL2916 | Enzyme | 0.097874534 | 0 / 16 | Cynidin |
| Butyrylcholinesterase | BCHE | P06276 | CHEMBL1914 | Hydrolase | 0.097874534 | 0 / 7 | Cynidin |
| Beta amyloid A4 protein | APP | P05067 | CHEMBL2487 | Membrane receptor | 0.097874534 | 0 / 10 | Cynidin |
| Poly [ADP-ribose] polymerase-1 | PARP1 | P09874 | CHEMBL3105 | Enzyme | 0.097874534 | 0 / 9 | Cynidin |
| Transthyretin | TTR | P02766 | CHEMBL3194 | Secreted protein | 0.097874534 | 0 / 2 | Cynidin |
| Aldehyde dehydrogenase | ALDH2 | P05091 | CHEMBL1935 | Oxidoreductase | 1 | 1 / 51 | Daidzein |
| Estrogen receptor alpha | ESR1 | P03372 | CHEMBL206 | Nuclear receptor | 1 | 76 / 79 | Daidzein |
| Carbonic anhydrase VII | CA7 | P43166 | CHEMBL2326 | Lyase | 1 | 8 / 12 | Daidzein |
| Estrogen receptor beta | ESR2 | Q92731 | CHEMBL242 | Nuclear receptor | 1 | 92 / 69 | Daidzein |
| Carbonic anhydrase XII | CA12 | O43570 | CHEMBL3242 | Lyase | 1 | 8 / 18 | Daidzein |
| Carbonic anhydrase IV | CA4 | P22748 | CHEMBL3729 | Lyase | 1 | 7 / 9 | Daidzein |
| Thromboxane-A synthase | TBXAS1 | P24557 | CHEMBL1835 | Cytochrome P450 | 0.536298049 | 1 / 2 | Daidzein |
| Monoamine oxidase A | MAOA | P21397 | CHEMBL1951 | Oxidoreductase | 0.536298049 | 6 / 37 | Daidzein |
| Epidermal growth factor receptor erbB1 | EGFR | P00533 | CHEMBL203 | Kinase | 0.536298049 | 6 / 6 | Daidzein |
| Maltase-glucoamylase | MGAM | O43451 | CHEMBL2074 | Hydrolase | 0.536298049 | 1 / 1 | Daidzein |
| Serotonin 2a (5-HT2a) receptor | HTR2A | P28223 | CHEMBL224 | Family A G protein-coupled receptor | 0.536298049 | 1 / 2 | Daidzein |
| Serotonin 2c (5-HT2c) receptor | HTR2C | P28335 | CHEMBL225 | Family A G protein-coupled receptor | 0.536298049 | 1 / 3 | Daidzein |
| Adenosine A1 receptor (by homology) | ADORA1 | P30542 | CHEMBL226 | Family A G protein-coupled receptor | 0.536298049 | 6 / 18 | Daidzein |
| Adenosine A2a receptor | ADORA2A | P29274 | CHEMBL251 | Family A G protein-coupled receptor | 0.536298049 | 5 / 15 | Daidzein |
| Estradiol 17-beta-dehydrogenase 1 | HSD17B1 | P14061 | CHEMBL3181 | Enzyme | 0.536298049 | 10 / 5 | Daidzein |
| Estrogen-related receptor alpha | ESRRA | P11474 | CHEMBL3429 | Nuclear receptor | 0.536298049 | 2 / 2 | Daidzein |
| Estrogen-related receptor beta | ESRRB | O95718 | CHEMBL3751 | Nuclear receptor | 0.536298049 | 1 / 1 | Daidzein |
| ATP-binding cassette sub-family G member 2 | ABCG2 | Q9UNQ0 | CHEMBL5393 | Primary active transporter | 0.536298049 | 6 / 25 | Daidzein |
| Cytochrome P450 19A1 | CYP19A1 | P11511 | CHEMBL1978 | Cytochrome P450 | 0.387023621 | 6 / 27 | Daidzein |
| Arachidonate 12-lipoxygenase | ALOX12 | P18054 | CHEMBL3687 | Enzyme | 0.316300346 | 9 / 11 | Daidzein |
| Tyrosinase (by homology) | TYR | P14679 | CHEMBL1973 | Oxidoreductase | 0.292767344 | 2 / 2 | Daidzein |
| Macrophage migration inhibitory factor | MIF | P14174 | CHEMBL2085 | Enzyme | 0.292767344 | 1 / 8 | Daidzein |
| Xanthine dehydrogenase | XDH | P47989 | CHEMBL1929 | Oxidoreductase | 0.237759865 | 11 / 20 | Daidzein |
| 6-phosphofructo-2-kinase/fructose-2,6-bisphosphatase 3 | PFKFB3 | Q16875 | CHEMBL2331053 | Enzyme | 0.206337197 | 1 / 2 | Daidzein |
| Interleukin-2 | IL2 | P60568 | CHEMBL5880 | Secreted protein | 0.198443988 | 0 / 4 | Daidzein |
| Carbonyl reductase [NADPH] 1 | CBR1 | P16152 | CHEMBL5586 | Enzyme | 0.182723414 | 2 / 2 | Daidzein |
| Estradiol 17-beta-dehydrogenase 2 | HSD17B2 | P37059 | CHEMBL2789 | Enzyme | 0.167041662 | 10 / 4 | Daidzein |
| P-glycoprotein 1 | ABCB1 | P08183 | CHEMBL4302 | Primary active transporter | 0.151315357 | 12 / 37 | Daidzein |
| Receptor-type tyrosine-protein phosphatase S | PTPRS | Q13332 | CHEMBL2396508 | Phosphatase | 0.143473412 | 3 / 7 | Daidzein |
| Arachidonate 15-lipoxygenase | ALOX15 | P16050 | CHEMBL2903 | Enzyme | 0.143473412 | 6 / 8 | Daidzein |
| Cyclooxygenase-1 | PTGS1 | P23219 | CHEMBL221 | Oxidoreductase | 0.127750341 | 0 / 3 | Daidzein |
| Norepinephrine transporter | SLC6A2 | P23975 | CHEMBL222 | Electrochemical transporter | 0.127750341 | 0 / 1 | Daidzein |
| NADPH oxidase 4 | NOX4 | Q9NPH5 | CHEMBL1250375 | Enzyme | 0.127750341 | 5 / 6 | Daidzein |
| Monoamine oxidase B | MAOB | P27338 | CHEMBL2039 | Oxidoreductase | 0.119895127 | 0 / 83 | Daidzein |
| Serum paraoxonase/arylesterase 1 | PON1 | P27169 | CHEMBL3167 | Enzyme | 0.119895127 | 0 / 1 | Daidzein |
| Carbonic anhydrase II | CA2 | P00918 | CHEMBL205 | Lyase | 0.112041901 | 6 / 22 | Daidzein |
| Carbonic anhydrase I | CA1 | P00915 | CHEMBL261 | Lyase | 0.112041901 | 2 / 20 | Daidzein |
| Steryl-sulfatase | STS | P08842 | CHEMBL3559 | Enzyme | 0.112041901 | 0 / 6 | Daidzein |
| Acetylcholinesterase | ACHE | P22303 | CHEMBL220 | Hydrolase | 0.112041901 | 4 / 33 | Daidzein |
| Toll-like receptor (TLR7/TLR9) | TLR9 | Q9NR96 | CHEMBL5804 | Toll-like and Il-1 receptors | 0.112041901 | 0 / 1 | Daidzein |
| Arachidonate 5-lipoxygenase | ALOX5 | P09917 | CHEMBL215 | Oxidoreductase | 0.112041901 | 4 / 11 | Daidzein |
| Peroxisome proliferator-activated receptor alpha | PPARA | Q07869 | CHEMBL239 | Nuclear receptor | 0.112041901 | 1 / 1 | Daidzein |
| Tankyrase-2 | TNKS2 | Q9H2K2 | CHEMBL6154 | Enzyme | 0.112041901 | 4 / 9 | Daidzein |
| Tankyrase-1 | TNKS | O95271 | CHEMBL6164 | Enzyme | 0.112041901 | 4 / 13 | Daidzein |
| Protein-tyrosine phosphatase 1B | PTPN1 | P18031 | CHEMBL335 | Phosphatase | 0.112041901 | 4 / 22 | Daidzein |
| Carbonic anhydrase IX | CA9 | Q16790 | CHEMBL3594 | Lyase | 0.112041901 | 2 / 15 | Daidzein |
| Tissue-type plasminogen activator | PLAT | P00750 | CHEMBL1873 | Protease | 0.112041901 | 8 / 0 | Daidzein |
| Thrombin and coagulation factor X | F10 | P00742 | CHEMBL244 | Protease | 0.112041901 | 9 / 0 | Daidzein |
| Urokinase-type plasminogen activator | PLAU | P00749 | CHEMBL3286 | Protease | 0.112041901 | 12 / 0 | Daidzein |
| Carbonic anhydrase VB | CA5B | Q9Y2D0 | CHEMBL3969 | Lyase | 0.112041901 | 0 / 2 | Daidzein |
| Induced myeloid leukemia cell differentiation protein Mcl-1 | MCL1 | Q07820 | CHEMBL4361 | Other cytosolic protein | 0.112041901 | 4 / 4 | Daidzein |
| Peroxisome proliferator-activated receptor gamma | PPARG | P37231 | CHEMBL235 | Nuclear receptor | 0.112041901 | 0 / 2 | Daidzein |
| Aldose reductase | AKR1B1 | P15121 | CHEMBL1900 | Enzyme | 0.710440816 | 25 / 68 | Dimethyl Quercetin |
| Xanthine dehydrogenase | XDH | P47989 | CHEMBL1929 | Oxidoreductase | 0.346957709 | 14 / 21 | Dimethyl Quercetin |
| Carbonic anhydrase II | CA2 | P00918 | CHEMBL205 | Lyase | 0.346957709 | 23 / 16 | Dimethyl Quercetin |
| Carbonic anhydrase VII | CA7 | P43166 | CHEMBL2326 | Lyase | 0.346957709 | 9 / 13 | Dimethyl Quercetin |
| Carbonic anhydrase XII | CA12 | O43570 | CHEMBL3242 | Lyase | 0.346957709 | 14 / 16 | Dimethyl Quercetin |
| Carbonic anhydrase IV | CA4 | P22748 | CHEMBL3729 | Lyase | 0.346957709 | 9 / 12 | Dimethyl Quercetin |
| Cytochrome P450 1B1 | CYP1B1 | Q16678 | CHEMBL4878 | Cytochrome P450 | 0.346957709 | 12 / 46 | Dimethyl Quercetin |
| Beta amyloid A4 protein | APP | P05067 | CHEMBL2487 | Membrane receptor | 0.250033463 | 3 / 9 | Dimethyl Quercetin |
| NADPH oxidase 4 | NOX4 | Q9NPH5 | CHEMBL1250375 | Enzyme | 0.233887255 | 6 / 8 | Dimethyl Quercetin |
| Plasminogen | PLG | P00747 | CHEMBL1801 | Protease | 0.225803139 | 6 / 2 | Dimethyl Quercetin |
| Induced myeloid leukemia cell differentiation protein Mcl-1 | MCL1 | Q07820 | CHEMBL4361 | Other cytosolic protein | 0.209643327 | 7 / 4 | Dimethyl Quercetin |
| Beta-secretase 1 | BACE1 | P56817 | CHEMBL4822 | Protease | 0.169270502 | 19 / 17 | Dimethyl Quercetin |
| Multidrug resistance-associated protein 1 | ABCC1 | P33527 | CHEMBL3004 | Primary active transporter | 0.161187413 | 7 / 11 | Dimethyl Quercetin |
| ATP-binding cassette sub-family G member 2 | ABCG2 | Q9UNQ0 | CHEMBL5393 | Primary active transporter | 0.153092564 | 7 / 49 | Dimethyl Quercetin |
| P-glycoprotein 1 | ABCB1 | P08183 | CHEMBL4302 | Primary active transporter | 0.136969565 | 17 / 47 | Dimethyl Quercetin |
| Cytochrome P450 19A1 | CYP19A1 | P11511 | CHEMBL1978 | Cytochrome P450 | 0.120823672 | 7 / 16 | Dimethyl Quercetin |
| Estradiol 17-beta-dehydrogenase 2 | HSD17B2 | P37059 | CHEMBL2789 | Enzyme | 0.120823672 | 12 / 3 | Dimethyl Quercetin |
| Thrombin | F2 | P00734 | CHEMBL204 | Protease | 0.120823672 | 16 / 2 | Dimethyl Quercetin |
| Epidermal growth factor receptor erbB1 | EGFR | P00533 | CHEMBL203 | Kinase | 0.112748418 | 8 / 24 | Dimethyl Quercetin |
| Insulin-like growth factor I receptor | IGF1R | P08069 | CHEMBL1957 | Kinase | 0.112748418 | 3 / 3 | Dimethyl Quercetin |
| Adenosine A1 receptor (by homology) | ADORA1 | P30542 | CHEMBL226 | Family A G protein-coupled receptor | 0.112748418 | 8 / 22 | Dimethyl Quercetin |
| Delta opioid receptor | OPRD1 | P41143 | CHEMBL236 | Family A G protein-coupled receptor | 0.112748418 | 2 / 5 | Dimethyl Quercetin |
| Acetylcholinesterase | ACHE | P22303 | CHEMBL220 | Hydrolase | 0.104671941 | 4 / 30 | Dimethyl Quercetin |
| Arachidonate 15-lipoxygenase | ALOX15 | P16050 | CHEMBL2903 | Enzyme | 0.104671941 | 9 / 5 | Dimethyl Quercetin |
| Arachidonate 12-lipoxygenase | ALOX12 | P18054 | CHEMBL3687 | Enzyme | 0.104671941 | 10 / 5 | Dimethyl Quercetin |
| Estradiol 17-beta-dehydrogenase 1 | HSD17B1 | P14061 | CHEMBL3181 | Enzyme | 0.104671941 | 12 / 3 | Dimethyl Quercetin |
| Cyclin-dependent kinase 1 | CDK1 | P06493 | CHEMBL308 | Kinase | 0.104671941 | 10 / 9 | Dimethyl Quercetin |
| Hepatocyte growth factor receptor | MET | P08581 | CHEMBL3717 | Kinase | 0.104671941 | 4 / 4 | Dimethyl Quercetin |
| Estrogen receptor beta | ESR2 | Q92731 | CHEMBL242 | Nuclear receptor | 0.104671941 | 53 / 15 | Dimethyl Quercetin |
| PI3-kinase p110-gamma subunit | PIK3CG | P48736 | CHEMBL3267 | Enzyme | 0.104671941 | 3 / 1 | Dimethyl Quercetin |
| Monoamine oxidase A | MAOA | P21397 | CHEMBL1951 | Oxidoreductase | 0.104671941 | 5 / 5 | Dimethyl Quercetin |
| Adenosine A2a receptor | ADORA2A | P29274 | CHEMBL251 | Family A G protein-coupled receptor | 0.104671941 | 7 / 10 | Dimethyl Quercetin |
| Mu opioid receptor | OPRM1 | P35372 | CHEMBL233 | Family A G protein-coupled receptor | 0.104671941 | 2 / 1 | Dimethyl Quercetin |
| Vasopressin V2 receptor | AVPR2 | P30518 | CHEMBL1790 | Family A G protein-coupled receptor | 0.104671941 | 3 / 1 | Dimethyl Quercetin |
| Tyrosine-protein kinase receptor FLT3 | FLT3 | P36888 | CHEMBL1974 | Kinase | 0.104671941 | 7 / 6 | Dimethyl Quercetin |
| Serine/threonine-protein kinase PIM1 | PIM1 | P11309 | CHEMBL2147 | Kinase | 0.104671941 | 8 / 6 | Dimethyl Quercetin |
| Arachidonate 5-lipoxygenase | ALOX5 | P09917 | CHEMBL215 | Oxidoreductase | 0.104671941 | 5 / 47 | Dimethyl Quercetin |
| Serine/threonine-protein kinase Aurora-B | AURKB | Q96GD4 | CHEMBL2185 | Kinase | 0.104671941 | 3 / 4 | Dimethyl Quercetin |
| Dopamine D4 receptor | DRD4 | P21917 | CHEMBL219 | Family A G protein-coupled receptor | 0.104671941 | 4 / 1 | Dimethyl Quercetin |
| Glyoxalase I | GLO1 | Q04760 | CHEMBL2424 | Enzyme | 0.104671941 | 3 / 4 | Dimethyl Quercetin |
| Myeloperoxidase | MPO | P05164 | CHEMBL2439 | Enzyme | 0.104671941 | 1 / 1 | Dimethyl Quercetin |
| PI3-kinase p85-alpha subunit | PIK3R1 | P27986 | CHEMBL2506 | Enzyme | 0.104671941 | 1 / 1 | Dimethyl Quercetin |
| Death-associated protein kinase 1 | DAPK1 | P53355 | CHEMBL2558 | Kinase | 0.104671941 | 2 / 2 | Dimethyl Quercetin |
| Liver glycogen phosphorylase | PYGL | P06737 | CHEMBL2568 | Enzyme | 0.104671941 | 1 / 1 | Dimethyl Quercetin |
| Carbonic anhydrase I | CA1 | P00915 | CHEMBL261 | Lyase | 0.104671941 | 8 / 6 | Dimethyl Quercetin |
| Glycogen synthase kinase-3 beta | GSK3B | P49841 | CHEMBL262 | Kinase | 0.104671941 | 10 / 4 | Dimethyl Quercetin |
| Tyrosine-protein kinase SRC | SRC | P12931 | CHEMBL267 | Kinase | 0.104671941 | 2 / 10 | Dimethyl Quercetin |
| Focal adhesion kinase 1 | PTK2 | Q05397 | CHEMBL2695 | Kinase | 0.104671941 | 1 / 2 | Dimethyl Quercetin |
| Vascular endothelial growth factor receptor 2 | KDR | P35968 | CHEMBL279 | Kinase | 0.104671941 | 2 / 3 | Dimethyl Quercetin |
| Matrix metalloproteinase 13 | MMP13 | P45452 | CHEMBL280 | Protease | 0.104671941 | 1 / 1 | Dimethyl Quercetin |
| Matrix metalloproteinase 3 | MMP3 | P08254 | CHEMBL283 | Protease | 0.104671941 | 1 / 1 | Dimethyl Quercetin |
| Carbonic anhydrase III | CA3 | P07451 | CHEMBL2885 | Lyase | 0.104671941 | 1 / 1 | Dimethyl Quercetin |
| Serine/threonine-protein kinase PLK1 | PLK1 | P53350 | CHEMBL3024 | Kinase | 0.104671941 | 2 / 3 | Dimethyl Quercetin |
| Carbonic anhydrase VI | CA6 | P23280 | CHEMBL3025 | Lyase | 0.104671941 | 2 / 1 | Dimethyl Quercetin |
| Matrix metalloproteinase 9 | MMP9 | P14780 | CHEMBL321 | Protease | 0.104671941 | 4 / 2 | Dimethyl Quercetin |
| Matrix metalloproteinase 2 | MMP2 | P08253 | CHEMBL333 | Protease | 0.104671941 | 4 / 2 | Dimethyl Quercetin |
| Protein kinase N1 | PKN1 | Q16512 | CHEMBL3384 | Kinase | 0.104671941 | 2 / 2 | Dimethyl Quercetin |
| Carbonic anhydrase XIV | CA14 | Q9ULX7 | CHEMBL3510 | Lyase | 0.104671941 | 1 / 1 | Dimethyl Quercetin |
| Carbonic anhydrase IX | CA9 | Q16790 | CHEMBL3594 | Lyase | 0.104671941 | 9 / 5 | Dimethyl Quercetin |
| Casein kinase II alpha | CSNK2A1 | P68400 | CHEMBL3629 | Kinase | 0.104671941 | 2 / 2 | Dimethyl Quercetin |
| Serine/threonine-protein kinase NEK2 | NEK2 | P51955 | CHEMBL3835 | Kinase | 0.104671941 | 1 / 2 | Dimethyl Quercetin |
| Interleukin-8 receptor A | CXCR1 | P25024 | CHEMBL4029 | Family A G protein-coupled receptor | 0.104671941 | 2 / 1 | Dimethyl Quercetin |
| CaM kinase II beta | CAMK2B | Q13554 | CHEMBL4121 | Kinase | 0.104671941 | 2 / 1 | Dimethyl Quercetin |
| ALK tyrosine kinase receptor | ALK | Q9UM73 | CHEMBL4247 | Kinase | 0.104671941 | 4 / 3 | Dimethyl Quercetin |
| Serine/threonine-protein kinase AKT | AKT1 | P31749 | CHEMBL4282 | Kinase | 0.104671941 | 2 / 4 | Dimethyl Quercetin |
| Serine/threonine-protein kinase NEK6 | NEK6 | Q9HC98 | CHEMBL4309 | Kinase | 0.104671941 | 1 / 2 | Dimethyl Quercetin |
| Phospholipase A2 group 1B | PLA2G1B | P04054 | CHEMBL4426 | Enzyme | 0.104671941 | 1 / 1 | Dimethyl Quercetin |
| Carbonic anhydrase VA | CA5A | P35218 | CHEMBL4789 | Lyase | 0.104671941 | 1 / 1 | Dimethyl Quercetin |
| Tyrosine-protein kinase receptor UFO | AXL | P30530 | CHEMBL4895 | Kinase | 0.104671941 | 3 / 3 | Dimethyl Quercetin |
| NUAK family SNF1-like kinase 1 | NUAK1 | O60285 | CHEMBL5784 | Kinase | 0.104671941 | 1 / 2 | Dimethyl Quercetin |
| Aldo-keto reductase family 1 member C2 (by homology) | AKR1C2 | P52895 | CHEMBL5847 | Enzyme | 0.104671941 | 1 / 1 | Dimethyl Quercetin |
| Aldo-keto reductase family 1 member C1 (by homology) | AKR1C1 | Q04828 | CHEMBL5905 | Enzyme | 0.104671941 | 1 / 1 | Dimethyl Quercetin |
| Aldo-keto-reductase family 1 member C3 (by homology) | AKR1C3 | P42330 | CHEMBL4681 | Enzyme | 0.104671941 | 1 / 1 | Dimethyl Quercetin |
| Aldo-keto reductase family 1 member C4 (by homology) | AKR1C4 | P17516 | CHEMBL4999 | Enzyme | 0.104671941 | 1 / 1 | Dimethyl Quercetin |
| Carbonic anhydrase XIII (by homology) | CA13 | Q8N1Q1 | CHEMBL3912 | Lyase | 0.104671941 | 1 / 1 | Dimethyl Quercetin |
| Aldehyde reductase (by homology) | AKR1A1 | P14550 | CHEMBL2246 | Enzyme | 0.104671941 | 1 / 1 | Dimethyl Quercetin |
| Microtubule-associated protein tau | MAPT | P10636 | CHEMBL1293224 | Unclassified protein | 0.104671941 | 1 / 1 | Dimethyl Quercetin |
| Lysine-specific demethylase 4D-like | KDM4E | B2RXH2 | CHEMBL1293226 | Eraser | 0.104671941 | 2 / 2 | Dimethyl Quercetin |
| G-protein coupled receptor 35 | GPR35 | Q9HC97 | CHEMBL1293267 | Family A G protein-coupled receptor | 0.104671941 | 2 / 4 | Dimethyl Quercetin |
| DNA topoisomerase II alpha | TOP2A | P11388 | CHEMBL1806 | Isomerase | 0.104671941 | 1 / 1 | Dimethyl Quercetin |
| Insulin receptor | INSR | P06213 | CHEMBL1981 | Kinase | 0.104671941 | 1 / 1 | Dimethyl Quercetin |
| Myosin light chain kinase, smooth muscle | MYLK | Q15746 | CHEMBL2428 | Kinase | 0.104671941 | 1 / 1 | Dimethyl Quercetin |
| Tyrosine-protein kinase SYK | SYK | P43405 | CHEMBL2599 | Kinase | 0.104671941 | 3 / 3 | Dimethyl Quercetin |
| DNA-(apurinic or apyrimidinic site) lyase | APEX1 | P27695 | CHEMBL5619 | Enzyme | 0.104671941 | 1 / 1 | Dimethyl Quercetin |
| Phospholipase A2 group IIA | PLA2G2A | P14555 | CHEMBL3474 | Enzyme | 0.104671941 | 0 / 2 | Dimethyl Quercetin |
| Cyclin-dependent kinase 5/CDK5 activator 1 | CDK5R1 CDK5 | Q15078 Q00535 | CHEMBL1907600 | Kinase | 0.104671941 | 11 / 16 | Dimethyl Quercetin |
| Cyclin-dependent kinase 1/cyclin B | CCNB3 CDK1 CCNB1 CCNB2 | Q8WWL7 P06493 P14635 O95067 | CHEMBL2094127 | Other cytosolic protein | 0.104671941 | 12 / 4 | Dimethyl Quercetin |
| Cyclin-dependent kinase 6 | CDK6 | Q00534 | CHEMBL2508 | Kinase | 0.104671941 | 4 / 3 | Dimethyl Quercetin |
| Cyclin-dependent kinase 2 | CDK2 | P24941 | CHEMBL301 | Kinase | 0.104671941 | 11 / 13 | Dimethyl Quercetin |
| Arginase-1 (by homology) | ARG1 | P05089 | CHEMBL1075097 | Enzyme | 0.104671941 | 2 / 2 | Dimethyl Quercetin |
| Telomerase reverse transcriptase | TERT | O14746 | CHEMBL2916 | Enzyme | 0.104671941 | 10 / 22 | Dimethyl Quercetin |
| Nitric oxide synthase, inducible (by homology) | NOS2 | P35228 | CHEMBL4481 | Enzyme | 0.104671941 | 5 / 3 | Dimethyl Quercetin |
| Solute carrier family 22 member 12 | SLC22A12 | Q96S37 | CHEMBL6120 | Electrochemical transporter | 0.104671941 | 6 / 1 | Dimethyl Quercetin |
| Beta-galactoside alpha-2,6-sialyltransferase 1 | ST6GAL1 | P15907 | CHEMBL3596075 | Transferase | 0.104671941 | 0 / 2 | Dimethyl Quercetin |
| Stem cell growth factor receptor | KIT | P10721 | CHEMBL1936 | Kinase | 0.104671941 | 2 / 2 | Dimethyl Quercetin |
| Estrogen receptor alpha | ESR1 | P03372 | CHEMBL206 | Nuclear receptor | 0.104671941 | 42 / 16 | Dimethyl Quercetin |
| Adenosine A3 receptor | ADORA3 | P0DMS8 | CHEMBL256 | Family A G protein-coupled receptor | 0.104671941 | 1 / 20 | Dimethyl Quercetin |
| Tyrosinase | TYR | P14679 | CHEMBL1973 | Oxidoreductase | 0.104671941 | 2 / 2 | Dimethyl Quercetin |
| Aryl hydrocarbon receptor | AHR | P35869 | CHEMBL3201 | Transcription factor | 0.104671941 | 1 / 1 | Dimethyl Quercetin |
| Estrogen-related receptor alpha | ESRRA | P11474 | CHEMBL3429 | Nuclear receptor | 0.104671941 | 2 / 1 | Dimethyl Quercetin |
| G-protein coupled receptor 35 | GPR35 | Q9HC97 | CHEMBL1293267 | Family A G protein-coupled receptor | 1 | 1 / 2 | Ellagic acid |
| Receptor protein-tyrosine kinase erbB-2 | ERBB2 | P04626 | CHEMBL1824 | Kinase | 1 | 1 / 1 | Ellagic acid |
| Aldose reductase | AKR1B1 | P15121 | CHEMBL1900 | Enzyme | 1 | 2 / 8 | Ellagic acid |
| Cyclin-dependent kinase 4/cyclin D1 | CCND1 CDK4 | P24385 P11802 | CHEMBL1907601 | Kinase | 1 | 1 / 1 | Ellagic acid |
| Platelet-derived growth factor receptor beta | PDGFRB | P09619 | CHEMBL1913 | Kinase | 1 | 1 / 1 | Ellagic acid |
| Vascular endothelial growth factor receptor 3 | FLT4 | P35916 | CHEMBL1955 | Kinase | 1 | 1 / 1 | Ellagic acid |
| Insulin-like growth factor I receptor | IGF1R | P08069 | CHEMBL1957 | Kinase | 1 | 1 / 2 | Ellagic acid |
| Insulin receptor | INSR | P06213 | CHEMBL1981 | Kinase | 1 | 1 / 1 | Ellagic acid |
| Epidermal growth factor receptor erbB1 | EGFR | P00533 | CHEMBL203 | Kinase | 1 | 1 / 2 | Ellagic acid |
| Carbonic anhydrase II | CA2 | P00918 | CHEMBL205 | Lyase | 1 | 1 / 6 | Ellagic acid |
| Cyclin-dependent kinase 2/cyclin A | CDK2 CCNA1 CCNA2 | P24941 P78396 P20248 | CHEMBL2094128 | Other cytosolic protein | 1 | 1 / 1 | Ellagic acid |
| Serine/threonine-protein kinase Aurora-B | AURKB | Q96GD4 | CHEMBL2185 | Kinase | 1 | 1 / 3 | Ellagic acid |
| Carbonic anhydrase VII | CA7 | P43166 | CHEMBL2326 | Lyase | 1 | 1 / 17 | Ellagic acid |
| Carbonic anhydrase I | CA1 | P00915 | CHEMBL261 | Lyase | 1 | 1 / 15 | Ellagic acid |
| Glycogen synthase kinase-3 beta | GSK3B | P49841 | CHEMBL262 | Kinase | 1 | 1 / 1 | Ellagic acid |
| Tyrosine-protein kinase SRC | SRC | P12931 | CHEMBL267 | Kinase | 1 | 1 / 1 | Ellagic acid |
| Focal adhesion kinase 1 | PTK2 | Q05397 | CHEMBL2695 | Kinase | 1 | 1 / 1 | Ellagic acid |
| Vascular endothelial growth factor receptor 2 | KDR | P35968 | CHEMBL279 | Kinase | 1 | 1 / 2 | Ellagic acid |
| Serine/threonine-protein kinase PLK1 | PLK1 | P53350 | CHEMBL3024 | Kinase | 1 | 1 / 2 | Ellagic acid |
| Carbonic anhydrase VI | CA6 | P23280 | CHEMBL3025 | Lyase | 1 | 1 / 8 | Ellagic acid |
| Carbonic anhydrase XII | CA12 | O43570 | CHEMBL3242 | Lyase | 1 | 1 / 35 | Ellagic acid |
| Carbonic anhydrase XIV | CA14 | Q9ULX7 | CHEMBL3510 | Lyase | 1 | 1 / 14 | Ellagic acid |
| Carbonic anhydrase IX | CA9 | Q16790 | CHEMBL3594 | Lyase | 1 | 1 / 43 | Ellagic acid |
| Casein kinase II alpha | CSNK2A1 | P68400 | CHEMBL3629 | Kinase | 1 | 1 / 1 | Ellagic acid |
| Hepatocyte growth factor receptor | MET | P08581 | CHEMBL3717 | Kinase | 1 | 1 / 2 | Ellagic acid |
| Carbonic anhydrase IV | CA4 | P22748 | CHEMBL3729 | Lyase | 1 | 1 / 7 | Ellagic acid |
| Serine/threonine-protein kinase PLK4 | PLK4 | O00444 | CHEMBL3788 | Kinase | 1 | 1 / 1 | Ellagic acid |
| Carbonic anhydrase XIII | CA13 | Q8N1Q1 | CHEMBL3912 | Lyase | 1 | 1 / 21 | Ellagic acid |
| Tyrosine-protein kinase TIE-2 | TEK | Q02763 | CHEMBL4128 | Kinase | 1 | 1 / 1 | Ellagic acid |
| Serine/threonine-protein kinase AKT | AKT1 | P31749 | CHEMBL4282 | Kinase | 1 | 1 / 1 | Ellagic acid |
| Serine/threonine-protein kinase Aurora-A | AURKA | O14965 | CHEMBL4722 | Kinase | 1 | 1 / 1 | Ellagic acid |
| Carbonic anhydrase VA | CA5A | P35218 | CHEMBL4789 | Lyase | 1 | 1 / 9 | Ellagic acid |
| Beta-secretase 1 | BACE1 | P56817 | CHEMBL4822 | Protease | 1 | 1 / 5 | Ellagic acid |
| Mitogen-activated protein kinase kinase kinase 8 | MAP3K8 | P41279 | CHEMBL4899 | Kinase | 1 | 1 / 1 | Ellagic acid |
| Serine/threonine-protein kinase B-raf | BRAF | P15056 | CHEMBL5145 | Kinase | 1 | 1 / 1 | Ellagic acid |
| Ephrin receptor | EPHB4 | P54760 | CHEMBL5147 | Kinase | 1 | 1 / 1 | Ellagic acid |
| Heat shock 70 kDa protein 1 | HSPA1A | P0DMV8 | CHEMBL5460 | Other cytosolic protein | 1 | 1 / 1 | Ellagic acid |
| NUAK family SNF1-like kinase 1 | NUAK1 | O60285 | CHEMBL5784 | Kinase | 1 | 1 / 1 | Ellagic acid |
| Squalene monooxygenase (by homology) | SQLE | Q14534 | CHEMBL3592 | Enzyme | 1 | 1 / 1 | Ellagic acid |
| Tyrosine-protein kinase FGR (by homology) | FGR | P09769 | CHEMBL4454 | Kinase | 1 | 1 / 1 | Ellagic acid |
| Tyrosine-protein kinase Lyn (by homology) | LYN | P07948 | CHEMBL3905 | Kinase | 1 | 1 / 1 | Ellagic acid |
| Glutathione reductase | GSR | P00390 | CHEMBL2755 | Oxidoreductase | 0.174646372 | 1 / 1 | Ellagic acid |
| Troponin, cardiac muscle | TNNC1 TNNT2 TNNI3 | P63316 P45379 P19429 | CHEMBL2095202 | Unclassified protein | 0.119403562 | 0 / 1 | Ellagic acid |
| Xanthine dehydrogenase | XDH | P47989 | CHEMBL1929 | Oxidoreductase | 0.119403562 | 0 / 3 | Ellagic acid |
| D-amino-acid oxidase | DAO | P14920 | CHEMBL5485 | Enzyme | 0.111501865 | 0 / 1 | Ellagic acid |
| Cyclooxygenase-2 | PTGS2 | P35354 | CHEMBL230 | Oxidoreductase | 0.111501865 | 0 / 3 | Ellagic acid |
| Estrogen receptor alpha | ESR1 | P03372 | CHEMBL206 | Nuclear receptor | 0.111501865 | 0 / 43 | Ellagic acid |
| Estrogen receptor beta | ESR2 | Q92731 | CHEMBL242 | Nuclear receptor | 0.111501865 | 0 / 61 | Ellagic acid |
| Monoamine oxidase A | MAOA | P21397 | CHEMBL1951 | Oxidoreductase | 0.111501865 | 0 / 34 | Ellagic acid |
| Estradiol 17-beta-dehydrogenase 3 | HSD17B3 | P37058 | CHEMBL4234 | Enzyme | 0.111501865 | 0 / 19 | Ellagic acid |
| Protein-tyrosine phosphatase 1B | PTPN1 | P18031 | CHEMBL335 | Phosphatase | 0.111501865 | 2 / 0 | Ellagic acid |
| Arachidonate 5-lipoxygenase | ALOX5 | P09917 | CHEMBL215 | Oxidoreductase | 0.111501865 | 0 / 1 | Ellagic acid |
| Carbonyl reductase [NADPH] 1 | CBR1 | P16152 | CHEMBL5586 | Enzyme | 0.111501865 | 0 / 1 | Ellagic acid |
| Cyclin-dependent kinase 5/CDK5 activator 1 | CDK5R1 CDK5 | Q15078 Q00535 | CHEMBL1907600 | Kinase | 0.111501865 | 0 / 6 | Ellagic acid |
| Carboxylesterase 2 | CES2 | O00748 | CHEMBL3180 | Enzyme | 0.111501865 | 1 / 0 | Ellagic acid |
| Carbonic anhydrase VB | CA5B | Q9Y2D0 | CHEMBL3969 | Lyase | 0.111501865 | 0 / 9 | Ellagic acid |
| Alpha-synuclein | SNCA | P37840 | CHEMBL6152 | Unclassified protein | 0.111501865 | 1 / 0 | Ellagic acid |
| Carbonic anhydrase II | CA2 | P00918 | CHEMBL205 | Lyase | 0.937741278 | 62 / 20 | Ferrulic Acid |
| Carbonic anhydrase VII | CA7 | P43166 | CHEMBL2326 | Lyase | 0.937741278 | 11 / 19 | Ferrulic Acid |
| Carbonic anhydrase I | CA1 | P00915 | CHEMBL261 | Lyase | 0.937741278 | 57 / 24 | Ferrulic Acid |
| Carbonic anhydrase VI | CA6 | P23280 | CHEMBL3025 | Lyase | 0.937741278 | 7 / 15 | Ferrulic Acid |
| Carbonic anhydrase XII | CA12 | O43570 | CHEMBL3242 | Lyase | 0.937741278 | 27 / 21 | Ferrulic Acid |
| Carbonic anhydrase XIV | CA14 | Q9ULX7 | CHEMBL3510 | Lyase | 0.937741278 | 10 / 19 | Ferrulic Acid |
| Carbonic anhydrase IX | CA9 | Q16790 | CHEMBL3594 | Lyase | 0.937741278 | 22 / 26 | Ferrulic Acid |
| Carbonic anhydrase VA | CA5A | P35218 | CHEMBL4789 | Lyase | 0.937741278 | 10 / 15 | Ferrulic Acid |
| Carbonic anhydrase VB | CA5B | Q9Y2D0 | CHEMBL3969 | Lyase | 0.133586597 | 5 / 13 | Ferrulic Acid |
| Monoamine oxidase B | MAOB | P27338 | CHEMBL2039 | Oxidoreductase | 0.082333793 | 2 / 38 | Ferrulic Acid |
| Aldose reductase | AKR1B1 | P15121 | CHEMBL1900 | Enzyme | 0.07218044 | 68 / 34 | Ferrulic Acid |
| Arachidonate 5-lipoxygenase | ALOX5 | P09917 | CHEMBL215 | Oxidoreductase | 0.07218044 | 1 / 44 | Ferrulic Acid |
| Matrix metalloproteinase 9 | MMP9 | P14780 | CHEMBL321 | Protease | 0.07218044 | 2 / 24 | Ferrulic Acid |
| Matrix metalloproteinase 1 | MMP1 | P03956 | CHEMBL332 | Protease | 0.07218044 | 2 / 24 | Ferrulic Acid |
| Matrix metalloproteinase 2 | MMP2 | P08253 | CHEMBL333 | Protease | 0.07218044 | 2 / 23 | Ferrulic Acid |
| Protein-tyrosine phosphatase 1B | PTPN1 | P18031 | CHEMBL335 | Phosphatase | 0.07218044 | 35 / 2 | Ferrulic Acid |
| Carbonic anhydrase XIII (by homology) | CA13 | Q8N1Q1 | CHEMBL3912 | Lyase | 0.061914931 | 10 / 10 | Ferrulic Acid |
| Carbonic anhydrase III | CA3 | P07451 | CHEMBL2885 | Lyase | 0.061914931 | 5 / 4 | Ferrulic Acid |
| Beta amyloid A4 protein | APP | P05067 | CHEMBL2487 | Membrane receptor | 0.051713312 | 0 / 55 | Ferrulic Acid |
| Nuclear factor erythroid 2-related factor 2 | NFE2L2 | Q16236 | CHEMBL1075094 | Unclassified protein | 0.041470299 | 0 / 4 | Ferrulic Acid |
| Signal transducer and activator of transcription 3 | STAT3 | P40763 | CHEMBL4026 | Transcription factor | 0.041470299 | 0 / 1 | Ferrulic Acid |
| 11-beta-hydroxysteroid dehydrogenase 1 | HSD11B1 | P28845 | CHEMBL4235 | Enzyme | 0.041470299 | 0 / 4 | Ferrulic Acid |
| Estrogen receptor beta | ESR2 | Q92731 | CHEMBL242 | Nuclear receptor | 0.041470299 | 5 / 17 | Ferrulic Acid |
| Carbonic anhydrase IV | CA4 | P22748 | CHEMBL3729 | Lyase | 0.041470299 | 4 / 7 | Ferrulic Acid |
| Toll-like receptor 4 (by homology) | TLR4 | O00206 | CHEMBL5255 | Toll-like and Il-1 receptors | 0.041470299 | 0 / 13 | Ferrulic Acid |
| Cyclooxygenase-1 | PTGS1 | P23219 | CHEMBL221 | Oxidoreductase | 0.031226558 | 4 / 17 | Ferrulic Acid |
| Hepatocyte growth factor receptor | MET | P08581 | CHEMBL3717 | Kinase | 0.031226558 | 0 / 22 | Ferrulic Acid |
| Cytochrome P450 1A1 | CYP1A1 | P04798 | CHEMBL2231 | Cytochrome P450 | 0.031226558 | 0 / 9 | Ferrulic Acid |
| Cytochrome P450 1A2 | CYP1A2 | P05177 | CHEMBL3356 | Cytochrome P450 | 0.031226558 | 0 / 5 | Ferrulic Acid |
| Quinone reductase 2 | NQO2 | P16083 | CHEMBL3959 | Enzyme | 0.031226558 | 0 / 17 | Ferrulic Acid |
| Cytochrome P450 1B1 | CYP1B1 | Q16678 | CHEMBL4878 | Cytochrome P450 | 0.031226558 | 0 / 25 | Ferrulic Acid |
| Carboxypeptidase A1 | CPA1 | P15085 | CHEMBL2088 | Protease | 0.031226558 | 11 / 0 | Ferrulic Acid |
| Epidermal growth factor receptor erbB1 | EGFR | P00533 | CHEMBL203 | Kinase | 0.031226558 | 4 / 34 | Ferrulic Acid |
| Cyclooxygenase-2 | PTGS2 | P35354 | CHEMBL230 | Oxidoreductase | 0.031226558 | 11 / 47 | Ferrulic Acid |
| Transthyretin | TTR | P02766 | CHEMBL3194 | Secreted protein | 0.031226558 | 5 / 6 | Ferrulic Acid |
| Lysine-specific demethylase 4D-like | KDM4E | B2RXH2 | CHEMBL1293226 | Eraser | 0.031226558 | 5 / 0 | Ferrulic Acid |
| Lysine-specific demethylase 3A | KDM3A | Q9Y4C1 | CHEMBL1938209 | Eraser | 0.031226558 | 2 / 0 | Ferrulic Acid |
| Lysine-specific demethylase 6B | KDM6B | O15054 | CHEMBL1938211 | Eraser | 0.031226558 | 2 / 0 | Ferrulic Acid |
| Alpha-ketoglutarate-dependent dioxygenase FTO | FTO | Q9C0B1 | CHEMBL2331065 | Oxidoreductase | 0.031226558 | 2 / 0 | Ferrulic Acid |
| Lysine-specific demethylase 4A | KDM4A | O75164 | CHEMBL5896 | Eraser | 0.031226558 | 7 / 0 | Ferrulic Acid |
| Lysine-specific demethylase 4C | KDM4C | Q9H3R0 | CHEMBL6175 | Eraser | 0.031226558 | 10 / 0 | Ferrulic Acid |
| Tubulin beta-1 chain | TUBB1 | Q9H4B7 | CHEMBL1915 | Structural protein | 0.031226558 | 0 / 29 | Ferrulic Acid |
| Nuclear factor NF-kappa-B p65 subunit | RELA | Q04206 | CHEMBL5533 | Transcription factor | 0.031226558 | 0 / 13 | Ferrulic Acid |
| Tyrosine-protein kinase FYN | FYN | P06241 | CHEMBL1841 | Kinase | 0.031226558 | 4 / 2 | Ferrulic Acid |
| Tyrosine-protein kinase LCK | LCK | P06239 | CHEMBL258 | Kinase | 0.031226558 | 3 / 9 | Ferrulic Acid |
| Monocarboxylate transporter 1 (by homology) | SLC16A1 | P53985 | CHEMBL4360 | Electrochemical transporter | 0.031226558 | 2 / 12 | Ferrulic Acid |
| Toll-like receptor (TLR7/TLR9) | TLR9 | Q9NR96 | CHEMBL5804 | Toll-like and Il-1 receptors | 0.031226558 | 0 / 3 | Ferrulic Acid |
| Aldo-keto reductase family 1 member B10 | AKR1B10 | O60218 | CHEMBL5983 | Enzyme | 0.031226558 | 0 / 18 | Ferrulic Acid |
| Arachidonate 15-lipoxygenase | ALOX15 | P16050 | CHEMBL2903 | Enzyme | 0.031226558 | 0 / 2 | Ferrulic Acid |
| Protein kinase C epsilon | PRKCE | Q02156 | CHEMBL3582 | Kinase | 0.031226558 | 0 / 2 | Ferrulic Acid |
| Coagulation factor VII/tissue factor | F3 | P13726 | CHEMBL4081 | Surface antigen | 0.031226558 | 0 / 9 | Ferrulic Acid |
| Nitric oxide synthase, inducible | NOS2 | P35228 | CHEMBL4481 | Enzyme | 0.031226558 | 0 / 7 | Ferrulic Acid |
| Low affinity neurotrophin receptor p75NTR | NGFR | P08138 | CHEMBL4762 | Membrane receptor | 0.031226558 | 2 / 0 | Ferrulic Acid |
| Cyclin-dependent kinase 4/cyclin D1 | CCND1 CDK4 | P24385 P11802 | CHEMBL1907601 | Kinase | 0.031226558 | 0 / 1 | Ferrulic Acid |
| Tubulin beta-3 chain | TUBB3 | Q13509 | CHEMBL2597 | Structural protein | 0.031226558 | 0 / 1 | Ferrulic Acid |
| P-glycoprotein 1 | ABCB1 | P08183 | CHEMBL4302 | Primary active transporter | 0.031226558 | 0 / 14 | Ferrulic Acid |
| Fructose-1,6-bisphosphatase | FBP1 | P09467 | CHEMBL3975 | Enzyme | 0.031226558 | 22 / 0 | Ferrulic Acid |
| DNA topoisomerase II alpha | TOP2A | P11388 | CHEMBL1806 | Isomerase | 0.031226558 | 0 / 15 | Ferrulic Acid |
| Glyoxalase I | GLO1 | Q04760 | CHEMBL2424 | Enzyme | 0.031226558 | 0 / 5 | Ferrulic Acid |
| Beta-secretase 1 | BACE1 | P56817 | CHEMBL4822 | Protease | 0.031226558 | 1 / 62 | Ferrulic Acid |
| Angiotensin-converting enzyme (by homology) | ACE | P12821 | CHEMBL1808 | Protease | 0.031226558 | 16 / 0 | Ferrulic Acid |
| Renin | REN | P00797 | CHEMBL286 | Protease | 0.031226558 | 1 / 0 | Ferrulic Acid |
| Poly [ADP-ribose] polymerase-1 | PARP1 | P09874 | CHEMBL3105 | Enzyme | 0.031226558 | 10 / 0 | Ferrulic Acid |
| Monoamine oxidase A | MAOA | P21397 | CHEMBL1951 | Oxidoreductase | 0.031226558 | 2 / 14 | Ferrulic Acid |
| Aryl hydrocarbon receptor | AHR | P35869 | CHEMBL3201 | Transcription factor | 0.031226558 | 0 / 4 | Ferrulic Acid |
| Lysine-specific demethylase 2A | KDM2A | Q9Y2K7 | CHEMBL1938210 | Eraser | 0.031226558 | 3 / 0 | Ferrulic Acid |
| Thiopurine S-methyltransferase | TPMT | P51580 | CHEMBL2500 | Enzyme | 0.031226558 | 4 / 0 | Ferrulic Acid |
| Axin1/beta-catenin | CTNNB1 | P35222 | CHEMBL5866 | Unclassified protein | 0.031226558 | 3 / 0 | Ferrulic Acid |
| Thrombin | F2 | P00734 | CHEMBL204 | Protease | 0.031226558 | 10 / 0 | Ferrulic Acid |
| Solute carrier family 13 member 5 | SLC13A5 | Q86YT5 | CHEMBL3769293 | Electrochemical transporter | 0.031226558 | 10 / 0 | Ferrulic Acid |
| Carbonic anhydrase II | CA2 | P00918 | CHEMBL205 | Lyase | 0.999408275 | 5 / 49 | Gallic Acid |
| Carbonic anhydrase VII | CA7 | P43166 | CHEMBL2326 | Lyase | 0.999408275 | 3 / 26 | Gallic Acid |
| Carbonic anhydrase I | CA1 | P00915 | CHEMBL261 | Lyase | 0.999408275 | 5 / 47 | Gallic Acid |
| Carbonic anhydrase III | CA3 | P07451 | CHEMBL2885 | Lyase | 0.999408275 | 1 / 3 | Gallic Acid |
| Carbonic anhydrase VI | CA6 | P23280 | CHEMBL3025 | Lyase | 0.999408275 | 2 / 5 | Gallic Acid |
| Carbonic anhydrase XII | CA12 | O43570 | CHEMBL3242 | Lyase | 0.999408275 | 5 / 30 | Gallic Acid |
| Carbonic anhydrase XIV | CA14 | Q9ULX7 | CHEMBL3510 | Lyase | 0.999408275 | 3 / 26 | Gallic Acid |
| Carbonic anhydrase IX | CA9 | Q16790 | CHEMBL3594 | Lyase | 0.999408275 | 4 / 28 | Gallic Acid |
| Alpha-(1,3)-fucosyltransferase 7 | FUT7 | Q11130 | CHEMBL3596077 | Transferase | 0.999408275 | 1 / 2 | Gallic Acid |
| Carbonic anhydrase IV | CA4 | P22748 | CHEMBL3729 | Lyase | 0.999408275 | 2 / 4 | Gallic Acid |
| Carbonic anhydrase VB | CA5B | Q9Y2D0 | CHEMBL3969 | Lyase | 0.999408275 | 1 / 2 | Gallic Acid |
| Carbonic anhydrase VA | CA5A | P35218 | CHEMBL4789 | Lyase | 0.999408275 | 1 / 3 | Gallic Acid |
| Carbonic anhydrase XIII (by homology) | CA13 | Q8N1Q1 | CHEMBL3912 | Lyase | 0.999408275 | 1 / 2 | Gallic Acid |
| Squalene monooxygenase (by homology) | SQLE | Q14534 | CHEMBL3592 | Enzyme | 0.150097811 | 0 / 7 | Gallic Acid |
| L-lactate dehydrogenase A chain | LDHA | P00338 | CHEMBL4835 | Enzyme | 0.12507596 | 0 / 14 | Gallic Acid |
| L-lactate dehydrogenase B chain | LDHB | P07195 | CHEMBL4940 | Enzyme | 0.12507596 | 0 / 11 | Gallic Acid |
| Transthyretin | TTR | P02766 | CHEMBL3194 | Secreted protein | 0.12507596 | 0 / 8 | Gallic Acid |
| Insulin-like growth factor I receptor | IGF1R | P08069 | CHEMBL1957 | Kinase | 0.12507596 | 0 / 3 | Gallic Acid |
| ALK tyrosine kinase receptor | ALK | Q9UM73 | CHEMBL4247 | Kinase | 0.12507596 | 0 / 3 | Gallic Acid |
| Plasminogen activator inhibitor-1 | SERPINE1 | P05121 | CHEMBL3475 | Secreted protein | 0.12507596 | 0 / 8 | Gallic Acid |
| Estrogen receptor beta | ESR2 | Q92731 | CHEMBL242 | Nuclear receptor | 0.12507596 | 0 / 8 | Gallic Acid |
| Apoptosis regulator Bcl-X | BCL2L1 | Q07817 | CHEMBL4625 | Other ion channel | 0.116739032 | 0 / 2 | Gallic Acid |
| G-protein coupled receptor 35 | GPR35 | Q9HC97 | CHEMBL1293267 | Family A G protein-coupled receptor | 0.116739032 | 0 / 2 | Gallic Acid |
| Catechol O-methyltransferase (by homology) | COMT | P21964 | CHEMBL2023 | Transferase | 0.116739032 | 0 / 7 | Gallic Acid |
| Thiopurine S-methyltransferase | TPMT | P51580 | CHEMBL2500 | Enzyme | 0.116739032 | 0 / 5 | Gallic Acid |
| Voltage-gated calcium channel alpha2/delta subunit 1 (by homology) | CACNA2D1 | P54289 | CHEMBL1919 | Calcium channel auxiliary subunit alpha2delta family | 0.063025715 | 14 / 0 | Histidine |
| Carboxypeptidase B2 isoform A | CPB2 | Q96IY4 | CHEMBL3419 | Protease | 0.053517944 | 5 / 60 | Histidine |
| Histamine H3 receptor | HRH3 | Q9Y5N1 | CHEMBL264 | Family A G protein-coupled receptor | 0.053517944 | 0 / 129 | Histidine |
| Histamine H4 receptor | HRH4 | Q9H3N8 | CHEMBL3759 | Family A G protein-coupled receptor | 0.053517944 | 0 / 80 | Histidine |
| Glutamate receptor ionotropic, AMPA 1 | GRIA1 | P42261 | CHEMBL2009 | Ligand-gated ion channel | 0.053517944 | 11 / 0 | Histidine |
| Glutamate receptor ionotropic, AMPA 4 | GRIA4 | P48058 | CHEMBL3190 | Ligand-gated ion channel | 0.053517944 | 11 / 0 | Histidine |
| Glutamate receptor ionotropic, AMPA 2 | GRIA2 | P42262 | CHEMBL4016 | Ligand-gated ion channel | 0.053517944 | 10 / 0 | Histidine |
| Glucosamine--fructose-6-phosphate aminotransferase [isomerizing] 1 | GFPT1 | Q06210 | CHEMBL1909481 | Enzyme | 0.053517944 | 2 / 0 | Histidine |
| GABA-B receptor | GABBR2 GABBR1 | O75899 Q9UBS5 | CHEMBL2111463 | Family C G protein-coupled receptor | 0.053517944 | 16 / 0 | Histidine |
| Glutamate receptor ionotropic kainate 5 | GRIK5 | Q16478 | CHEMBL2675 | Ligand-gated ion channel | 0.053517944 | 8 / 0 | Histidine |
| GABA-B receptor (by homology) | GABBR1 | Q9UBS5 | CHEMBL2064 | Family C G protein-coupled receptor | 0.043918633 | 5 / 0 | Histidine |
| Histamine H2 receptor | HRH2 | P25021 | CHEMBL1941 | Family A G protein-coupled receptor | 0.043918633 | 0 / 6 | Histidine |
| Histamine H1 receptor | HRH1 | P35367 | CHEMBL231 | Family A G protein-coupled receptor | 0.043918633 | 0 / 14 | Histidine |
| Kynureninase | KYNU | Q16719 | CHEMBL5100 | Enzyme | 0.043918633 | 4 / 0 | Histidine |
| Aminopeptidase A | ENPEP | Q07075 | CHEMBL3439 | Protease | 0.043918633 | 11 / 0 | Histidine |
| Kynurenine 3-monooxygenase (by homology) | KMO | O15229 | CHEMBL2145 | Oxidoreductase | 0.043918633 | 8 / 0 | Histidine |
| GABA transporter 1 (by homology) | SLC6A1 | P30531 | CHEMBL1903 | Electrochemical transporter | 0.043918633 | 4 / 0 | Histidine |
| Estrogen receptor alpha | ESR1 | P03372 | CHEMBL206 | Nuclear receptor | 0.321690507 | 93 / 73 | Isoformononetin |
| Estradiol 17-beta-dehydrogenase 2 | HSD17B2 | P37059 | CHEMBL2789 | Enzyme | 0.313572767 | 76 / 3 | Isoformononetin |
| Carbonic anhydrase XII | CA12 | O43570 | CHEMBL3242 | Lyase | 0.264392858 | 133 / 34 | Isoformononetin |
| Aldehyde dehydrogenase | ALDH2 | P05091 | CHEMBL1935 | Oxidoreductase | 0.264392858 | 13 / 44 | Isoformononetin |
| Interleukin-2 | IL2 | P60568 | CHEMBL5880 | Secreted protein | 0.239574696 | 0 / 4 | Isoformononetin |
| Epidermal growth factor receptor erbB1 | EGFR | P00533 | CHEMBL203 | Kinase | 0.239574696 | 162 / 26 | Isoformononetin |
| Cytochrome P450 19A1 | CYP19A1 | P11511 | CHEMBL1978 | Cytochrome P450 | 0.223449266 | 34 / 28 | Isoformononetin |
| Carbonic anhydrase VII | CA7 | P43166 | CHEMBL2326 | Lyase | 0.215238178 | 30 / 22 | Isoformononetin |
| Estrogen receptor beta | ESR2 | Q92731 | CHEMBL242 | Nuclear receptor | 0.207053974 | 89 / 58 | Isoformononetin |
| Estradiol 17-beta-dehydrogenase 1 | HSD17B1 | P14061 | CHEMBL3181 | Enzyme | 0.19883376 | 74 / 4 | Isoformononetin |
| Cyclooxygenase-1 | PTGS1 | P23219 | CHEMBL221 | Oxidoreductase | 0.190656977 | 16 / 1 | Isoformononetin |
| ATP-binding cassette sub-family G member 2 | ABCG2 | Q9UNQ0 | CHEMBL5393 | Primary active transporter | 0.174270753 | 47 / 40 | Isoformononetin |
| Carbonic anhydrase IV | CA4 | P22748 | CHEMBL3729 | Lyase | 0.174270753 | 13 / 13 | Isoformononetin |
| 6-phosphofructo-2-kinase/fructose-2,6-bisphosphatase 3 | PFKFB3 | Q16875 | CHEMBL2331053 | Enzyme | 0.166097445 | 24 / 2 | Isoformononetin |
| Macrophage migration inhibitory factor | MIF | P14174 | CHEMBL2085 | Enzyme | 0.166097445 | 22 / 8 | Isoformononetin |
| Serotonin 2a (5-HT2a) receptor | HTR2A | P28223 | CHEMBL224 | Family A G protein-coupled receptor | 0.166097445 | 46 / 3 | Isoformononetin |
| Adenosine A1 receptor | ADORA1 | P30542 | CHEMBL226 | Family A G protein-coupled receptor | 0.149732594 | 116 / 19 | Isoformononetin |
| Adenosine A2a receptor | ADORA2A | P29274 | CHEMBL251 | Family A G protein-coupled receptor | 0.149732594 | 83 / 14 | Isoformononetin |
| Monoamine oxidase A | MAOA | P21397 | CHEMBL1951 | Oxidoreductase | 0.141522086 | 43 / 70 | Isoformononetin |
| Monoamine oxidase B | MAOB | P27338 | CHEMBL2039 | Oxidoreductase | 0.141522086 | 87 / 142 | Isoformononetin |
| Norepinephrine transporter | SLC6A2 | P23975 | CHEMBL222 | Electrochemical transporter | 0.125142649 | 3 / 1 | Isoformononetin |
| Carbonic anhydrase II | CA2 | P00918 | CHEMBL205 | Lyase | 0.116965063 | 244 / 26 | Isoformononetin |
| P-glycoprotein 1 | ABCB1 | P08183 | CHEMBL4302 | Primary active transporter | 0.116965063 | 17 / 32 | Isoformononetin |
| Carbonic anhydrase I | CA1 | P00915 | CHEMBL261 | Lyase | 0.116965063 | 215 / 27 | Isoformononetin |
| Thromboxane-A synthase | TBXAS1 | P24557 | CHEMBL1835 | Cytochrome P450 | 0.116965063 | 6 / 1 | Isoformononetin |
| Arachidonate 12-lipoxygenase | ALOX12 | P18054 | CHEMBL3687 | Enzyme | 0.108770969 | 1 / 12 | Isoformononetin |
| Carbonyl reductase [NADPH] 1 | CBR1 | P16152 | CHEMBL5586 | Enzyme | 0.108770969 | 1 / 3 | Isoformononetin |
| Serotonin 2c (5-HT2c) receptor | HTR2C | P28335 | CHEMBL225 | Family A G protein-coupled receptor | 0.108770969 | 21 / 3 | Isoformononetin |
| Estrogen-related receptor alpha | ESRRA | P11474 | CHEMBL3429 | Nuclear receptor | 0.108770969 | 1 / 2 | Isoformononetin |
| Estrogen-related receptor beta | ESRRB | O95718 | CHEMBL3751 | Nuclear receptor | 0.108770969 | 1 / 1 | Isoformononetin |
| Receptor-type tyrosine-protein phosphatase S | PTPRS | Q13332 | CHEMBL2396508 | Phosphatase | 0.108770969 | 0 / 7 | Isoformononetin |
| Maltase-glucoamylase | MGAM | O43451 | CHEMBL2074 | Hydrolase | 0.100578902 | 2 / 1 | Isoformononetin |
| Arachidonate 15-lipoxygenase | ALOX15 | P16050 | CHEMBL2903 | Enzyme | 0.100578902 | 7 / 9 | Isoformononetin |
| Peroxisome proliferator-activated receptor alpha | PPARA | Q07869 | CHEMBL239 | Nuclear receptor | 0.100578902 | 0 / 1 | Isoformononetin |
| Xanthine dehydrogenase | XDH | P47989 | CHEMBL1929 | Oxidoreductase | 0.100578902 | 5 / 19 | Isoformononetin |
| Tyrosinase | TYR | P14679 | CHEMBL1973 | Oxidoreductase | 0.100578902 | 2 / 2 | Isoformononetin |
| NADPH oxidase 4 | NOX4 | Q9NPH5 | CHEMBL1250375 | Enzyme | 0.100578902 | 9 / 7 | Isoformononetin |
| Serum paraoxonase/arylesterase 1 | PON1 | P27169 | CHEMBL3167 | Enzyme | 0.100578902 | 0 / 1 | Isoformononetin |
| Carbonic anhydrase IX | CA9 | Q16790 | CHEMBL3594 | Lyase | 0.100578902 | 154 / 41 | Isoformononetin |
| Dual specificity mitogen-activated protein kinase kinase 1 | MAP2K1 | Q02750 | CHEMBL3587 | Kinase | 0.100578902 | 17 / 0 | Isoformononetin |
| Acetylcholinesterase | ACHE | P22303 | CHEMBL220 | Hydrolase | 0.100578902 | 26 / 67 | Isoformononetin |
| Protein-tyrosine phosphatase 1B | PTPN1 | P18031 | CHEMBL335 | Phosphatase | 0.100578902 | 28 / 17 | Isoformononetin |
| Vascular endothelial growth factor receptor 1 | FLT1 | P17948 | CHEMBL1868 | Kinase | 0.100578902 | 22 / 0 | Isoformononetin |
| Kinesin-1 heavy chain/ Tyrosine-protein kinase receptor RET | RET | P07949 | CHEMBL2041 | Kinase | 0.100578902 | 58 / 0 | Isoformononetin |
| Neuronal acetylcholine receptor protein alpha-7 subunit | CHRNA7 | P36544 | CHEMBL2492 | Ligand-gated ion channel | 0.100578902 | 16 / 0 | Isoformononetin |
| Ephrin type-B receptor 2 | EPHB2 | P29323 | CHEMBL3290 | Kinase | 0.100578902 | 4 / 0 | Isoformononetin |
| Dopamine D1 receptor | DRD1 | P21728 | CHEMBL2056 | Family A G protein-coupled receptor | 0.100578902 | 63 / 0 | Isoformononetin |
| Carbonic anhydrase XIV | CA14 | Q9ULX7 | CHEMBL3510 | Lyase | 0.100578902 | 20 / 12 | Isoformononetin |
| Glutamate receptor ionotropic, AMPA 1 | GRIA1 | P42261 | CHEMBL2009 | Ligand-gated ion channel | 0.100578902 | 9 / 0 | Isoformononetin |
| Serine/threonine-protein kinase Chk1 | CHEK1 | O14757 | CHEMBL4630 | Kinase | 0.100578902 | 106 / 0 | Isoformononetin |
| Serine/threonine-protein kinase WEE1 | WEE1 | P30291 | CHEMBL5491 | Kinase | 0.100578902 | 81 / 0 | Isoformononetin |
| Carbonic anhydrase III | CA3 | P07451 | CHEMBL2885 | Lyase | 0.100578902 | 0 / 8 | Isoformononetin |
| Toll-like receptor (TLR7/TLR9) | TLR9 | Q9NR96 | CHEMBL5804 | Toll-like and Il-1 receptors | 0.100578902 | 6 / 2 | Isoformononetin |
| Ezrin | EZR | P15311 | CHEMBL1932896 | Unclassified protein | 0.100578902 | 3 / 0 | Isoformononetin |
| Beta-secretase 1 | BACE1 | P56817 | CHEMBL4822 | Protease | 0.100578902 | 80 / 19 | Isoformononetin |
| ADAM17 | ADAM17 | P78536 | CHEMBL3706 | Protease | 0.100578902 | 229 / 0 | Isoformononetin |
| Histone deacetylase 1 | HDAC1 | Q13547 | CHEMBL325 | Eraser | 0.100578902 | 213 / 0 | Isoformononetin |
| Histone deacetylase 6 | HDAC6 | Q9UBN7 | CHEMBL1865 | Eraser | 0.100578902 | 168 / 0 | Isoformononetin |
| Alkaline phosphatase, tissue-nonspecific isozyme | ALPL | P05186 | CHEMBL5979 | Enzyme | 0.100578902 | 18 / 0 | Isoformononetin |
| Dual specificity phosphatase Cdc25A | CDC25A | P30304 | CHEMBL3775 | Phosphatase | 0.100578902 | 11 / 0 | Isoformononetin |
| Dual specificity phosphatase Cdc25B | CDC25B | P30305 | CHEMBL4804 | Phosphatase | 0.100578902 | 13 / 0 | Isoformononetin |
| Matrix metalloproteinase 1 | MMP1 | P03956 | CHEMBL332 | Protease | 0.100578902 | 461 / 0 | Isoformononetin |
| Coagulation factor VII/tissue factor | F3 | P13726 | CHEMBL4081 | Surface antigen | 0.100578902 | 17 / 0 | Isoformononetin |
| Ribosomal protein S6 kinase alpha 2 | RPS6KA2 | Q15349 | CHEMBL3906 | Kinase | 0.100578902 | 6 / 0 | Isoformononetin |
| Lysine-specific histone demethylase 1 | KDM1A | O60341 | CHEMBL6136 | Eraser | 0.100578902 | 7 / 0 | Isoformononetin |
| Carbonic anhydrase VI | CA6 | P23280 | CHEMBL3025 | Lyase | 0.100578902 | 16 / 7 | Isoformononetin |
| Carbonic anhydrase XIII | CA13 | Q8N1Q1 | CHEMBL3912 | Lyase | 0.100578902 | 13 / 16 | Isoformononetin |
| Carbonic anhydrase VB | CA5B | Q9Y2D0 | CHEMBL3969 | Lyase | 0.100578902 | 12 / 7 | Isoformononetin |
| Carbonic anhydrase VA | CA5A | P35218 | CHEMBL4789 | Lyase | 0.100578902 | 14 / 6 | Isoformononetin |
| Glycogen synthase kinase-3 alpha | GSK3A | P49840 | CHEMBL2850 | Kinase | 0.100578902 | 23 / 0 | Isoformononetin |
| Serine/threonine-protein kinase PIM2 | PIM2 | Q9P1W9 | CHEMBL4523 | Kinase | 0.100578902 | 16 / 0 | Isoformononetin |
| Serine/threonine-protein kinase MARK1 | MARK1 | Q9P0L2 | CHEMBL5940 | Kinase | 0.100578902 | 5 / 0 | Isoformononetin |
| Transient receptor potential cation channel subfamily M member 8 (by homology) | TRPM8 | Q7Z2W7 | CHEMBL1075319 | Voltage-gated ion channel | 0.100578902 | 17 / 0 | Isoformononetin |
| Sodium/hydrogen exchanger 1 (by homology) | SLC9A1 | P19634 | CHEMBL2781 | Electrochemical transporter | 0.100578902 | 11 / 0 | Isoformononetin |
| Rho-associated protein kinase 1 | ROCK1 | Q13464 | CHEMBL3231 | Kinase | 0.100578902 | 54 / 0 | Isoformononetin |
| Tyrosine-protein kinase ABL | ABL1 | P00519 | CHEMBL1862 | Kinase | 0.100578902 | 89 / 0 | Isoformononetin |
| Cyclin-dependent kinase 2/cyclin A | CDK2 CCNA1 CCNA2 | P24941 P78396 P20248 | CHEMBL2094128 | Other cytosolic protein | 0.100578902 | 75 / 0 | Isoformononetin |
| Tyrosine-protein kinase HCK | HCK | P08631 | CHEMBL3234 | Kinase | 0.100578902 | 35 / 0 | Isoformononetin |
| PI4-kinase beta subunit | PI4KB | Q9UBF8 | CHEMBL3268 | Enzyme | 0.100578902 | 13 / 0 | Isoformononetin |
| Ephrin receptor | EPHB4 | P54760 | CHEMBL5147 | Kinase | 0.100578902 | 37 / 0 | Isoformononetin |
| Phospholipase A-2-activating protein | PLAA | Q9Y263 | CHEMBL6114 | Unclassified protein | 0.100578902 | 6 / 0 | Isoformononetin |
| Histone deacetylase 3/Nuclear receptor corepressor 2 (HDAC3/NCoR2) | NCOR2 HDAC3 | Q9Y618 O15379 | CHEMBL2111363 | Eraser | 0.100578902 | 6 / 0 | Isoformononetin |
| Histone deacetylase 11 | HDAC11 | Q96DB2 | CHEMBL3310 | Eraser | 0.100578902 | 8 / 0 | Isoformononetin |
| Tyrosine-protein kinase BRK | PTK6 | Q13882 | CHEMBL4601 | Kinase | 0.100578902 | 17 / 0 | Isoformononetin |
| Histone deacetylase 10 | HDAC10 | Q969S8 | CHEMBL5103 | Eraser | 0.100578902 | 12 / 0 | Isoformononetin |
| Matrix metalloproteinase 8 | MMP8 | P22894 | CHEMBL4588 | Protease | 0.100578902 | 174 / 0 | Isoformononetin |
| Dual-specificity tyrosine-phosphorylation regulated kinase 1A (by homology) | DYRK1A | Q13627 | CHEMBL2292 | Kinase | 0.100578902 | 61 / 0 | Isoformononetin |
| Metabotropic glutamate receptor 4 | GRM4 | Q14833 | CHEMBL2736 | Family C G protein-coupled receptor | 0.100578902 | 7 / 0 | Isoformononetin |
| Matrix metalloproteinase 7 | MMP7 | P09237 | CHEMBL4073 | Protease | 0.100578902 | 92 / 0 | Isoformononetin |
| Ribosomal protein S6 kinase 1 | RPS6KB1 | P23443 | CHEMBL4501 | Kinase | 0.100578902 | 62 / 0 | Isoformononetin |
| Serine/threonine-protein kinase Aurora-A | AURKA | O14965 | CHEMBL4722 | Kinase | 0.100578902 | 162 / 0 | Isoformononetin |
| Cyclin-dependent kinase 4/cyclin D | CCND3 CCND1 CDK4 CCND2 | P30281 P24385 P11802 P30279 | CHEMBL2095942 | Other cytosolic protein | 0.100578902 | 3 / 0 | Isoformononetin |
| Alkaline phosphatase placental-like | ALPG | P10696 | CHEMBL3402 | Enzyme | 0.100578902 | 3 / 0 | Isoformononetin |
| Bone morphogenetic protein 1 | BMP1 | P13497 | CHEMBL3898 | Protease | 0.100578902 | 125 / 0 | Isoformononetin |
| Pyruvate dehydrogenase kinase isoform 1 | PDK1 | Q15118 | CHEMBL4766 | Kinase | 0.100578902 | 147 / 0 | Isoformononetin |
| Serine/threonine-protein kinase RAF | RAF1 | P04049 | CHEMBL1906 | Kinase | 0.100578902 | 69 / 0 | Isoformononetin |
| 3-phosphoinositide dependent protein kinase-1 | PDPK1 | O15530 | CHEMBL2534 | Kinase | 0.100578902 | 23 / 0 | Isoformononetin |
| Serine/threonine-protein kinase B-raf | BRAF | P15056 | CHEMBL5145 | Kinase | 0.100578902 | 93 / 0 | Isoformononetin |
| Cyclin-dependent kinase 4 | CDK4 | P11802 | CHEMBL331 | Kinase | 0.100578902 | 41 / 0 | Isoformononetin |
| Matrix metalloproteinase 25 | MMP25 | Q9NPA2 | CHEMBL1795103 | Enzyme | 0.100578902 | 1 / 0 | Isoformononetin |
| NADPH oxidase 4 | NOX4 | Q9NPH5 | CHEMBL1250375 | Enzyme | 1 | 7 / 8 | Kaempferol |
| Aldose reductase (by homology) | AKR1B1 | P15121 | CHEMBL1900 | Enzyme | 1 | 18 / 71 | Kaempferol |
| Xanthine dehydrogenase | XDH | P47989 | CHEMBL1929 | Oxidoreductase | 1 | 12 / 20 | Kaempferol |
| Tyrosinase | TYR | P14679 | CHEMBL1973 | Oxidoreductase | 1 | 2 / 3 | Kaempferol |
| Tyrosine-protein kinase receptor FLT3 | FLT3 | P36888 | CHEMBL1974 | Kinase | 1 | 5 / 7 | Kaempferol |
| Carbonic anhydrase II | CA2 | P00918 | CHEMBL205 | Lyase | 1 | 8 / 14 | Kaempferol |
| Arachidonate 5-lipoxygenase | ALOX5 | P09917 | CHEMBL215 | Oxidoreductase | 1 | 5 / 47 | Kaempferol |
| Carbonic anhydrase VII | CA7 | P43166 | CHEMBL2326 | Lyase | 1 | 8 / 13 | Kaempferol |
| Estradiol 17-beta-dehydrogenase 2 | HSD17B2 | P37059 | CHEMBL2789 | Enzyme | 1 | 9 / 3 | Kaempferol |
| Multidrug resistance-associated protein 1 | ABCC1 | P33527 | CHEMBL3004 | Primary active transporter | 1 | 7 / 11 | Kaempferol |
| Estradiol 17-beta-dehydrogenase 1 | HSD17B1 | P14061 | CHEMBL3181 | Enzyme | 1 | 9 / 4 | Kaempferol |
| Aryl hydrocarbon receptor | AHR | P35869 | CHEMBL3201 | Transcription factor | 1 | 1 / 1 | Kaempferol |
| Carbonic anhydrase XII | CA12 | O43570 | CHEMBL3242 | Lyase | 1 | 10 / 17 | Kaempferol |
| Estrogen-related receptor alpha | ESRRA | P11474 | CHEMBL3429 | Nuclear receptor | 1 | 2 / 2 | Kaempferol |
| P-glycoprotein 1 | ABCB1 | P08183 | CHEMBL4302 | Primary active transporter | 1 | 12 / 48 | Kaempferol |
| Cytochrome P450 1B1 | CYP1B1 | Q16678 | CHEMBL4878 | Cytochrome P450 | 1 | 12 / 46 | Kaempferol |
| ATP-binding cassette sub-family G member 2 | ABCG2 | Q9UNQ0 | CHEMBL5393 | Primary active transporter | 1 | 6 / 47 | Kaempferol |
| Adenosine A1 receptor (by homology) | ADORA1 | P30542 | CHEMBL226 | Family A G protein-coupled receptor | 0.795047263 | 6 / 23 | Kaempferol |
| Carbonic anhydrase IV | CA4 | P22748 | CHEMBL3729 | Lyase | 0.795047263 | 7 / 12 | Kaempferol |
| Acetylcholinesterase | ACHE | P22303 | CHEMBL220 | Hydrolase | 0.768469201 | 4 / 26 | Kaempferol |
| Monoamine oxidase A | MAOA | P21397 | CHEMBL1951 | Oxidoreductase | 0.658019738 | 5 / 18 | Kaempferol |
| Glyoxalase I | GLO1 | Q04760 | CHEMBL2424 | Enzyme | 0.658019738 | 3 / 4 | Kaempferol |
| Tyrosine-protein kinase SYK | SYK | P43405 | CHEMBL2599 | Kinase | 0.658019738 | 3 / 3 | Kaempferol |
| Glycogen synthase kinase-3 beta | GSK3B | P49841 | CHEMBL262 | Kinase | 0.658019738 | 3 / 6 | Kaempferol |
| Matrix metalloproteinase 9 | MMP9 | P14780 | CHEMBL321 | Protease | 0.658019738 | 2 / 2 | Kaempferol |
| Matrix metalloproteinase 2 | MMP2 | P08253 | CHEMBL333 | Protease | 0.658019738 | 2 / 2 | Kaempferol |
| Arachidonate 15-lipoxygenase | ALOX15 | P16050 | CHEMBL2903 | Enzyme | 0.64978075 | 6 / 8 | Kaempferol |
| Arachidonate 12-lipoxygenase | ALOX12 | P18054 | CHEMBL3687 | Enzyme | 0.633326262 | 9 / 10 | Kaempferol |
| Receptor-type tyrosine-protein phosphatase S | PTPRS | Q13332 | CHEMBL2396508 | Phosphatase | 0.608446264 | 7 / 8 | Kaempferol |
| Adenosine A2a receptor (by homology) | ADORA2A | P29274 | CHEMBL251 | Family A G protein-coupled receptor | 0.5673019 | 5 / 11 | Kaempferol |
| Cyclin-dependent kinase 5/CDK5 activator 1 | CDK5R1 CDK5 | Q15078 Q00535 | CHEMBL1907600 | Kinase | 0.517921498 | 6 / 18 | Kaempferol |
| Cyclin-dependent kinase 1/cyclin B | CCNB3 CDK1 CCNB1 CCNB2 | Q8WWL7 P06493 P14635 O95067 | CHEMBL2094127 | Other cytosolic protein | 0.517921498 | 4 / 11 | Kaempferol |
| Arginase-1 (by homology) | ARG1 | P05089 | CHEMBL1075097 | Enzyme | 0.517921498 | 2 / 2 | Kaempferol |
| G-protein coupled receptor 35 | GPR35 | Q9HC97 | CHEMBL1293267 | Family A G protein-coupled receptor | 0.501530018 | 2 / 4 | Kaempferol |
| Estrogen receptor beta | ESR2 | Q92731 | CHEMBL242 | Nuclear receptor | 0.501530018 | 93 / 38 | Kaempferol |
| Death-associated protein kinase 1 | DAPK1 | P53355 | CHEMBL2558 | Kinase | 0.501530018 | 2 / 2 | Kaempferol |
| DNA-3-methyladenine glycosylase | MPG | P29372 | CHEMBL3396943 | Enzyme | 0.501530018 | 1 / 1 | Kaempferol |
| Solute carrier family 22 member 12 | SLC22A12 | Q96S37 | CHEMBL6120 | Electrochemical transporter | 0.501530018 | 6 / 1 | Kaempferol |
| Transthyretin | TTR | P02766 | CHEMBL3194 | Secreted protein | 0.484896363 | 2 / 3 | Kaempferol |
| Aldo-keto reductase family 1 member B10 | AKR1B10 | O60218 | CHEMBL5983 | Enzyme | 0.484896363 | 2 / 3 | Kaempferol |
| Tankyrase-2 | TNKS2 | Q9H2K2 | CHEMBL6154 | Enzyme | 0.484896363 | 4 / 12 | Kaempferol |
| Tankyrase-1 | TNKS | O95271 | CHEMBL6164 | Enzyme | 0.484896363 | 4 / 28 | Kaempferol |
| Cyclin-dependent kinase 6 | CDK6 | Q00534 | CHEMBL2508 | Kinase | 0.476835618 | 3 / 4 | Kaempferol |
| Cyclin-dependent kinase 2 | CDK2 | P24941 | CHEMBL301 | Kinase | 0.476835618 | 1 / 17 | Kaempferol |
| Cytochrome P450 19A1 | CYP19A1 | P11511 | CHEMBL1978 | Cytochrome P450 | 0.427205275 | 6 / 18 | Kaempferol |
| Casein kinase II alpha | CSNK2A1 | P68400 | CHEMBL3629 | Kinase | 0.427205275 | 3 / 2 | Kaempferol |
| Epidermal growth factor receptor erbB1 | EGFR | P00533 | CHEMBL203 | Kinase | 0.40264338 | 7 / 29 | Kaempferol |
| Vasopressin V2 receptor | AVPR2 | P30518 | CHEMBL1790 | Family A G protein-coupled receptor | 0.40264338 | 1 / 1 | Kaempferol |
| Insulin-like growth factor I receptor | IGF1R | P08069 | CHEMBL1957 | Kinase | 0.40264338 | 3 / 3 | Kaempferol |
| Thrombin | F2 | P00734 | CHEMBL204 | Protease | 0.40264338 | 11 / 3 | Kaempferol |
| Serine/threonine-protein kinase PIM1 | PIM1 | P11309 | CHEMBL2147 | Kinase | 0.40264338 | 8 / 7 | Kaempferol |
| Serine/threonine-protein kinase Aurora-B | AURKB | Q96GD4 | CHEMBL2185 | Kinase | 0.40264338 | 3 / 4 | Kaempferol |
| Dopamine D4 receptor | DRD4 | P21917 | CHEMBL219 | Family A G protein-coupled receptor | 0.40264338 | 1 / 1 | Kaempferol |
| Myeloperoxidase | MPO | P05164 | CHEMBL2439 | Enzyme | 0.40264338 | 1 / 1 | Kaempferol |
| PI3-kinase p85-alpha subunit | PIK3R1 | P27986 | CHEMBL2506 | Enzyme | 0.40264338 | 1 / 1 | Kaempferol |
| Liver glycogen phosphorylase | PYGL | P06737 | CHEMBL2568 | Enzyme | 0.40264338 | 1 / 1 | Kaempferol |
| Carbonic anhydrase I | CA1 | P00915 | CHEMBL261 | Lyase | 0.40264338 | 4 / 5 | Kaempferol |
| Tyrosine-protein kinase SRC | SRC | P12931 | CHEMBL267 | Kinase | 0.40264338 | 3 / 10 | Kaempferol |
| Focal adhesion kinase 1 | PTK2 | Q05397 | CHEMBL2695 | Kinase | 0.40264338 | 1 / 2 | Kaempferol |
| Vascular endothelial growth factor receptor 2 | KDR | P35968 | CHEMBL279 | Kinase | 0.40264338 | 2 / 3 | Kaempferol |
| Matrix metalloproteinase 13 | MMP13 | P45452 | CHEMBL280 | Protease | 0.40264338 | 1 / 1 | Kaempferol |
| Matrix metalloproteinase 3 | MMP3 | P08254 | CHEMBL283 | Protease | 0.40264338 | 1 / 1 | Kaempferol |
| Carbonic anhydrase III | CA3 | P07451 | CHEMBL2885 | Lyase | 0.40264338 | 1 / 1 | Kaempferol |
| Serine/threonine-protein kinase PLK1 | PLK1 | P53350 | CHEMBL3024 | Kinase | 0.40264338 | 2 / 3 | Kaempferol |
| Carbonic anhydrase VI | CA6 | P23280 | CHEMBL3025 | Lyase | 0.40264338 | 1 / 1 | Kaempferol |
| Cyclin-dependent kinase 1 | CDK1 | P06493 | CHEMBL308 | Kinase | 0.40264338 | 3 / 13 | Kaempferol |
| Protein kinase N1 | PKN1 | Q16512 | CHEMBL3384 | Kinase | 0.40264338 | 1 / 3 | Kaempferol |
| Carbonic anhydrase XIV | CA14 | Q9ULX7 | CHEMBL3510 | Lyase | 0.40264338 | 1 / 1 | Kaempferol |
| Carbonic anhydrase IX | CA9 | Q16790 | CHEMBL3594 | Lyase | 0.40264338 | 3 / 6 | Kaempferol |
| Hepatocyte growth factor receptor | MET | P08581 | CHEMBL3717 | Kinase | 0.40264338 | 4 / 4 | Kaempferol |
| Serine/threonine-protein kinase NEK2 | NEK2 | P51955 | CHEMBL3835 | Kinase | 0.40264338 | 1 / 2 | Kaempferol |
| Interleukin-8 receptor A | CXCR1 | P25024 | CHEMBL4029 | Family A G protein-coupled receptor | 0.40264338 | 1 / 1 | Kaempferol |
| CaM kinase II beta | CAMK2B | Q13554 | CHEMBL4121 | Kinase | 0.40264338 | 1 / 2 | Kaempferol |
| ALK tyrosine kinase receptor | ALK | Q9UM73 | CHEMBL4247 | Kinase | 0.40264338 | 2 / 4 | Kaempferol |
| Serine/threonine-protein kinase AKT | AKT1 | P31749 | CHEMBL4282 | Kinase | 0.40264338 | 1 / 4 | Kaempferol |
| Serine/threonine-protein kinase NEK6 | NEK6 | Q9HC98 | CHEMBL4309 | Kinase | 0.40264338 | 1 / 2 | Kaempferol |
| Phospholipase A2 group 1B | PLA2G1B | P04054 | CHEMBL4426 | Enzyme | 0.40264338 | 1 / 1 | Kaempferol |
| Carbonic anhydrase VA | CA5A | P35218 | CHEMBL4789 | Lyase | 0.40264338 | 1 / 1 | Kaempferol |
| Beta-secretase 1 | BACE1 | P56817 | CHEMBL4822 | Protease | 0.40264338 | 6 / 17 | Kaempferol |
| Tyrosine-protein kinase receptor UFO | AXL | P30530 | CHEMBL4895 | Kinase | 0.40264338 | 2 / 4 | Kaempferol |
| NUAK family SNF1-like kinase 1 | NUAK1 | O60285 | CHEMBL5784 | Kinase | 0.40264338 | 1 / 2 | Kaempferol |
| Aldo-keto reductase family 1 member C2 (by homology) | AKR1C2 | P52895 | CHEMBL5847 | Enzyme | 0.40264338 | 1 / 1 | Kaempferol |
| Aldo-keto reductase family 1 member C1 (by homology) | AKR1C1 | Q04828 | CHEMBL5905 | Enzyme | 0.40264338 | 1 / 1 | Kaempferol |
| Aldo-keto-reductase family 1 member C3 (by homology) | AKR1C3 | P42330 | CHEMBL4681 | Enzyme | 0.40264338 | 1 / 1 | Kaempferol |
| Aldo-keto reductase family 1 member C4 (by homology) | AKR1C4 | P17516 | CHEMBL4999 | Enzyme | 0.40264338 | 1 / 1 | Kaempferol |
| Carbonic anhydrase XIII (by homology) | CA13 | Q8N1Q1 | CHEMBL3912 | Lyase | 0.40264338 | 1 / 1 | Kaempferol |
| Aldehyde reductase (by homology) | AKR1A1 | P14550 | CHEMBL2246 | Enzyme | 0.40264338 | 1 / 1 | Kaempferol |
| Beta amyloid A4 protein | APP | P05067 | CHEMBL2487 | Membrane receptor | 0.295453305 | 2 / 12 | Kaempferol |
| Poly [ADP-ribose] polymerase-1 | PARP1 | P09874 | CHEMBL3105 | Enzyme | 0.295453305 | 3 / 9 | Kaempferol |
| Matrix metalloproteinase 12 | MMP12 | P39900 | CHEMBL4393 | Protease | 0.295453305 | 1 / 1 | Kaempferol |
| Lymphocyte differentiation antigen CD38 | CD38 | P28907 | CHEMBL4660 | Enzyme | 0.295453305 | 2 / 2 | Kaempferol |
| DNA topoisomerase I (by homology) | TOP1 | P11387 | CHEMBL1781 | Isomerase | 0.295453305 | 1 / 1 | Kaempferol |
| Estrogen receptor alpha | ESR1 | P03372 | CHEMBL206 | Nuclear receptor | 0.270836923 | 73 / 38 | Kaempferol |
| Cyclooxygenase-2 | PTGS2 | P35354 | CHEMBL230 | Oxidoreductase | 0.270836923 | 1 / 25 | Kaempferol |
| Cystic fibrosis transmembrane conductance regulator | CFTR | P13569 | CHEMBL4051 | Other ion channel | 0.270836923 | 1 / 1 | Kaempferol |
| 6-phosphofructo-2-kinase/fructose-2,6-bisphosphatase 3 | PFKFB3 | Q16875 | CHEMBL2331053 | Enzyme | 0.262581506 | 2 / 2 | Kaempferol |
| AMY1C | AMY1A | P04745 | CHEMBL2478 | Enzyme | 0.237885168 | 1 / 1 | Kaempferol |
| G protein-coupled receptor kinase 6 | GRK6 | P43250 | CHEMBL6144 | Kinase | 0.237885168 | 2 / 4 | Kaempferol |
| Telomerase reverse transcriptase | TERT | O14746 | CHEMBL2916 | Enzyme | 0.229685699 | 9 / 22 | Kaempferol |
| Microtubule-associated protein tau | MAPT | P10636 | CHEMBL1293224 | Unclassified protein | 0.171978589 | 1 / 1 | Kaempferol |
| Beta-secretase 1 | BACE1 | P56817 | CHEMBL4822 | Protease | 0.30896078 | 2 / 12 | leucocianidol |
| Carbonic anhydrase VII | CA7 | P43166 | CHEMBL2326 | Lyase | 0.277371341 | 2 / 8 | leucocianidol |
| Carbonic anhydrase XII | CA12 | O43570 | CHEMBL3242 | Lyase | 0.277371341 | 27 / 8 | leucocianidol |
| Carbonic anhydrase IV | CA4 | P22748 | CHEMBL3729 | Lyase | 0.277371341 | 2 / 8 | leucocianidol |
| Stem cell growth factor receptor | KIT | P10721 | CHEMBL1936 | Kinase | 0.182601417 | 3 / 3 | leucocianidol |
| Tyrosine-protein kinase SRC | SRC | P12931 | CHEMBL267 | Kinase | 0.182601417 | 3 / 3 | leucocianidol |
| Vascular endothelial growth factor receptor 2 | KDR | P35968 | CHEMBL279 | Kinase | 0.182601417 | 3 / 10 | leucocianidol |
| Fibroblast growth factor receptor 1 | FGFR1 | P11362 | CHEMBL3650 | Kinase | 0.182601417 | 3 / 3 | leucocianidol |
| Hepatocyte growth factor receptor | MET | P08581 | CHEMBL3717 | Kinase | 0.182601417 | 3 / 4 | leucocianidol |
| Carbonic anhydrase II | CA2 | P00918 | CHEMBL205 | Lyase | 0.174646372 | 47 / 3 | leucocianidol |
| Carbonic anhydrase I | CA1 | P00915 | CHEMBL261 | Lyase | 0.174646372 | 46 / 2 | leucocianidol |
| Carbonic anhydrase III | CA3 | P07451 | CHEMBL2885 | Lyase | 0.174646372 | 1 / 2 | leucocianidol |
| Carbonic anhydrase VI | CA6 | P23280 | CHEMBL3025 | Lyase | 0.174646372 | 1 / 2 | leucocianidol |
| Carbonic anhydrase IX | CA9 | Q16790 | CHEMBL3594 | Lyase | 0.174646372 | 27 / 1 | leucocianidol |
| Carbonic anhydrase VB | CA5B | Q9Y2D0 | CHEMBL3969 | Lyase | 0.174646372 | 1 / 2 | leucocianidol |
| Carbonic anhydrase VA | CA5A | P35218 | CHEMBL4789 | Lyase | 0.174646372 | 1 / 2 | leucocianidol |
| Cyclooxygenase-1 | PTGS1 | P23219 | CHEMBL221 | Oxidoreductase | 0.143102156 | 3 / 10 | leucocianidol |
| P-glycoprotein 1 | ABCB1 | P08183 | CHEMBL4302 | Primary active transporter | 0.12730257 | 0 / 112 | leucocianidol |
| Matrix metalloproteinase 12 | MMP12 | P39900 | CHEMBL4393 | Protease | 0.119403562 | 8 / 2 | leucocianidol |
| Apoptosis regulator Bcl-2 | BCL2 | P10415 | CHEMBL4860 | Other ion channel | 0.111501865 | 1 / 7 | leucocianidol |
| Matrix metalloproteinase 13 | MMP13 | P45452 | CHEMBL280 | Protease | 0.111501865 | 8 / 1 | leucocianidol |
| Placenta growth factor | PGF | P49763 | CHEMBL1697671 | Unclassified protein | 0.111501865 | 0 / 3 | leucocianidol |
| Vascular endothelial growth factor A | VEGFA | P15692 | CHEMBL1783 | Secreted protein | 0.111501865 | 0 / 3 | leucocianidol |
| Matrix metalloproteinase 9 | MMP9 | P14780 | CHEMBL321 | Protease | 0.111501865 | 10 / 2 | leucocianidol |
| Microtubule-associated protein tau | MAPT | P10636 | CHEMBL1293224 | Unclassified protein | 0.111501865 | 0 / 1 | leucocianidol |
| DNA (cytosine-5)-methyltransferase 1 | DNMT1 | P26358 | CHEMBL1993 | Writer | 0.111501865 | 0 / 1 | leucocianidol |
| Dual-specificity tyrosine-phosphorylation regulated kinase 1A | DYRK1A | Q13627 | CHEMBL2292 | Kinase | 0.111501865 | 0 / 1 | leucocianidol |
| HERG | KCNH2 | Q12809 | CHEMBL240 | Voltage-gated ion channel | 0.111501865 | 0 / 4 | leucocianidol |
| Beta amyloid A4 protein | APP | P05067 | CHEMBL2487 | Membrane receptor | 0.111501865 | 0 / 2 | leucocianidol |
| MAP kinase p38 alpha | MAPK14 | Q16539 | CHEMBL260 | Kinase | 0.111501865 | 0 / 2 | leucocianidol |
| Telomerase reverse transcriptase | TERT | O14746 | CHEMBL2916 | Enzyme | 0.111501865 | 0 / 1 | leucocianidol |
| 6-phosphogluconate dehydrogenase | PGD | P52209 | CHEMBL3404 | Enzyme | 0.111501865 | 0 / 4 | leucocianidol |
| CMP-N-acetylneuraminate-beta-1,4-galactoside alpha-2,3-sialyltransferase | ST3GAL3 | Q11203 | CHEMBL3596076 | Transferase | 0.111501865 | 0 / 1 | leucocianidol |
| Alpha-(1,3)-fucosyltransferase 7 | FUT7 | Q11130 | CHEMBL3596077 | Transferase | 0.111501865 | 0/ 1 | leucocianidol |
| Fucosyltransferase 4 | FUT4 | P22083 | CHEMBL4996 | Enzyme | 0.111501865 | 0 / 1 | leucocianidol |
| Signal transducer and activator of transcription 1-alpha/beta | STAT1 | P42224 | CHEMBL6101 | Transcription factor | 0.111501865 | 0 / 1 | leucocianidol |
| Squalene monooxygenase (by homology) | SQLE | Q14534 | CHEMBL3592 | Enzyme | 0.111501865 | 0 / 3 | leucocianidol |
| Matrix metalloproteinase 2 | MMP2 | P08253 | CHEMBL333 | Protease | 0.111501865 | 1 / 4 | leucocianidol |
| GABA-A receptor; alpha-1/beta-2/gamma-2 | GABRA1 GABRB2 GABRG2 | P14867 P47870 P18507 | CHEMBL2095172 | Ligand-gated ion channel | 0.111501865 | 0 / 1 | leucocianidol |
| Carbonic anhydrase XIII | CA13 | Q8N1Q1 | CHEMBL3912 | Lyase | 0.111501865 | 0 / 1 | leucocianidol |
| Hypoxia-inducible factor 1 alpha | HIF1A | Q16665 | CHEMBL4261 | Transcription factor | 0.111501865 | 0 / 7 | leucocianidol |
| Arachidonate 15-lipoxygenase | ALOX15 | P16050 | CHEMBL2903 | Enzyme | 0.111501865 | 0 / 8 | leucocianidol |
| Arachidonate 12-lipoxygenase | ALOX12 | P18054 | CHEMBL3687 | Enzyme | 0.111501865 | 0 / 4 | leucocianidol |
| Cytochrome P450 1B1 | CYP1B1 | Q16678 | CHEMBL4878 | Cytochrome P450 | 0.111501865 | 0 / 6 | leucocianidol |
| Estrogen receptor beta | ESR2 | Q92731 | CHEMBL242 | Nuclear receptor | 0.111501865 | 3 / 87 | leucocianidol |
| Kallikrein 1 | KLK1 | P06870 | CHEMBL2319 | Protease | 0.111501865 | 0 / 1 | leucocianidol |
| Kallikrein 2 | KLK2 | P20151 | CHEMBL2442 | Protease | 0.111501865 | 0 / 1 | leucocianidol |
| Cytochrome P450 19A1 | CYP19A1 | P11511 | CHEMBL1978 | Cytochrome P450 | 0.118883307 | 53 / 162 | Lupenone |
| Nuclear receptor subfamily 1 group I member 3 (by homology) | NR1I3 | Q14994 | CHEMBL5503 | Nuclear receptor | 0.118883307 | 0 / 2 | Lupenone |
| Cyclooxygenase-1 | PTGS1 | P23219 | CHEMBL221 | Oxidoreductase | 0.118883307 | 1 / 2 | Lupenone |
| 11-beta-hydroxysteroid dehydrogenase 1 | HSD11B1 | P28845 | CHEMBL4235 | Enzyme | 0.118883307 | 142 / 20 | Lupenone |
| Steroid 5-alpha-reductase 1 | SRD5A1 | P18405 | CHEMBL1787 | Oxidoreductase | 0.118883307 | 116 / 5 | Lupenone |
| SUMO-activating enzyme | SAE1 UBA2 | Q9UBE0 Q9UBT2 | CHEMBL2095174 | Enzyme | 0.118883307 | 0 / 1 | Lupenone |
| DNA polymerase beta (by homology) | POLB | P06746 | CHEMBL2392 | Enzyme | 0.118883307 | 0 / 5 | Lupenone |
| Aldo-keto reductase family 1 member B10 | AKR1B10 | O60218 | CHEMBL5983 | Enzyme | 0.118883307 | 0 / 7 | Lupenone |
| Protein-tyrosine phosphatase 1B | PTPN1 | P18031 | CHEMBL335 | Phosphatase | 0.118883307 | 5 / 23 | Lupenone |
| Cannabinoid receptor 2 | CNR2 | P34972 | CHEMBL253 | Family A G protein-coupled receptor | 0.118883307 | 55 / 0 | Lupenone |
| Steroid 5-alpha-reductase 2 | SRD5A2 | P31213 | CHEMBL1856 | Oxidoreductase | 0.118883307 | 128 / 6 | Lupenone |
| C-C chemokine receptor type 2 | CCR2 | P41597 | CHEMBL4015 | Family A G protein-coupled receptor | 0.118883307 | 5 / 0 | Lupenone |
| Vanilloid receptor | TRPV1 | Q8NER1 | CHEMBL4794 | Voltage-gated ion channel | 0.118883307 | 93 / 2 | Lupenone |
| Anandamide amidohydrolase | FAAH | O00519 | CHEMBL2243 | Enzyme | 0.118883307 | 26 / 16 | Lupenone |
| Epoxide hydrolase 1 | EPHX1 | P07099 | CHEMBL1968 | Protease | 0.118883307 | 22 / 0 | Lupenone |
| Epoxide hydratase | EPHX2 | P34913 | CHEMBL2409 | Protease | 0.118883307 | 91 / 0 | Lupenone |
| Androgen Receptor | AR | P10275 | CHEMBL1871 | Nuclear receptor | 0.118883307 | 40 / 26 | Lupenone |
| Carbonic anhydrase IV | CA4 | P22748 | CHEMBL3729 | Lyase | 0.118883307 | 0 / 1 | Lupenone |
| 15-hydroxyprostaglandin dehydrogenase [NAD+] | HPGD | P15428 | CHEMBL1293255 | Enzyme | 0 | 4 / 0 | Lupenone |
| Sodium channel protein type IX alpha subunit | SCN9A | Q15858 | CHEMBL4296 | Voltage-gated ion channel | 0 | 10 / 0 | Lupenone |
| Isoprenylcysteine carboxyl methyltransferase | ICMT | O60725 | CHEMBL4699 | Enzyme | 0 | 1 / 0 | Lupenone |
| MAP kinase p38 alpha | MAPK14 | Q16539 | CHEMBL260 | Kinase | 0 | 73 / 0 | Lupenone |
| Poly [ADP-ribose] polymerase-1 | PARP1 | P09874 | CHEMBL3105 | Enzyme | 0 | 16 / 0 | Lupenone |
| Vasopressin V2 receptor | AVPR2 | P30518 | CHEMBL1790 | Family A G protein-coupled receptor | 0 | 22 / 0 | Lupenone |
| Vasopressin V1a receptor | AVPR1A | P37288 | CHEMBL1889 | Family A G protein-coupled receptor | 0 | 25 / 0 | Lupenone |
| Melatonin receptor 1A | MTNR1A | P48039 | CHEMBL1945 | Family A G protein-coupled receptor | 0 | 45 / 0 | Lupenone |
| Mu opioid receptor | OPRM1 | P35372 | CHEMBL233 | Family A G protein-coupled receptor | 0 | 6 / 0 | Lupenone |
| Delta opioid receptor | OPRD1 | P41143 | CHEMBL236 | Family A G protein-coupled receptor | 0 | 13 / 0 | Lupenone |
| Serine/threonine-protein kinase AKT | AKT1 | P31749 | CHEMBL4282 | Kinase | 0 | 4 / 0 | Lupenone |
| Transient receptor potential cation channel subfamily A member 1 | TRPA1 | O75762 | CHEMBL6007 | Voltage-gated ion channel | 0 | 0 / 1 | Lupenone |
| Carboxylesterase 2 | CES2 | O00748 | CHEMBL3180 | Enzyme | 0 | 0 / 13 | Lupenone |
| Monoglyceride lipase | MGLL | Q99685 | CHEMBL4191 | Enzyme | 0 | 3 / 0 | Lupenone |
| 3-keto-steroid reductase | HSD17B7 | P56937 | CHEMBL5999 | Enzyme | 0 | 1 / 0 | Lupenone |
| Translocator protein (by homology) | TSPO | P30536 | CHEMBL5742 | Membrane receptor | 0 | 21 / 0 | Lupenone |
| Geranylgeranyl transferase type I | PGGT1B FNTA | P53609 P49354 | CHEMBL2095164 | Enzyme | 0 | 10 / 0 | Lupenone |
| Calpain 1 | CAPN1 | P07384 | CHEMBL3891 | Protease | 0 | 3 / 0 | Lupenone |
| C-X-C chemokine receptor type 3 | CXCR3 | P49682 | CHEMBL4441 | Family A G protein-coupled receptor | 0 | 13 / 0 | Lupenone |
| Vascular endothelial growth factor receptor 2 | KDR | P35968 | CHEMBL279 | Kinase | 0 | 45 / 0 | Lupenone |
| Metabotropic glutamate receptor 1 | GRM1 | Q13255 | CHEMBL3772 | Family C G protein-coupled receptor | 0 | 12 / 0 | Lupenone |
| Glutamate receptor ionotropic, AMPA 2 | GRIA2 | P42262 | CHEMBL4016 | Ligand-gated ion channel | 0 | 4 / 0 | Lupenone |
| Progesterone receptor | PGR | P06401 | CHEMBL208 | Nuclear receptor | 0 | 47 / 7 | Lupenone |
| 3-beta-hydroxysteroid dehydrogenase/delta 5-->4-isomerase type I | HSD3B1 | P14060 | CHEMBL1958 | Enzyme | 0 | 3 / 0 | Lupenone |
| Sphingosine kinase 2 | SPHK2 | Q9NRA0 | CHEMBL3023 | Enzyme | 0 | 2 / 0 | Lupenone |
| Cytochrome P450 2C19 | CYP2C19 | P33261 | CHEMBL3622 | Cytochrome P450 | 0 | 6 / 0 | Lupenone |
| Acetylcholinesterase | ACHE | P22303 | CHEMBL220 | Hydrolase | 0 | 11 / 0 | Lupenone |
| Cytochrome P450 11B2 | CYP11B2 | P19099 | CHEMBL2722 | Cytochrome P450 | 0 | 12 / 0 | Lupenone |
| HERG | KCNH2 | Q12809 | CHEMBL240 | Voltage-gated ion channel | 0 | 7 / 0 | Lupenone |
| Muscarinic acetylcholine receptor M3 | CHRM3 | P20309 | CHEMBL245 | Family A G protein-coupled receptor | 0 | 10 / 0 | Lupenone |
| Mineralocorticoid receptor | NR3C2 | P08235 | CHEMBL1994 | Nuclear receptor | 0 | 8 / 5 | Lupenone |
| Calcitonin-gene-related peptide receptor, CALCRL/RAMP1 | CALCRL RAMP1 | Q16602 O60894 | CHEMBL2107838 | Other membrane protein | 0 | 2 / 0 | Lupenone |
| CDK8/Cyclin C | CCNC CDK8 | P24863 P49336 | CHEMBL3038474 | Kinase | 0 | 6 / 0 | Lupenone |
| Voltage-gated potassium channel subunit Kv1.3 | KCNA3 | P22001 | CHEMBL4633 | Voltage-gated ion channel | 0 | 14 / 0 | Lupenone |
| Cell division protein kinase 8 | CDK8 | P49336 | CHEMBL5719 | Kinase | 0 | 9 / 0 | Lupenone |
| Bromodomain-containing protein 4 | BRD4 | O60885 | CHEMBL1163125 | Reader | 0 | 10 / 0 | Lupenone |
| Gamma-secretase | PSEN2 PSENEN NCSTN APH1A PSEN1 APH1B | P49810 Q9NZ42 Q92542 Q96BI3 P49768 Q8WW43 | CHEMBL2094135 | Protease | 0 | 11 / 0 | Lupenone |
| Serotonin 6 (5-HT6) receptor | HTR6 | P50406 | CHEMBL3371 | Family A G protein-coupled receptor | 0 | 2 / 0 | Lupenone |
| Bile acid receptor FXR | NR1H4 | Q96RI1 | CHEMBL2047 | Nuclear receptor | 0 | 2 / 8 | Lupenone |
| Voltage-gated calcium channel alpha2/delta subunit 1 | CACNA2D1 | P54289 | CHEMBL1919 | Calcium channel auxiliary subunit alpha2delta family | 0 | 23 / 0 | Lupenone |
| Melatonin receptor 1B | MTNR1B | P49286 | CHEMBL1946 | Family A G protein-coupled receptor | 0 | 53 / 0 | Lupenone |
| Kappa Opioid receptor | OPRK1 | P41145 | CHEMBL237 | Family A G protein-coupled receptor | 0 | 9 / 0 | Lupenone |
| Platelet activating factor receptor | PTAFR | P25105 | CHEMBL250 | Family A G protein-coupled receptor | 0 | 26 / 0 | Lupenone |
| Cathepsin K | CTSK | P43235 | CHEMBL268 | Protease | 0 | 16 / 0 | Lupenone |
| Cathepsin S | CTSS | P25774 | CHEMBL2954 | Protease | 0 | 17 / 0 | Lupenone |
| Signal transducer and activator of transcription 3 | STAT3 | P40763 | CHEMBL4026 | Transcription factor | 0 | 4 / 0 | Lupenone |
| C-C chemokine receptor type 8 | CCR8 | P51685 | CHEMBL4596 | Family A G protein-coupled receptor | 0 | 1 / 0 | Lupenone |
| Orexin receptor 2 | HCRTR2 | O43614 | CHEMBL4792 | Family A G protein-coupled receptor | 0 | 8 / 0 | Lupenone |
| Orexin receptor 1 | HCRTR1 | O43613 | CHEMBL5113 | Family A G protein-coupled receptor | 0 | 6 / 0 | Lupenone |
| C-C chemokine receptor type 5 | CCR5 | P51681 | CHEMBL274 | Family A G protein-coupled receptor | 0 | 2 / 1 | Lupenone |
| Glucocorticoid receptor | NR3C1 | P04150 | CHEMBL2034 | Nuclear receptor | 0 | 27 / 10 | Lupenone |
| Serine/threonine-protein kinase PIM1 | PIM1 | P11309 | CHEMBL2147 | Kinase | 0 | 8 / 0 | Lupenone |
| Serine/threonine-protein kinase PIM3 | PIM3 | Q86V86 | CHEMBL5407 | Kinase | 0 | 6 / 0 | Lupenone |
| Bromodomain-containing protein 2 | BRD2 | P25440 | CHEMBL1293289 | Reader | 0 | 6 / 0 | Lupenone |
| Hexokinase type IV | GCK | P35557 | CHEMBL3820 | Enzyme | 0 | 7 / 0 | Lupenone |
| Kinesin-like protein 1 | KIF11 | P52732 | CHEMBL4581 | Other cytosolic protein | 0 | 3 / 0 | Lupenone |
| Cytochrome P450 2C9 | CYP2C9 | P11712 | CHEMBL3397 | Cytochrome P450 | 0 | 2 / 0 | Lupenone |
| Sodium/glucose cotransporter 1 | SLC5A1 | P13866 | CHEMBL4979 | Electrochemical transporter | 0 | 27 / 0 | Lupenone |
| Aldehyde dehydrogenase | ALDH2 | P05091 | CHEMBL1935 | Oxidoreductase | 0 | 2 / 0 | Lupenone |
| c-Jun N-terminal kinase 3 | MAPK10 | P53779 | CHEMBL2637 | Kinase | 0 | 5 / 0 | Lupenone |
| Adenosine A1 receptor (by homology) | ADORA1 | P30542 | CHEMBL226 | Family A G protein-coupled receptor | 0 | 3 / 0 | Lupenone |
| Acyl coenzyme A:cholesterol acyltransferase 1 | SOAT1 | P35610 | CHEMBL2782 | Enzyme | 0 | 4 / 0 | Lupenone |
| Nicotinamide phosphoribosyltransferase | NAMPT | P43490 | CHEMBL1744525 | Enzyme | 0 | 7 / 0 | Lupenone |
| Lysosomal alpha-glucosidase | GAA | P10253 | CHEMBL2608 | Hydrolase | 0 | 1 / 0 | Lupenone |
| Protein kinase C delta | PRKCD | Q05655 | CHEMBL2996 | Kinase | 0 | 4 / 0 | Lupenone |
| Protein kinase C theta | PRKCQ | Q04759 | CHEMBL3920 | Kinase | 0 | 7 / 0 | Lupenone |
| Neuropeptide Y receptor type 5 | NPY5R | Q15761 | CHEMBL4561 | Family A G protein-coupled receptor | 0 | 16 / 0 | Lupenone |
| Prostaglandin E synthase | PTGES | O14684 | CHEMBL5658 | Enzyme | 0 | 2 / 8 | Lupenone |
| Retinoic acid receptor gamma | RARG | P13631 | CHEMBL2003 | Nuclear receptor | 0 | 1 / 0 | Lupenone |
| Retinoic acid receptor beta | RARB | P10826 | CHEMBL2008 | Nuclear receptor | 0 | 1 / 0 | Lupenone |
| Thrombin | F2 | P00734 | CHEMBL204 | Protease | 0 | 5 / 0 | Lupenone |
| Retinoic acid receptor alpha | RARA | P10276 | CHEMBL2055 | Nuclear receptor | 0 | 1 / 0 | Lupenone |
| Trypsin I | PRSS1 | P07477 | CHEMBL209 | Protease | 0 | 1 / 0 | Lupenone |
| Chymotrypsin C | CTRC | Q99895 | CHEMBL2386 | Protease | 0 | 1 / 0 | Lupenone |
| Adenosine A2a receptor | ADORA2A | P29274 | CHEMBL251 | Family A G protein-coupled receptor | 0 | 2 / 0 | Lupenone |
| N-acylsphingosine-amidohydrolase | NAAA | Q02083 | CHEMBL4349 | Enzyme | 0 | 6 / 0 | Lupenone |
| Retinoid X receptor alpha (by homology) | RXRA | P19793 | CHEMBL2061 | Nuclear receptor | 0 | 1 / 0 | Lupenone |
| Retinoid X receptor gamma (by homology) | RXRG | P48443 | CHEMBL2004 | Nuclear receptor | 0 | 1 / 0 | Lupenone |
| Phospholipase A2 group IIA | PLA2G2A | P14555 | CHEMBL3474 | Enzyme | 0 | 1 / 0 | Lupenone |
| Cytochrome P450 11B1 | CYP11B1 | P15538 | CHEMBL1908 | Cytochrome P450 | 0 | 12 / 0 | Lupenone |
| Estradiol 17-beta-dehydrogenase 2 | HSD17B2 | P37059 | CHEMBL2789 | Enzyme | 0 | 4 / 0 | Lupenone |
| Hypoxia-inducible factor 1 alpha | HIF1A | Q16665 | CHEMBL4261 | Transcription factor | 0 | 5 / 0 | Lupenone |
| 11-beta-hydroxysteroid dehydrogenase 1 | HSD11B1 | P28845 | CHEMBL4235 | Enzyme | 0.191271932 | 11 / 21 | Lupeol |
| UDP-glucuronosyltransferase 2B7 | UGT2B7 | P16662 | CHEMBL4370 | Enzyme | 0.175186649 | 0 / 26 | Lupeol |
| Androgen Receptor | AR | P10275 | CHEMBL1871 | Nuclear receptor | 0.118883307 | 4 / 64 | Lupeol |
| Cyclooxygenase-1 | PTGS1 | P23219 | CHEMBL221 | Oxidoreductase | 0.118883307 | 0 / 2 | Lupeol |
| LXR-alpha | NR1H3 | Q13133 | CHEMBL2808 | Nuclear receptor | 0.118883307 | 3 / 19 | Lupeol |
| Estrogen receptor alpha | ESR1 | P03372 | CHEMBL206 | Nuclear receptor | 0.118883307 | 0 / 35 | Lupeol |
| Muscarinic acetylcholine receptor M2 | CHRM2 | P08172 | CHEMBL211 | Family A G protein-coupled receptor | 0.118883307 | 0 / 1 | Lupeol |
| Acetylcholinesterase | ACHE | P22303 | CHEMBL220 | Hydrolase | 0.118883307 | 0 / 1 | Lupeol |
| Norepinephrine transporter | SLC6A2 | P23975 | CHEMBL222 | Electrochemical transporter | 0.118883307 | 0 / 2 | Lupeol |
| Serotonin transporter | SLC6A4 | P31645 | CHEMBL228 | Electrochemical transporter | 0.118883307 | 0 / 1 | Lupeol |
| Cytochrome P450 2C19 | CYP2C19 | P33261 | CHEMBL3622 | Cytochrome P450 | 0.118883307 | 0 / 1 | Lupeol |
| Nuclear receptor subfamily 1 group I member 3 (by homology) | NR1I3 | Q14994 | CHEMBL5503 | Nuclear receptor | 0.118883307 | 0 / 2 | Lupeol |
| Estrogen receptor beta | ESR2 | Q92731 | CHEMBL242 | Nuclear receptor | 0.118883307 | 0 / 44 | Lupeol |
| Cytochrome P450 19A1 | CYP19A1 | P11511 | CHEMBL1978 | Cytochrome P450 | 0.118883307 | 3 / 49 | Lupeol |
| Dual specificity phosphatase Cdc25A | CDC25A | P30304 | CHEMBL3775 | Phosphatase | 0.118883307 | 3 / 13 | Lupeol |
| Dual specificity phosphatase Cdc25B | CDC25B | P30305 | CHEMBL4804 | Phosphatase | 0.118883307 | 4 / 7 | Lupeol |
| SUMO-activating enzyme | SAE1 UBA2 | Q9UBE0 Q9UBT2 | CHEMBL2095174 | Enzyme | 0.118883307 | 0 / 1 | Lupeol |
| DNA polymerase beta (by homology) | POLB | P06746 | CHEMBL2392 | Enzyme | 0.118883307 | 0 / 6 | Lupeol |
| Prostaglandin E synthase | PTGES | O14684 | CHEMBL5658 | Enzyme | 0.118883307 | 0 / 8 | Lupeol |
| Aldo-keto reductase family 1 member B10 | AKR1B10 | O60218 | CHEMBL5983 | Enzyme | 0.118883307 | 0 / 7 | Lupeol |
| G-protein coupled bile acid receptor 1 | GPBAR1 | Q8TDU6 | CHEMBL5409 | Family A G protein-coupled receptor | 0.118883307 | 0 / 16 | Lupeol |
| Sonic hedgehog protein (by homology) | SHH | Q15465 | CHEMBL5602 | Unclassified protein | 0.118883307 | 0 / 10 | Lupeol |
| Cannabinoid receptor 2 | CNR2 | P34972 | CHEMBL253 | Family A G protein-coupled receptor | 0.118883307 | 18 / 0 | Lupeol |
| Protein-tyrosine phosphatase 1B | PTPN1 | P18031 | CHEMBL335 | Phosphatase | 0.118883307 | 6 / 29 | Lupeol |
| Transient receptor potential cation channel subfamily M member 8 | TRPM8 | Q7Z2W7 | CHEMBL1075319 | Voltage-gated ion channel | 0.118883307 | 0 / 1 | Lupeol |
| Carbonic anhydrase II | CA2 | P00918 | CHEMBL205 | Lyase | 0.118883307 | 0 / 3 | Lupeol |
| Carbonic anhydrase I | CA1 | P00915 | CHEMBL261 | Lyase | 0.118883307 | 0 / 3 | Lupeol |
| Carbonic anhydrase IV | CA4 | P22748 | CHEMBL3729 | Lyase | 0.118883307 | 0 / 2 | Lupeol |
| NADPH oxidase 4 | NOX4 | Q9NPH5 | CHEMBL1250375 | Enzyme | 1 | 6 / 8 | Luteolin |
| Aldose reductase | AKR1B1 | P15121 | CHEMBL1900 | Enzyme | 1 | 17 / 71 | Luteolin |
| Cyclin-dependent kinase 5/CDK5 activator 1 | CDK5R1 CDK5 | Q15078 Q00535 | CHEMBL1907600 | Kinase | 1 | 6 / 18 | Luteolin |
| Xanthine dehydrogenase | XDH | P47989 | CHEMBL1929 | Oxidoreductase | 1 | 12 / 20 | Luteolin |
| Monoamine oxidase A | MAOA | P21397 | CHEMBL1951 | Oxidoreductase | 1 | 5 / 18 | Luteolin |
| Tyrosine-protein kinase receptor FLT3 | FLT3 | P36888 | CHEMBL1974 | Kinase | 1 | 5 / 7 | Luteolin |
| Carbonic anhydrase II | CA2 | P00918 | CHEMBL205 | Lyase | 1 | 8 / 14 | Luteolin |
| Cyclin-dependent kinase 1/cyclin B | CCNB3 CDK1 CCNB1 CCNB2 | Q8WWL7 P06493 P14635 O95067 | CHEMBL2094127 | Other cytosolic protein | 1 | 4 / 11 | Luteolin |
| Arachidonate 5-lipoxygenase | ALOX5 | P09917 | CHEMBL215 | Oxidoreductase | 1 | 5 / 47 | Luteolin |
| Adenosine A1 receptor (by homology) | ADORA1 | P30542 | CHEMBL226 | Family A G protein-coupled receptor | 1 | 6 / 23 | Luteolin |
| Carbonic anhydrase VII | CA7 | P43166 | CHEMBL2326 | Lyase | 1 | 8 / 11 | Luteolin |
| Glyoxalase I | GLO1 | Q04760 | CHEMBL2424 | Enzyme | 1 | 3 / 4 | Luteolin |
| Beta amyloid A4 protein | APP | P05067 | CHEMBL2487 | Membrane receptor | 1 | 2 / 12 | Luteolin |
| Tyrosine-protein kinase SYK | SYK | P43405 | CHEMBL2599 | Kinase | 1 | 3 / 3 | Luteolin |
| Glycogen synthase kinase-3 beta | GSK3B | P49841 | CHEMBL262 | Kinase | 1 | 3 / 7 | Luteolin |
| Poly [ADP-ribose] polymerase-1 | PARP1 | P09874 | CHEMBL3105 | Enzyme | 1 | 3 / 9 | Luteolin |
| Transthyretin | TTR | P02766 | CHEMBL3194 | Secreted protein | 1 | 2 / 2 | Luteolin |
| Matrix metalloproteinase 9 | MMP9 | P14780 | CHEMBL321 | Protease | 1 | 2 / 2 | Luteolin |
| Carbonic anhydrase XII | CA12 | O43570 | CHEMBL3242 | Lyase | 1 | 10 / 15 | Luteolin |
| Matrix metalloproteinase 2 | MMP2 | P08253 | CHEMBL333 | Protease | 1 | 2 / 2 | Luteolin |
| Carbonic anhydrase IV | CA4 | P22748 | CHEMBL3729 | Lyase | 1 | 7 / 10 | Luteolin |
| Matrix metalloproteinase 12 | MMP12 | P39900 | CHEMBL4393 | Protease | 1 | 1 / 1 | Luteolin |
| Lymphocyte differentiation antigen CD38 | CD38 | P28907 | CHEMBL4660 | Enzyme | 1 | 2 / 2 | Luteolin |
| Cytochrome P450 1B1 | CYP1B1 | Q16678 | CHEMBL4878 | Cytochrome P450 | 1 | 12 / 46 | Luteolin |
| ATP-binding cassette sub-family G member 2 | ABCG2 | Q9UNQ0 | CHEMBL5393 | Primary active transporter | 1 | 6 / 49 | Luteolin |
| Aldo-keto reductase family 1 member B10 | AKR1B10 | O60218 | CHEMBL5983 | Enzyme | 1 | 2 / 3 | Luteolin |
| Tankyrase-2 | TNKS2 | Q9H2K2 | CHEMBL6154 | Enzyme | 1 | 4 / 12 | Luteolin |
| Tankyrase-1 | TNKS | O95271 | CHEMBL6164 | Enzyme | 1 | 4 / 28 | Luteolin |
| DNA topoisomerase I (by homology) | TOP1 | P11387 | CHEMBL1781 | Isomerase | 1 | 1 / 1 | Luteolin |
| Arginase-1 (by homology) | ARG1 | P05089 | CHEMBL1075097 | Enzyme | 1 | 2 / 2 | Luteolin |
| Receptor-type tyrosine-protein phosphatase S | PTPRS | Q13332 | CHEMBL2396508 | Phosphatase | 0.857022738 | 7 / 8 | Luteolin |
| Multidrug resistance-associated protein 1 | ABCC1 | P33527 | CHEMBL3004 | Primary active transporter | 0.658019738 | 7 / 11 | Luteolin |
| Estradiol 17-beta-dehydrogenase 1 | HSD17B1 | P14061 | CHEMBL3181 | Enzyme | 0.658019738 | 10 / 4 | Luteolin |
| Acetylcholinesterase | ACHE | P22303 | CHEMBL220 | Hydrolase | 0.6004118 | 4 / 27 | Luteolin |
| Cyclin-dependent kinase 6 | CDK6 | Q00534 | CHEMBL2508 | Kinase | 0.6004118 | 3 / 4 | Luteolin |
| P-glycoprotein 1 | ABCB1 | P08183 | CHEMBL4302 | Primary active transporter | 0.534504957 | 12 / 48 | Luteolin |
| Estradiol 17-beta-dehydrogenase 2 | HSD17B2 | P37059 | CHEMBL2789 | Enzyme | 0.468552736 | 10 / 3 | Luteolin |
| Cytochrome P450 19A1 | CYP19A1 | P11511 | CHEMBL1978 | Cytochrome P450 | 0.460288742 | 6 / 19 | Luteolin |
| Estrogen receptor beta | ESR2 | Q92731 | CHEMBL242 | Nuclear receptor | 0.460288742 | 84 / 36 | Luteolin |
| Adenosine A2a receptor (by homology) | ADORA2A | P29274 | CHEMBL251 | Family A G protein-coupled receptor | 0.460288742 | 5 / 11 | Luteolin |
| Casein kinase II alpha | CSNK2A1 | P68400 | CHEMBL3629 | Kinase | 0.460288742 | 3 / 2 | Luteolin |
| Arachidonate 15-lipoxygenase | ALOX15 | P16050 | CHEMBL2903 | Enzyme | 0.435520295 | 6 / 8 | Luteolin |
| Arachidonate 12-lipoxygenase | ALOX12 | P18054 | CHEMBL3687 | Enzyme | 0.435520295 | 9 / 11 | Luteolin |
| Estrogen receptor alpha | ESR1 | P03372 | CHEMBL206 | Nuclear receptor | 0.386211984 | 66 / 37 | Luteolin |
| Cyclooxygenase-2 | PTGS2 | P35354 | CHEMBL230 | Oxidoreductase | 0.386211984 | 1 / 12 | Luteolin |
| Cystic fibrosis transmembrane conductance regulator | CFTR | P13569 | CHEMBL4051 | Other ion channel | 0.386211984 | 1 / 1 | Luteolin |
| AMY1C | AMY1A | P04745 | CHEMBL2478 | Enzyme | 0.361228277 | 1 / 1 | Luteolin |
| G protein-coupled receptor kinase 6 | GRK6 | P43250 | CHEMBL6144 | Kinase | 0.361228277 | 2 / 4 | Luteolin |
| Carbonic anhydrase I | CA1 | P00915 | CHEMBL261 | Lyase | 0.336739633 | 4 / 6 | Luteolin |
| Carbonic anhydrase IX | CA9 | Q16790 | CHEMBL3594 | Lyase | 0.336739633 | 3 / 6 | Luteolin |
| Cyclin-dependent kinase 2 | CDK2 | P24941 | CHEMBL301 | Kinase | 0.328395696 | 1 / 14 | Luteolin |
| Telomerase reverse transcriptase | TERT | O14746 | CHEMBL2916 | Enzyme | 0.320218721 | 9 / 22 | Luteolin |
| Cyclin-dependent kinase 1 | CDK1 | P06493 | CHEMBL308 | Kinase | 0.303816095 | 3 / 10 | Luteolin |
| Tyrosinase | TYR | P14679 | CHEMBL1973 | Oxidoreductase | 0.287307298 | 2 / 2 | Luteolin |
| Aryl hydrocarbon receptor | AHR | P35869 | CHEMBL3201 | Transcription factor | 0.287307298 | 1 / 1 | Luteolin |
| Estrogen-related receptor alpha | ESRRA | P11474 | CHEMBL3429 | Nuclear receptor | 0.287307298 | 2 / 2 | Luteolin |
| G-protein coupled receptor 35 | GPR35 | Q9HC97 | CHEMBL1293267 | Family A G protein-coupled receptor | 0.279037062 | 2 / 4 | Luteolin |
| Vasopressin V2 receptor | AVPR2 | P30518 | CHEMBL1790 | Family A G protein-coupled receptor | 0.279037062 | 1 / 1 | Luteolin |
| Insulin-like growth factor I receptor | IGF1R | P08069 | CHEMBL1957 | Kinase | 0.279037062 | 3 / 3 | Luteolin |
| Epidermal growth factor receptor erbB1 | EGFR | P00533 | CHEMBL203 | Kinase | 0.279037062 | 6 / 30 | Luteolin |
| Thrombin | F2 | P00734 | CHEMBL204 | Protease | 0.279037062 | 11 / 3 | Luteolin |
| Serine/threonine-protein kinase PIM1 | PIM1 | P11309 | CHEMBL2147 | Kinase | 0.279037062 | 8 / 7 | Luteolin |
| Serine/threonine-protein kinase Aurora-B | AURKB | Q96GD4 | CHEMBL2185 | Kinase | 0.279037062 | 3 / 4 | Luteolin |
| Dopamine D4 receptor | DRD4 | P21917 | CHEMBL219 | Family A G protein-coupled receptor | 0.279037062 | 1 / 1 | Luteolin |
| Myeloperoxidase | MPO | P05164 | CHEMBL2439 | Enzyme | 0.279037062 | 1 / 1 | Luteolin |
| PI3-kinase p85-alpha subunit | PIK3R1 | P27986 | CHEMBL2506 | Enzyme | 0.279037062 | 1 / 1 | Luteolin |
| Death-associated protein kinase 1 | DAPK1 | P53355 | CHEMBL2558 | Kinase | 0.279037062 | 2 / 2 | Luteolin |
| Liver glycogen phosphorylase | PYGL | P06737 | CHEMBL2568 | Enzyme | 0.279037062 | 1 / 1 | Luteolin |
| Tyrosine-protein kinase SRC | SRC | P12931 | CHEMBL267 | Kinase | 0.279037062 | 3 / 10 | Luteolin |
| Focal adhesion kinase 1 | PTK2 | Q05397 | CHEMBL2695 | Kinase | 0.279037062 | 1 / 2 | Luteolin |
| Vascular endothelial growth factor receptor 2 | KDR | P35968 | CHEMBL279 | Kinase | 0.279037062 | 2 / 3 | Luteolin |
| Matrix metalloproteinase 13 | MMP13 | P45452 | CHEMBL280 | Protease | 0.279037062 | 1 / 1 | Luteolin |
| Matrix metalloproteinase 3 | MMP3 | P08254 | CHEMBL283 | Protease | 0.279037062 | 1 / 1 | Luteolin |
| Carbonic anhydrase III | CA3 | P07451 | CHEMBL2885 | Lyase | 0.279037062 | 1 / 1 | Luteolin |
| Serine/threonine-protein kinase PLK1 | PLK1 | P53350 | CHEMBL3024 | Kinase | 0.279037062 | 2 / 3 | Luteolin |
| Carbonic anhydrase VI | CA6 | P23280 | CHEMBL3025 | Lyase | 0.279037062 | 1 / 1 | Luteolin |
| Protein kinase N1 | PKN1 | Q16512 | CHEMBL3384 | Kinase | 0.279037062 | 1 / 3 | Luteolin |
| Carbonic anhydrase XIV | CA14 | Q9ULX7 | CHEMBL3510 | Lyase | 0.279037062 | 1 / 1 | Luteolin |
| Hepatocyte growth factor receptor | MET | P08581 | CHEMBL3717 | Kinase | 0.279037062 | 3 / 4 | Luteolin |
| Serine/threonine-protein kinase NEK2 | NEK2 | P51955 | CHEMBL3835 | Kinase | 0.279037062 | 1 / 2 | Luteolin |
| Interleukin-8 receptor A | CXCR1 | P25024 | CHEMBL4029 | Family A G protein-coupled receptor | 0.279037062 | 1 / 1 | Luteolin |
| CaM kinase II beta | CAMK2B | Q13554 | CHEMBL4121 | Kinase | 0.279037062 | 1 / 2 | Luteolin |
| ALK tyrosine kinase receptor | ALK | Q9UM73 | CHEMBL4247 | Kinase | 0.279037062 | 2 / 4 | Luteolin |
| Serine/threonine-protein kinase AKT | AKT1 | P31749 | CHEMBL4282 | Kinase | 0.279037062 | 1 / 4 | Luteolin |
| Serine/threonine-protein kinase NEK6 | NEK6 | Q9HC98 | CHEMBL4309 | Kinase | 0.279037062 | 1 / 2 | Luteolin |
| Phospholipase A2 group 1B | PLA2G1B | P04054 | CHEMBL4426 | Enzyme | 0.279037062 | 1 / 1 | Luteolin |
| Carbonic anhydrase VA | CA5A | P35218 | CHEMBL4789 | Lyase | 0.279037062 | 1 / 1 | Luteolin |
| Beta-secretase 1 | BACE1 | P56817 | CHEMBL4822 | Protease | 0.279037062 | 5 / 14 | Luteolin |
| Tyrosine-protein kinase receptor UFO | AXL | P30530 | CHEMBL4895 | Kinase | 0.279037062 | 2 / 4 | Luteolin |
| NUAK family SNF1-like kinase 1 | NUAK1 | O60285 | CHEMBL5784 | Kinase | 0.279037062 | 1 / 2 | Luteolin |
| Aldo-keto reductase family 1 member C2 (by homology) | AKR1C2 | P52895 | CHEMBL5847 | Enzyme | 0.279037062 | 1 / 1 | Luteolin |
| Aldo-keto reductase family 1 member C1 (by homology) | AKR1C1 | Q04828 | CHEMBL5905 | Enzyme | 0.279037062 | 1 / 1 | Luteolin |
| Aldo-keto-reductase family 1 member C3 (by homology) | AKR1C3 | P42330 | CHEMBL4681 | Enzyme | 0.279037062 | 1 / 1 | Luteolin |
| Aldo-keto reductase family 1 member C4 (by homology) | AKR1C4 | P17516 | CHEMBL4999 | Enzyme | 0.279037062 | 1 / 1 | Luteolin |
| Carbonic anhydrase XIII (by homology) | CA13 | Q8N1Q1 | CHEMBL3912 | Lyase | 0.279037062 | 1 / 1 | Luteolin |
| Aldehyde reductase (by homology) | AKR1A1 | P14550 | CHEMBL2246 | Enzyme | 0.279037062 | 1 / 1 | Luteolin |
| 6-phosphofructo-2-kinase/fructose-2,6-bisphosphatase 3 | PFKFB3 | Q16875 | CHEMBL2331053 | Enzyme | 0.147256737 | 2 / 2 | Luteolin |
| Plasminogen | PLG | P00747 | CHEMBL1801 | Protease | 0.147256737 | 6 / 3 | Luteolin |
| Lysine-specific demethylase 4D-like | KDM4E | B2RXH2 | CHEMBL1293226 | Eraser | 0.147256737 | 2 / 2 | Luteolin |
| Androgen Receptor | AR | P10275 | CHEMBL1871 | Nuclear receptor | 0.139061947 | 0 / 16 | Luteolin |
| Protein-tyrosine phosphatase 1B | PTPN1 | P18031 | CHEMBL335 | Phosphatase | 0.116965063 | 24 / 9 | Medicarpin |
| Cytochrome P450 19A1 | CYP19A1 | P11511 | CHEMBL1978 | Cytochrome P450 | 0.100578902 | 111 / 44 | Medicarpin |
| Estrogen receptor alpha | ESR1 | P03372 | CHEMBL206 | Nuclear receptor | 0.100578902 | 116 / 137 | Medicarpin |
| Kinesin-1 heavy chain/ Tyrosine-protein kinase receptor RET | RET | P07949 | CHEMBL2041 | Kinase | 0.100578902 | 61 / 0 | Medicarpin |
| Tyrosine-protein kinase JAK3 | JAK3 | P52333 | CHEMBL2148 | Kinase | 0.100578902 | 61 / 0 | Medicarpin |
| Tyrosine-protein kinase LCK | LCK | P06239 | CHEMBL258 | Kinase | 0.100578902 | 65 / 0 | Medicarpin |
| ALK tyrosine kinase receptor | ALK | Q9UM73 | CHEMBL4247 | Kinase | 0.100578902 | 29 / 0 | Medicarpin |
| Serine/threonine-protein kinase PIM1 | PIM1 | P11309 | CHEMBL2147 | Kinase | 0.100578902 | 90 / 0 | Medicarpin |
| Serine/threonine-protein kinase PIM2 | PIM2 | Q9P1W9 | CHEMBL4523 | Kinase | 0.100578902 | 41 / 0 | Medicarpin |
| Estrogen receptor beta | ESR2 | Q92731 | CHEMBL242 | Nuclear receptor | 0.100578902 | 114 / 132 | Medicarpin |
| Vascular endothelial growth factor receptor 2 | KDR | P35968 | CHEMBL279 | Kinase | 0.100578902 | 280 / 3 | Medicarpin |
| Dual specificity mitogen-activated protein kinase kinase 1 | MAP2K1 | Q02750 | CHEMBL3587 | Kinase | 0.100578902 | 16 / 0 | Medicarpin |
| Serine/threonine-protein kinase Chk1 | CHEK1 | O14757 | CHEMBL4630 | Kinase | 0.100578902 | 98 / 0 | Medicarpin |
| Serine/threonine-protein kinase WEE1 | WEE1 | P30291 | CHEMBL5491 | Kinase | 0.100578902 | 49 / 0 | Medicarpin |
| Arachidonate 15-lipoxygenase | ALOX15 | P16050 | CHEMBL2903 | Enzyme | 0.100578902 | 14 / 8 | Medicarpin |
| Glycogen synthase kinase-3 beta | GSK3B | P49841 | CHEMBL262 | Kinase | 0.100578902 | 186 / 0 | Medicarpin |
| Stem cell growth factor receptor | KIT | P10721 | CHEMBL1936 | Kinase | 0.100578902 | 47 / 3 | Medicarpin |
| Phosphodiesterase 4D | PDE4D | Q08499 | CHEMBL288 | Phosphodiesterase | 0.100578902 | 23 / 0 | Medicarpin |
| MAP kinase-interacting serine/threonine-protein kinase MNK1 | MKNK1 | Q9BUB5 | CHEMBL4718 | Kinase | 0.100578902 | 11 / 0 | Medicarpin |
| Estradiol 17-beta-dehydrogenase 3 | HSD17B3 | P37058 | CHEMBL4234 | Enzyme | 0.100578902 | 24 / 0 | Medicarpin |
| Matrix metalloproteinase 1 | MMP1 | P03956 | CHEMBL332 | Protease | 0.100578902 | 342 / 0 | Medicarpin |
| Ribosomal protein S6 kinase alpha 2 | RPS6KA2 | Q15349 | CHEMBL3906 | Kinase | 0.100578902 | 5 / 0 | Medicarpin |
| Macrophage migration inhibitory factor | MIF | P14174 | CHEMBL2085 | Enzyme | 0.100578902 | 26 / 0 | Medicarpin |
| MAP kinase-activated protein kinase 2 | MAPKAPK2 | P49137 | CHEMBL2208 | Kinase | 0.100578902 | 55 / 0 | Medicarpin |
| Poly [ADP-ribose] polymerase-1 | PARP1 | P09874 | CHEMBL3105 | Enzyme | 0.100578902 | 133 / 0 | Medicarpin |
| Eukaryotic initiation factor 4A-I | EIF4A1 | P60842 | CHEMBL2052028 | Hydrolase | 0.100578902 | 0 / 29 | Medicarpin |
| Cyclooxygenase-1 | PTGS1 | P23219 | CHEMBL221 | Oxidoreductase | 0.100578902 | 17 / 14 | Medicarpin |
| Catechol O-methyltransferase | COMT | P21964 | CHEMBL2023 | Transferase | 0.100578902 | 12 / 0 | Medicarpin |
| Huntingtin | HTT | P42858 | CHEMBL5514 | Unclassified protein | 0.100578902 | 3 / 0 | Medicarpin |
| Transitional endoplasmic reticulum ATPase | VCP | P55072 | CHEMBL1075145 | Primary active transporter | 0.100578902 | 15 / 0 | Medicarpin |
| Estradiol 17-beta-dehydrogenase 2 | HSD17B2 | P37059 | CHEMBL2789 | Enzyme | 0.100578902 | 51 / 0 | Medicarpin |
| Cytochrome P450 11B1 | CYP11B1 | P15538 | CHEMBL1908 | Cytochrome P450 | 0.100578902 | 40 / 0 | Medicarpin |
| Histone deacetylase 2 | HDAC2 | Q92769 | CHEMBL1937 | Eraser | 0.100578902 | 108 / 0 | Medicarpin |
| Cyclin-dependent kinase 4/cyclin D | CCND3 CCND1 CDK4 CCND2 | P30281 P24385 P11802 P30279 | CHEMBL2095942 | Other cytosolic protein | 0.100578902 | 5 / 0 | Medicarpin |
| Cytochrome P450 11B2 | CYP11B2 | P19099 | CHEMBL2722 | Cytochrome P450 | 0.100578902 | 42 / 0 | Medicarpin |
| Cytochrome P450 17A1 | CYP17A1 | P05093 | CHEMBL3522 | Cytochrome P450 | 0.100578902 | 8 / 0 | Medicarpin |
| Serine/threonine-protein kinase/endoribonuclease IRE1 | ERN1 | O75460 | CHEMBL1163101 | Enzyme | 0.100578902 | 71 / 0 | Medicarpin |
| D-amino-acid oxidase | DAO | P14920 | CHEMBL5485 | Enzyme | 0.100578902 | 12 / 0 | Medicarpin |
| Fibroblast growth factor receptor 1 | FGFR1 | P11362 | CHEMBL3650 | Kinase | 0.100578902 | 34 / 3 | Medicarpin |
| Cystinyl aminopeptidase | LNPEP | Q9UIQ6 | CHEMBL2693 | Protease | 0.100578902 | 21 / 0 | Medicarpin |
| Carbonic anhydrase XIV | CA14 | Q9ULX7 | CHEMBL3510 | Lyase | 0.100578902 | 30 / 0 | Medicarpin |
| Arachidonate 12-lipoxygenase | ALOX12 | P18054 | CHEMBL3687 | Enzyme | 0.100578902 | 5 / 4 | Medicarpin |
| Matrix metalloproteinase 3 | MMP3 | P08254 | CHEMBL283 | Protease | 0.100578902 | 195 / 0 | Medicarpin |
| Thymidylate synthase | TYMS | P04818 | CHEMBL1952 | Transferase | 0.100578902 | 35 / 0 | Medicarpin |
| Serine/threonine-protein kinase RAF | RAF1 | P04049 | CHEMBL1906 | Kinase | 0.100578902 | 66 / 0 | Medicarpin |
| Caspase-3 | CASP3 | P42574 | CHEMBL2334 | Protease | 0.100578902 | 19 / 0 | Medicarpin |
| Caspase-7 | CASP7 | P55210 | CHEMBL3468 | Protease | 0.100578902 | 10 / 0 | Medicarpin |
| Hepatocyte growth factor receptor | MET | P08581 | CHEMBL3717 | Kinase | 0.100578902 | 220 / 4 | Medicarpin |
| Carbonic anhydrase II | CA2 | P00918 | CHEMBL205 | Lyase | 0.100578902 | 279 / 2 | Medicarpin |
| Serine/threonine-protein kinase mTOR | MTOR | P42345 | CHEMBL2842 | Kinase | 0.100578902 | 156 / 0 | Medicarpin |
| PI3-kinase p110-delta subunit | PIK3CD | O00329 | CHEMBL3130 | Enzyme | 0.100578902 | 170 / 0 | Medicarpin |
| PI3-kinase p110-beta subunit | PIK3CB | P42338 | CHEMBL3145 | Enzyme | 0.100578902 | 119 / 0 | Medicarpin |
| PI3-kinase p110-gamma subunit | PIK3CG | P48736 | CHEMBL3267 | Enzyme | 0.100578902 | 161 / 0 | Medicarpin |
| Glutathione S-transferase Pi | GSTP1 | P09211 | CHEMBL3902 | Enzyme | 0.100578902 | 9 / 0 | Medicarpin |
| PI3-kinase p110-alpha subunit | PIK3CA | P42336 | CHEMBL4005 | Enzyme | 0.100578902 | 250 / 0 | Medicarpin |
| Glutathione S-transferase Mu 2 | GSTM2 | P28161 | CHEMBL4589 | Enzyme | 0.100578902 | 9 / 0 | Medicarpin |
| Tyrosine-protein kinase ABL | ABL1 | P00519 | CHEMBL1862 | Kinase | 0.100578902 | 101 / 0 | Medicarpin |
| Phosphodiesterase 4A | PDE4A | P27815 | CHEMBL254 | Phosphodiesterase | 0.100578902 | 23 / 0 | Medicarpin |
| Phosphodiesterase 4B | PDE4B | Q07343 | CHEMBL275 | Phosphodiesterase | 0.100578902 | 72 / 0 | Medicarpin |
| Phosphodiesterase 4C | PDE4C | Q08493 | CHEMBL291 | Phosphodiesterase | 0.100578902 | 4 / 0 | Medicarpin |
| DNA-dependent protein kinase | PRKDC | P78527 | CHEMBL3142 | Kinase | 0.100578902 | 66 / 0 | Medicarpin |
| Tyrosine-protein kinase HCK | HCK | P08631 | CHEMBL3234 | Kinase | 0.100578902 | 51 / 0 | Medicarpin |
| Adenosine A2b receptor | ADORA2B | P29275 | CHEMBL255 | Family A G protein-coupled receptor | 0.100578902 | 43 / 0 | Medicarpin |
| Tyrosine-protein kinase SRC | SRC | P12931 | CHEMBL267 | Kinase | 0.100578902 | 125 / 3 | Medicarpin |
| ADAM17 | ADAM17 | P78536 | CHEMBL3706 | Protease | 0.100578902 | 159 / 0 | Medicarpin |
| Matrix metalloproteinase 8 | MMP8 | P22894 | CHEMBL4588 | Protease | 0.100578902 | 137 / 0 | Medicarpin |
| Carbonic anhydrase I | CA1 | P00915 | CHEMBL261 | Lyase | 0.100578902 | 224 / 2 | Medicarpin |
| Carbonic anhydrase XII | CA12 | O43570 | CHEMBL3242 | Lyase | 0.100578902 | 141 / 7 | Medicarpin |
| Carbonic anhydrase IX | CA9 | Q16790 | CHEMBL3594 | Lyase | 0.100578902 | 163 / 1 | Medicarpin |
| Carbonic anhydrase IV | CA4 | P22748 | CHEMBL3729 | Lyase | 0.100578902 | 17 / 7 | Medicarpin |
| Carbonic anhydrase VB | CA5B | Q9Y2D0 | CHEMBL3969 | Lyase | 0.100578902 | 8 / 2 | Medicarpin |
| Carbonic anhydrase VA | CA5A | P35218 | CHEMBL4789 | Lyase | 0.100578902 | 10 / 2 | Medicarpin |
| Alkaline phosphatase, tissue-nonspecific isozyme | ALPL | P05186 | CHEMBL5979 | Enzyme | 0.100578902 | 23 / 0 | Medicarpin |
| Tubulin beta-1 chain | TUBB1 | Q9H4B7 | CHEMBL1915 | Structural protein | 0.100578902 | 14 / 0 | Medicarpin |
| Adenosine A2a receptor (by homology) | ADORA2A | P29274 | CHEMBL251 | Family A G protein-coupled receptor | 0.100578902 | 109 / 0 | Medicarpin |
| Tubulin beta-3 chain | TUBB3 | Q13509 | CHEMBL2597 | Structural protein | 0.100578902 | 2 / 0 | Medicarpin |
| Cyclin-dependent kinase 2 | CDK2 | P24941 | CHEMBL301 | Kinase | 0.100578902 | 112 / 0 | Medicarpin |
| Cyclin-dependent kinase 4 | CDK4 | P11802 | CHEMBL331 | Kinase | 0.100578902 | 48 / 0 | Medicarpin |
| Tankyrase-2 | TNKS2 | Q9H2K2 | CHEMBL6154 | Enzyme | 0.100578902 | 15 / 0 | Medicarpin |
| Tankyrase-1 | TNKS | O95271 | CHEMBL6164 | Enzyme | 0.100578902 | 14 / 0 | Medicarpin |
| Matrix metalloproteinase 7 | MMP7 | P09237 | CHEMBL4073 | Protease | 0.100578902 | 69 / 0 | Medicarpin |
| Methyl-CpG-binding domain protein 2 | MBD2 | Q9UBB5 | CHEMBL3707462 | Reader | 0.100578902 | 2 / 0 | Medicarpin |
| P-glycoprotein 1 | ABCB1 | P08183 | CHEMBL4302 | Primary active transporter | 0.100578902 | 27 / 68 | Medicarpin |
| Cyclin-dependent kinase 5/CDK5 activator 1 | CDK5R1 CDK5 | Q15078 Q00535 | CHEMBL1907600 | Kinase | 0.100578902 | 65 / 0 | Medicarpin |
| Dual specificty protein kinase CLK1 (by homology) | CLK1 | P49759 | CHEMBL4224 | Kinase | 0.100578902 | 66 / 0 | Medicarpin |
| Dual specificity protein kinase CLK3 (by homology) | CLK3 | P49761 | CHEMBL4226 | Kinase | 0.100578902 | 25 / 0 | Medicarpin |
| Dual-specificity tyrosine-phosphorylation regulated kinase 2 | DYRK2 | Q92630 | CHEMBL4376 | Kinase | 0.100578902 | 25 / 0 | Medicarpin |
| Diacylglycerol O-acyltransferase 1 | DGAT1 | O75907 | CHEMBL6009 | Enzyme | 0.100578902 | 2 / 0 | Medicarpin |
| PI3-kinase p110-alpha/p85-alpha | PIK3CA PIK3R1 | P42336 P27986 | CHEMBL2111367 | Enzyme | 0.100578902 | 6 / 0 | Medicarpin |
| Serine/threonine-protein kinase PLK1 | PLK1 | P53350 | CHEMBL3024 | Kinase | 0.100578902 | 69 / 0 | Medicarpin |
| Interleukin-1 receptor-associated kinase 4 | IRAK4 | Q9NWZ3 | CHEMBL3778 | Kinase | 0.100578902 | 24 / 0 | Medicarpin |
| Serine/threonine-protein kinase Aurora-A | AURKA | O14965 | CHEMBL4722 | Kinase | 0.100578902 | 131 / 0 | Medicarpin |
| Ribosomal protein S6 kinase alpha 5 | RPS6KA5 | O75582 | CHEMBL4237 | Kinase | 0.100578902 | 9 / 0 | Medicarpin |
| Monoamine oxidase B | MAOB | P27338 | CHEMBL2039 | Oxidoreductase | 0.100578902 | 112 / 55 | Medicarpin |
| Matrix metalloproteinase 9 | MMP9 | P14780 | CHEMBL321 | Protease | 0.100578902 | 339 / 1 | Medicarpin |
| Matrix metalloproteinase 2 | MMP2 | P08253 | CHEMBL333 | Protease | 0.100578902 | 281 / 4 | Medicarpin |
| Glutamate receptor ionotropic, AMPA 1 | GRIA1 | P42261 | CHEMBL2009 | Ligand-gated ion channel | 0.100578902 | 8 / 0 | Medicarpin |
| Tyrosine-protein kinase SYK | SYK | P43405 | CHEMBL2599 | Kinase | 0.100578902 | 107 / 0 | Medicarpin |
| Rho-associated protein kinase 2 | ROCK2 | O75116 | CHEMBL2973 | Kinase | 0.100578902 | 38 / 0 | Medicarpin |
| Alkaline phosphatase placental-like | ALPG | P10696 | CHEMBL3402 | Enzyme | 0.100578902 | 5 / 0 | Medicarpin |
| Receptor-type tyrosine-protein phosphatase S | PTPRS | Q13332 | CHEMBL2396508 | Phosphatase | 0.340526084 | 0 / 8 | Morin |
| Estrogen receptor beta | ESR2 | Q92731 | CHEMBL242 | Nuclear receptor | 0.340526084 | 0 / 28 | Morin |
| Death-associated protein kinase 1 | DAPK1 | P53355 | CHEMBL2558 | Kinase | 0.340526084 | 0 / 2 | Morin |
| DNA-3-methyladenine glycosylase | MPG | P29372 | CHEMBL3396943 | Enzyme | 0.340526084 | 0 / 1 | Morin |
| Solute carrier family 22 member 12 | SLC22A12 | Q96S37 | CHEMBL6120 | Electrochemical transporter | 0.340526084 | 0 / 1 | Morin |
| NADPH oxidase 4 | NOX4 | Q9NPH5 | CHEMBL1250375 | Enzyme | 0.214178827 | 0 / 8 | Morin |
| Xanthine dehydrogenase | XDH | P47989 | CHEMBL1929 | Oxidoreductase | 0.214178827 | 0 / 20 | Morin |
| Tyrosinase | TYR | P14679 | CHEMBL1973 | Oxidoreductase | 0.214178827 | 0 / 3 | Morin |
| Tyrosine-protein kinase receptor FLT3 | FLT3 | P36888 | CHEMBL1974 | Kinase | 0.214178827 | 0 / 7 | Morin |
| Arachidonate 5-lipoxygenase | ALOX5 | P09917 | CHEMBL215 | Oxidoreductase | 0.214178827 | 0 / 46 | Morin |
| Estradiol 17-beta-dehydrogenase 2 | HSD17B2 | P37059 | CHEMBL2789 | Enzyme | 0.214178827 | 0 / 3 | Morin |
| Multidrug resistance-associated protein 1 | ABCC1 | P33527 | CHEMBL3004 | Primary active transporter | 0.214178827 | 0 / 11 | Morin |
| Estradiol 17-beta-dehydrogenase 1 | HSD17B1 | P14061 | CHEMBL3181 | Enzyme | 0.214178827 | 0 / 4 | Morin |
| Aryl hydrocarbon receptor | AHR | P35869 | CHEMBL3201 | Transcription factor | 0.214178827 | 0 / 1 | Morin |
| Estrogen-related receptor alpha | ESRRA | P11474 | CHEMBL3429 | Nuclear receptor | 0.214178827 | 0 / 2 | Morin |
| P-glycoprotein 1 | ABCB1 | P08183 | CHEMBL4302 | Primary active transporter | 0.214178827 | 0 / 47 | Morin |
| Cytochrome P450 1B1 | CYP1B1 | Q16678 | CHEMBL4878 | Cytochrome P450 | 0.214178827 | 0 / 46 | Morin |
| ATP-binding cassette sub-family G member 2 | ABCG2 | Q9UNQ0 | CHEMBL5393 | Primary active transporter | 0.214178827 | 0 / 48 | Morin |
| Butyrylcholinesterase | BCHE | P06276 | CHEMBL1914 | Hydrolase | 0.166798772 | 0 / 5 | Morin |
| Acetylcholinesterase | ACHE | P22303 | CHEMBL220 | Hydrolase | 0.166798772 | 0 / 27 | Morin |
| Adenosine A1 receptor (by homology) | ADORA1 | P30542 | CHEMBL226 | Family A G protein-coupled receptor | 0.166798772 | 0 / 23 | Morin |
| Adenosine A2a receptor (by homology) | ADORA2A | P29274 | CHEMBL251 | Family A G protein-coupled receptor | 0.166798772 | 0 / 11 | Morin |
| Adenosine A3 receptor | ADORA3 | P0DMS8 | CHEMBL256 | Family A G protein-coupled receptor | 0.166798772 | 0 / 20 | Morin |
| G-protein coupled receptor 35 | GPR35 | Q9HC97 | CHEMBL1293267 | Family A G protein-coupled receptor | 0.166798772 | 1 / 4 | Morin |
| Arachidonate 15-lipoxygenase | ALOX15 | P16050 | CHEMBL2903 | Enzyme | 0.143102156 | 0 / 8 | Morin |
| Microtubule-associated protein tau | MAPT | P10636 | CHEMBL1293224 | Unclassified protein | 0.143102156 | 0 / 1 | Morin |
| Lysine-specific demethylase 4D-like | KDM4E | B2RXH2 | CHEMBL1293226 | Eraser | 0.143102156 | 0 / 2 | Morin |
| Vasopressin V2 receptor | AVPR2 | P30518 | CHEMBL1790 | Family A G protein-coupled receptor | 0.143102156 | 0 / 1 | Morin |
| DNA topoisomerase II alpha | TOP2A | P11388 | CHEMBL1806 | Isomerase | 0.143102156 | 0 / 1 | Morin |
| Monoamine oxidase A | MAOA | P21397 | CHEMBL1951 | Oxidoreductase | 0.143102156 | 0 / 14 | Morin |
| Cytochrome P450 19A1 | CYP19A1 | P11511 | CHEMBL1978 | Cytochrome P450 | 0.143102156 | 0 / 16 | Morin |
| Serine/threonine-protein kinase PIM1 | PIM1 | P11309 | CHEMBL2147 | Kinase | 0.143102156 | 0 / 7 | Morin |
| Dopamine D4 receptor | DRD4 | P21917 | CHEMBL219 | Family A G protein-coupled receptor | 0.143102156 | 0 / 1 | Morin |
| Glyoxalase I | GLO1 | Q04760 | CHEMBL2424 | Enzyme | 0.143102156 | 0 / 4 | Morin |
| Myosin light chain kinase, smooth muscle | MYLK | Q15746 | CHEMBL2428 | Kinase | 0.143102156 | 0 / 1 | Morin |
| Myeloperoxidase | MPO | P05164 | CHEMBL2439 | Enzyme | 0.143102156 | 0 / 1 | Morin |
| PI3-kinase p85-alpha subunit | PIK3R1 | P27986 | CHEMBL2506 | Enzyme | 0.143102156 | 0 / 1 | Morin |
| Liver glycogen phosphorylase | PYGL | P06737 | CHEMBL2568 | Enzyme | 0.143102156 | 0 / 1 | Morin |
| Tyrosine-protein kinase SYK | SYK | P43405 | CHEMBL2599 | Kinase | 0.143102156 | 0 / 3 | Morin |
| Matrix metalloproteinase 13 | MMP13 | P45452 | CHEMBL280 | Protease | 0.143102156 | 0 / 1 | Morin |
| Matrix metalloproteinase 3 | MMP3 | P08254 | CHEMBL283 | Protease | 0.143102156 | 0 / 1 | Morin |
| Carbonic anhydrase III | CA3 | P07451 | CHEMBL2885 | Lyase | 0.143102156 | 0 / 1 | Morin |
| Cyclin-dependent kinase 1 | CDK1 | P06493 | CHEMBL308 | Kinase | 0.143102156 | 0 / 11 | Morin |
| Matrix metalloproteinase 9 | MMP9 | P14780 | CHEMBL321 | Protease | 0.143102156 | 0 / 2 | Morin |
| PI3-kinase p110-gamma subunit | PIK3CG | P48736 | CHEMBL3267 | Enzyme | 0.143102156 | 0 / 1 | Morin |
| Matrix metalloproteinase 2 | MMP2 | P08253 | CHEMBL333 | Protease | 0.143102156 | 0 / 2 | Morin |
| Protein kinase N1 | PKN1 | Q16512 | CHEMBL3384 | Kinase | 0.143102156 | 0 / 3 | Morin |
| Serine/threonine-protein kinase NEK2 | NEK2 | P51955 | CHEMBL3835 | Kinase | 0.143102156 | 0 / 2 | Morin |
| Interleukin-8 receptor A | CXCR1 | P25024 | CHEMBL4029 | Family A G protein-coupled receptor | 0.143102156 | 0 / 1 | Morin |
| CaM kinase II beta | CAMK2B | Q13554 | CHEMBL4121 | Kinase | 0.143102156 | 0 / 2 | Morin |
| ALK tyrosine kinase receptor | ALK | Q9UM73 | CHEMBL4247 | Kinase | 0.143102156 | 0 / 4 | Morin |
| Serine/threonine-protein kinase NEK6 | NEK6 | Q9HC98 | CHEMBL4309 | Kinase | 0.143102156 | 0 / 2 | Morin |
| Phospholipase A2 group 1B | PLA2G1B | P04054 | CHEMBL4426 | Enzyme | 0.143102156 | 0 / 1 | Morin |
| Tyrosine-protein kinase receptor UFO | AXL | P30530 | CHEMBL4895 | Kinase | 0.143102156 | 0 / 4 | Morin |
| DNA-(apurinic or apyrimidinic site) lyase | APEX1 | P27695 | CHEMBL5619 | Enzyme | 0.143102156 | 0 / 1 | Morin |
| Aldo-keto reductase family 1 member C2 (by homology) | AKR1C2 | P52895 | CHEMBL5847 | Enzyme | 0.143102156 | 0 / 1 | Morin |
| Aldo-keto reductase family 1 member C1 (by homology) | AKR1C1 | Q04828 | CHEMBL5905 | Enzyme | 0.143102156 | 0 / 1 | Morin |
| Aldo-keto-reductase family 1 member C3 (by homology) | AKR1C3 | P42330 | CHEMBL4681 | Enzyme | 0.143102156 | 0 / 1 | Morin |
| Aldo-keto reductase family 1 member C4 (by homology) | AKR1C4 | P17516 | CHEMBL4999 | Enzyme | 0.143102156 | 0 / 1 | Morin |
| Aldehyde reductase (by homology) | AKR1A1 | P14550 | CHEMBL2246 | Enzyme | 0.143102156 | 0 / 1 | Morin |
| Aldose reductase | AKR1B1 | P15121 | CHEMBL1900 | Enzyme | 0.12730257 | 1 / 71 | Morin |
| Carbonic anhydrase II | CA2 | P00918 | CHEMBL205 | Lyase | 0.12730257 | 1 / 13 | Morin |
| Carbonic anhydrase VII | CA7 | P43166 | CHEMBL2326 | Lyase | 0.12730257 | 1 / 12 | Morin |
| Carbonic anhydrase XII | CA12 | O43570 | CHEMBL3242 | Lyase | 0.12730257 | 1 / 16 | Morin |
| 6-phosphofructo-2-kinase/fructose-2,6-bisphosphatase 3 | PFKFB3 | Q16875 | CHEMBL2331053 | Enzyme | 0.12730257 | 0 / 2 | Morin |
| Cyclin-dependent kinase 5/CDK5 activator 1 | CDK5R1 CDK5 | Q15078 Q00535 | CHEMBL1907600 | Kinase | 0.12730257 | 0 / 18 | Morin |
| Cyclin-dependent kinase 1/cyclin B | CCNB3 CDK1 CCNB1 CCNB2 | Q8WWL7 P06493 P14635 O95067 | CHEMBL2094127 | Other cytosolic protein | 0.12730257 | 0 / 9 | Morin |
| Cyclin-dependent kinase 6 | CDK6 | Q00534 | CHEMBL2508 | Kinase | 0.12730257 | 0 / 4 | Morin |
| Cyclin-dependent kinase 2 | CDK2 | P24941 | CHEMBL301 | Kinase | 0.12730257 | 0 / 15 | Morin |
| Arginase-1 (by homology) | ARG1 | P05089 | CHEMBL1075097 | Enzyme | 0.12730257 | 0 / 2 | Morin |
| NEDD8-activating enzyme E1 regulatory subunit | NAE1 | Q13564 | CHEMBL2016431 | Unclassified protein | 0.119403562 | 0 / 1 | Morin |
| Estrogen receptor alpha | ESR1 | P03372 | CHEMBL206 | Nuclear receptor | 0.119403562 | 0 / 29 | Morin |
| Cyclooxygenase-2 | PTGS2 | P35354 | CHEMBL230 | Oxidoreductase | 0.119403562 | 0 / 20 | Morin |
| Transthyretin | TTR | P02766 | CHEMBL3194 | Secreted protein | 0.119403562 | 0 / 2 | Morin |
| Cystic fibrosis transmembrane conductance regulator | CFTR | P13569 | CHEMBL4051 | Other ion channel | 0.119403562 | 0 / 1 | Morin |
| Aldo-keto reductase family 1 member B10 | AKR1B10 | O60218 | CHEMBL5983 | Enzyme | 0.119403562 | 0 / 3 | Morin |
| Tankyrase-2 | TNKS2 | Q9H2K2 | CHEMBL6154 | Enzyme | 0.119403562 | 0 / 12 | Morin |
| Tankyrase-1 | TNKS | O95271 | CHEMBL6164 | Enzyme | 0.119403562 | 0 / 28 | Morin |
| Carbonic anhydrase IV | CA4 | P22748 | CHEMBL3729 | Lyase | 0.119403562 | 1 / 11 | Morin |
| Induced myeloid leukemia cell differentiation protein Mcl-1 | MCL1 | Q07820 | CHEMBL4361 | Other cytosolic protein | 0.119403562 | 0 / 4 | Morin |
| AMY1C | AMY1A | P04745 | CHEMBL2478 | Enzyme | 0.119403562 | 0 / 1 | Morin |
| G protein-coupled receptor kinase 6 | GRK6 | P43250 | CHEMBL6144 | Kinase | 0.119403562 | 0 / 4 | Morin |
| Thrombin | F2 | P00734 | CHEMBL204 | Protease | 0.119403562 | 1 / 3 | Morin |
| Arachidonate 12-lipoxygenase | ALOX12 | P18054 | CHEMBL3687 | Enzyme | 0.119403562 | 1 / 10 | Morin |
| Androgen Receptor | AR | P10275 | CHEMBL1871 | Nuclear receptor | 0.111501865 | 0 / 16 | Morin |
| Carbonyl reductase [NADPH] 1 | CBR1 | P16152 | CHEMBL5586 | Enzyme | 0.111501865 | 0 / 2 | Morin |
| Telomerase reverse transcriptase | TERT | O14746 | CHEMBL2916 | Enzyme | 0.111501865 | 0 / 22 | Morin |
| Insulin-like growth factor I receptor | IGF1R | P08069 | CHEMBL1957 | Kinase | 0.111501865 | 1 / 3 | Morin |
| Insulin receptor | INSR | P06213 | CHEMBL1981 | Kinase | 0.111501865 | 1 / 1 | Morin |
| Epidermal growth factor receptor erbB1 | EGFR | P00533 | CHEMBL203 | Kinase | 0.111501865 | 1 / 28 | Morin |
| Serine/threonine-protein kinase Aurora-B | AURKB | Q96GD4 | CHEMBL2185 | Kinase | 0.111501865 | 1 / 4 | Morin |
| Carbonic anhydrase I | CA1 | P00915 | CHEMBL261 | Lyase | 0.111501865 | 1 / 5 | Morin |
| Glycogen synthase kinase-3 beta | GSK3B | P49841 | CHEMBL262 | Kinase | 0.111501865 | 1 / 5 | Morin |
| Tyrosine-protein kinase SRC | SRC | P12931 | CHEMBL267 | Kinase | 0.111501865 | 1 / 10 | Morin |
| Focal adhesion kinase 1 | PTK2 | Q05397 | CHEMBL2695 | Kinase | 0.111501865 | 1 / 2 | Morin |
| Vascular endothelial growth factor receptor 2 | KDR | P35968 | CHEMBL279 | Kinase | 0.111501865 | 1 / 3 | Morin |
| Serine/threonine-protein kinase PLK1 | PLK1 | P53350 | CHEMBL3024 | Kinase | 0.111501865 | 1 / 3 | Morin |
| Carbonic anhydrase VI | CA6 | P23280 | CHEMBL3025 | Lyase | 0.111501865 | 1 / 1 | Morin |
| Carbonic anhydrase XIV | CA14 | Q9ULX7 | CHEMBL3510 | Lyase | 0.111501865 | 1 / 1 | Morin |
| Carbonic anhydrase IX | CA9 | Q16790 | CHEMBL3594 | Lyase | 0.111501865 | 1 / 6 | Morin |
| Estrogen receptor beta | ESR2 | Q92731 | CHEMBL242 | Nuclear receptor | 0.542515762 | 44 / 54 | Prunetin |
| Aldehyde dehydrogenase | ALDH2 | P05091 | CHEMBL1935 | Oxidoreductase | 0.320218721 | 0 / 42 | Prunetin |
| Carbonyl reductase [NADPH] 1 | CBR1 | P16152 | CHEMBL5586 | Enzyme | 0.287307298 | 2 / 3 | Prunetin |
| Adenosine A1 receptor (by homology) | ADORA1 | P30542 | CHEMBL226 | Family A G protein-coupled receptor | 0.262581506 | 3 / 21 | Prunetin |
| Adenosine A2a receptor | ADORA2A | P29274 | CHEMBL251 | Family A G protein-coupled receptor | 0.262581506 | 2 / 14 | Prunetin |
| Estradiol 17-beta-dehydrogenase 2 | HSD17B2 | P37059 | CHEMBL2789 | Enzyme | 0.229685699 | 6 / 3 | Prunetin |
| Carbonic anhydrase XII | CA12 | O43570 | CHEMBL3242 | Lyase | 0.213125923 | 8 / 33 | Prunetin |
| Estrogen receptor alpha | ESR1 | P03372 | CHEMBL206 | Nuclear receptor | 0.180252494 | 27 / 67 | Prunetin |
| Epidermal growth factor receptor erbB1 | EGFR | P00533 | CHEMBL203 | Kinase | 0.180252494 | 4 / 26 | Prunetin |
| Interleukin-2 | IL2 | P60568 | CHEMBL5880 | Secreted protein | 0.171978589 | 0 / 4 | Prunetin |
| Estradiol 17-beta-dehydrogenase 1 | HSD17B1 | P14061 | CHEMBL3181 | Enzyme | 0.155528102 | 7 / 4 | Prunetin |
| Thromboxane-A synthase | TBXAS1 | P24557 | CHEMBL1835 | Cytochrome P450 | 0.139061947 | 0 / 1 | Prunetin |
| Maltase-glucoamylase | MGAM | O43451 | CHEMBL2074 | Hydrolase | 0.139061947 | 0 / 1 | Prunetin |
| Serotonin 2a (5-HT2a) receptor | HTR2A | P28223 | CHEMBL224 | Family A G protein-coupled receptor | 0.139061947 | 0 / 2 | Prunetin |
| Serotonin 2c (5-HT2c) receptor | HTR2C | P28335 | CHEMBL225 | Family A G protein-coupled receptor | 0.139061947 | 0 / 2 | Prunetin |
| Estrogen-related receptor beta | ESRRB | O95718 | CHEMBL3751 | Nuclear receptor | 0.139061947 | 0 / 1 | Prunetin |
| Cytochrome P450 19A1 | CYP19A1 | P11511 | CHEMBL1978 | Cytochrome P450 | 0.139061947 | 3 / 28 | Prunetin |
| Cyclooxygenase-1 | PTGS1 | P23219 | CHEMBL221 | Oxidoreductase | 0.130791955 | 0 / 2 | Prunetin |
| Norepinephrine transporter | SLC6A2 | P23975 | CHEMBL222 | Electrochemical transporter | 0.130791955 | 0 / 1 | Prunetin |
| Carbonic anhydrase VII | CA7 | P43166 | CHEMBL2326 | Lyase | 0.114337559 | 4 / 22 | Prunetin |
| Carbonic anhydrase IV | CA4 | P22748 | CHEMBL3729 | Lyase | 0.114337559 | 5 / 13 | Prunetin |
| Macrophage migration inhibitory factor | MIF | P14174 | CHEMBL2085 | Enzyme | 0.106099949 | 0 / 7 | Prunetin |
| P-glycoprotein 1 | ABCB1 | P08183 | CHEMBL4302 | Primary active transporter | 0.097874534 | 6 / 36 | Prunetin |
| Estrogen-related receptor alpha | ESRRA | P11474 | CHEMBL3429 | Nuclear receptor | 0.097874534 | 1 / 2 | Prunetin |
| ATP-binding cassette sub-family G member 2 | ABCG2 | Q9UNQ0 | CHEMBL5393 | Primary active transporter | 0.097874534 | 4 / 37 | Prunetin |
| Monoamine oxidase A | MAOA | P21397 | CHEMBL1951 | Oxidoreductase | 0.097874534 | 2 / 64 | Prunetin |
| Carbonic anhydrase II | CA2 | P00918 | CHEMBL205 | Lyase | 0.097874534 | 10 / 25 | Prunetin |
| Carbonic anhydrase I | CA1 | P00915 | CHEMBL261 | Lyase | 0.097874534 | 5 / 26 | Prunetin |
| Arachidonate 12-lipoxygenase | ALOX12 | P18054 | CHEMBL3687 | Enzyme | 0.097874534 | 8 / 12 | Prunetin |
| Monoamine oxidase B | MAOB | P27338 | CHEMBL2039 | Oxidoreductase | 0.097874534 | 0 / 130 | Prunetin |
| Receptor-type tyrosine-protein phosphatase S | PTPRS | Q13332 | CHEMBL2396508 | Phosphatase | 0.097874534 | 2 / 7 | Prunetin |
| Xanthine dehydrogenase | XDH | P47989 | CHEMBL1929 | Oxidoreductase | 0.097874534 | 9 / 19 | Prunetin |
| Peroxisome proliferator-activated receptor alpha | PPARA | Q07869 | CHEMBL239 | Nuclear receptor | 0.097874534 | 0 / 1 | Prunetin |
| 6-phosphofructo-2-kinase/fructose-2,6-bisphosphatase 3 | PFKFB3 | Q16875 | CHEMBL2331053 | Enzyme | 0.097874534 | 2 / 2 | Prunetin |
| Toll-like receptor (TLR7/TLR9) | TLR9 | Q9NR96 | CHEMBL5804 | Toll-like and Il-1 receptors | 0.097874534 | 0 / 2 | Prunetin |
| Arachidonate 15-lipoxygenase | ALOX15 | P16050 | CHEMBL2903 | Enzyme | 0.097874534 | 5 / 9 | Prunetin |
| Tyrosinase | TYR | P14679 | CHEMBL1973 | Oxidoreductase | 0.097874534 | 1 / 2 | Prunetin |
| Serum paraoxonase/arylesterase 1 | PON1 | P27169 | CHEMBL3167 | Enzyme | 0.097874534 | 0 / 1 | Prunetin |
| NADPH oxidase 4 | NOX4 | Q9NPH5 | CHEMBL1250375 | Enzyme | 0.097874534 | 5 / 7 | Prunetin |
| Carbonic anhydrase IX | CA9 | Q16790 | CHEMBL3594 | Lyase | 0.097874534 | 4 / 36 | Prunetin |
| Steryl-sulfatase | STS | P08842 | CHEMBL3559 | Enzyme | 0.097874534 | 0 / 2 | Prunetin |
| Carbonic anhydrase III | CA3 | P07451 | CHEMBL2885 | Lyase | 0.097874534 | 0 / 8 | Prunetin |
| Carbonic anhydrase XIV | CA14 | Q9ULX7 | CHEMBL3510 | Lyase | 0.097874534 | 0 / 12 | Prunetin |
| Carbonic anhydrase XIII | CA13 | Q8N1Q1 | CHEMBL3912 | Lyase | 0.097874534 | 0 / 16 | Prunetin |
| Carbonic anhydrase VB | CA5B | Q9Y2D0 | CHEMBL3969 | Lyase | 0.097874534 | 0 / 7 | Prunetin |
| Carbonic anhydrase VA | CA5A | P35218 | CHEMBL4789 | Lyase | 0.097874534 | 0 / 6 | Prunetin |
| Calcium-activated potassium channel subunit alpha-1 | KCNMA1 | Q12791 | CHEMBL4304 | Voltage-gated ion channel | 0.097874534 | 5 / 0 | Prunetin |
| Arachidonate 5-lipoxygenase | ALOX5 | P09917 | CHEMBL215 | Oxidoreductase | 0.097874534 | 4 / 42 | Prunetin |
| Dopamine beta-hydroxylase | DBH | P09172 | CHEMBL3102 | Enzyme | 0.116739032 | 2 / 0 | Pyrocatechin |
| NADPH oxidase 4 | NOX4 | Q9NPH5 | CHEMBL1250375 | Enzyme | 1 | 7 / 8 | Quercetin |
| Vasopressin V2 receptor | AVPR2 | P30518 | CHEMBL1790 | Family A G protein-coupled receptor | 1 | 1 / 1 | Quercetin |
| Aldose reductase | AKR1B1 | P15121 | CHEMBL1900 | Enzyme | 1 | 18 / 72 | Quercetin |
| Xanthine dehydrogenase | XDH | P47989 | CHEMBL1929 | Oxidoreductase | 1 | 12 / 20 | Quercetin |
| Monoamine oxidase A | MAOA | P21397 | CHEMBL1951 | Oxidoreductase | 1 | 4 / 14 | Quercetin |
| Insulin-like growth factor I receptor | IGF1R | P08069 | CHEMBL1957 | Kinase | 1 | 3 / 3 | Quercetin |
| Tyrosine-protein kinase receptor FLT3 | FLT3 | P36888 | CHEMBL1974 | Kinase | 1 | 5 / 7 | Quercetin |
| Cytochrome P450 19A1 | CYP19A1 | P11511 | CHEMBL1978 | Cytochrome P450 | 1 | 5 / 18 | Quercetin |
| Epidermal growth factor receptor erbB1 | EGFR | P00533 | CHEMBL203 | Kinase | 1 | 5 / 28 | Quercetin |
| Thrombin | F2 | P00734 | CHEMBL204 | Protease | 1 | 11 / 3 | Quercetin |
| Carbonic anhydrase II | CA2 | P00918 | CHEMBL205 | Lyase | 1 | 7 / 15 | Quercetin |
| Serine/threonine-protein kinase PIM1 | PIM1 | P11309 | CHEMBL2147 | Kinase | 1 | 7 / 7 | Quercetin |
| Arachidonate 5-lipoxygenase | ALOX5 | P09917 | CHEMBL215 | Oxidoreductase | 1 | 5 / 46 | Quercetin |
| Serine/threonine-protein kinase Aurora-B | AURKB | Q96GD4 | CHEMBL2185 | Kinase | 1 | 3 / 4 | Quercetin |
| Dopamine D4 receptor | DRD4 | P21917 | CHEMBL219 | Family A G protein-coupled receptor | 1 | 1 / 1 | Quercetin |
| Adenosine A1 receptor (by homology) | ADORA1 | P30542 | CHEMBL226 | Family A G protein-coupled receptor | 1 | 6 / 23 | Quercetin |
| Carbonic anhydrase VII | CA7 | P43166 | CHEMBL2326 | Lyase | 1 | 8 / 14 | Quercetin |
| Glyoxalase I | GLO1 | Q04760 | CHEMBL2424 | Enzyme | 1 | 3 / 4 | Quercetin |
| Myeloperoxidase | MPO | P05164 | CHEMBL2439 | Enzyme | 1 | 1 / 1 | Quercetin |
| PI3-kinase p85-alpha subunit | PIK3R1 | P27986 | CHEMBL2506 | Enzyme | 1 | 1 / 1 | Quercetin |
| Adenosine A2a receptor (by homology) | ADORA2A | P29274 | CHEMBL251 | Family A G protein-coupled receptor | 1 | 5 / 11 | Quercetin |
| Death-associated protein kinase 1 | DAPK1 | P53355 | CHEMBL2558 | Kinase | 1 | 2 / 2 | Quercetin |
| Liver glycogen phosphorylase | PYGL | P06737 | CHEMBL2568 | Enzyme | 1 | 1 / 1 | Quercetin |
| Carbonic anhydrase I | CA1 | P00915 | CHEMBL261 | Lyase | 1 | 3 / 5 | Quercetin |
| Glycogen synthase kinase-3 beta | GSK3B | P49841 | CHEMBL262 | Kinase | 1 | 3 / 6 | Quercetin |
| Tyrosine-protein kinase SRC | SRC | P12931 | CHEMBL267 | Kinase | 1 | 2 / 10 | Quercetin |
| Focal adhesion kinase 1 | PTK2 | Q05397 | CHEMBL2695 | Kinase | 1 | 1 / 2 | Quercetin |
| Estradiol 17-beta-dehydrogenase 2 | HSD17B2 | P37059 | CHEMBL2789 | Enzyme | 1 | 8 / 3 | Quercetin |
| Vascular endothelial growth factor receptor 2 | KDR | P35968 | CHEMBL279 | Kinase | 1 | 2 / 3 | Quercetin |
| Matrix metalloproteinase 13 | MMP13 | P45452 | CHEMBL280 | Protease | 1 | 1 / 1 | Quercetin |
| Matrix metalloproteinase 3 | MMP3 | P08254 | CHEMBL283 | Protease | 1 | 1 / 1 | Quercetin |
| Carbonic anhydrase III | CA3 | P07451 | CHEMBL2885 | Lyase | 1 | 1 / 1 | Quercetin |
| Arachidonate 15-lipoxygenase | ALOX15 | P16050 | CHEMBL2903 | Enzyme | 1 | 4 / 8 | Quercetin |
| Multidrug resistance-associated protein 1 | ABCC1 | P33527 | CHEMBL3004 | Primary active transporter | 1 | 7 / 11 | Quercetin |
| Serine/threonine-protein kinase PLK1 | PLK1 | P53350 | CHEMBL3024 | Kinase | 1 | 2 / 3 | Quercetin |
| Carbonic anhydrase VI | CA6 | P23280 | CHEMBL3025 | Lyase | 1 | 1 / 1 | Quercetin |
| Cyclin-dependent kinase 1 | CDK1 | P06493 | CHEMBL308 | Kinase | 1 | 2 / 13 | Quercetin |
| Matrix metalloproteinase 9 | MMP9 | P14780 | CHEMBL321 | Protease | 1 | 2 / 2 | Quercetin |
| Carbonic anhydrase XII | CA12 | O43570 | CHEMBL3242 | Lyase | 1 | 8 / 17 | Quercetin |
| Matrix metalloproteinase 2 | MMP2 | P08253 | CHEMBL333 | Protease | 1 | 2 / 2 | Quercetin |
| Protein kinase N1 | PKN1 | Q16512 | CHEMBL3384 | Kinase | 1 | 1 / 3 | Quercetin |
| Carbonic anhydrase XIV | CA14 | Q9ULX7 | CHEMBL3510 | Lyase | 1 | 1 / 1 | Quercetin |
| Carbonic anhydrase IX | CA9 | Q16790 | CHEMBL3594 | Lyase | 1 | 3 / 5 | Quercetin |
| Casein kinase II alpha | CSNK2A1 | P68400 | CHEMBL3629 | Kinase | 1 | 3 / 2 | Quercetin |
| Arachidonate 12-lipoxygenase | ALOX12 | P18054 | CHEMBL3687 | Enzyme | 1 | 5 / 10 | Quercetin |
| Hepatocyte growth factor receptor | MET | P08581 | CHEMBL3717 | Kinase | 1 | 2 / 4 | Quercetin |
| Carbonic anhydrase IV | CA4 | P22748 | CHEMBL3729 | Lyase | 1 | 7 / 13 | Quercetin |
| Serine/threonine-protein kinase NEK2 | NEK2 | P51955 | CHEMBL3835 | Kinase | 1 | 1 / 2 | Quercetin |
| Interleukin-8 receptor A | CXCR1 | P25024 | CHEMBL4029 | Family A G protein-coupled receptor | 1 | 1 / 1 | Quercetin |
| CaM kinase II beta | CAMK2B | Q13554 | CHEMBL4121 | Kinase | 1 | 1 / 2 | Quercetin |
| ALK tyrosine kinase receptor | ALK | Q9UM73 | CHEMBL4247 | Kinase | 1 | 2 / 4 | Quercetin |
| Serine/threonine-protein kinase AKT | AKT1 | P31749 | CHEMBL4282 | Kinase | 1 | 1 / 4 | Quercetin |
| P-glycoprotein 1 | ABCB1 | P08183 | CHEMBL4302 | Primary active transporter | 1 | 11 / 48 | Quercetin |
| Serine/threonine-protein kinase NEK6 | NEK6 | Q9HC98 | CHEMBL4309 | Kinase | 1 | 1 / 2 | Quercetin |
| Phospholipase A2 group 1B | PLA2G1B | P04054 | CHEMBL4426 | Enzyme | 1 | 1 / 1 | Quercetin |
| Carbonic anhydrase VA | CA5A | P35218 | CHEMBL4789 | Lyase | 1 | 1 / 1 | Quercetin |
| Beta-secretase 1 | BACE1 | P56817 | CHEMBL4822 | Protease | 1 | 8 / 14 | Quercetin |
| Cytochrome P450 1B1 | CYP1B1 | Q16678 | CHEMBL4878 | Cytochrome P450 | 1 | 12 / 46 | Quercetin |
| Tyrosine-protein kinase receptor UFO | AXL | P30530 | CHEMBL4895 | Kinase | 1 | 2 / 4 | Quercetin |
| ATP-binding cassette sub-family G member 2 | ABCG2 | Q9UNQ0 | CHEMBL5393 | Primary active transporter | 1 | 6 / 50 | Quercetin |
| NUAK family SNF1-like kinase 1 | NUAK1 | O60285 | CHEMBL5784 | Kinase | 1 | 1 / 2 | Quercetin |
| Aldo-keto reductase family 1 member C2 (by homology) | AKR1C2 | P52895 | CHEMBL5847 | Enzyme | 1 | 1 / 1 | Quercetin |
| Aldo-keto reductase family 1 member C1 (by homology) | AKR1C1 | Q04828 | CHEMBL5905 | Enzyme | 1 | 1 / 1 | Quercetin |
| Aldo-keto-reductase family 1 member C3 (by homology) | AKR1C3 | P42330 | CHEMBL4681 | Enzyme | 1 | 1 / 1 | Quercetin |
| Aldo-keto reductase family 1 member C4 (by homology) | AKR1C4 | P17516 | CHEMBL4999 | Enzyme | 1 | 1 / 1 | Quercetin |
| Carbonic anhydrase XIII (by homology) | CA13 | Q8N1Q1 | CHEMBL3912 | Lyase | 1 | 1 / 1 | Quercetin |
| Aldehyde reductase (by homology) | AKR1A1 | P14550 | CHEMBL2246 | Enzyme | 1 | 1 / 1 | Quercetin |
| G-protein coupled receptor 35 | GPR35 | Q9HC97 | CHEMBL1293267 | Family A G protein-coupled receptor | 1 | 2 / 4 | Quercetin |
| Microtubule-associated protein tau | MAPT | P10636 | CHEMBL1293224 | Unclassified protein | 0.680283592 | 1 / 1 | Quercetin |
| Lysine-specific demethylase 4D-like | KDM4E | B2RXH2 | CHEMBL1293226 | Eraser | 0.680283592 | 2 / 2 | Quercetin |
| DNA topoisomerase II alpha | TOP2A | P11388 | CHEMBL1806 | Isomerase | 0.680283592 | 1 / 1 | Quercetin |
| Insulin receptor | INSR | P06213 | CHEMBL1981 | Kinase | 0.680283592 | 1 / 1 | Quercetin |
| Acetylcholinesterase | ACHE | P22303 | CHEMBL220 | Hydrolase | 0.680283592 | 4 / 27 | Quercetin |
| Myosin light chain kinase, smooth muscle | MYLK | Q15746 | CHEMBL2428 | Kinase | 0.680283592 | 1 / 1 | Quercetin |
| Tyrosine-protein kinase SYK | SYK | P43405 | CHEMBL2599 | Kinase | 0.680283592 | 3 / 3 | Quercetin |
| PI3-kinase p110-gamma subunit | PIK3CG | P48736 | CHEMBL3267 | Enzyme | 0.680283592 | 3 / 1 | Quercetin |
| DNA-(apurinic or apyrimidinic site) lyase | APEX1 | P27695 | CHEMBL5619 | Enzyme | 0.680283592 | 1 / 1 | Quercetin |
| Receptor-type tyrosine-protein phosphatase S | PTPRS | Q13332 | CHEMBL2396508 | Phosphatase | 0.545871745 | 6 / 8 | Quercetin |
| Estrogen receptor beta | ESR2 | Q92731 | CHEMBL242 | Nuclear receptor | 0.545871745 | 62 / 29 | Quercetin |
| DNA-3-methyladenine glycosylase | MPG | P29372 | CHEMBL3396943 | Enzyme | 0.545871745 | 1 / 1 | Quercetin |
| Solute carrier family 22 member 12 | SLC22A12 | Q96S37 | CHEMBL6120 | Electrochemical transporter | 0.545871745 | 4 / 1 | Quercetin |
| Cyclin-dependent kinase 5/CDK5 activator 1 | CDK5R1 CDK5 | Q15078 Q00535 | CHEMBL1907600 | Kinase | 0.538061807 | 6 / 18 | Quercetin |
| Cyclin-dependent kinase 1/cyclin B | CCNB3 CDK1 CCNB1 CCNB2 | Q8WWL7 P06493 P14635 O95067 | CHEMBL2094127 | Other cytosolic protein | 0.538061807 | 4 / 9 | Quercetin |
| Arginase-1 (by homology) | ARG1 | P05089 | CHEMBL1075097 | Enzyme | 0.538061807 | 2 / 2 | Quercetin |
| Cyclin-dependent kinase 6 | CDK6 | Q00534 | CHEMBL2508 | Kinase | 0.49851319 | 3 / 4 | Quercetin |
| Cyclin-dependent kinase 2 | CDK2 | P24941 | CHEMBL301 | Kinase | 0.49851319 | 1 / 17 | Quercetin |
| Tyrosinase | TYR | P14679 | CHEMBL1973 | Oxidoreductase | 0.395895175 | 2 / 3 | Quercetin |
| Estradiol 17-beta-dehydrogenase 1 | HSD17B1 | P14061 | CHEMBL3181 | Enzyme | 0.395895175 | 8 / 4 | Quercetin |
| Aryl hydrocarbon receptor | AHR | P35869 | CHEMBL3201 | Transcription factor | 0.395895175 | 1 / 1 | Quercetin |
| Estrogen-related receptor alpha | ESRRA | P11474 | CHEMBL3429 | Nuclear receptor | 0.395895175 | 2 / 2 | Quercetin |
| Beta amyloid A4 protein | APP | P05067 | CHEMBL2487 | Membrane receptor | 0.261559713 | 2 / 12 | Quercetin |
| Poly [ADP-ribose] polymerase-1 | PARP1 | P09874 | CHEMBL3105 | Enzyme | 0.261559713 | 3 / 9 | Quercetin |
| Transthyretin | TTR | P02766 | CHEMBL3194 | Secreted protein | 0.261559713 | 2 / 2 | Quercetin |
| Matrix metalloproteinase 12 | MMP12 | P39900 | CHEMBL4393 | Protease | 0.261559713 | 1 / 1 | Quercetin |
| Lymphocyte differentiation antigen CD38 | CD38 | P28907 | CHEMBL4660 | Enzyme | 0.261559713 | 2 / 3 | Quercetin |
| Aldo-keto reductase family 1 member B10 | AKR1B10 | O60218 | CHEMBL5983 | Enzyme | 0.261559713 | 2 / 3 | Quercetin |
| Tankyrase-2 | TNKS2 | Q9H2K2 | CHEMBL6154 | Enzyme | 0.261559713 | 4 / 12 | Quercetin |
| Tankyrase-1 | TNKS | O95271 | CHEMBL6164 | Enzyme | 0.261559713 | 4 / 28 | Quercetin |
| DNA topoisomerase I (by homology) | TOP1 | P11387 | CHEMBL1781 | Isomerase | 0.261559713 | 1 / 1 | Quercetin |
| Telomerase reverse transcriptase | TERT | O14746 | CHEMBL2916 | Enzyme | 0.198389229 | 9 / 22 | Quercetin |
| Aldose reductase | AKR1B1 | P15121 | CHEMBL1900 | Enzyme | 0.77654935 | 25 / 75 | Rhamnetin |
| NADPH oxidase 4 | NOX4 | Q9NPH5 | CHEMBL1250375 | Enzyme | 0.659830583 | 7 / 8 | Rhamnetin |
| Epidermal growth factor receptor erbB1 | EGFR | P00533 | CHEMBL203 | Kinase | 0.384850756 | 7 / 29 | Rhamnetin |
| Serine/threonine-protein kinase PIM1 | PIM1 | P11309 | CHEMBL2147 | Kinase | 0.36819132 | 8 / 7 | Rhamnetin |
| Adenosine A1 receptor (by homology) | ADORA1 | P30542 | CHEMBL226 | Family A G protein-coupled receptor | 0.36819132 | 6 / 23 | Rhamnetin |
| Adenosine A2a receptor (by homology) | ADORA2A | P29274 | CHEMBL251 | Family A G protein-coupled receptor | 0.36819132 | 6 / 11 | Rhamnetin |
| Xanthine dehydrogenase | XDH | P47989 | CHEMBL1929 | Oxidoreductase | 0.284878497 | 13 / 21 | Rhamnetin |
| Cytochrome P450 1B1 | CYP1B1 | Q16678 | CHEMBL4878 | Cytochrome P450 | 0.218248072 | 12 / 46 | Rhamnetin |
| Thrombin | F2 | P00734 | CHEMBL204 | Protease | 0.218248072 | 16 / 2 | Rhamnetin |
| Arachidonate 5-lipoxygenase | ALOX5 | P09917 | CHEMBL215 | Oxidoreductase | 0.193269571 | 5 / 47 | Rhamnetin |
| Beta-secretase 1 | BACE1 | P56817 | CHEMBL4822 | Protease | 0.193269571 | 12 / 17 | Rhamnetin |
| Carbonic anhydrase II | CA2 | P00918 | CHEMBL205 | Lyase | 0.184930052 | 11 / 17 | Rhamnetin |
| Carbonic anhydrase VII | CA7 | P43166 | CHEMBL2326 | Lyase | 0.184930052 | 8 / 16 | Rhamnetin |
| Carbonic anhydrase XII | CA12 | O43570 | CHEMBL3242 | Lyase | 0.184930052 | 11 / 20 | Rhamnetin |
| Carbonic anhydrase IV | CA4 | P22748 | CHEMBL3729 | Lyase | 0.184930052 | 8 / 13 | Rhamnetin |
| Estradiol 17-beta-dehydrogenase 2 | HSD17B2 | P37059 | CHEMBL2789 | Enzyme | 0.184930052 | 8 / 3 | Rhamnetin |
| Insulin-like growth factor I receptor | IGF1R | P08069 | CHEMBL1957 | Kinase | 0.168264714 | 5 / 3 | Rhamnetin |
| Beta amyloid A4 protein | APP | P05067 | CHEMBL2487 | Membrane receptor | 0.159936683 | 2 / 12 | Rhamnetin |
| G-protein coupled receptor 35 | GPR35 | Q9HC97 | CHEMBL1293267 | Family A G protein-coupled receptor | 0.159936683 | 2 / 4 | Rhamnetin |
| Tyrosine-protein kinase receptor FLT3 | FLT3 | P36888 | CHEMBL1974 | Kinase | 0.159936683 | 6 / 7 | Rhamnetin |
| Death-associated protein kinase 1 | DAPK1 | P53355 | CHEMBL2558 | Kinase | 0.159936683 | 2 / 2 | Rhamnetin |
| Multidrug resistance-associated protein 1 | ABCC1 | P33527 | CHEMBL3004 | Primary active transporter | 0.159936683 | 7 / 11 | Rhamnetin |
| P-glycoprotein 1 | ABCB1 | P08183 | CHEMBL4302 | Primary active transporter | 0.159936683 | 14 / 47 | Rhamnetin |
| ATP-binding cassette sub-family G member 2 | ABCG2 | Q9UNQ0 | CHEMBL5393 | Primary active transporter | 0.159936683 | 6 / 50 | Rhamnetin |
| Vasopressin V2 receptor | AVPR2 | P30518 | CHEMBL1790 | Family A G protein-coupled receptor | 0.143269257 | 1 / 1 | Rhamnetin |
| Monoamine oxidase A | MAOA | P21397 | CHEMBL1951 | Oxidoreductase | 0.143269257 | 4 / 15 | Rhamnetin |
| Cytochrome P450 19A1 | CYP19A1 | P11511 | CHEMBL1978 | Cytochrome P450 | 0.143269257 | 6 / 18 | Rhamnetin |
| Serine/threonine-protein kinase Aurora-B | AURKB | Q96GD4 | CHEMBL2185 | Kinase | 0.143269257 | 5 / 4 | Rhamnetin |
| Dopamine D4 receptor | DRD4 | P21917 | CHEMBL219 | Family A G protein-coupled receptor | 0.143269257 | 1 / 1 | Rhamnetin |
| Glyoxalase I | GLO1 | Q04760 | CHEMBL2424 | Enzyme | 0.143269257 | 3 / 4 | Rhamnetin |
| Myeloperoxidase | MPO | P05164 | CHEMBL2439 | Enzyme | 0.143269257 | 1 / 1 | Rhamnetin |
| PI3-kinase p85-alpha subunit | PIK3R1 | P27986 | CHEMBL2506 | Enzyme | 0.143269257 | 1 / 1 | Rhamnetin |
| Liver glycogen phosphorylase | PYGL | P06737 | CHEMBL2568 | Enzyme | 0.143269257 | 1 / 1 | Rhamnetin |
| Carbonic anhydrase I | CA1 | P00915 | CHEMBL261 | Lyase | 0.143269257 | 5 / 10 | Rhamnetin |
| Glycogen synthase kinase-3 beta | GSK3B | P49841 | CHEMBL262 | Kinase | 0.143269257 | 3 / 6 | Rhamnetin |
| Tyrosine-protein kinase SRC | SRC | P12931 | CHEMBL267 | Kinase | 0.143269257 | 4 / 10 | Rhamnetin |
| Focal adhesion kinase 1 | PTK2 | Q05397 | CHEMBL2695 | Kinase | 0.143269257 | 3 / 2 | Rhamnetin |
| Vascular endothelial growth factor receptor 2 | KDR | P35968 | CHEMBL279 | Kinase | 0.143269257 | 4 / 3 | Rhamnetin |
| Matrix metalloproteinase 13 | MMP13 | P45452 | CHEMBL280 | Protease | 0.143269257 | 1 / 1 | Rhamnetin |
| Matrix metalloproteinase 3 | MMP3 | P08254 | CHEMBL283 | Protease | 0.143269257 | 1 / 1 | Rhamnetin |
| Carbonic anhydrase III | CA3 | P07451 | CHEMBL2885 | Lyase | 0.143269257 | 1 / 2 | Rhamnetin |
| Arachidonate 15-lipoxygenase | ALOX15 | P16050 | CHEMBL2903 | Enzyme | 0.143269257 | 7 / 6 | Rhamnetin |
| Serine/threonine-protein kinase PLK1 | PLK1 | P53350 | CHEMBL3024 | Kinase | 0.143269257 | 4 / 3 | Rhamnetin |
| Carbonic anhydrase VI | CA6 | P23280 | CHEMBL3025 | Lyase | 0.143269257 | 2 / 1 | Rhamnetin |
| Cyclin-dependent kinase 1 | CDK1 | P06493 | CHEMBL308 | Kinase | 0.143269257 | 3 / 12 | Rhamnetin |
| Matrix metalloproteinase 9 | MMP9 | P14780 | CHEMBL321 | Protease | 0.143269257 | 4 / 2 | Rhamnetin |
| Matrix metalloproteinase 2 | MMP2 | P08253 | CHEMBL333 | Protease | 0.143269257 | 4 / 2 | Rhamnetin |
| Protein kinase N1 | PKN1 | Q16512 | CHEMBL3384 | Kinase | 0.143269257 | 3 / 3 | Rhamnetin |
| Carbonic anhydrase XIV | CA14 | Q9ULX7 | CHEMBL3510 | Lyase | 0.143269257 | 1 / 2 | Rhamnetin |
| Carbonic anhydrase IX | CA9 | Q16790 | CHEMBL3594 | Lyase | 0.143269257 | 5 / 8 | Rhamnetin |
| Casein kinase II alpha | CSNK2A1 | P68400 | CHEMBL3629 | Kinase | 0.143269257 | 2 / 2 | Rhamnetin |
| Arachidonate 12-lipoxygenase | ALOX12 | P18054 | CHEMBL3687 | Enzyme | 0.143269257 | 10 / 8 | Rhamnetin |
| Hepatocyte growth factor receptor | MET | P08581 | CHEMBL3717 | Kinase | 0.143269257 | 6 / 4 | Rhamnetin |
| Serine/threonine-protein kinase NEK2 | NEK2 | P51955 | CHEMBL3835 | Kinase | 0.143269257 | 3 / 2 | Rhamnetin |
| Carbonic anhydrase XIII (by homology) | CA13 | Q8N1Q1 | CHEMBL3912 | Lyase | 0.143269257 | 1 / 3 | Rhamnetin |
| Interleukin-8 receptor A | CXCR1 | P25024 | CHEMBL4029 | Family A G protein-coupled receptor | 0.143269257 | 2 / 1 | Rhamnetin |
| CaM kinase II beta | CAMK2B | Q13554 | CHEMBL4121 | Kinase | 0.143269257 | 1 / 2 | Rhamnetin |
| ALK tyrosine kinase receptor | ALK | Q9UM73 | CHEMBL4247 | Kinase | 0.143269257 | 5 / 4 | Rhamnetin |
| Serine/threonine-protein kinase AKT | AKT1 | P31749 | CHEMBL4282 | Kinase | 0.143269257 | 4 / 4 | Rhamnetin |
| Serine/threonine-protein kinase NEK6 | NEK6 | Q9HC98 | CHEMBL4309 | Kinase | 0.143269257 | 3 / 2 | Rhamnetin |
| Phospholipase A2 group 1B | PLA2G1B | P04054 | CHEMBL4426 | Enzyme | 0.143269257 | 1 / 1 | Rhamnetin |
| Carbonic anhydrase VA | CA5A | P35218 | CHEMBL4789 | Lyase | 0.143269257 | 1 / 2 | Rhamnetin |
| Tyrosine-protein kinase receptor UFO | AXL | P30530 | CHEMBL4895 | Kinase | 0.143269257 | 4 / 4 | Rhamnetin |
| NUAK family SNF1-like kinase 1 | NUAK1 | O60285 | CHEMBL5784 | Kinase | 0.143269257 | 3 / 2 | Rhamnetin |
| Aldo-keto reductase family 1 member C2 (by homology) | AKR1C2 | P52895 | CHEMBL5847 | Enzyme | 0.143269257 | 1 / 1 | Rhamnetin |
| Aldo-keto reductase family 1 member C1 (by homology) | AKR1C1 | Q04828 | CHEMBL5905 | Enzyme | 0.143269257 | 1 / 1 | Rhamnetin |
| Aldo-keto-reductase family 1 member C3 (by homology) | AKR1C3 | P42330 | CHEMBL4681 | Enzyme | 0.143269257 | 1 / 1 | Rhamnetin |
| Aldo-keto reductase family 1 member C4 (by homology) | AKR1C4 | P17516 | CHEMBL4999 | Enzyme | 0.143269257 | 1 / 1 | Rhamnetin |
| Aldehyde reductase (by homology) | AKR1A1 | P14550 | CHEMBL2246 | Enzyme | 0.143269257 | 1 / 1 | Rhamnetin |
| Cyclin-dependent kinase 5/CDK5 activator 1 | CDK5R1 CDK5 | Q15078 Q00535 | CHEMBL1907600 | Kinase | 0.134939009 | 5 / 18 | Rhamnetin |
| Telomerase reverse transcriptase | TERT | O14746 | CHEMBL2916 | Enzyme | 0.134939009 | 9 / 22 | Rhamnetin |
| Tyrosine-protein kinase SYK | SYK | P43405 | CHEMBL2599 | Kinase | 0.134939009 | 3 / 3 | Rhamnetin |
| Acetylcholinesterase | ACHE | P22303 | CHEMBL220 | Hydrolase | 0.126610169 | 4 / 31 | Rhamnetin |
| Microtubule-associated protein tau | MAPT | P10636 | CHEMBL1293224 | Unclassified protein | 0.126610169 | 1 / 1 | Rhamnetin |
| Lysine-specific demethylase 4D-like | KDM4E | B2RXH2 | CHEMBL1293226 | Eraser | 0.126610169 | 2 / 2 | Rhamnetin |
| DNA topoisomerase II alpha | TOP2A | P11388 | CHEMBL1806 | Isomerase | 0.126610169 | 1 / 1 | Rhamnetin |
| Insulin receptor | INSR | P06213 | CHEMBL1981 | Kinase | 0.126610169 | 1 / 1 | Rhamnetin |
| Myosin light chain kinase, smooth muscle | MYLK | Q15746 | CHEMBL2428 | Kinase | 0.126610169 | 1 / 1 | Rhamnetin |
| PI3-kinase p110-gamma subunit | PIK3CG | P48736 | CHEMBL3267 | Enzyme | 0.126610169 | 5 / 1 | Rhamnetin |
| DNA-(apurinic or apyrimidinic site) lyase | APEX1 | P27695 | CHEMBL5619 | Enzyme | 0.126610169 | 1 / 1 | Rhamnetin |
| Estradiol 17-beta-dehydrogenase 1 | HSD17B1 | P14061 | CHEMBL3181 | Enzyme | 0.126610169 | 8 / 4 | Rhamnetin |
| Plasminogen | PLG | P00747 | CHEMBL1801 | Protease | 0.118277085 | 6 / 2 | Rhamnetin |
| Tyrosinase | TYR | P14679 | CHEMBL1973 | Oxidoreductase | 0.118277085 | 2 / 2 | Rhamnetin |
| Aryl hydrocarbon receptor | AHR | P35869 | CHEMBL3201 | Transcription factor | 0.118277085 | 1 / 1 | Rhamnetin |
| Estrogen-related receptor alpha | ESRRA | P11474 | CHEMBL3429 | Nuclear receptor | 0.118277085 | 2 / 2 | Rhamnetin |
| Cyclin-dependent kinase 1/cyclin B | CCNB3 CDK1 CCNB1 CCNB2 | Q8WWL7 P06493 P14635 O95067 | CHEMBL2094127 | Other cytosolic protein | 0.10994577 | 4 / 6 | Rhamnetin |
| Arginase-1 (by homology) | ARG1 | P05089 | CHEMBL1075097 | Enzyme | 0.10994577 | 2 / 2 | Rhamnetin |
| Cyclin-dependent kinase 6 | CDK6 | Q00534 | CHEMBL2508 | Kinase | 0.10994577 | 3 / 4 | Rhamnetin |
| Cyclin-dependent kinase 2 | CDK2 | P24941 | CHEMBL301 | Kinase | 0.10994577 | 1 / 16 | Rhamnetin |
| Adenosine A3 receptor | ADORA3 | P0DMS8 | CHEMBL256 | Family A G protein-coupled receptor | 0.10994577 | 1 / 23 | Rhamnetin |
| Estrogen receptor beta | ESR2 | Q92731 | CHEMBL242 | Nuclear receptor | 0.10994577 | 55 / 23 | Rhamnetin |
| Beta-galactoside alpha-2,6-sialyltransferase 1 | ST6GAL1 | P15907 | CHEMBL3596075 | Transferase | 0.10994577 | 0 / 2 | Rhamnetin |
| Receptor-type tyrosine-protein phosphatase S | PTPRS | Q13332 | CHEMBL2396508 | Phosphatase | 0.10994577 | 4 / 8 | Rhamnetin |
| Delta opioid receptor | OPRD1 | P41143 | CHEMBL236 | Family A G protein-coupled receptor | 0.10994577 | 2 / 5 | Rhamnetin |
| DNA-3-methyladenine glycosylase | MPG | P29372 | CHEMBL3396943 | Enzyme | 0.101613855 | 1 / 1 | Rhamnetin |
| Solute carrier family 22 member 12 | SLC22A12 | Q96S37 | CHEMBL6120 | Electrochemical transporter | 0.101613855 | 6 / 1 | Rhamnetin |
| 6-phosphofructo-2-kinase/fructose-2,6-bisphosphatase 3 | PFKFB3 | Q16875 | CHEMBL2331053 | Enzyme | 0.101613855 | 2 / 2 | Rhamnetin |
| Induced myeloid leukemia cell differentiation protein Mcl-1 | MCL1 | Q07820 | CHEMBL4361 | Other cytosolic protein | 0.101613855 | 4 / 4 | Rhamnetin |
| Nitric oxide synthase, inducible (by homology) | NOS2 | P35228 | CHEMBL4481 | Enzyme | 0.101613855 | 4 / 3 | Rhamnetin |
| Stem cell growth factor receptor | KIT | P10721 | CHEMBL1936 | Kinase | 0.101613855 | 1 / 3 | Rhamnetin |
| Protein farnesyltransferase | FNTA FNTB | P49354 P49356 | CHEMBL2094108 | Enzyme | 0.106099949 | 0 / 10 | Shelloic acid |
| TNF-alpha | TNF | P01375 | CHEMBL1825 | Secreted protein | 0.106099949 | 0 / 8 | Shelloic acid |
| Nitric oxide synthase, inducible (by homology) | NOS2 | P35228 | CHEMBL4481 | Enzyme | 0.106099949 | 0 / 22 | Shelloic acid |
| Cytochrome P450 19A1 | CYP19A1 | P11511 | CHEMBL1978 | Cytochrome P450 | 0.106099949 | 0 / 247 | Shelloic acid |
| Steroid 5-alpha-reductase 2 | SRD5A2 | P31213 | CHEMBL1856 | Oxidoreductase | 0.097874534 | 0 / 30 | Shelloic acid |
| Mineralocorticoid receptor | NR3C2 | P08235 | CHEMBL1994 | Nuclear receptor | 0.097874534 | 0 / 27 | Shelloic acid |
| 11-beta-hydroxysteroid dehydrogenase 2 | HSD11B2 | P80365 | CHEMBL3746 | Enzyme | 0.097874534 | 0 / 20 | Shelloic acid |
| 11-beta-hydroxysteroid dehydrogenase 1 | HSD11B1 | P28845 | CHEMBL4235 | Enzyme | 0.097874534 | 0 / 35 | Shelloic acid |
| Glucocorticoid receptor | NR3C1 | P04150 | CHEMBL2034 | Nuclear receptor | 0.097874534 | 0 / 40 | Shelloic acid |
| Corticosteroid binding globulin | SERPINA6 | P08185 | CHEMBL2421 | Secreted protein | 0.097874534 | 0 / 20 | Shelloic acid |
| Testis-specific androgen-binding protein | SHBG | P04278 | CHEMBL3305 | Secreted protein | 0.097874534 | 0 / 41 | Shelloic acid |
| DNA polymerase beta (by homology) | POLB | P06746 | CHEMBL2392 | Enzyme | 0.097874534 | 0 / 12 | Shelloic acid |
| Serotonin transporter (by homology) | SLC6A4 | P31645 | CHEMBL228 | Electrochemical transporter | 0.097874534 | 0 / 6 | Shelloic acid |
| Estrogen receptor alpha | ESR1 | P03372 | CHEMBL206 | Nuclear receptor | 0.097874534 | 0 / 24 | Shelloic acid |
| Androgen Receptor | AR | P10275 | CHEMBL1871 | Nuclear receptor | 0.097874534 | 0 / 110 | Shelloic acid |
| Protein kinase C eta | PRKCH | P24723 | CHEMBL3616 | Kinase | 0.097874534 | 0 / 3 | Shelloic acid |
| Dual specificity phosphatase Cdc25A | CDC25A | P30304 | CHEMBL3775 | Phosphatase | 0.097874534 | 0 / 14 | Shelloic acid |
| Protein-tyrosine phosphatase 2C | PTPN11 | Q06124 | CHEMBL3864 | Phosphatase | 0.097874534 | 0 / 2 | Shelloic acid |
| Aldo-keto reductase family 1 member B10 | AKR1B10 | O60218 | CHEMBL5983 | Enzyme | 0.097874534 | 0 / 6 | Shelloic acid |
| Neprilysin | MME | P08473 | CHEMBL1944 | Protease | 0.097874534 | 19 / 0 | Shelloic acid |
| Butyrylcholinesterase | BCHE | P06276 | CHEMBL1914 | Hydrolase | 0.097874534 | 0 / 2 | Shelloic acid |
| Leukocyte adhesion glycoprotein LFA-1 alpha | ITGAL | P20701 | CHEMBL1803 | Adhesion | 0.097874534 | 0 / 4 | Shelloic acid |
| Niemann-Pick C1-like protein 1 | NPC1L1 | Q9UHC9 | CHEMBL2027 | Other membrane protein | 0.097874534 | 0 / 9 | Shelloic acid |
| Sigma opioid receptor | SIGMAR1 | Q99720 | CHEMBL287 | Membrane receptor | 0.097874534 | 0 / 3 | Shelloic acid |
| Cytochrome P450 17A1 | CYP17A1 | P05093 | CHEMBL3522 | Cytochrome P450 | 0.097874534 | 0 / 22 | Shelloic acid |
| Prostaglandin E synthase | PTGES | O14684 | CHEMBL5658 | Enzyme | 0.097874534 | 0 / 12 | Shelloic acid |
| Prostanoid EP1 receptor | PTGER1 | P34995 | CHEMBL1811 | Family A G protein-coupled receptor | 0.097874534 | 0 / 13 | Shelloic acid |
| Prostanoid EP4 receptor | PTGER4 | P35408 | CHEMBL1836 | Family A G protein-coupled receptor | 0.097874534 | 0 / 10 | Shelloic acid |
| Prostanoid FP receptor | PTGFR | P43088 | CHEMBL1987 | Family A G protein-coupled receptor | 0.097874534 | 0 / 6 | Shelloic acid |
| Prostanoid EP3 receptor | PTGER3 | P43115 | CHEMBL3710 | Family A G protein-coupled receptor | 0.097874534 | 0 / 3 | Shelloic acid |
| Prostanoid DP receptor | PTGDR | Q13258 | CHEMBL4427 | Family A G protein-coupled receptor | 0.097874534 | 0 / 1 | Shelloic acid |
| Progesterone receptor | PGR | P06401 | CHEMBL208 | Nuclear receptor | 0.097874534 | 0 / 50 | Shelloic acid |
| Adenosine A3 receptor | ADORA3 | P0DMS8 | CHEMBL256 | Family A G protein-coupled receptor | 0.097874534 | 0 / 1 | Shelloic acid |
| MAP kinase ERK1 | MAPK3 | P27361 | CHEMBL3385 | Kinase | 0.097874534 | 0 / 1 | Shelloic acid |
| Fructose-1,6-bisphosphatase | FBP1 | P09467 | CHEMBL3975 | Enzyme | 0.097874534 | 21 / 0 | Shelloic acid |
| Carboxylesterase 2 | CES2 | O00748 | CHEMBL3180 | Enzyme | 0.097874534 | 0 / 14 | Shelloic acid |
| Aldose reductase | AKR1B1 | P15121 | CHEMBL1900 | Enzyme | 0.097874534 | 49 / 0 | Shelloic acid |
| Epidermal growth factor receptor erbB1 | EGFR | P00533 | CHEMBL203 | Kinase | 0.097874534 | 4 / 0 | Shelloic acid |
| T-cell protein-tyrosine phosphatase | PTPN2 | P17706 | CHEMBL3807 | Phosphatase | 0.097874534 | 0 / 17 | Shelloic acid |
| Leukotriene B4 receptor 1 | LTB4R | Q15722 | CHEMBL3911 | Family A G protein-coupled receptor | 0.097874534 | 0 / 6 | Shelloic acid |
| Indoleamine 2,3-dioxygenase | IDO1 | P14902 | CHEMBL4685 | Enzyme | 0.097874534 | 0 / 1 | Shelloic acid |
| Nuclear receptor ROR-alpha | RORA | P35398 | CHEMBL5868 | Nuclear receptor | 0.097874534 | 0 / 3 | Shelloic acid |
| Prostanoid EP2 receptor | PTGER2 | P43116 | CHEMBL1881 | Family A G protein-coupled receptor | 0.097874534 | 0 / 24 | Shelloic acid |
| Prostanoid IP receptor | PTGIR | P43119 | CHEMBL1995 | Family A G protein-coupled receptor | 0.097874534 | 0 / 9 | Shelloic acid |
| Solute carrier family 22 member 6 (by homology) | SLC22A6 | Q4U2R8 | CHEMBL1641347 | Electrochemical transporter | 0.097874534 | 0 / 1 | Shelloic acid |
| Peroxisome proliferator-activated receptor gamma | PPARG | P37231 | CHEMBL235 | Nuclear receptor | 0.097874534 | 0 / 18 | Shelloic acid |
| Peroxisome proliferator-activated receptor alpha | PPARA | Q07869 | CHEMBL239 | Nuclear receptor | 0.097874534 | 0 / 12 | Shelloic acid |
| Peroxisome proliferator-activated receptor delta | PPARD | Q03181 | CHEMBL3979 | Nuclear receptor | 0.097874534 | 0 / 10 | Shelloic acid |
| Solute carrier family 13 member 5 | SLC13A5 | Q86YT5 | CHEMBL3769293 | Electrochemical transporter | 0.097874534 | 10 / 0 | Shelloic acid |
| Estrogen receptor beta | ESR2 | Q92731 | CHEMBL242 | Nuclear receptor | 0.097874534 | 1 / 28 | Shelloic acid |
| Beta-secretase 1 | BACE1 | P56817 | CHEMBL4822 | Protease | 0.097874534 | 1 / 1 | Shelloic acid |
| Interleukin-6 | IL6 | P05231 | CHEMBL1795129 | Secreted protein | 0.097874534 | 0 / 1 | Shelloic acid |
| ADAM17 | ADAM17 | P78536 | CHEMBL3706 | Protease | 0.097874534 | 0 / 1 | Shelloic acid |
| Glutamine synthetase | GLUL | P15104 | CHEMBL4612 | Ligase | 0.097874534 | 0 / 1 | Shelloic acid |
| G-protein coupled bile acid receptor 1 | GPBAR1 | Q8TDU6 | CHEMBL5409 | Family A G protein-coupled receptor | 0.097874534 | 0 / 1 | Shelloic acid |
| Fatty acid-binding protein, liver (by homology) | FABP1 | P07148 | CHEMBL5421 | Fatty acid binding protein family | 0.097874534 | 0 / 3 | Shelloic acid |
| Androgen Receptor | AR | P10275 | CHEMBL1871 | Nuclear receptor | 0.829282047 | 10 / 17 | Sitosterol |
| Dual specificity phosphatase Cdc25A | CDC25A | P30304 | CHEMBL3775 | Phosphatase | 0.539364824 | 6 / 7 | Sitosterol |
| Testis-specific androgen-binding protein | SHBG | P04278 | CHEMBL3305 | Secreted protein | 0.45601846 | 0 / 22 | Sitosterol |
| Nuclear receptor subfamily 1 group I member 3 | NR1I3 | Q14994 | CHEMBL5503 | Nuclear receptor | 0.45601846 | 0 / 2 | Sitosterol |
| Dual specificity phosphatase Cdc25B | CDC25B | P30305 | CHEMBL4804 | Phosphatase | 0.339398684 | 6 / 5 | Sitosterol |
| UDP-glucuronosyltransferase 2B7 | UGT2B7 | P16662 | CHEMBL4370 | Enzyme | 0.214435241 | 0 / 24 | Sitosterol |
| Transient receptor potential cation channel subfamily M member 8 | TRPM8 | Q7Z2W7 | CHEMBL1075319 | Voltage-gated ion channel | 0.206119055 | 0 / 1 | Sitosterol |
| Estrogen receptor beta | ESR2 | Q92731 | CHEMBL242 | Nuclear receptor | 0.181090099 | 2 / 5 | Sitosterol |
| Carbonic anhydrase II | CA2 | P00918 | CHEMBL205 | Lyase | 0.172809876 | 30 / 2 | Sitosterol |
| Carbonic anhydrase I | CA1 | P00915 | CHEMBL261 | Lyase | 0.172809876 | 26 / 2 | Sitosterol |
| Niemann-Pick C1-like protein 1 | NPC1L1 | Q9UHC9 | CHEMBL2027 | Other membrane protein | 0.16447208 | 13 / 7 | Sitosterol |
| Estrogen receptor alpha | ESR1 | P03372 | CHEMBL206 | Nuclear receptor | 0.156149437 | 1 / 6 | Sitosterol |
| 11-beta-hydroxysteroid dehydrogenase 1 | HSD11B1 | P28845 | CHEMBL4235 | Enzyme | 0.147815042 | 19 / 11 | Sitosterol |
| Sonic hedgehog protein (by homology) | SHH | Q15465 | CHEMBL5602 | Unclassified protein | 0.139453236 | 13 / 10 | Sitosterol |
| Carbonic anhydrase IV | CA4 | P22748 | CHEMBL3729 | Lyase | 0.11449479 | 1 / 2 | Sitosterol |
| Bile acid receptor FXR | NR1H4 | Q96RI1 | CHEMBL2047 | Nuclear receptor | 0.11449479 | 3 / 12 | Sitosterol |
| G-protein coupled bile acid receptor 1 | GPBAR1 | Q8TDU6 | CHEMBL5409 | Family A G protein-coupled receptor | 0.11449479 | 2 / 16 | Sitosterol |
| DNA polymerase alpha subunit | POLA1 | P09884 | CHEMBL1828 | Transferase | 0.11449479 | 0 / 1 | Sitosterol |
| LXR-alpha | NR1H3 | Q13133 | CHEMBL2808 | Nuclear receptor | 0.106165761 | 18 / 10 | Sitosterol |
| Vitamin D receptor | VDR | P11473 | CHEMBL1977 | Nuclear receptor | 0.106165761 | 18 / 1 | Sitosterol |
| Anti-estrogen binding site (AEBS) (by homology) | DHCR7 | Q9UBM7 | CHEMBL2169735 | Enzyme | 0.106165761 | 3 / 0 | Sitosterol |
| Nuclear receptor ROR-alpha | RORA | P35398 | CHEMBL5868 | Nuclear receptor | 0.106165761 | 3 / 0 | Sitosterol |
| Glycine receptor subunit alpha-1 | GLRA1 | P23415 | CHEMBL5845 | Ligand-gated ion channel | 0.106165761 | 1 / 0 | Sitosterol |
| Corticosteroid binding globulin | SERPINA6 | P08185 | CHEMBL2421 | Secreted protein | 0.106165761 | 0 / 3 | Sitosterol |
| Estradiol 17-beta-dehydrogenase 3 | HSD17B3 | P37058 | CHEMBL4234 | Enzyme | 0.106165761 | 0 / 11 | Sitosterol |
| GABA-B receptor (by homology) | GABBR1 | Q9UBS5 | CHEMBL2064 | Family C G protein-coupled receptor | 0.106165761 | 0 / 4 | Sitosterol |
| Nitric oxide synthase, inducible (by homology) | NOS2 | P35228 | CHEMBL4481 | Enzyme | 0.106165761 | 1 / 0 | Sitosterol |
| LXR-beta | NR1H2 | P55055 | CHEMBL4093 | Nuclear receptor | 0.106165761 | 2 / 0 | Sitosterol |
| Carboxylesterase 2 | CES2 | O00748 | CHEMBL3180 | Enzyme | 0.106165761 | 5 / 0 | Sitosterol |
| Cytochrome P450 51 (by homology) | CYP51A1 | Q16850 | CHEMBL3849 | Cytochrome P450 | 0.106165761 | 3 / 2 | Sitosterol |
| GABA A receptor alpha-2/beta-2/gamma-2 | GABRA2 GABRB2 GABRG2 | P47869 P47870 P18507 | CHEMBL2111413 | Ligand-gated ion channel | 0.106165761 | 0 / 1 | Sitosterol |
| Aldo-keto reductase family 1 member B10 | AKR1B10 | O60218 | CHEMBL5983 | Enzyme | 0.106165761 | 0 / 2 | Sitosterol |
| DNA polymerase beta (by homology) | POLB | P06746 | CHEMBL2392 | Enzyme | 0.106165761 | 0 / 2 | Sitosterol |
| Protein kinase C alpha | PRKCA | P17252 | CHEMBL299 | Kinase | 0.106165761 | 8 / 0 | Sitosterol |
| Quinone reductase 1 | NQO1 | P15559 | CHEMBL3623 | Enzyme | 0 | 2 / 0 | Kino-Tannic acid |
| Thrombin and coagulation factor X | F10 | P00742 | CHEMBL244 | Protease | 0 | 15 / 0 | Kino-Tannic acid |
| Protein kinase C gamma | PRKCG | P05129 | CHEMBL2938 | Kinase | 0 | 19 / 0 | Kino-Tannic acid |
| Protein kinase C delta | PRKCD | Q05655 | CHEMBL2996 | Kinase | 0 | 19 / 0 | Kino-Tannic acid |
| Protein kinase C alpha | PRKCA | P17252 | CHEMBL299 | Kinase | 0 | 19 / 0 | Kino-Tannic acid |
| Protein kinase C beta | PRKCB | P05771 | CHEMBL3045 | Kinase | 0 | 21 / 0 | Kino-Tannic acid |
| Protein kinase C (PKC) | PRKCZ | Q05513 | CHEMBL3438 | Kinase | 0 | 9 / 0 | Kino-Tannic acid |
| Protein kinase C epsilon | PRKCE | Q02156 | CHEMBL3582 | Kinase | 0 | 18 / 0 | Kino-Tannic acid |
| Protein kinase C eta | PRKCH | P24723 | CHEMBL3616 | Kinase | 0 | 19 / 0 | Kino-Tannic acid |
| cAMP-dependent protein kinase alpha-catalytic subunit | PRKACA | P17612 | CHEMBL4101 | Kinase | 0 | 4 / 0 | Kino-Tannic acid |
| Cyclooxygenase-2 | PTGS2 | P35354 | CHEMBL230 | Oxidoreductase | 0 | 1 / 0 | Kino-Tannic acid |
| Insulin-like growth factor binding protein 3 | IGFBP3 | P17936 | CHEMBL3997 | Secreted protein | 0 | 3 / 0 | Kino-Tannic acid |
| Integrin alpha-V/beta-3 | ITGAV ITGB3 | P06756 P05106 | CHEMBL1907598 | Membrane receptor | 0 | 19 / 0 | Kino-Tannic acid |
| Metastin receptor | KISS1R | Q969F8 | CHEMBL5413 | Family A G protein-coupled receptor | 0 | 6 / 0 | Kino-Tannic acid |
| Coagulation factor VII | F7 | P08709 | CHEMBL3991 | Protease | 0 | 5 / 0 | Kino-Tannic acid |
| Inhibitor of nuclear factor kappa B kinase beta subunit | IKBKB | O14920 | CHEMBL1991 | Kinase | 0 | 2 / 0 | Kino-Tannic acid |
| Integrin alpha-IIb/beta-3 | ITGA2B ITGB3 | P08514 P05106 | CHEMBL2093869 | Membrane receptor | 0 | 2 / 0 | Kino-Tannic acid |
| Integrin alpha-5/beta-1 | ITGB1 ITGA5 | P05556 P08648 | CHEMBL2095226 | Membrane receptor | 0 | 2 / 0 | Kino-Tannic acid |
| Integrin alpha-V/beta-6 | ITGAV ITGB6 | P06756 P18564 | CHEMBL2111416 | Membrane receptor | 0 | 8 / 0 | Kino-Tannic acid |
| P2X purinoceptor 3 | P2RX3 | P56373 | CHEMBL2998 | Ligand-gated ion channel | 0 | 1 / 0 | Kino-Tannic acid |
| Catechol O-methyltransferase | COMT | P21964 | CHEMBL2023 | Transferase | 0 | 2 / 0 | Kino-Tannic acid |
| Coagulation factor IX | F9 | P00740 | CHEMBL2016 | Protease | 0 | 1 / 0 | Kino-Tannic acid |
| Equilibrative nucleoside transporter 1 | SLC29A1 | Q99808 | CHEMBL1997 | Electrochemical transporter | 0 | 2 / 0 | Kino-Tannic acid |
| Insulin-like growth factor binding protein 6 | IGFBP6 | P24592 | CHEMBL2139 | Secreted protein | 0 | 1 / 0 | Kino-Tannic acid |
| Insulin-like growth factor binding protein 4 | IGFBP4 | P22692 | CHEMBL2310 | Secreted protein | 0 | 1 / 0 | Kino-Tannic acid |
| Insulin-like growth factor binding protein 5 | IGFBP5 | P24593 | CHEMBL2665 | Secreted protein | 0 | 1 / 0 | Kino-Tannic acid |
| Insulin-like growth factor binding protein 2 | IGFBP2 | P18065 | CHEMBL3088 | Secreted protein | 0 | 1 / 0 | Kino-Tannic acid |
| Insulin-like growth factor binding protein 1 | IGFBP1 | P08833 | CHEMBL4178 | Secreted protein | 0 | 1 / 0 | Kino-Tannic acid |
| Urokinase-type plasminogen activator | PLAU | P00749 | CHEMBL3286 | Protease | 0 | 2 / 0 | Kino-Tannic acid |
| Aldose reductase | AKR1B1 | P15121 | CHEMBL1900 | Enzyme | 0 | 8 / 22 | Kino-Tannic acid |
| Proto-oncogene c-JUN | JUN | P05412 | CHEMBL4977 | Transcription factor | 0 | 1 / 0 | Kino-Tannic acid |
| G protein-coupled receptor kinase 7 | GRK7 | Q8WTQ7 | CHEMBL1075133 | Kinase | 0 | 1 / 0 | Kino-Tannic acid |
| Beta-adrenergic receptor kinase 2 | GRK3 | P35626 | CHEMBL1075166 | Kinase | 0 | 1 / 0 | Kino-Tannic acid |
| G-protein coupled receptor kinase 2 | GRK2 | P25098 | CHEMBL4079 | Kinase | 0 | 1 / 0 | Kino-Tannic acid |
| Rhodopsin kinase | GRK1 | Q15835 | CHEMBL5607 | Kinase | 0 | 1 / 0 | Kino-Tannic acid |
| G protein-coupled receptor kinase 5 | GRK5 | P34947 | CHEMBL5678 | Kinase | 0 | 1 / 0 | Kino-Tannic acid |
| G protein-coupled receptor kinase 4 | GRK4 | P32298 | CHEMBL5861 | Kinase | 0 | 1 / 0 | Kino-Tannic acid |
| Apoptosis regulator Bcl-X | BCL2L1 | Q07817 | CHEMBL4625 | Other ion channel | 0 | 1 / 0 | Kino-Tannic acid |
| Apoptosis regulator Bcl-2 | BCL2 | P10415 | CHEMBL4860 | Other ion channel | 0 | 1 / 0 | Kino-Tannic acid |
| Bcl-2-related protein A1 | BCL2A1 | Q16548 | CHEMBL6044 | Unclassified protein | 0 | 1 / 0 | Kino-Tannic acid |
| Histone deacetylase 3 | HDAC3 | O15379 | CHEMBL1829 | Eraser | 0 | 0 / 1 | Kino-Tannic acid |
| Histone deacetylase 6 | HDAC6 | Q9UBN7 | CHEMBL1865 | Eraser | 0 | 0 / 1 | Kino-Tannic acid |
| Histone deacetylase 2 | HDAC2 | Q92769 | CHEMBL1937 | Eraser | 0 | 0 / 1 | Kino-Tannic acid |
| Insulin-like growth factor I receptor | IGF1R | P08069 | CHEMBL1957 | Kinase | 0 | 0 / 3 | Kino-Tannic acid |
| Serine/threonine-protein kinase Aurora-B | AURKB | Q96GD4 | CHEMBL2185 | Kinase | 0 | 0 / 3 | Kino-Tannic acid |
| Tyrosine-protein kinase SRC | SRC | P12931 | CHEMBL267 | Kinase | 0 | 0 / 3 | Kino-Tannic acid |
| Focal adhesion kinase 1 | PTK2 | Q05397 | CHEMBL2695 | Kinase | 0 | 0 / 3 | Kino-Tannic acid |
| Vascular endothelial growth factor receptor 2 | KDR | P35968 | CHEMBL279 | Kinase | 0 | 0 / 3 | Kino-Tannic acid |
| Serine/threonine-protein kinase PLK1 | PLK1 | P53350 | CHEMBL3024 | Kinase | 0 | 0 / 3 | Kino-Tannic acid |
| Histone deacetylase 8 | HDAC8 | Q9BY41 | CHEMBL3192 | Eraser | 0 | 0 / 1 | Kino-Tannic acid |
| Protein kinase N1 | PKN1 | Q16512 | CHEMBL3384 | Kinase | 0 | 0 / 2 | Kino-Tannic acid |
| Hepatocyte growth factor receptor | MET | P08581 | CHEMBL3717 | Kinase | 0 | 0 / 3 | Kino-Tannic acid |
| Serine/threonine-protein kinase NEK2 | NEK2 | P51955 | CHEMBL3835 | Kinase | 0 | 0 / 2 | Kino-Tannic acid |
| ALK tyrosine kinase receptor | ALK | Q9UM73 | CHEMBL4247 | Kinase | 0 | 0 / 2 | Kino-Tannic acid |
| Serine/threonine-protein kinase AKT | AKT1 | P31749 | CHEMBL4282 | Kinase | 0 | 0 / 3 | Kino-Tannic acid |
| Serine/threonine-protein kinase NEK6 | NEK6 | Q9HC98 | CHEMBL4309 | Kinase | 0 | 0 / 2 | Kino-Tannic acid |
| Tyrosine-protein kinase receptor UFO | AXL | P30530 | CHEMBL4895 | Kinase | 0 | 0 / 2 | Kino-Tannic acid |
| Histone deacetylase 10 | HDAC10 | Q969S8 | CHEMBL5103 | Eraser | 0 | 0 / 1 | Kino-Tannic acid |
| NUAK family SNF1-like kinase 1 | NUAK1 | O60285 | CHEMBL5784 | Kinase | 0 | 0 / 3 | Kino-Tannic acid |
| PI3-kinase p110-alpha subunit | PIK3CA | P42336 | CHEMBL4005 | Enzyme | 0 | 1 / 0 | Kino-Tannic acid |
| Cytochrome P450 2C19 | CYP2C19 | P33261 | CHEMBL3622 | Cytochrome P450 | 0 | 0 / 1 | Kino-Tannic acid |
| Solute carrier family 22 member 6 (by homology) | SLC22A6 | Q4U2R8 | CHEMBL1641347 | Electrochemical transporter | 0 | 0 / 1 | Kino-Tannic acid |
| NADPH oxidase 4 | NOX4 | Q9NPH5 | CHEMBL1250375 | Enzyme | 0 | 0 / 6 | Kino-Tannic acid |
| Vasopressin V2 receptor | AVPR2 | P30518 | CHEMBL1790 | Family A G protein-coupled receptor | 0 | 0 / 1 | Kino-Tannic acid |
| Monoamine oxidase A | MAOA | P21397 | CHEMBL1951 | Oxidoreductase | 0 | 0 / 2 | Kino-Tannic acid |
| Cytochrome P450 19A1 | CYP19A1 | P11511 | CHEMBL1978 | Cytochrome P450 | 0 | 0 / 1 | Kino-Tannic acid |
| Epidermal growth factor receptor erbB1 | EGFR | P00533 | CHEMBL203 | Kinase | 0 | 0 / 2 | Kino-Tannic acid |
| Arachidonate 5-lipoxygenase | ALOX5 | P09917 | CHEMBL215 | Oxidoreductase | 0 | 0 / 4 | Kino-Tannic acid |
| Dopamine D4 receptor | DRD4 | P21917 | CHEMBL219 | Family A G protein-coupled receptor | 0 | 0 / 1 | Kino-Tannic acid |
| Myeloperoxidase | MPO | P05164 | CHEMBL2439 | Enzyme | 0 | 0 / 1 | Kino-Tannic acid |
| PI3-kinase p85-alpha subunit | PIK3R1 | P27986 | CHEMBL2506 | Enzyme | 0 | 0 / 1 | Kino-Tannic acid |
| Death-associated protein kinase 1 | DAPK1 | P53355 | CHEMBL2558 | Kinase | 0 | 0 / 2 | Kino-Tannic acid |
| Liver glycogen phosphorylase | PYGL | P06737 | CHEMBL2568 | Enzyme | 0 | 0 / 1 | Kino-Tannic acid |
| Glycogen synthase kinase-3 beta | GSK3B | P49841 | CHEMBL262 | Kinase | 0 | 0 / 3 | Kino-Tannic acid |
| Estradiol 17-beta-dehydrogenase 2 | HSD17B2 | P37059 | CHEMBL2789 | Enzyme | 0 | 0 / 2 | Kino-Tannic acid |
| Matrix metalloproteinase 13 | MMP13 | P45452 | CHEMBL280 | Protease | 0 | 0 / 1 | Kino-Tannic acid |
| Matrix metalloproteinase 3 | MMP3 | P08254 | CHEMBL283 | Protease | 0 | 0 / 1 | Kino-Tannic acid |
| Carbonic anhydrase III | CA3 | P07451 | CHEMBL2885 | Lyase | 0 | 0 / 2 | Kino-Tannic acid |
| Arachidonate 15-lipoxygenase | ALOX15 | P16050 | CHEMBL2903 | Enzyme | 0 | 0 / 4 | Kino-Tannic acid |
| Cyclin-dependent kinase 1 | CDK1 | P06493 | CHEMBL308 | Kinase | 0 | 0 / 1 | Kino-Tannic acid |
| Matrix metalloproteinase 9 | MMP9 | P14780 | CHEMBL321 | Protease | 0 | 0 / 2 | Kino-Tannic acid |
| Matrix metalloproteinase 2 | MMP2 | P08253 | CHEMBL333 | Protease | 0 | 0 / 2 | Kino-Tannic acid |
| Carbonic anhydrase XIV | CA14 | Q9ULX7 | CHEMBL3510 | Lyase | 0 | 0 / 4 | Kino-Tannic acid |
| Casein kinase II alpha | CSNK2A1 | P68400 | CHEMBL3629 | Kinase | 0 | 0 / 2 | Kino-Tannic acid |
| Arachidonate 12-lipoxygenase | ALOX12 | P18054 | CHEMBL3687 | Enzyme | 0 | 0 / 3 | Kino-Tannic acid |
| Carbonic anhydrase XIII (by homology) | CA13 | Q8N1Q1 | CHEMBL3912 | Lyase | 0 | 0 / 4 | Kino-Tannic acid |
| Interleukin-8 receptor A | CXCR1 | P25024 | CHEMBL4029 | Family A G protein-coupled receptor | 0 | 0 / 1 | Kino-Tannic acid |
| CaM kinase II beta | CAMK2B | Q13554 | CHEMBL4121 | Kinase | 0 | 0 / 1 | Kino-Tannic acid |
| P-glycoprotein 1 | ABCB1 | P08183 | CHEMBL4302 | Primary active transporter | 0 | 0 / 6 | Kino-Tannic acid |
| Phospholipase A2 group 1B | PLA2G1B | P04054 | CHEMBL4426 | Enzyme | 0 | 0 / 1 | Kino-Tannic acid |
| Carbonic anhydrase VA | CA5A | P35218 | CHEMBL4789 | Lyase | 0 | 0 / 4 | Kino-Tannic acid |
| Cytochrome P450 1B1 | CYP1B1 | Q16678 | CHEMBL4878 | Cytochrome P450 | 0 | 0 / 7 | Kino-Tannic acid |
| ATP-binding cassette sub-family G member 2 | ABCG2 | Q9UNQ0 | CHEMBL5393 | Primary active transporter | 0 | 0 / 10 | Kino-Tannic acid |
| Aldo-keto reductase family 1 member C2 (by homology) | AKR1C2 | P52895 | CHEMBL5847 | Enzyme | 0 | 0 / 1 | Kino-Tannic acid |
| Aldo-keto reductase family 1 member C1 (by homology) | AKR1C1 | Q04828 | CHEMBL5905 | Enzyme | 0 | 0 / 1 | Kino-Tannic acid |
| Aldo-keto-reductase family 1 member C3 (by homology) | AKR1C3 | P42330 | CHEMBL4681 | Enzyme | 0 | 0 / 1 | Kino-Tannic acid |
| Aldo-keto reductase family 1 member C4 (by homology) | AKR1C4 | P17516 | CHEMBL4999 | Enzyme | 0 | 0 / 1 | Kino-Tannic acid |
| Adenosine A2a receptor (by homology) | ADORA2A | P29274 | CHEMBL251 | Family A G protein-coupled receptor | 0 | 0 / 2 | Kino-Tannic acid |
| Adenosine A1 receptor (by homology) | ADORA1 | P30542 | CHEMBL226 | Family A G protein-coupled receptor | 0 | 0 / 3 | Kino-Tannic acid |
| Aldehyde reductase (by homology) | AKR1A1 | P14550 | CHEMBL2246 | Enzyme | 0 | 0 / 1 | Kino-Tannic acid |
| Transketolase | TKT | P29401 | CHEMBL4983 | Enzyme | 0.189728914 | 28 / 39 | Thiamine |
| 15-hydroxyprostaglandin dehydrogenase [NAD+] | HPGD | P15428 | CHEMBL1293255 | Enzyme | 0.053556076 | 51 / 0 | Thiamine |
| Carbonic anhydrase II | CA2 | P00918 | CHEMBL205 | Lyase | 0.053556076 | 49 / 0 | Thiamine |
| Carbonic anhydrase I | CA1 | P00915 | CHEMBL261 | Lyase | 0.053556076 | 39 / 0 | Thiamine |
| Hydroxycarboxylic acid receptor 2 | HCAR2 | Q8TDS4 | CHEMBL3785 | Family A G protein-coupled receptor | 0.053556076 | 33 / 0 | Thiamine |
| Aldo-keto-reductase family 1 member C3 | AKR1C3 | P42330 | CHEMBL4681 | Enzyme | 0.053556076 | 121 / 0 | Thiamine |
| Bile acid receptor FXR | NR1H4 | Q96RI1 | CHEMBL2047 | Nuclear receptor | 0.053556076 | 30 / 0 | Thiamine |
| ADAMTS5 | ADAMTS5 | Q9UNA0 | CHEMBL2285 | Protease | 0.053556076 | 48 / 0 | Thiamine |
| Matrix metalloproteinase 1 | MMP1 | P03956 | CHEMBL332 | Protease | 0.053556076 | 26 / 0 | Thiamine |
| Matrix metalloproteinase 14 | MMP14 | P50281 | CHEMBL3869 | Protease | 0.053556076 | 33 / 0 | Thiamine |
| Cytochrome P450 26B1 | CYP26B1 | Q9NR63 | CHEMBL3713687 | Cytochrome P450 | 0.053556076 | 2 / 0 | Thiamine |
| p53-binding protein Mdm-2 | MDM2 | Q00987 | CHEMBL5023 | Other nuclear protein | 0.053556076 | 56 / 0 | Thiamine |
| Cytochrome P450 26A1 | CYP26A1 | O43174 | CHEMBL5141 | Cytochrome P450 | 0.053556076 | 4 / 0 | Thiamine |
| Peroxisome proliferator-activated receptor gamma | PPARG | P37231 | CHEMBL235 | Nuclear receptor | 0.053556076 | 202 / 0 | Thiamine |
| Peroxisome proliferator-activated receptor alpha | PPARA | Q07869 | CHEMBL239 | Nuclear receptor | 0.053556076 | 137 / 0 | Thiamine |
| Peroxisome proliferator-activated receptor delta | PPARD | Q03181 | CHEMBL3979 | Nuclear receptor | 0.053556076 | 36 / 0 | Thiamine |
| Macrophage colony stimulating factor receptor (by homology) | CSF1R | P07333 | CHEMBL1844 | Kinase | 0.053556076 | 6 / 0 | Thiamine |
| Methionine aminopeptidase 2 | METAP2 | P50579 | CHEMBL3922 | Protease | 0.053556076 | 32 / 0 | Thiamine |
| G-protein coupled receptor 35 | GPR35 | Q9HC97 | CHEMBL1293267 | Family A G protein-coupled receptor | 0.053556076 | 38 / 0 | Thiamine |
| Prostanoid EP1 receptor | PTGER1 | P34995 | CHEMBL1811 | Family A G protein-coupled receptor | 0.053556076 | 263 / 0 | Thiamine |
| Plasma retinol-binding protein | RBP4 | P02753 | CHEMBL3100 | Secreted protein | 0.053556076 | 10 / 0 | Thiamine |
| Metabotropic glutamate receptor 2 | GRM2 | Q14416 | CHEMBL5137 | Family C G protein-coupled receptor | 0.053556076 | 8 / 0 | Thiamine |
| Metabotropic glutamate receptor 3 | GRM3 | Q14832 | CHEMBL2888 | Family C G protein-coupled receptor | 0.053556076 | 1 / 0 | Thiamine |
| Glutamate [NMDA] receptor PROTEIN | GRIN1 | Q05586 | CHEMBL2015 | Ligand-gated ion channel | 0.053556076 | 18 / 0 | Thiamine |
| Muscarinic acetylcholine receptor M3 | CHRM3 | P20309 | CHEMBL245 | Family A G protein-coupled receptor | 0.053556076 | 12 / 0 | Thiamine |
| Induced myeloid leukemia cell differentiation protein Mcl-1 | MCL1 | Q07820 | CHEMBL4361 | Other cytosolic protein | 0.053556076 | 57 / 0 | Thiamine |
| Cathepsin (V and K) | CTSV | O60911 | CHEMBL3272 | Protease | 0.053556076 | 7 / 0 | Thiamine |
| Cathepsin L | CTSL | P07711 | CHEMBL3837 | Protease | 0.053556076 | 23 / 0 | Thiamine |
| Apoptosis regulator Bcl-2 | BCL2 | P10415 | CHEMBL4860 | Other ion channel | 0.053556076 | 20 / 0 | Thiamine |
| Lysine-specific demethylase 4C | KDM4C | Q9H3R0 | CHEMBL6175 | Eraser | 0.053556076 | 25 / 0 | Thiamine |
| Carbonic anhydrase XII | CA12 | O43570 | CHEMBL3242 | Lyase | 0.989975593 | 20 / 43 | Umbelliferone |
| Carbonic anhydrase IX | CA9 | Q16790 | CHEMBL3594 | Lyase | 0.989975593 | 18 / 57 | Umbelliferone |
| Epidermal growth factor receptor erbB1 | EGFR | P00533 | CHEMBL203 | Kinase | 0.265882416 | 14 / 3 | Umbelliferone |
| Carbonic anhydrase VII | CA7 | P43166 | CHEMBL2326 | Lyase | 0.265882416 | 8 / 23 | Umbelliferone |
| Xanthine dehydrogenase | XDH | P47989 | CHEMBL1929 | Oxidoreductase | 0.225011513 | 3 / 2 | Umbelliferone |
| Carbonic anhydrase VA | CA5A | P35218 | CHEMBL4789 | Lyase | 0.191771802 | 4 / 11 | Umbelliferone |
| Carbonic anhydrase II | CA2 | P00918 | CHEMBL205 | Lyase | 0.16664584 | 44 / 9 | Umbelliferone |
| Carbonic anhydrase III | CA3 | P07451 | CHEMBL2885 | Lyase | 0.16664584 | 4 / 10 | Umbelliferone |
| Estradiol 17-beta-dehydrogenase 3 | HSD17B3 | P37058 | CHEMBL4234 | Enzyme | 0.158397607 | 10 / 20 | Umbelliferone |
| Carbonic anhydrase I | CA1 | P00915 | CHEMBL261 | Lyase | 0.158397607 | 16 / 22 | Umbelliferone |
| Carbonic anhydrase XIV | CA14 | Q9ULX7 | CHEMBL3510 | Lyase | 0.150097811 | 7 / 18 | Umbelliferone |
| Aldose reductase | AKR1B1 | P15121 | CHEMBL1900 | Enzyme | 0.141787381 | 2 / 12 | Umbelliferone |
| Monoamine oxidase A | MAOA | P21397 | CHEMBL1951 | Oxidoreductase | 0.133391038 | 0 / 42 | Umbelliferone |
| G-protein coupled receptor 35 | GPR35 | Q9HC97 | CHEMBL1293267 | Family A G protein-coupled receptor | 0.133391038 | 0 / 2 | Umbelliferone |
| Cyclin-dependent kinase 4/cyclin D1 | CCND1 CDK4 | P24385 P11802 | CHEMBL1907601 | Kinase | 0.133391038 | 0 / 1 | Umbelliferone |
| Platelet-derived growth factor receptor beta | PDGFRB | P09619 | CHEMBL1913 | Kinase | 0.133391038 | 0 / 1 | Umbelliferone |
| Vascular endothelial growth factor receptor 3 | FLT4 | P35916 | CHEMBL1955 | Kinase | 0.133391038 | 0 / 1 | Umbelliferone |
| Insulin-like growth factor I receptor | IGF1R | P08069 | CHEMBL1957 | Kinase | 0.133391038 | 0 / 2 | Umbelliferone |
| Insulin receptor | INSR | P06213 | CHEMBL1981 | Kinase | 0.133391038 | 0 / 1 | Umbelliferone |
| Cyclin-dependent kinase 2/cyclin A | CDK2 CCNA1 CCNA2 | P24941 P78396 P20248 | CHEMBL2094128 | Other cytosolic protein | 0.133391038 | 0 / 1 | Umbelliferone |
| Focal adhesion kinase 1 | PTK2 | Q05397 | CHEMBL2695 | Kinase | 0.133391038 | 0 / 1 | Umbelliferone |
| Serine/threonine-protein kinase PLK1 | PLK1 | P53350 | CHEMBL3024 | Kinase | 0.133391038 | 0 / 2 | Umbelliferone |
| Casein kinase II alpha | CSNK2A1 | P68400 | CHEMBL3629 | Kinase | 0.133391038 | 0 / 2 | Umbelliferone |
| Hepatocyte growth factor receptor | MET | P08581 | CHEMBL3717 | Kinase | 0.133391038 | 0 / 2 | Umbelliferone |
| Tyrosine-protein kinase TIE-2 | TEK | Q02763 | CHEMBL4128 | Kinase | 0.133391038 | 0 / 1 | Umbelliferone |
| Serine/threonine-protein kinase AKT | AKT1 | P31749 | CHEMBL4282 | Kinase | 0.133391038 | 0 / 1 | Umbelliferone |
| Beta-secretase 1 | BACE1 | P56817 | CHEMBL4822 | Protease | 0.133391038 | 0 / 4 | Umbelliferone |
| Mitogen-activated protein kinase kinase kinase 8 | MAP3K8 | P41279 | CHEMBL4899 | Kinase | 0.133391038 | 0 / 1 | Umbelliferone |
| Ephrin receptor | EPHB4 | P54760 | CHEMBL5147 | Kinase | 0.133391038 | 0 / 1 | Umbelliferone |
| Heat shock 70 kDa protein 1 | HSPA1A | P0DMV8 | CHEMBL5460 | Other cytosolic protein | 0.133391038 | 0 / 1 | Umbelliferone |
| NUAK family SNF1-like kinase 1 | NUAK1 | O60285 | CHEMBL5784 | Kinase | 0.133391038 | 0 / 1 | Umbelliferone |
| Tyrosine-protein kinase FGR (by homology) | FGR | P09769 | CHEMBL4454 | Kinase | 0.133391038 | 0 / 1 | Umbelliferone |
| Tyrosine-protein kinase Lyn (by homology) | LYN | P07948 | CHEMBL3905 | Kinase | 0.133391038 | 0 / 1 | Umbelliferone |
| Myoglobin | MB | P02144 | CHEMBL2406892 | Unclassified protein | 0.133391038 | 1 / 0 | Umbelliferone |
| Macrophage migration inhibitory factor | MIF | P14174 | CHEMBL2085 | Enzyme | 0.133391038 | 5 / 5 | Umbelliferone |
| Ribonuclease H1 | RNASEH1 | O60930 | CHEMBL5893 | Enzyme | 0.12507596 | 1 / 0 | Umbelliferone |
| Cytochrome P450 1A2 | CYP1A2 | P05177 | CHEMBL3356 | Cytochrome P450 | 0.12507596 | 1 / 3 | Umbelliferone |
| D-amino-acid oxidase | DAO | P14920 | CHEMBL5485 | Enzyme | 0.12507596 | 17 / 1 | Umbelliferone |
| Glutathione reductase | GSR | P00390 | CHEMBL2755 | Oxidoreductase | 0.12507596 | 0 / 1 | Umbelliferone |
| Quinone reductase 2 | NQO2 | P16083 | CHEMBL3959 | Enzyme | 0.12507596 | 1 / 0 | Umbelliferone |
| Carbonic anhydrase IV | CA4 | P22748 | CHEMBL3729 | Lyase | 0.12507596 | 4 / 11 | Umbelliferone |
| Carbonic anhydrase VI | CA6 | P23280 | CHEMBL3025 | Lyase | 0.12507596 | 2 / 9 | Umbelliferone |
| Carbonic anhydrase XIII | CA13 | Q8N1Q1 | CHEMBL3912 | Lyase | 0.12507596 | 1 / 26 | Umbelliferone |
| Estrogen receptor alpha | ESR1 | P03372 | CHEMBL206 | Nuclear receptor | 0.12507596 | 14 / 45 | Umbelliferone |
| Estrogen receptor beta | ESR2 | Q92731 | CHEMBL242 | Nuclear receptor | 0.12507596 | 16 / 65 | Umbelliferone |
| Dihydropteridine reductase | QDPR | P09417 | CHEMBL3730 | Enzyme | 0.12507596 | 1 / 0 | Umbelliferone |
| Poly [ADP-ribose] polymerase-1 | PARP1 | P09874 | CHEMBL3105 | Enzyme | 0.12507596 | 4 / 0 | Umbelliferone |
| Cyclin-dependent kinase 2/cyclin E1 | CCNE1 CDK2 | P24864 P24941 | CHEMBL1907605 | Kinase | 0.12507596 | 1 / 0 | Umbelliferone |
| Protein tyrosine kinase 2 beta | PTK2B | Q14289 | CHEMBL5469 | Kinase | 0.12507596 | 1 / 0 | Umbelliferone |
| Carbonic anhydrase VB | CA5B | Q9Y2D0 | CHEMBL3969 | Lyase | 0.12507596 | 3 / 12 | Umbelliferone |
| Indoleamine 2,3-dioxygenase | IDO1 | P14902 | CHEMBL4685 | Enzyme | 0.12507596 | 8 / 0 | Umbelliferone |
| Serine/threonine-protein kinase Aurora-B | AURKB | Q96GD4 | CHEMBL2185 | Kinase | 0.12507596 | 2 / 3 | Umbelliferone |
| Glycogen synthase kinase-3 beta | GSK3B | P49841 | CHEMBL262 | Kinase | 0.12507596 | 4 / 1 | Umbelliferone |
| Vascular endothelial growth factor receptor 2 | KDR | P35968 | CHEMBL279 | Kinase | 0.12507596 | 1 / 2 | Umbelliferone |
| Aldo-keto-reductase family 1 member C3 | AKR1C3 | P42330 | CHEMBL4681 | Enzyme | 0.116739032 | 0 / 1 | Umbelliferone |
| Aldo-keto reductase family 1 member C1 | AKR1C1 | Q04828 | CHEMBL5905 | Enzyme | 0.116739032 | 0 / 1 | Umbelliferone |
| Alpha-synuclein | SNCA | P37840 | CHEMBL6152 | Unclassified protein | 0.116739032 | 5 / 0 | Umbelliferone |
| Methionine aminopeptidase 2 | METAP2 | P50579 | CHEMBL3922 | Protease | 0.116739032 | 4 / 0 | Umbelliferone |
| Aldehyde dehydrogenase | ALDH2 | P05091 | CHEMBL1935 | Oxidoreductase | 0.116739032 | 0 / 2 | Umbelliferone |
| Succinate semialdehyde dehydrogenase | ALDH5A1 | P51649 | CHEMBL1911 | Oxidoreductase | 0.116739032 | 1 / 0 | Umbelliferone |
| Gamma-amino-N-butyrate transaminase | ABAT | P80404 | CHEMBL2044 | Transferase | 0.116739032 | 2 / 0 | Umbelliferone |
| Acetylcholinesterase | ACHE | P22303 | CHEMBL220 | Hydrolase | 0.116739032 | 0 / 23 | Umbelliferone |
| Nuclear factor NF-kappa-B p105 subunit | NFKB1 | P19838 | CHEMBL3251 | Other cytosolic protein | 0.116739032 | 0 / 2 | Umbelliferone |
| Voltage-gated potassium channel subunit Kv1.3 | KCNA3 | P22001 | CHEMBL4633 | Voltage-gated ion channel | 0.116739032 | 0 / 8 | Umbelliferone |

**Supplementary Table S8. Disgenet-predicted disease targets for Ulcerative colitis**

| **Disease UMLSCUI** | **disease** | **gene** | **Num Variants Associated To Gene** | **Number Pmids With Chems Included In Evidence** | **lastRef** | **Gene Full Name** | **geneDSI** |
| --- | --- | --- | --- | --- | --- | --- | --- |
| C0009324 | Ulcerative Colitis | **STAT3** | 214 | 0 | 2010 | signal transducer and activator of transcription 3 | 0.32 |
| C0009324 | Ulcerative Colitis | **IL23R** | 28 | 0 | 2010 | interleukin 23 receptor | 0.46 |
| C0009324 | Ulcerative Colitis | **TNFSF15** | 8 | 0 | 2010 | TNF superfamily member 15 | 0.53 |
| C0009324 | Ulcerative Colitis | **TNF** | 9 | 1 | 2012 | tumor necrosis factor | 0.23 |
| C0009324 | Ulcerative Colitis | **NOD2** | 442 | 0 | 2010 | nucleotide binding oligomerization domain containing 2 | 0.41 |
| C0009324 | Ulcerative Colitis | **JAK2** | 30 | 0 | 2011 | Janus kinase 2 | 0.36 |
| C0009324 | Ulcerative Colitis | **IL10** | 37 | 0 | 2010 | interleukin 10 | 0.27 |
| C0009324 | Ulcerative Colitis | **ALB** | 45 | 0 | 2022 | albumin | 0.28 |
| C0009324 | Ulcerative Colitis | **MPO** | 18 | 2 | 2013 | myeloperoxidase | 0.36 |
| C0009324 | Ulcerative Colitis | **CDH1** | 1632 | 0 | 2009 | cadherin 1 | 0.32 |
| C0009324 | Ulcerative Colitis | **LTF** | 2 | 0 | 2022 | lactotransferrin | 0.4 |
| C0009324 | Ulcerative Colitis | **IL1B** | 4 | 2 | 2012 | interleukin 1 beta | 0.27 |
| C0009324 | Ulcerative Colitis | **CCL20** | 0 | 0 | 2005 | C-C motif chemokine ligand 20 | 0.45 |
| C0009324 | Ulcerative Colitis | **APOA4** | 3 | 0 | 2022 | apolipoprotein A4 | 0.51 |
| C0009324 | Ulcerative Colitis | **NKX2-3** | 2 | 0 | 2010 | NK2 homeobox 3 | 0.68 |
| C0009324 | Ulcerative Colitis | **IL1R2** | 0 | 0 | 2011 | interleukin 1 receptor type 2 | 0.36 |
| C0009324 | Ulcerative Colitis | **IL12B** | 92 | 0 | 2011 | interleukin 12B | 0.32 |
| C0009324 | Ulcerative Colitis | **HNF4A** | 214 | 0 | 2009 | hepatocyte nuclear factor 4 alpha | 0.44 |
| C0009324 | Ulcerative Colitis | **SLC11A1** | 3 | 0 | 2006 | solute carrier family 11 member 1 | 0.47 |
| C0009324 | Ulcerative Colitis | **MMP9** | 38 | 1 | 2012 | matrix metallopeptidase 9 | 0.29 |
| C0009324 | Ulcerative Colitis | **FCGR2A** | 8 | 0 | 2010 | Fc gamma receptor IIa | 0.45 |
| C0009324 | Ulcerative Colitis | **ICAM1** | 7 | 0 | 2004 | intercellular adhesion molecule 1 | 0.33 |
| C0009324 | Ulcerative Colitis | **DEFA5** | 1 | 0 | 2010 | defensin alpha 5 | 0.67 |
| C0009324 | Ulcerative Colitis | **CASP3** | 1 | 1 | 2013 | caspase 3 | 0.36 |
| C0009324 | Ulcerative Colitis | **HP** | 3 | 0 | 2022 | haptoglobin | 0.36 |
| C0009324 | Ulcerative Colitis | **ABCB1** | 16 | 0 |  | ATP binding cassette subfamily B member 1 | 0.33 |
| C0009324 | Ulcerative Colitis | **HLA-DRB1** | 19 | 0 | 2015 | major histocompatibility complex, class II, DR beta 1 | 0.38 |
| C0009324 | Ulcerative Colitis | **IL1RN** | 96 | 0 |  | interleukin 1 receptor antagonist | 0.35 |
| C0009324 | Ulcerative Colitis | **SLC26A3** | 101 | 0 | 2009 | solute carrier family 26 member 3 | 0.56 |
| C0009324 | Ulcerative Colitis | **RELA** | 8 | 1 | 2005 | RELA proto-oncogene, NF-kB subunit | 0.4 |
| C0009324 | Ulcerative Colitis | **CCNY** | 4 | 0 | 2008 | cyclin Y | 0.66 |
| C0009324 | Ulcerative Colitis | **GC** | 10 | 0 |  | GC vitamin D binding protein | 0.45 |
| C0009324 | Ulcerative Colitis | **GSTM1** | 3 | 0 |  | glutathione S-transferase mu 1 | 0.37 |
| C0009324 | Ulcerative Colitis | **GSTT1** | 0 | 0 |  | glutathione S-transferase theta 1 | 0.39 |
| C0009324 | Ulcerative Colitis | **C3** | 158 | 0 | 2022 | complement C3 | 0.41 |
| C0009324 | Ulcerative Colitis | **SPHK1** | 0 | 1 | 2013 | sphingosine kinase 1 | 0.46 |
| C0009324 | Ulcerative Colitis | **IRGM** | 9 | 0 | 2010 | immunity related GTPase M | 0.61 |
| C0009324 | Ulcerative Colitis | **PTPN2** | 20 | 0 | 2008 | protein tyrosine phosphatase non-receptor type 2 | 0.5 |
| C0009324 | Ulcerative Colitis | **CARD9** | 207 | 0 | 2010 | caspase recruitment domain family member 9 | 0.52 |
| C0009324 | Ulcerative Colitis | **CXCR2** | 3 | 0 | 2011 | C-X-C motif chemokine receptor 2 | 0.43 |
| C0009324 | Ulcerative Colitis | **IRF5** | 18 | 0 | 2011 | interferon regulatory factor 5 | 0.42 |
| C0009324 | Ulcerative Colitis | **MST1** | 3 | 0 | 2010 | macrophage stimulating 1 | 0.47 |
| C0009324 | Ulcerative Colitis | **PTGER4** | 4 | 0 |  | prostaglandin E receptor 4 | 0.47 |
| C0009324 | Ulcerative Colitis | **CFB** | 66 | 0 | 2022 | complement factor B | 0.46 |
| C0009324 | Ulcerative Colitis | **SELL** | 2 | 0 |  | selectin L | 0.43 |
| C0009324 | Ulcerative Colitis | **GHRL** | 4 | 0 | 2009 | ghrelin and obestatin prepropeptide | 0.37 |
| C0009324 | Ulcerative Colitis | **BTNL2** | 41 | 0 |  | butyrophilin like 2 | 0.53 |
| C0009324 | Ulcerative Colitis | **VCAM1** | 0 | 0 | 2004 | vascular cell adhesion molecule 1 | 0.38 |
| C0009324 | Ulcerative Colitis | **IL18RAP** | 10 | 0 | 2010 | interleukin 18 receptor accessory protein | 0.61 |
| C0009324 | Ulcerative Colitis | **ECM1** | 17 | 0 | 2008 | extracellular matrix protein 1 | 0.53 |
| C0009324 | Ulcerative Colitis | **CCR6** | 9 | 0 | 2005 | C-C motif chemokine receptor 6 | 0.45 |
| C0009324 | Ulcerative Colitis | **LAMB1** | 30 | 0 | 2009 | laminin subunit beta 1 | 0.55 |
| C0009324 | Ulcerative Colitis | **ORMDL3** | 4 | 0 | 2010 | ORMDL sphingolipid biosynthesis regulator 3 | 0.62 |
| C0009324 | Ulcerative Colitis | **ARPC2** | 3 | 0 | 2008 | actin related protein 2/3 complex subunit 2 | 0.7 |
| C0009324 | Ulcerative Colitis | **HERC2** | 133 | 0 | 2008 | HECT and RLD domain containing E3 ubiquitin protein ligase 2 | 0.54 |
| C0009324 | Ulcerative Colitis | **RIPK2** | 3 | 0 | 2010 | receptor interacting serine/threonine kinase 2 | 0.57 |
| C0009324 | Ulcerative Colitis | **MTRR** | 142 | 0 |  | 5-methyltetrahydrofolate-homocysteine methyltransferase reductase | 0.47 |
| C0009324 | Ulcerative Colitis | **CDH3** | 110 | 0 | 2009 | cadherin 3 | 0.48 |
| C0009324 | Ulcerative Colitis | **ADCY7** | 5 | 0 | 2017 | adenylate cyclase 7 | 0.6 |
| C0009324 | Ulcerative Colitis | **CYP24A1** | 92 | 0 |  | cytochrome P450 family 24 subfamily A member 1 | 0.46 |
| C0009324 | Ulcerative Colitis | **IL7R** | 213 | 0 | 2011 | interleukin 7 receptor | 0.46 |
| C0009324 | Ulcerative Colitis | **ICOSLG** | 5 | 0 | 2010 | inducible T cell costimulator ligand | 0.54 |
| C0009324 | Ulcerative Colitis | **GPR65** | 3 | 0 | 2019 | G protein-coupled receptor 65 | 0.63 |
| C0009324 | Ulcerative Colitis | **HLA-DQB1** | 58 | 0 |  | major histocompatibility complex, class II, DQ beta 1 | 0.42 |
| C0009324 | Ulcerative Colitis | **DAP** | 8 | 0 | 2011 | death associated protein | 0.53 |
| C0009324 | Ulcerative Colitis | **LSP1** | 24 | 0 | 2011 | lymphocyte specific protein 1 | 0.62 |
| C0009324 | Ulcerative Colitis | **MASP2** | 62 | 0 | 2003 | MBL associated serine protease 2 | 0.53 |
| C0009324 | Ulcerative Colitis | **PRDM1** | 5 | 0 | 2011 | PR/SET domain 1 | 0.5 |
| C0009324 | Ulcerative Colitis | **GNA12** | 38 | 0 | 2011 | G protein subunit alpha 12 | 0.6 |
| C0009324 | Ulcerative Colitis | **DPEP1** | 15 | 0 | 2022 | dipeptidase 1 | 0.5 |
| C0009324 | Ulcerative Colitis | **IKZF1** | 44 | 0 | 2010 | IKAROS family zinc finger 1 | 0.48 |
| C0009324 | Ulcerative Colitis | **CXCR1** | 5 | 0 | 2011 | C-X-C motif chemokine receptor 1 | 0.48 |
| C0009324 | Ulcerative Colitis | **PSMG1** | 2 | 0 | 2010 | proteasome assembly chaperone 1 | 0.53 |
| C0009324 | Ulcerative Colitis | **KIF21B** | 8 | 0 | 2010 | kinesin family member 21B | 0.65 |

**Supplementary Table S9. GeneCards predicted disease targets for Ulcerative colitis**

| **Gene Symbol** | **Description** | **Category** | **Uniprot ID** | **Gifts** | **GC Id** | **Relevance score** |
| --- | --- | --- | --- | --- | --- | --- |
| TNF | Tumor Necrosis Factor | Protein Coding | P01375 | 65 | GC06P144764 | 49.11486816 |
| H19 | H19 Imprinted Maternally Expressed Transcript | RNA Gene |  | 36 | GC11M001995 | 46.90610123 |
| IL1B | Interleukin 1 Beta | Protein Coding | P01584 | 60 | GC02M112829 | 38.19383621 |
| STAT3 | Signal Transducer And Activator Of Transcription 3 | Protein Coding | P40763 | 67 | GC17M042313 | 35.93743515 |
| TGFB1 | Transforming Growth Factor Beta 1 | Protein Coding | P01137 | 66 | GC19M041301 | 35.2916832 |
| HLA-DRB1 | Major Histocompatibility Complex, Class II, DR Beta 1 | Protein Coding | P01911 | 59 | GC06M097159 | 35.00274658 |
| CXCL8 | C-X-C Motif Chemokine Ligand 8 | Protein Coding | P10145 | 56 | GC04P073740 | 32.43598938 |
| NF-κB | Nuclear Factor Kappa B Subunit 1 | Protein Coding | P19838 | 66 | GC04P102501 | 30.84584999 |
| ABCB1 | ATP Binding Cassette Subfamily B Member 1 | Protein Coding | P08183 | 64 | GC07M087504 | 30.15694809 |
| TP53 | Tumor Protein P53 | Protein Coding | P04637 | 66 | GC17M007661 | 29.38644981 |
| CTLA4 | Cytotoxic T-Lymphocyte Associated Protein 4 | Protein Coding | P16410 | 61 | GC02P204076 | 28.66501236 |
| IL2 | Interleukin 2 | Protein Coding | P60568 | 59 | GC04M122451 | 27.08722687 |
| CTNNB1 | Catenin Beta 1 | Protein Coding | P35222 | 66 | GC03P041194 | 23.70237541 |
| MLH1 | MutL Homolog 1 | Protein Coding | P40692 | 62 | GC03P036993 | 23.2844944 |
| HLA-B | Major Histocompatibility Complex, Class I, B | Protein Coding | P01889 | 59 | GC06M097059 | 22.6858902 |
| LTF | Lactotransferrin | Protein Coding | P02788 | 58 | GC03M046435 | 21.79938126 |
| SMAD4 | SMAD Family Member 4 | Protein Coding | Q13485 | 66 | GC18P051028 | 21.4578495 |
| AKT1 | AKT Serine/Threonine Kinase 1 | Protein Coding | P31749 | 66 | GC14M104769 | 21.33337212 |
| SLC11A1 | Solute Carrier Family 11 Member 1 | Protein Coding | P49279 | 59 | GC02P218382 | 20.83840179 |
| ALB | Albumin | Protein Coding | P02768 | 62 | GC04P073397 | 18.79597092 |
| LINC01672 | Long Intergenic Non-Protein Coding RNA 1672 | RNA Gene |  | 21 | GC01P050860 | 17.99621201 |
| MSH2 | MutS Homolog 2 | Protein Coding | P43246 | 60 | GC02P047403 | 17.57174873 |
| MIR21 | MicroRNA 21 | RNA Gene |  | 33 | GC17P123488 | 17.57157898 |
| CHEK2 | Checkpoint Kinase 2 | Protein Coding | O96017 | 67 | GC22M028687 | 17.53258896 |
| PTEN | Phosphatase And Tensin Homolog | Protein Coding | P60484 | 65 | GC10P112611 | 17.39987946 |
| RELA | RELA Proto-Oncogene, NF-KB Subunit | Protein Coding | Q04206 | 64 | GC11M065653 | 17.34803391 |
| HSPA4 | Heat Shock Protein Family A (Hsp70) Member 4 | Protein Coding | P34932 | 56 | GC05P133062 | 16.70232773 |
| VDR | Vitamin D Receptor | Protein Coding | P11473 | 62 | GC12M047841 | 16.64021683 |
| CDKN2A | Cyclin Dependent Kinase Inhibitor 2A | Protein Coding | Q8N726 | 64 | GC09M021967 | 16.43361855 |
| APC | APC Regulator Of WNT Signaling Pathway | Protein Coding | P25054 | 62 | GC05P112707 | 16.36962318 |
| CD40LG | CD40 Ligand | Protein Coding | P29965 | 63 | GC0XP136649 | 16.29842758 |
| ADA | Adenosine Deaminase | Protein Coding | P00813 | 64 | GC20M044620 | 16.09852791 |
| CPT1A | Carnitine Palmitoyltransferase 1A | Protein Coding | P50416 | 62 | GC11M068754 | 15.60929966 |
| MIR155 | MicroRNA 155 | RNA Gene |  | 31 | GC21P025573 | 15.53923035 |
| MGLL | Monoglyceride Lipase | Protein Coding | Q99685 | 56 | GC03M127689 | 15.31926346 |
| HLA-DQB1 | Major Histocompatibility Complex, Class II, DQ Beta 1 | Protein Coding | P01920 | 55 | GC06M097169 | 15.28769779 |
| KRAS | KRAS Proto-Oncogene, GTPase | Protein Coding | P01116 | 66 | GC12M031027 | 14.97677231 |
| CYP2C19 | Cytochrome P450 Family 2 Subfamily C Member 19 | Protein Coding | P33261 | 57 | GC10P094762 | 14.93804646 |
| JAK2 | Janus Kinase 2 | Protein Coding | O60674 | 66 | GC09P004985 | 14.89556408 |
| SMAD7 | SMAD Family Member 7 | Protein Coding | O15105 | 57 | GC18M048919 | 14.8591547 |
| CERNA3 | Competing Endogenous LncRNA 3 For MiR-645 | RNA Gene |  | 21 | GC08P056352 | 14.55252075 |
| TERT | Telomerase Reverse Transcriptase | Protein Coding | O14746 | 64 | GC05M001253 | 14.54717159 |
| NCF4 | Neutrophil Cytosolic Factor 4 | Protein Coding | Q15080 | 61 | GC22P036860 | 14.35909939 |
| PIK3CD | Phosphatidylinositol-4,5-Bisphosphate 3-Kinase Catalytic Subunit Delta | Protein Coding | O00329 | 66 | GC01P051042 | 13.97309685 |
| CD14 | CD14 Molecule | Protein Coding | P08571 | 59 | GC05M140631 | 13.63690948 |
| HLA-DQA1 | Major Histocompatibility Complex, Class II, DQ Alpha 1 | Protein Coding | P01909 | 55 | GC06P144795 | 13.56350327 |
| MMP3 | Matrix Metallopeptidase 3 | Protein Coding | P08254 | 63 | GC11M102835 | 13.48101234 |
| SOD1 | Superoxide Dismutase 1 | Protein Coding | P00441 | 66 | GC21P031659 | 12.89106083 |
| F2 | Coagulation Factor II, Thrombin | Protein Coding | P00734 | 63 | GC11P048054 | 12.88357735 |
| HMOX1 | Heme Oxygenase 1 | Protein Coding | P09601 | 65 | GC22P035380 | 12.65835953 |
| NFKBIA | NFKB Inhibitor Alpha | Protein Coding | P25963 | 64 | GC14M035401 | 12.48330688 |
| RASGRP1 | RAS Guanyl Releasing Protein 1 | Protein Coding | O95267 | 59 | GC15M038488 | 12.44692421 |
| CYP3A4 | Cytochrome P450 Family 3 Subfamily A Member 4 | Protein Coding | P08684 | 61 | GC07M105725 | 12.40321159 |
| IL2RG | Interleukin 2 Receptor Subunit Gamma | Protein Coding | P31785 | 60 | GC0XM071108 | 12.28787708 |
| CBS | Cystathionine Beta-Synthase | Protein Coding | P35520 | 64 | GC21M043053 | 12.18206215 |
| ERBB2 | Erb-B2 Receptor Tyrosine Kinase 2 | Protein Coding | P04626 | 68 | GC17P039687 | 12.17922974 |
| MET | MET Proto-Oncogene, Receptor Tyrosine Kinase | Protein Coding | P08581 | 67 | GC07P116672 | 11.32883453 |
| TMX2-CTNND1 | TMX2-CTNND1 Readthrough (NMD Candidate) | RNA Gene |  | 25 | GC11P057712 | 11.31734276 |
| TGFBR2 | Transforming Growth Factor Beta Receptor 2 | Protein Coding | P37173 | 65 | GC03P030623 | 11.24274445 |
| BCL2 | BCL2 Apoptosis Regulator | Protein Coding | P10415 | 64 | GC18M063123 | 11.23299217 |
| MIR141 | MicroRNA 141 | RNA Gene |  | 31 | GC12P042129 | 11.18796062 |
| IL1R1 | Interleukin 1 Receptor Type 1 | Protein Coding | P14778 | 60 | GC02P102136 | 11.09755325 |
| CCND1 | Cyclin D1 | Protein Coding | P24385 | 66 | GC11P069641 | 11.08208847 |
| MICA | MHC Class I Polypeptide-Related Sequence A | Protein Coding | Q29983 | 51 | GC06P031399 | 10.95916367 |
| HLA-DRA | Major Histocompatibility Complex, Class II, DR Alpha | Protein Coding | P01903 | 58 | GC06P032439 | 10.91418457 |
| NOD1 | Nucleotide Binding Oligomerization Domain Containing 1 | Protein Coding | Q9Y239 | 56 | GC07M030424 | 10.90171814 |
| CXCR2 | C-X-C Motif Chemokine Receptor 2 | Protein Coding | P25025 | 63 | GC02P218125 | 10.81480598 |
| CTSG | Cathepsin G | Protein Coding | P08311 | 56 | GC14M024573 | 10.79537296 |
| PTPN11 | Protein Tyrosine Phosphatase Non-Receptor Type 11 | Protein Coding | Q06124 | 67 | GC12P112418 | 10.74704647 |
| MIR223 | MicroRNA 223 | RNA Gene |  | 30 | GC0XP066018 | 10.72362137 |
| MIR146B | MicroRNA 146b | RNA Gene |  | 31 | GC10P102436 | 10.72315884 |
| BDNF-AS | BDNF Antisense RNA | RNA Gene |  | 31 | GC11P027466 | 10.62296009 |
| INS | Insulin | Protein Coding | P01308 | 60 | GC11M002159 | 10.57881069 |
| MIR142 | MicroRNA 142 | RNA Gene |  | 31 | GC17M058331 | 10.53050423 |
| CD55 | CD55 Molecule (Cromer Blood Group) | Protein Coding | P08174 | 62 | GC01P207321 | 10.5101366 |
| SI | Sucrase-Isomaltase | Protein Coding | P14410 | 58 | GC03M164978 | 10.42142773 |
| HIF1A | Hypoxia Inducible Factor 1 Subunit Alpha | Protein Coding | Q16665 | 62 | GC14P061695 | 10.33737659 |
| HLA-A | Major Histocompatibility Complex, Class I, A | Protein Coding | P04439 | 59 | GC06P144720 | 10.22826481 |
| RET | Ret Proto-Oncogene | Protein Coding | P07949 | 67 | GC10P044766 | 10.06537914 |
| GREM1 | Gremlin 1, DAN Family BMP Antagonist | Protein Coding | O60565 | 56 | GC15P165907 | 10.02371597 |
| TERC | Telomerase RNA Component | RNA Gene |  | 39 | GC03M169765 | 9.965943336 |
| PRF1 | Perforin 1 | Protein Coding | P14222 | 59 | GC10M070597 | 9.864364624 |
| SELE | Selectin E | Protein Coding | P16581 | 57 | GC01M169722 | 9.770454407 |
| TFF3 | Trefoil Factor 3 | Protein Coding | Q07654 | 53 | GC21M042311 | 9.53553009 |
| MIR34A | MicroRNA 34a | RNA Gene |  | 32 | GC01M015281 | 9.438709259 |
| MIR19A | MicroRNA 19a | RNA Gene |  | 29 | GC13P091695 | 9.434821129 |
| ITGAM | Integrin Subunit Alpha M | Protein Coding | P11215 | 61 | GC16P096773 | 9.320607185 |
| ATM | ATM Serine/Threonine Kinase | Protein Coding | Q13315 | 66 | GC11P108223 | 9.27920723 |
| RHOA | Ras Homolog Family Member A | Protein Coding | P61586 | 63 | GC03M049359 | 9.265026093 |
| MEG3 | Maternally Expressed 3 | RNA Gene |  | 37 | GC14P117888 | 9.221583366 |
| MIR29A | MicroRNA 29a | RNA Gene |  | 31 | GC07M130876 | 9.184449196 |
| HLA-C | Major Histocompatibility Complex, Class I, C | Protein Coding | P10321 | 58 | GC06M097058 | 9.088072777 |
| HAVCR2 | Hepatitis A Virus Cellular Receptor 2 | Protein Coding | Q8TDQ0 | 59 | GC05M157063 | 9.000575066 |
| CXCL1 | C-X-C Motif Chemokine Ligand 1 | Protein Coding | P09341 | 56 | GC04P073869 | 8.996490479 |
| MIR23A | MicroRNA 23a | RNA Gene |  | 30 | GC19M096312 | 8.991211891 |
| GCG | Glucagon | Protein Coding | P01275 | 55 | GC02M162142 | 8.934185982 |
| CP | Ceruloplasmin | Protein Coding | P00450 | 61 | GC03M149162 | 8.925534248 |
| MIR146A | MicroRNA 146a | RNA Gene |  | 31 | GC05P160485 | 8.923511505 |
| CYP2D6 | Cytochrome P450 Family 2 Subfamily D Member 6 | Protein Coding | P10635 | 60 | GC22M042126 | 8.892949104 |
| PEBP1 | Phosphatidylethanolamine Binding Protein 1 | Protein Coding | P30086 | 58 | GC12P118158 | 8.844546318 |
| MIR342 | MicroRNA 342 | RNA Gene |  | 29 | GC14P100109 | 8.824724197 |
| MIR18A | MicroRNA 18a | RNA Gene |  | 28 | GC13P091701 | 8.740675926 |
| STX3 | Syntaxin 3 | Protein Coding | Q13277 | 55 | GC11P059713 | 8.709976196 |
| KRT7 | Keratin 7 | Protein Coding | P08729 | 56 | GC12P052232 | 8.695508957 |
| MIR145 | MicroRNA 145 | RNA Gene |  | 32 | GC05P149430 | 8.688708305 |
| TOR1A | Torsin Family 1 Member A | Protein Coding | O14656 | 59 | GC09M129812 | 8.679722786 |
| SERPINA1 | Serpin Family A Member 1 | Protein Coding | P01009 | 62 | GC14M094376 | 8.667785645 |
| NOP10 | NOP10 Ribonucleoprotein | Protein Coding | Q9NPE3 | 54 | GC15M040534 | 8.64159584 |
| CREB1 | CAMP Responsive Element Binding Protein 1 | Protein Coding | P16220 | 63 | GC02P207529 | 8.634127617 |
| CYLD | CYLD Lysine 63 Deubiquitinase | Protein Coding | Q9NQC7 | 60 | GC16P050742 | 8.586898804 |
| MALAT1 | Metastasis Associated Lung Adenocarcinoma Transcript 1 | RNA Gene |  | 33 | GC11P094626 | 8.549487114 |
| RB1 | RB Transcriptional Corepressor 1 | Protein Coding | P06400 | 62 | GC13P048303 | 8.546415329 |
| IKBKG | Inhibitor Of Nuclear Factor Kappa B Kinase Regulatory Subunit Gamma | Protein Coding | Q9Y6K9 | 60 | GC0XP154541 | 8.529872894 |
| DOCK8 | Dedicator Of Cytokinesis 8 | Protein Coding | Q8NF50 | 58 | GC09P000286 | 8.495676041 |
| CDKN2B | Cyclin Dependent Kinase Inhibitor 2B | Protein Coding | P42772 | 58 | GC09M022002 | 8.421738625 |
| MUC1 | Mucin 1, Cell Surface Associated | Protein Coding | P15941 | 61 | GC01M155185 | 8.340052605 |
| MIR214 | MicroRNA 214 | RNA Gene |  | 31 | GC01M172234 | 8.293404579 |
| G6PC3 | Glucose-6-Phosphatase Catalytic Subunit 3 | Protein Coding | Q9BUM1 | 55 | GC17P044070 | 8.258938789 |
| KLRC4 | Killer Cell Lectin Like Receptor C4 | Protein Coding | O43908 | 48 | GC12M030834 | 8.256343842 |
| GAS5 | Growth Arrest Specific 5 | RNA Gene |  | 33 | GC01M173947 | 8.242789268 |
| SKIC3 | SKI3 Subunit Of Superkiller Complex | Protein Coding | Q6PGP7 | 52 | GC05M095461 | 8.180307388 |
| FCGR2B | Fc Gamma Receptor IIb | Protein Coding | P31994 | 61 | GC01P167729 | 8.179071426 |
| CD3G | CD3 Gamma Subunit Of T-Cell Receptor Complex | Protein Coding | P09693 | 59 | GC11P118344 | 8.162471771 |
| ALOX5 | Arachidonate 5-Lipoxygenase | Protein Coding | P09917 | 62 | GC10P045374 | 8.154673576 |
| FLNA | Filamin A | Protein Coding | P21333 | 61 | GC0XM154348 | 8.148604393 |
| MIR106A | MicroRNA 106a | RNA Gene |  | 28 | GC0XM134505 | 8.13899231 |
| IGF1R | Insulin Like Growth Factor 1 Receptor | Protein Coding | P08069 | 68 | GC15P098648 | 8.100543976 |
| SOD2-OT1 | SOD2 Overlapping Transcript 1 | RNA Gene |  | 21 | GC06M159772 | 8.095186234 |
| TREX1 | Three Prime Repair Exonuclease 1 | Protein Coding | Q9NSU2 | 55 | GC03P059849 | 8.073572159 |
| PCNA | Proliferating Cell Nuclear Antigen | Protein Coding | P12004 | 63 | GC20M005114 | 8.069150925 |
| CYP2C9 | Cytochrome P450 Family 2 Subfamily C Member 9 | Protein Coding | P11712 | 59 | GC10P094938 | 7.999009609 |
| FOS | Fos Proto-Oncogene, AP-1 Transcription Factor Subunit | Protein Coding | P01100 | 64 | GC14P075278 | 7.98929739 |
| HSPD1 | Heat Shock Protein Family D (Hsp60) Member 1 | Protein Coding | P10809 | 62 | GC02M197486 | 7.975208282 |
| MASP2 | MBL Associated Serine Protease 2 | Protein Coding | O00187 | 59 | GC01M015332 | 7.970240593 |
| PYY | Peptide YY | Protein Coding | P10082 | 55 | GC17M043952 | 7.962424278 |
| TPI1 | Triosephosphate Isomerase 1 | Protein Coding | P60174 | 60 | GC12P006867 | 7.947912216 |
| PPIG | Peptidylprolyl Isomerase G | Protein Coding | Q13427 | 52 | GC02P169584 | 7.947005749 |
| FGF10 | Fibroblast Growth Factor 10 | Protein Coding | O15520 | 60 | GC05M044965 | 7.933804512 |
| SLC6A4 | Solute Carrier Family 6 Member 4 | Protein Coding | P31645 | 61 | GC17M030194 | 7.920550346 |
| CXCL5 | C-X-C Motif Chemokine Ligand 5 | Protein Coding | P42830 | 52 | GC04M073995 | 7.913314342 |
| RAF1 | Raf-1 Proto-Oncogene, Serine/Threonine Kinase | Protein Coding | P04049 | 67 | GC03M012583 | 7.855781078 |
| SNHG5 | Small Nucleolar RNA Host Gene 5 | RNA Gene |  | 29 | GC06M085650 | 7.841715336 |
| RN7SL1 | RNA Component Of Signal Recognition Particle 7SL1 | RNA Gene |  | 27 | GC14P049554 | 7.836630821 |
| TUG1 | Taurine Up-Regulated 1 | Protein Coding | A0A6I8PU40 | 34 | GC22P030969 | 7.825580597 |
| MIR27A | MicroRNA 27a | RNA Gene |  | 32 | GC19M096311 | 7.811195374 |
| MIR224 | MicroRNA 224 | RNA Gene |  | 25 | GC0XM151958 | 7.806379318 |
| CYP1A2 | Cytochrome P450 Family 1 Subfamily A Member 2 | Protein Coding | P05177 | 59 | GC15P074748 | 7.804227829 |
| MIR200C | MicroRNA 200c | RNA Gene |  | 31 | GC12P042128 | 7.802313805 |
| MIR100 | MicroRNA 100 | RNA Gene |  | 30 | GC11M122152 | 7.746480942 |
| SOD2 | Superoxide Dismutase 2 | Protein Coding | P04179 | 60 | GC06M159669 | 7.662748814 |
| MIR17 | MicroRNA 17 | RNA Gene |  | 28 | GC13P091350 | 7.596123219 |
| NOS1 | Nitric Oxide Synthase 1 | Protein Coding | P29475 | 62 | GC12M117208 | 7.579146385 |
| NRG1 | Neuregulin 1 | Protein Coding | Q02297 | 62 | GC08P031639 | 7.572542667 |
| XIST | X Inactive Specific Transcript | RNA Gene |  | 33 | GC0XM073820 | 7.532829762 |
| B2M | Beta-2-Microglobulin | Protein Coding | P61769 | 63 | GC15P044711 | 7.486343861 |
| NRAS | NRAS Proto-Oncogene, GTPase | Protein Coding | P01111 | 63 | GC01M114704 | 7.381304264 |
| KCNQ1OT1 | KCNQ1 Opposite Strand/Antisense Transcript 1 | RNA Gene |  | 36 | GC11M012603 | 7.380053997 |
| RASSF1 | Ras Association Domain Family Member 1 | Protein Coding | Q9NS23 | 56 | GC03M050329 | 7.310554981 |
| ENG | Endoglin | Protein Coding | P17813 | 60 | GC09M128107 | 7.283957005 |
| BRCA1 | BRCA1 DNA Repair Associated | Protein Coding | P38398 | 63 | GC17M043044 | 7.252216816 |
| GHRL | Ghrelin And Obestatin Prepropeptide | Protein Coding | Q9UBU3 | 55 | GC03M010285 | 7.250573158 |
| ARHGAP45 | Rho GTPase Activating Protein 45 | Protein Coding | Q92619 | 49 | GC19P001065 | 7.226342201 |
| MIR203A | MicroRNA 203a | RNA Gene |  | 29 | GC14P116969 | 7.219092846 |
| MIR320A | MicroRNA 320a | RNA Gene |  | 31 | GC08M022339 | 7.201840878 |
| TRAF6 | TNF Receptor Associated Factor 6 | Protein Coding | Q9Y4K3 | 59 | GC11M036467 | 7.19925642 |
| MIR149 | MicroRNA 149 | RNA Gene |  | 32 | GC02P240456 | 7.192047596 |
| LMNA | Lamin A/C | Protein Coding | P02545 | 62 | GC01P156082 | 7.138942242 |
| PGR-AS1 | PGR Antisense RNA 1 | RNA Gene |  | 20 | GC11P101131 | 7.137010098 |
| JUN | Jun Proto-Oncogene, AP-1 Transcription Factor Subunit | Protein Coding | P05412 | 63 | GC01M058780 | 7.126149178 |
| CYP1B1 | Cytochrome P450 Family 1 Subfamily B Member 1 | Protein Coding | Q16678 | 60 | GC02M038066 | 7.094403267 |
| CD59 | CD59 Molecule (CD59 Blood Group) | Protein Coding | P13987 | 59 | GC11M033724 | 7.077324867 |
| PTCH1 | Patched 1 | Protein Coding | Q13635 | 64 | GC09M095442 | 7.07473278 |
| TNIP1 | TNFAIP3 Interacting Protein 1 | Protein Coding | Q15025 | 51 | GC05M151029 | 7.033548355 |
| MIR34C | MicroRNA 34c | RNA Gene |  | 31 | GC11P112585 | 7.031018734 |
| HLA-DPB1 | Major Histocompatibility Complex, Class II, DP Beta 1 | Protein Coding | P04440 | 57 | GC06P144802 | 7.015036106 |
| MIR139 | MicroRNA 139 | RNA Gene |  | 30 | GC11M072615 | 6.991724014 |
| ACE2 | Angiotensin Converting Enzyme 2 | Protein Coding | Q9BYF1 | 62 | GC0XM015494 | 6.97638464 |
| MIR30A | MicroRNA 30a | RNA Gene |  | 28 | GC06M071403 | 6.971922874 |
| MIR182 | MicroRNA 182 | RNA Gene |  | 31 | GC07M129770 | 6.93815279 |
| TJP1 | Tight Junction Protein 1 | Protein Coding | Q07157 | 58 | GC15M029699 | 6.896519184 |
| MAPK1 | Mitogen-Activated Protein Kinase 1 | Protein Coding | P28482 | 66 | GC22M021759 | 6.878757477 |
| CLDN4 | Claudin 4 | Protein Coding | O14493 | 54 | GC07P073799 | 6.83749342 |
| MIR122 | MicroRNA 122 | RNA Gene |  | 30 | GC18P058451 | 6.828871727 |
| IL2RB | Interleukin 2 Receptor Subunit Beta | Protein Coding | P14784 | 63 | GC22M079329 | 6.82353878 |
| BIRC5 | Baculoviral IAP Repeat Containing 5 | Protein Coding | O15392 | 59 | GC17P078214 | 6.818388462 |
| CYP3A5 | Cytochrome P450 Family 3 Subfamily A Member 5 | Protein Coding | P20815 | 58 | GC07M099648 | 6.81297636 |
| PVT1 | Pvt1 Oncogene | RNA Gene |  | 35 | GC08P128175 | 6.741714001 |
| CTNNA1 | Catenin Alpha 1 | Protein Coding | P35221 | 59 | GC05P138710 | 6.735626698 |
| VIM | Vimentin | Protein Coding | P08670 | 64 | GC10P017227 | 6.65597868 |
| AGK | Acylglycerol Kinase | Protein Coding | Q53H12 | 53 | GC07P141551 | 6.648553371 |
| MIR22 | MicroRNA 22 | RNA Gene |  | 31 | GC17M001713 | 6.633424759 |
| THBD | Thrombomodulin | Protein Coding | P07204 | 59 | GC20M023026 | 6.630064011 |
| SMAD5-AS1 | SMAD5 Antisense RNA 1 | RNA Gene | Q9Y6J3 | 31 | GC05M136129 | 6.614343643 |
| TF | Transferrin | Protein Coding | P02787 | 64 | GC03P140082 | 6.581365108 |
| MT-CO1 | Mitochondrially Encoded Cytochrome C Oxidase I | Protein Coding | P00395 | 44 | GCMTP005906 | 6.538571835 |
| SLC17A5 | Solute Carrier Family 17 Member 5 | Protein Coding | Q9NRA2 | 58 | GC06M073593 | 6.52614069 |
| REN | Renin | Protein Coding | P00797 | 62 | GC01M204154 | 6.517397404 |
| MIR200A | MicroRNA 200a | RNA Gene |  | 29 | GC01P050633 | 6.51402235 |
| PLAU | Plasminogen Activator, Urokinase | Protein Coding | P00749 | 64 | GC10P073909 | 6.509222984 |
| TCF7L2 | Transcription Factor 7 Like 2 | Protein Coding | Q9NQB0 | 59 | GC10P112950 | 6.488698483 |
| DPP4 | Dipeptidyl Peptidase 4 | Protein Coding | P27487 | 62 | GC02M161992 | 6.463314533 |
| MIRLET7E | MicroRNA Let-7e | RNA Gene |  | 29 | GC19P123544 | 6.442446709 |
| WRN | WRN RecQ Like Helicase | Protein Coding | Q14191 | 60 | GC08P031033 | 6.4172616 |
| MDM2 | MDM2 Proto-Oncogene | Protein Coding | Q00987 | 67 | GC12P068808 | 6.41129446 |
| SLC46A1 | Solute Carrier Family 46 Member 1 | Protein Coding | Q96NT5 | 55 | GC17M084697 | 6.368622303 |
| ATF6 | Activating Transcription Factor 6 | Protein Coding | P18850 | 61 | GC01P161766 | 6.359892368 |
| NORAD | Non-Coding RNA Activated By DNA Damage | RNA Gene |  | 27 | GC20M036534 | 6.355011463 |
| STIM1 | Stromal Interaction Molecule 1 | Protein Coding | Q13586 | 62 | GC11P012431 | 6.339412689 |
| CCN2 | Cellular Communication Network Factor 2 | Protein Coding | P29279 | 59 | GC06M131948 | 6.327870369 |
| DNAH8 | Dynein Axonemal Heavy Chain 8 | Protein Coding | Q96JB1 | 51 | GC06P144930 | 6.285526276 |
| AOC1 | Amine Oxidase Copper Containing 1 | Protein Coding | P19801 | 54 | GC07P150824 | 6.271960258 |
| CD81 | CD81 Molecule | Protein Coding | P60033 | 59 | GC11P012372 | 6.259961128 |
| KLRK1 | Killer Cell Lectin Like Receptor K1 | Protein Coding | P26718 | 53 | GC12M030833 | 6.219133377 |
| LPCAT1 | Lysophosphatidylcholine Acyltransferase 1 | Protein Coding | Q8NF37 | 47 | GC05M001456 | 6.213470459 |
| POLE | DNA Polymerase Epsilon, Catalytic Subunit | Protein Coding | Q07864 | 60 | GC12M132789 | 6.177342892 |
| LRRK2 | Leucine Rich Repeat Kinase 2 | Protein Coding | Q5S007 | 62 | GC12P040196 | 6.131605625 |
| MIR494 | MicroRNA 494 | RNA Gene |  | 26 | GC14P117924 | 6.124019623 |
| MIR200B | MicroRNA 200b | RNA Gene |  | 30 | GC01P001167 | 6.117475033 |
| FABP2 | Fatty Acid Binding Protein 2 | Protein Coding | P12104 | 54 | GC04M119317 | 6.106635094 |
| SOAT1 | Sterol O-Acyltransferase 1 | Protein Coding | P35610 | 55 | GC01P179294 | 6.089148521 |
| ADIPOQ | Adiponectin, C1Q And Collagen Domain Containing | Protein Coding | Q15848 | 59 | GC03P186842 | 6.083623409 |
| TNFRSF4 | TNF Receptor Superfamily Member 4 | Protein Coding | P43489 | 56 | GC01M001211 | 6.083039284 |
| ASAH1 | N-Acylsphingosine Amidohydrolase 1 | Protein Coding | Q13510 | 62 | GC08M018055 | 6.082335472 |
| ATR | ATR Serine/Threonine Kinase | Protein Coding | Q13535 | 66 | GC03M142449 | 6.045758247 |
| RASSF5 | Ras Association Domain Family Member 5 | Protein Coding | Q8WWW0 | 46 | GC01P206507 | 6.016881943 |
| MIR19B1 | MicroRNA 19b-1 | RNA Gene |  | 27 | GC13P091696 | 6.002723694 |
| HERC2 | HECT And RLD Domain Containing E3 Ubiquitin Protein Ligase 2 | Protein Coding | O95714 | 57 | GC15M028111 | 5.997935772 |
| MFGE8 | Milk Fat Globule EGF And Factor V/VIII Domain Containing | Protein Coding | Q08431 | 55 | GC15M088898 | 5.979328632 |
| PTH | Parathyroid Hormone | Protein Coding | P01270 | 58 | GC11M013492 | 5.977647305 |
| CDKAL1 | CDK5 Regulatory Subunit Associated Protein 1 Like 1 | Protein Coding | Q5VV42 | 52 | GC06P020534 | 5.902732849 |
| PCAT1 | Prostate Cancer Associated Transcript 1 | RNA Gene |  | 27 | GC08P126553 | 5.899341106 |
| NOX1 | NADPH Oxidase 1 | Protein Coding | Q9Y5S8 | 56 | GC0XM100843 | 5.889604092 |
| ARPC2 | Actin Related Protein 2/3 Complex Subunit 2 | Protein Coding | O15144 | 53 | GC02P218217 | 5.850379467 |
| CHEK1 | Checkpoint Kinase 1 | Protein Coding | O14757 | 65 | GC11P125625 | 5.779563427 |
| MIR20A | MicroRNA 20a | RNA Gene |  | 29 | GC13P091699 | 5.775420666 |
| MIR150 | MicroRNA 150 | RNA Gene |  | 31 | GC19M049500 | 5.775303364 |
| MIRLET7B | MicroRNA Let-7b | RNA Gene |  | 29 | GC22P076285 | 5.760120392 |
| MIR222 | MicroRNA 222 | RNA Gene |  | 29 | GC0XM045747 | 5.737962246 |
| HSPA1L | Heat Shock Protein Family A (Hsp70) Member 1 Like | Protein Coding | P34931 | 55 | GC06M031809 | 5.703730106 |
| TNFRSF11B | TNF Receptor Superfamily Member 11b | Protein Coding | O00300 | 60 | GC08M118923 | 5.699372768 |
| MIR375 | MicroRNA 375 | RNA Gene |  | 29 | GC02M219001 | 5.628431797 |
| RAD51C | RAD51 Paralog C | Protein Coding | O43502 | 55 | GC17P058692 | 5.609739304 |
| PSMB8 | Proteasome 20S Subunit Beta 8 | Protein Coding | P28062 | 63 | GC06M032840 | 5.541259289 |
| TMSB4X | Thymosin Beta 4 X-Linked | Protein Coding | P62328 | 54 | GC0XP012975 | 5.537482738 |
| KRT1 | Keratin 1 | Protein Coding | P04264 | 60 | GC12M052674 | 5.527695656 |
| TRPV1 | Transient Receptor Potential Cation Channel Subfamily V Member 1 | Protein Coding | Q8NER1 | 61 | GC17M003565 | 5.510600567 |
| MIR30B | MicroRNA 30b | RNA Gene |  | 31 | GC08M134800 | 5.506058216 |
| DMD | Dystrophin | Protein Coding | P11532 | 58 | GC0XM031097 | 5.498627663 |
| AMACR | Alpha-Methylacyl-CoA Racemase | Protein Coding | Q9UHK6 | 58 | GC05M033986 | 5.495136738 |
| CRH | Corticotropin Releasing Hormone | Protein Coding | P06850 | 56 | GC08M066176 | 5.49175024 |
| MSH3 | MutS Homolog 3 | Protein Coding | P20585 | 55 | GC05P080654 | 5.475678921 |
| MIR144 | MicroRNA 144 | RNA Gene |  | 27 | GC17M084703 | 5.475346565 |
| MIR335 | MicroRNA 335 | RNA Gene |  | 29 | GC07P130496 | 5.453534126 |
| CAVIN1 | Caveolae Associated Protein 1 | Protein Coding | Q6NZI2 | 54 | GC17M085222 | 5.427858353 |
| MIR25 | MicroRNA 25 | RNA Gene |  | 29 | GC07M100093 | 5.426195621 |
| MIR499A | MicroRNA 499a | RNA Gene |  | 31 | GC20P034990 | 5.423052311 |
| HSPA1B | Heat Shock Protein Family A (Hsp70) Member 1B | Protein Coding | P0DMV9 | 52 | GC06P144780 | 5.40290451 |
| MIR424 | MicroRNA 424 | RNA Gene |  | 28 | GC0XM134881 | 5.391791821 |
| SQSTM1 | Sequestosome 1 | Protein Coding | Q13501 | 62 | GC05P179806 | 5.365958214 |
| AURKA | Aurora Kinase A | Protein Coding | O14965 | 65 | GC20M056370 | 5.359686852 |
| ELN | Elastin | Protein Coding | P15502 | 56 | GC07P074027 | 5.302668095 |
| KRT14 | Keratin 14 | Protein Coding | P02533 | 60 | GC17M041582 | 5.289113522 |
| MIR378A | MicroRNA 378a | RNA Gene |  | 31 | GC05P149732 | 5.285923958 |
| FADS2 | Fatty Acid Desaturase 2 | Protein Coding | O95864 | 54 | GC11P061792 | 5.285039902 |
| MAP2K2 | Mitogen-Activated Protein Kinase Kinase 2 | Protein Coding | P36507 | 67 | GC19M004090 | 5.279511452 |
| NR1H2 | Nuclear Receptor Subfamily 1 Group H Member 2 | Protein Coding | P55055 | 58 | GC19P050329 | 5.268876076 |
| MIR106B | MicroRNA 106b | RNA Gene |  | 31 | GC07M105744 | 5.22484827 |
| PDGFRL | Platelet Derived Growth Factor Receptor Like | Protein Coding | Q15198 | 54 | GC08P017576 | 5.221282482 |
| ADAD1 | Adenosine Deaminase Domain Containing 1 | Protein Coding | Q96M93 | 46 | GC04P122378 | 5.210790157 |
| CAV3 | Caveolin 3 | Protein Coding | P56539 | 56 | GC03P008733 | 5.19572401 |
| DAGLB | Diacylglycerol Lipase Beta | Protein Coding | Q8NCG7 | 46 | GC07M006416 | 5.194620609 |
| WNT4 | Wnt Family Member 4 | Protein Coding | P56705 | 60 | GC01M022190 | 5.166879177 |
| BLOC1S6 | Biogenesis Of Lysosomal Organelles Complex 1 Subunit 6 | Protein Coding | Q9UL45 | 50 | GC15P166255 | 5.159993649 |
| RC3H1 | Ring Finger And CCCH-Type Domains 1 | Protein Coding | Q5TC82 | 47 | GC01M173931 | 5.159436226 |
| LSP1 | Lymphocyte Specific Protein 1 | Protein Coding | P33241 | 51 | GC11P012308 | 5.157267094 |
| ARFGAP1 | ADP Ribosylation Factor GTPase Activating Protein 1 | Protein Coding | Q8N6T3 | 50 | GC20P063272 | 5.151195526 |
| ITGB4 | Integrin Subunit Beta 4 | Protein Coding | P16144 | 62 | GC17P075721 | 5.150372982 |
| MIR98 | MicroRNA 98 | RNA Gene |  | 28 | GC0XM053836 | 5.144683361 |
| ROCK2 | Rho Associated Coiled-Coil Containing Protein Kinase 2 | Protein Coding | O75116 | 58 | GC02M011507 | 5.125463009 |
| RMRP | RNA Component Of Mitochondrial RNA Processing Endoribonuclease | RNA Gene |  | 33 | GC09M035655 | 5.104451656 |
| ITPA | Inosine Triphosphatase | Protein Coding | Q9BY32 | 60 | GC20P008974 | 5.072163582 |
| PSMG2 | Proteasome Assembly Chaperone 2 | Protein Coding | Q969U7 | 50 | GC18P022442 | 5.054184437 |
| EZH2 | Enhancer Of Zeste 2 Polycomb Repressive Complex 2 Subunit | Protein Coding | Q15910 | 66 | GC07M148807 | 5.053070068 |
| MIR132 | MicroRNA 132 | RNA Gene |  | 31 | GC17M002049 | 5.03853035 |
| MAPT | Microtubule Associated Protein Tau | Protein Coding | P10636 | 63 | GC17P045894 | 5.019593239 |
| KRT5 | Keratin 5 | Protein Coding | P13647 | 59 | GC12M052514 | 5.013188839 |
| GJB2 | Gap Junction Protein Beta 2 | Protein Coding | P29033 | 58 | GC13M020187 | 5.007484913 |
| S1PR1 | Sphingosine-1-Phosphate Receptor 1 | Protein Coding | P21453 | 59 | GC01P101236 | 5.00047636 |
| GPX1 | Glutathione Peroxidase 1 | Protein Coding | P07203 | 56 | GC03M054007 | 4.98193121 |
| CCAT1 | Colon Cancer Associated Transcript 1 | RNA Gene |  | 23 | GC08M127207 | 4.973554611 |
| TNPO3 | Transportin 3 | Protein Coding | Q9Y5L0 | 54 | GC07M128954 | 4.9662323 |
| PRKDC | Protein Kinase, DNA-Activated, Catalytic Subunit | Protein Coding | P78527 | 63 | GC08M047773 | 4.964407921 |
| HOTTIP | HOXA Distal Transcript Antisense RNA | RNA Gene |  | 31 | GC07P027198 | 4.956602573 |
| MIR129-2 | MicroRNA 129-2 | RNA Gene |  | 27 | GC11P044042 | 4.924829006 |
| SLC15A1 | Solute Carrier Family 15 Member 1 | Protein Coding | P46059 | 54 | GC13M098683 | 4.917834759 |
| LEPR | Leptin Receptor | Protein Coding | P48357 | 63 | GC01P065444 | 4.904980183 |
| ARID1A | AT-Rich Interaction Domain 1A | Protein Coding | O14497 | 58 | GC01P026693 | 4.897803307 |
| NR1H4 | Nuclear Receptor Subfamily 1 Group H Member 4 | Protein Coding | Q96RI1 | 61 | GC12P100473 | 4.895965099 |
| HSP90AA1 | Heat Shock Protein 90 Alpha Family Class A Member 1 | Protein Coding | P07900 | 64 | GC14M102080 | 4.884851933 |
| CEP43 | Centrosomal Protein 43 | Protein Coding | O95684 | 51 | GC06P167025 | 4.87035656 |
| MT-ND1 | Mitochondrially Encoded NADH:Ubiquinone Oxidoreductase Core Subunit 1 | Protein Coding | P03886 | 45 | GCMTP003309 | 4.866453648 |
| PTK2 | Protein Tyrosine Kinase 2 | Protein Coding | Q05397 | 61 | GC08M140657 | 4.857784271 |
| BRIP1 | BRCA1 Interacting Helicase 1 | Protein Coding | Q9BX63 | 62 | GC17M061679 | 4.847064495 |
| KLF6 | KLF Transcription Factor 6 | Protein Coding | Q99612 | 56 | GC10M003779 | 4.823646545 |
| TWIST1 | Twist Family BHLH Transcription Factor 1 | Protein Coding | Q15672 | 59 | GC07M019020 | 4.819087505 |
| PLAUR | Plasminogen Activator, Urokinase Receptor | Protein Coding | Q03405 | 57 | GC19M043646 | 4.816606045 |
| SP1 | Sp1 Transcription Factor | Protein Coding | P08047 | 59 | GC12P053380 | 4.81248951 |
| SCG5 | Secretogranin V | Protein Coding | P05408 | 51 | GC15P032641 | 4.812236309 |
| PIGA | Phosphatidylinositol Glycan Anchor Biosynthesis Class A | Protein Coding | P37287 | 56 | GC0XM015319 | 4.797023773 |
| ADRA2A | Adrenoceptor Alpha 2A | Protein Coding | P08913 | 59 | GC10P111077 | 4.78638649 |
| FBXO11 | F-Box Protein 11 | Protein Coding | Q86XK2 | 55 | GC02M047789 | 4.776887417 |
| CD247 | CD247 Molecule | Protein Coding | P20963 | 62 | GC01M167399 | 4.765953064 |
| CYCS | Cytochrome C, Somatic | Protein Coding | P99999 | 61 | GC07M025118 | 4.759790897 |
| CTPS1 | CTP Synthase 1 | Protein Coding | P17812 | 58 | GC01P040979 | 4.745196819 |
| TMPRSS2 | Transmembrane Serine Protease 2 | Protein Coding | O15393 | 60 | GC21M041464 | 4.739105701 |
| CDK2 | Cyclin Dependent Kinase 2 | Protein Coding | P24941 | 64 | GC12P055966 | 4.735438347 |
| CAV2 | Caveolin 2 | Protein Coding | P51636 | 55 | GC07P116287 | 4.734286308 |
| TPM1 | Tropomyosin 1 | Protein Coding | P09493 | 61 | GC15P174366 | 4.728956223 |
| PIK3C3 | Phosphatidylinositol 3-Kinase Catalytic Subunit Type 3 | Protein Coding | Q8NEB9 | 62 | GC18P041955 | 4.726626396 |
| TYMS | Thymidylate Synthetase | Protein Coding | P04818 | 63 | GC18P000657 | 4.698867321 |
| TPD52L2 | TPD52 Like 2 | Protein Coding | O43399 | 48 | GC20P063865 | 4.695768833 |
| DANCR | Differentiation Antagonizing Non-Protein Coding RNA | RNA Gene | P0C864 | 32 | GC04P052712 | 4.692399025 |
| SLC6A14 | Solute Carrier Family 6 Member 14 | Protein Coding | Q9UN76 | 52 | GC0XP116436 | 4.689187527 |
| MIR27B | MicroRNA 27b | RNA Gene |  | 30 | GC09P102158 | 4.67797184 |
| IL16 | Interleukin 16 | Protein Coding | Q14005 | 54 | GC15P081159 | 4.674601078 |
| GZMA | Granzyme A | Protein Coding | P12544 | 55 | GC05P055102 | 4.672510624 |
| GBA1 | Glucosylceramidase Beta 1 | Protein Coding | P04062 | 63 | GC01M163977 | 4.67026329 |
| UHRF1 | Ubiquitin Like With PHD And Ring Finger Domains 1 | Protein Coding | Q96T88 | 52 | GC19P122016 | 4.651114464 |
| ZMIZ1 | Zinc Finger MIZ-Type Containing 1 | Protein Coding | Q9ULJ6 | 54 | GC10P079068 | 4.627260208 |
| MIR133B | MicroRNA 133b | RNA Gene |  | 31 | GC06P052148 | 4.625761032 |
| HSD11B1 | Hydroxysteroid 11-Beta Dehydrogenase 1 | Protein Coding | P28845 | 63 | GC01P209686 | 4.625038624 |
| KRT16 | Keratin 16 | Protein Coding | P08779 | 55 | GC17M041609 | 4.61426115 |
| MIR26A1 | MicroRNA 26a-1 | RNA Gene |  | 30 | GC03P037969 | 4.609630585 |
| ACVR1B | Activin A Receptor Type 1B | Protein Coding | P36896 | 59 | GC12P051951 | 4.606390476 |
| TXNIP | Thioredoxin Interacting Protein | Protein Coding | Q9H3M7 | 52 | GC01M145992 | 4.601480484 |
| MIR196A2 | MicroRNA 196a-2 | RNA Gene |  | 30 | GC12P061922 | 4.598503113 |
| MIR10A | MicroRNA 10a | RNA Gene |  | 30 | GC17M048579 | 4.581055641 |
| CD3E | CD3 Epsilon Subunit Of T-Cell Receptor Complex | Protein Coding | P07766 | 60 | GC11P118304 | 4.575366974 |
| NBN | Nibrin | Protein Coding | O60934 | 62 | GC08M089933 | 4.548735619 |
| MIR96 | MicroRNA 96 | RNA Gene |  | 30 | GC07M129774 | 4.541570663 |
| MIR148B | MicroRNA 148b | RNA Gene |  | 30 | GC12P054337 | 4.529978752 |
| HPRT1 | Hypoxanthine Phosphoribosyltransferase 1 | Protein Coding | P00492 | 61 | GC0XP134460 | 4.506791592 |
| SNORD15A | Small Nucleolar RNA, C/D Box 15A | RNA Gene |  | 24 | GC11P075400 | 4.505130768 |
| ROCR | Regulator Of Chondrogenesis RNA | RNA Gene |  | 22 | GC17M087858 | 4.485127449 |
| ZGPAT | Zinc Finger CCCH-Type And G-Patch Domain Containing | Protein Coding | Q8N5A5 | 47 | GC20P063707 | 4.483445168 |
| LGALS4 | Galectin 4 | Protein Coding | P56470 | 51 | GC19M096847 | 4.481950283 |
| INPP5E | Inositol Polyphosphate-5-Phosphatase E | Protein Coding | Q9NRR6 | 54 | GC09M136428 | 4.474435329 |
| CD69 | CD69 Molecule | Protein Coding | Q07108 | 55 | GC12M030826 | 4.437759399 |
| HSPA1A | Heat Shock Protein Family A (Hsp70) Member 1A | Protein Coding | P0DMV8 | 59 | GC06P144781 | 4.404420853 |
| BBC3 | BCL2 Binding Component 3 | Protein Coding | Q96PG8 | 53 | GC19M047220 | 4.395507336 |
| DOCK2 | Dedicator Of Cytokinesis 2 | Protein Coding | Q92608 | 56 | GC05P169637 | 4.393435001 |
| ADSL | Adenylosuccinate Lyase | Protein Coding | P30566 | 59 | GC22P040346 | 4.375697613 |
| PLEC | Plectin | Protein Coding | Q15149 | 56 | GC08M146785 | 4.366947174 |
| IL13RA2 | Interleukin 13 Receptor Subunit Alpha 2 | Protein Coding | Q14627 | 50 | GC0XM115003 | 4.362024307 |
| ERN1 | Endoplasmic Reticulum To Nucleus Signaling 1 | Protein Coding | O75460 | 60 | GC17M064039 | 4.354185581 |
| SLX1A-SULT1A3 | SLX1A-SULT1A3 Readthrough (NMD Candidate) | RNA Gene |  | 21 | GC16P096698 | 4.34882164 |
| RSPO3 | R-Spondin 3 | Protein Coding | Q9BXY4 | 51 | GC06P127118 | 4.348752499 |
| MMEL1 | Membrane Metalloendopeptidase Like 1 | Protein Coding | Q495T6 | 50 | GC01M002590 | 4.348350048 |
| YAP1 | Yes1 Associated Transcriptional Regulator | Protein Coding | P46937 | 60 | GC11P102110 | 4.326133728 |
| GMPPB | GDP-Mannose Pyrophosphorylase B | Protein Coding | Q9Y5P6 | 56 | GC03M049716 | 4.322422981 |
| RXRA | Retinoid X Receptor Alpha | Protein Coding | P19793 | 61 | GC09P134317 | 4.317754745 |
| MIR124-1 | MicroRNA 124-1 | RNA Gene |  | 28 | GC08M009903 | 4.305415154 |
| MIR205 | MicroRNA 205 | RNA Gene |  | 29 | GC01P209432 | 4.305106163 |
| MAD2L1 | Mitotic Arrest Deficient 2 Like 1 | Protein Coding | Q13257 | 56 | GC04M120055 | 4.300173759 |
| MIR202 | MicroRNA 202 | RNA Gene |  | 28 | GC10M133247 | 4.28869772 |
| SDHC | Succinate Dehydrogenase Complex Subunit C | Protein Coding | Q99643 | 55 | GC01P161314 | 4.285631657 |
| ENO1 | Enolase 1 | Protein Coding | P06733 | 60 | GC01M008861 | 4.262537003 |
| DBP | D-Box Binding PAR BZIP Transcription Factor | Protein Coding | Q10586 | 50 | GC19M048630 | 4.247955322 |
| PRPF8 | Pre-MRNA Processing Factor 8 | Protein Coding | Q6P2Q9 | 55 | GC17M001650 | 4.246107101 |
| MDM4 | MDM4 Regulator Of P53 | Protein Coding | O15151 | 59 | GC01P204516 | 4.232895374 |
| MIRLET7D | MicroRNA Let-7d | RNA Gene |  | 31 | GC09P094178 | 4.226500034 |
| CASR | Calcium Sensing Receptor | Protein Coding | P41180 | 64 | GC03P122183 | 4.219123363 |
| APEX1 | Apurinic/Apyrimidinic Endodeoxyribonuclease 1 | Protein Coding | P27695 | 58 | GC14P020455 | 4.199731827 |
| EMSLR | E2F1 MRNA Stabilizing LncRNA | RNA Gene |  | 21 | GC07P107551 | 4.172954082 |
| CXCL16 | C-X-C Motif Chemokine Ligand 16 | Protein Coding | Q9H2A7 | 51 | GC17M004733 | 4.168676376 |
| MIR7-3HG | MIR7-3 Host Gene | RNA Gene | Q8N6C7 | 35 | GC19P122009 | 4.166033745 |
| LRP6 | LDL Receptor Related Protein 6 | Protein Coding | O75581 | 60 | GC12M030861 | 4.144235611 |
| PTGES | Prostaglandin E Synthase | Protein Coding | O14684 | 54 | GC09M129738 | 4.126418591 |
| SREBF1 | Sterol Regulatory Element Binding Transcription Factor 1 | Protein Coding | P36956 | 62 | GC17M017810 | 4.093165874 |
| DHFR | Dihydrofolate Reductase | Protein Coding | P00374 | 61 | GC05M080626 | 4.08351326 |
| LOC110806262 | Solute Carrier Family 6 Member 4 Gene Promoter | Functional Element |  | 8 | GC17P030235 | 4.07681942 |
| ENTPD1 | Ectonucleoside Triphosphate Diphosphohydrolase 1 | Protein Coding | P49961 | 63 | GC10P112752 | 4.067348957 |
| TG | Thyroglobulin | Protein Coding | P01266 | 58 | GC08P132866 | 4.061672211 |
| SEPTIN9 | Septin 9 | Protein Coding | Q9UHD8 | 53 | GC17P123942 | 4.060350895 |
| MIR15B | MicroRNA 15b | RNA Gene |  | 29 | GC03P160404 | 4.052937984 |
| MXI1 | MAX Interactor 1, Dimerization Protein | Protein Coding | P50539 | 54 | GC10P113338 | 4.03650713 |
| EHMT2 | Euchromatic Histone Lysine Methyltransferase 2 | Protein Coding | Q96KQ7 | 58 | GC06M031879 | 4.031734943 |
| RARG | Retinoic Acid Receptor Gamma | Protein Coding | P13631 | 59 | GC12M053210 | 4.024292946 |
| CYP21A2 | Cytochrome P450 Family 21 Subfamily A Member 2 | Protein Coding | P08686 | 56 | GC06P144787 | 4.019051075 |
| MIR339 | MicroRNA 339 | RNA Gene |  | 30 | GC07M001022 | 4.01698637 |
| PTX3 | Pentraxin 3 | Protein Coding | P26022 | 56 | GC03P157436 | 4.015251637 |
| BIRC2 | Baculoviral IAP Repeat Containing 2 | Protein Coding | Q13490 | 60 | GC11P102347 | 4.004019737 |
| CHD4 | Chromodomain Helicase DNA Binding Protein 4 | Protein Coding | Q14839 | 58 | GC12M006570 | 3.973890781 |
| ASH1L | ASH1 Like Histone Lysine Methyltransferase | Protein Coding | Q9NR48 | 52 | GC01M155335 | 3.969344378 |
| FLOT1 | Flotillin 1 | Protein Coding | O75955 | 54 | GC06M097031 | 3.954855919 |
| FNBP1 | Formin Binding Protein 1 | Protein Coding | Q96RU3 | 47 | GC09M129887 | 3.952322483 |
| FOXO3 | Forkhead Box O3 | Protein Coding | O43524 | 60 | GC06P108559 | 3.938471794 |
| PNMT | Phenylethanolamine N-Methyltransferase | Protein Coding | P11086 | 56 | GC17P039667 | 3.925286293 |
| FSCN1 | Fascin Actin-Bundling Protein 1 | Protein Coding | Q16658 | 56 | GC07P005592 | 3.913449287 |
| MIR137 | MicroRNA 137 | RNA Gene |  | 27 | GC01M098046 | 3.905108213 |
| CALB2 | Calbindin 2 | Protein Coding | P22676 | 52 | GC16P071358 | 3.90404129 |
| MIR181A2 | MicroRNA 181a-2 | RNA Gene |  | 29 | GC09P124692 | 3.890251875 |
| CX3CR1 | C-X3-C Motif Chemokine Receptor 1 | Protein Coding | P49238 | 56 | GC03M039279 | 3.883578777 |
| CS | Citrate Synthase | Protein Coding | O75390 | 58 | GC12M056271 | 3.880432606 |
| MMUT | Methylmalonyl-CoA Mutase | Protein Coding | P22033 | 58 | GC06M049430 | 3.878669024 |
| ELAVL1 | ELAV Like RNA Binding Protein 1 | Protein Coding | Q15717 | 55 | GC19M007958 | 3.877356529 |
| DAXX | Death Domain Associated Protein | Protein Coding | Q9UER7 | 57 | GC06M033318 | 3.874592066 |
| USF1 | Upstream Transcription Factor 1 | Protein Coding | P22415 | 56 | GC01M161039 | 3.872061491 |
| IP6K2 | Inositol Hexakisphosphate Kinase 2 | Protein Coding | Q9UHH9 | 51 | GC03M048688 | 3.865800381 |
| IP6K1 | Inositol Hexakisphosphate Kinase 1 | Protein Coding | Q92551 | 47 | GC03M054021 | 3.865800381 |
| PMS1 | PMS1 Homolog 1, Mismatch Repair System Component | Protein Coding | P54277 | 52 | GC02P189784 | 3.865634918 |
| HSPA8 | Heat Shock Protein Family A (Hsp70) Member 8 | Protein Coding | P11142 | 62 | GC11M123057 | 3.857289791 |
| MYBPC3 | Myosin Binding Protein C3 | Protein Coding | Q14896 | 59 | GC11M128621 | 3.855966091 |
| MIR372 | MicroRNA 372 | RNA Gene |  | 25 | GC19P123653 | 3.855907917 |
| MDH2 | Malate Dehydrogenase 2 | Protein Coding | P40926 | 60 | GC07P076048 | 3.849444389 |
| SMPD1 | Sphingomyelin Phosphodiesterase 1 | Protein Coding | P17405 | 62 | GC11P006390 | 3.840783358 |
| RPS20 | Ribosomal Protein S20 | Protein Coding | P60866 | 55 | GC08M056067 | 3.824366093 |
| FTCD | Formimidoyltransferase Cyclodeaminase | Protein Coding | O95954 | 54 | GC21M053911 | 3.819073677 |
| HDAC1 | Histone Deacetylase 1 | Protein Coding | Q13547 | 63 | GC01P032292 | 3.807756901 |
| SCYL1 | SCY1 Like Pseudokinase 1 | Protein Coding | Q96KG9 | 53 | GC11P065525 | 3.803748131 |
| GTF2H4 | General Transcription Factor IIH Subunit 4 | Protein Coding | Q92759 | 48 | GC06P144745 | 3.802469015 |
| ITIH4 | Inter-Alpha-Trypsin Inhibitor Heavy Chain 4 | Protein Coding | Q14624 | 55 | GC03M052812 | 3.801840305 |
| CRIPTO | Cripto, EGF-CFC Family Member | Protein Coding | P13385 | 53 | GC03P060225 | 3.801143408 |
| ALPP | Alkaline Phosphatase, Placental | Protein Coding | P05187 | 56 | GC02P232378 | 3.798722506 |
| CYP2E1 | Cytochrome P450 Family 2 Subfamily E Member 1 | Protein Coding | P05181 | 59 | GC10P133520 | 3.792725325 |
| TBXT | T-Box Transcription Factor T | Protein Coding | O15178 | 58 | GC06M166158 | 3.778933525 |
| FKRP | Fukutin Related Protein | Protein Coding | Q9H9S5 | 52 | GC19P123289 | 3.763158798 |
| NPPB | Natriuretic Peptide B | Protein Coding | P16860 | 56 | GC01M015389 | 3.755672455 |
| NTHL1 | Nth Like DNA Glycosylase 1 | Protein Coding | P78549 | 56 | GC16M025583 | 3.749827623 |
| RAD54B | RAD54 Homolog B | Protein Coding | Q9Y620 | 52 | GC08M094371 | 3.747154713 |
| VTI1A | Vesicle Transport Through Interaction With T-SNAREs 1A | Protein Coding | Q96AJ9 | 50 | GC10P112446 | 3.747154713 |
| APP | Amyloid Beta Precursor Protein | Protein Coding | P05067 | 63 | GC21M025880 | 3.737985849 |
| NCAPD2 | Non-SMC Condensin I Complex Subunit D2 | Protein Coding | Q15021 | 53 | GC12P006493 | 3.728709698 |
| AGPAT1 | 1-Acylglycerol-3-Phosphate O-Acyltransferase 1 | Protein Coding | Q99943 | 49 | GC06M032168 | 3.728709698 |
| SERINC3 | Serine Incorporator 3 | Protein Coding | Q13530 | 50 | GC20M044496 | 3.723659515 |
| PRXL2B | Peroxiredoxin Like 2B | Protein Coding | Q8TBF2 | 36 | GC01P050703 | 3.723659515 |
| RPL18 | Ribosomal Protein L18 | Protein Coding | Q07020 | 55 | GC19M048615 | 3.721200943 |
| NFKBIZ | NFKB Inhibitor Zeta | Protein Coding | Q9BYH8 | 50 | GC03P101827 | 3.71978569 |
| MIR183 | MicroRNA 183 | RNA Gene |  | 27 | GC07M129887 | 3.696265221 |
| TUFM | Tu Translation Elongation Factor, Mitochondrial | Protein Coding | P49411 | 57 | GC16M044746 | 3.688824177 |
| SNHG6 | Small Nucleolar RNA Host Gene 6 | RNA Gene |  | 27 | GC08M066921 | 3.680557728 |
| ANGPTL4 | Angiopoietin Like 4 | Protein Coding | Q9BY76 | 57 | GC19P008363 | 3.665689945 |
| CDK1 | Cyclin Dependent Kinase 1 | Protein Coding | P06493 | 61 | GC10P060772 | 3.662815094 |
| GPX2 | Glutathione Peroxidase 2 | Protein Coding | P18283 | 54 | GC14M064939 | 3.662101269 |
| TPO | Thyroid Peroxidase | Protein Coding | P07202 | 60 | GC02P001374 | 3.635061502 |
| LMAN1 | Lectin, Mannose Binding 1 | Protein Coding | P49257 | 58 | GC18M059327 | 3.618419647 |
| PTPN12 | Protein Tyrosine Phosphatase Non-Receptor Type 12 | Protein Coding | Q05209 | 60 | GC07P077537 | 3.612566471 |
| DLC1 | DLC1 Rho GTPase Activating Protein | Protein Coding | Q96QB1 | 56 | GC08M013083 | 3.612566471 |
| TFDP1 | Transcription Factor Dp-1 | Protein Coding | Q14186 | 56 | GC13P113584 | 3.612566471 |
| EIF3E | Eukaryotic Translation Initiation Factor 3 Subunit E | Protein Coding | P60228 | 51 | GC08M108163 | 3.612566471 |
| AR | Androgen Receptor | Protein Coding | P10275 | 64 | GC0XP067544 | 3.610403776 |
| FAM136A | Family With Sequence Similarity 136 Member A | Protein Coding | Q96C01 | 47 | GC02M070296 | 3.607213974 |
| PSMD4 | Proteasome 26S Subunit Ubiquitin Receptor, Non-ATPase 4 | Protein Coding | P55036 | 58 | GC01P167002 | 3.603289604 |
| PGK1 | Phosphoglycerate Kinase 1 | Protein Coding | P00558 | 62 | GC0XP078190 | 3.592503071 |
| SLC30A8 | Solute Carrier Family 30 Member 8 | Protein Coding | Q8IWU4 | 54 | GC08P116950 | 3.576778412 |
| CD38 | CD38 Molecule | Protein Coding | P28907 | 59 | GC04P025740 | 3.571622133 |
| SCARNA5 | Small Cajal Body-Specific RNA 5 | RNA Gene |  | 28 | GC02P233275 | 3.57018137 |
| MIR506 | MicroRNA 506 | RNA Gene |  | 24 | GC0XM147230 | 3.561445951 |
| PTGER2 | Prostaglandin E Receptor 2 | Protein Coding | P43116 | 61 | GC14P052314 | 3.549271584 |
| GGH | Gamma-Glutamyl Hydrolase | Protein Coding | Q92820 | 54 | GC08M063014 | 3.548501015 |
| GAL | Galanin And GMAP Prepropeptide | Protein Coding | P22466 | 58 | GC11P094859 | 3.545581818 |
| MRPS18C | Mitochondrial Ribosomal Protein S18C | Protein Coding | Q9Y3D5 | 43 | GC04P083455 | 3.540816784 |
| PPP1R14A | Protein Phosphatase 1 Regulatory Inhibitor Subunit 14A | Protein Coding | Q96A00 | 52 | GC19M038251 | 3.524955511 |
| CALR | Calreticulin | Protein Coding | P27797 | 64 | GC19P012938 | 3.505910397 |
| EMG1 | EMG1 N1-Specific Pseudouridine Methyltransferase | Protein Coding | Q92979 | 52 | GC12P006970 | 3.50475502 |
| TH | Tyrosine Hydroxylase | Protein Coding | P07101 | 63 | GC11M002163 | 3.503865719 |
| GFM1 | G Elongation Factor Mitochondrial 1 | Protein Coding | Q96RP9 | 56 | GC03P158644 | 3.494514942 |
| SLC9A9 | Solute Carrier Family 9 Member A9 | Protein Coding | Q8IVB4 | 50 | GC03M143265 | 3.493786812 |
| ASH2L | ASH2 Like, Histone Lysine Methyltransferase Complex Subunit | Protein Coding | Q9UBL3 | 54 | GC08P040499 | 3.483495712 |
| FLVCR1 | FLVCR Choline And Heme Transporter 1 | Protein Coding | Q9Y5Y0 | 52 | GC01P212858 | 3.473257542 |
| MIR196B | MicroRNA 196b | RNA Gene |  | 30 | GC07M028050 | 3.456671476 |
| USP25 | Ubiquitin Specific Peptidase 25 | Protein Coding | Q9UHP3 | 48 | GC21P018976 | 3.455016613 |
| MIR373 | MicroRNA 373 | RNA Gene |  | 29 | GC19P123654 | 3.453791618 |
| DNMT3A | DNA Methyltransferase 3 Alpha | Protein Coding | Q9Y6K1 | 64 | GC02M025228 | 3.453365803 |
| UMPS | Uridine Monophosphate Synthetase | Protein Coding | P11172 | 59 | GC03P124730 | 3.442776918 |
| KIR2DL2 | Killer Cell Immunoglobulin Like Receptor, Two Ig Domains And Long Cytoplasmic Tail 2 | Protein Coding | P43627 | 33 | GC19MR00107 | 3.436852217 |
| OLFM4 | Olfactomedin 4 | Protein Coding | Q6UX06 | 51 | GC13P053028 | 3.431859493 |
| BRD4 | Bromodomain Containing 4 | Protein Coding | O60885 | 60 | GC19M096358 | 3.428458452 |
| TRC-GCA24-1 | TRNA-Cys (GCA) 24-1 | RNA Gene |  | 14 | GC17M085080 | 3.424909353 |
| GRB2 | Growth Factor Receptor Bound Protein 2 | Protein Coding | P62993 | 59 | GC17M075318 | 3.406744242 |
| NUDT15 | Nudix Hydrolase 15 | Protein Coding | Q9NV35 | 48 | GC13P048037 | 3.405153751 |
| E2F4 | E2F Transcription Factor 4 | Protein Coding | Q16254 | 56 | GC16P067192 | 3.394183636 |
| MARVELD2 | MARVEL Domain Containing 2 | Protein Coding | Q8N4S9 | 51 | GC05P069415 | 3.381653309 |
| CORO1A | Coronin 1A | Protein Coding | P31146 | 56 | GC16P096692 | 3.378637791 |
| SMOX | Spermine Oxidase | Protein Coding | Q9NWM0 | 48 | GC20P004120 | 3.361418247 |
| SLIT2 | Slit Guidance Ligand 2 | Protein Coding | O94813 | 59 | GC04P025805 | 3.355959892 |
| FOXO1 | Forkhead Box O1 | Protein Coding | Q12778 | 64 | GC13M040555 | 3.34630394 |
| ABCB7 | ATP Binding Cassette Subfamily B Member 7 | Protein Coding | O75027 | 57 | GC0XM075053 | 3.333144426 |
| ITGA5 | Integrin Subunit Alpha 5 | Protein Coding | P08648 | 60 | GC12M056062 | 3.329005241 |
| PPA2 | Inorganic Pyrophosphatase 2 | Protein Coding | Q9H2U2 | 52 | GC04M105369 | 3.327268124 |
| PRKAA1 | Protein Kinase AMP-Activated Catalytic Subunit Alpha 1 | Protein Coding | Q13131 | 60 | GC05M040759 | 3.320580006 |
| ZNF365 | Zinc Finger Protein 365 | Protein Coding | Q70YC4 | 49 | GC10P062374 | 3.318027496 |
| CD47 | CD47 Molecule | Protein Coding | Q08722 | 58 | GC03M108043 | 3.302467346 |
| CHMP4B | Charged Multivesicular Body Protein 4B | Protein Coding | Q9H444 | 55 | GC20P042272 | 3.295909405 |
| GAS7 | Growth Arrest Specific 7 | Protein Coding | O60861 | 48 | GC17M009910 | 3.286138535 |
| PSD | Pleckstrin And Sec7 Domain Containing | Protein Coding | A5PKW4 | 47 | GC10M102403 | 3.286138535 |
| PLK1 | Polo Like Kinase 1 | Protein Coding | P53350 | 64 | GC16P096393 | 3.28538847 |
| MIR429 | MicroRNA 429 | RNA Gene |  | 30 | GC01P050634 | 3.275951862 |
| CDKN3 | Cyclin Dependent Kinase Inhibitor 3 | Protein Coding | Q16667 | 54 | GC14P054401 | 3.270617485 |
| DYSF | Dysferlin | Protein Coding | O75923 | 56 | GC02P071453 | 3.267763138 |
| KHDRBS3 | KH RNA Binding Domain Containing, Signal Transduction Associated 3 | Protein Coding | O75525 | 48 | GC08P135457 | 3.255485773 |
| PLEK | Pleckstrin | Protein Coding | P08567 | 53 | GC02P068365 | 3.253903866 |
| NRBP1 | Nuclear Receptor Binding Protein 1 | Protein Coding | Q9UHY1 | 44 | GC02P027427 | 3.246470213 |
| BCYRN1 | Brain Cytoplasmic RNA 1 | RNA Gene |  | 28 | GC02P047253 | 3.244198322 |
| FAAH | Fatty Acid Amide Hydrolase | Protein Coding | O00519 | 61 | GC01P046394 | 3.241624594 |
| LNPEP | Leucyl And Cystinyl Aminopeptidase | Protein Coding | Q9UIQ6 | 58 | GC05P096935 | 3.23533988 |
| TNFRSF17 | TNF Receptor Superfamily Member 17 | Protein Coding | Q02223 | 59 | GC16P011965 | 3.23187542 |
| FLNC | Filamin C | Protein Coding | Q14315 | 58 | GC07P128830 | 3.230881929 |
| C1QBP | Complement C1q Binding Protein | Protein Coding | Q07021 | 58 | GC17M005432 | 3.186659575 |
| MRPS2 | Mitochondrial Ribosomal Protein S2 | Protein Coding | Q9Y399 | 50 | GC09P135499 | 3.182499886 |
| MRPS14 | Mitochondrial Ribosomal Protein S14 | Protein Coding | O60783 | 47 | GC01M175010 | 3.182499886 |
| MRPL51 | Mitochondrial Ribosomal Protein L51 | Protein Coding | Q4U2R6 | 40 | GC12M006491 | 3.182499886 |
| HSP90B1 | Heat Shock Protein 90 Beta Family Member 1 | Protein Coding | P14625 | 60 | GC12P103930 | 3.17865777 |
| GALT | Galactose-1-Phosphate Uridylyltransferase | Protein Coding | P07902 | 59 | GC09P058537 | 3.164712191 |
| ALDOB | Aldolase, Fructose-Bisphosphate B | Protein Coding | P05062 | 59 | GC09M101420 | 3.151045799 |
| NEU1 | Neuraminidase 1 | Protein Coding | Q99519 | 59 | GC06M031857 | 3.139737368 |
| BLZF1 | Basic Leucine Zipper Nuclear Factor 1 | Protein Coding | Q9H2G9 | 50 | GC01P169367 | 3.139042854 |
| TRPA1 | Transient Receptor Potential Cation Channel Subfamily A Member 1 | Protein Coding | O75762 | 60 | GC08M072019 | 3.134680748 |
| RPS6KB1 | Ribosomal Protein S6 Kinase B1 | Protein Coding | P23443 | 63 | GC17P059893 | 3.134039164 |
| ABCB8 | ATP Binding Cassette Subfamily B Member 8 | Protein Coding | Q9NUT2 | 51 | GC07P151028 | 3.1328969 |
| XRCC5 | X-Ray Repair Cross Complementing 5 | Protein Coding | P13010 | 57 | GC02P216107 | 3.132394791 |
| PANX1 | Pannexin 1 | Protein Coding | Q96RD7 | 59 | GC11P094128 | 3.131813526 |
| VCL | Vinculin | Protein Coding | P18206 | 60 | GC10P073995 | 3.127968788 |
| HDAC3 | Histone Deacetylase 3 | Protein Coding | O15379 | 62 | GC05M141620 | 3.120598793 |
| HCG26 | HLA Complex Group 26 | RNA Gene |  | 15 | GC06P144757 | 3.119697571 |
| SLC15A4 | Solute Carrier Family 15 Member 4 | Protein Coding | Q8N697 | 48 | GC12M128793 | 3.119546413 |
| HIPK2 | Homeodomain Interacting Protein Kinase 2 | Protein Coding | Q9H2X6 | 54 | GC07M139561 | 3.112567902 |
| SMAD6 | SMAD Family Member 6 | Protein Coding | O43541 | 59 | GC15P066702 | 3.102557421 |
| TOM1 | Target Of Myb1 Membrane Trafficking Protein | Protein Coding | O60784 | 51 | GC22P035299 | 3.099502563 |
| LITAF | Lipopolysaccharide Induced TNF Factor | Protein Coding | Q99732 | 56 | GC16M011547 | 3.090706587 |
| CNOT11 | CCR4-NOT Transcription Complex Subunit 11 | Protein Coding | Q9UKZ1 | 43 | GC02P101252 | 3.086484671 |
| ST7-OT3 | ST7 Overlapping Transcript 3 | RNA Gene |  | 20 | GC07P117217 | 3.079598427 |
| CXCL6 | C-X-C Motif Chemokine Ligand 6 | Protein Coding | P80162 | 51 | GC04P073837 | 3.076937437 |
| RNF5 | Ring Finger Protein 5 | Protein Coding | Q99942 | 51 | GC06P144791 | 3.076608658 |
| CTDP1 | CTD Phosphatase Subunit 1 | Protein Coding | Q9Y5B0 | 54 | GC18P079679 | 3.075481892 |
| MIR7-1 | MicroRNA 7-1 | RNA Gene |  | 25 | GC09M111066 | 3.073349476 |
| CHMP5 | Charged Multivesicular Body Protein 5 | Protein Coding | Q9NZZ3 | 48 | GC09P033264 | 3.058410883 |
| OXTR | Oxytocin Receptor | Protein Coding | P30559 | 60 | GC03M008767 | 3.040810823 |
| SETDB1 | SET Domain Bifurcated Histone Lysine Methyltransferase 1 | Protein Coding | Q15047 | 55 | GC01P150926 | 3.040233374 |
| FKTN | Fukutin | Protein Coding | O75072 | 53 | GC09P105558 | 3.038587093 |
| NSD1 | Nuclear Receptor Binding SET Domain Protein 1 | Protein Coding | Q96L73 | 56 | GC05P187580 | 3.032556295 |
| PRDX1 | Peroxiredoxin 1 | Protein Coding | Q06830 | 62 | GC01M045790 | 3.016492128 |
| MIR124-3 | MicroRNA 124-3 | RNA Gene |  | 25 | GC20P067529 | 3.008645535 |
| PRDM16 | PR/SET Domain 16 | Protein Coding | Q9HAZ2 | 58 | GC01P050708 | 3.003304958 |
| ILK | Integrin Linked Kinase | Protein Coding | Q13418 | 58 | GC11P012587 | 2.984707594 |
| TAOK1 | TAO Kinase 1 | Protein Coding | Q7L7X3 | 56 | GC17P122522 | 2.978166342 |
| FOLR1 | Folate Receptor Alpha | Protein Coding | P15328 | 62 | GC11P095015 | 2.975356102 |
| THBS1 | Thrombospondin 1 | Protein Coding | P07996 | 59 | GC15P039581 | 2.969722033 |
| IKBKE | Inhibitor Of Nuclear Factor Kappa B Kinase Subunit Epsilon | Protein Coding | Q14164 | 58 | GC01P206470 | 2.963046551 |
| BAP1 | BRCA1 Associated Protein 1 | Protein Coding | Q92560 | 60 | GC03M052401 | 2.946632385 |
| KMT2D | Lysine Methyltransferase 2D | Protein Coding | O14686 | 56 | GC12M049018 | 2.944785833 |
| GTF2E2 | General Transcription Factor IIE Subunit 2 | Protein Coding | P29084 | 54 | GC08M030578 | 2.930494547 |
| MRPL37 | Mitochondrial Ribosomal Protein L37 | Protein Coding | Q9BZE1 | 45 | GC01P058066 | 2.929131985 |
| MRPL16 | Mitochondrial Ribosomal Protein L16 | Protein Coding | Q9NX20 | 42 | GC11M128745 | 2.929131985 |
| CBFA2T3 | CBFA2/RUNX1 Partner Transcriptional Co-Repressor 3 | Protein Coding | O75081 | 52 | GC16M088874 | 2.90131855 |
| CBX3 | Chromobox 3 | Protein Coding | Q13185 | 55 | GC07P026201 | 2.900650501 |
| NDUFAF1 | NADH:Ubiquinone Oxidoreductase Complex Assembly Factor 1 | Protein Coding | Q9Y375 | 54 | GC15M041387 | 2.886967897 |
| CLOCK | Clock Circadian Regulator | Protein Coding | O15516 | 57 | GC04M055427 | 2.874657393 |
| FH | Fumarate Hydratase | Protein Coding | P07954 | 59 | GC01M241499 | 2.862384319 |
| PNPO | Pyridoxamine 5'-Phosphate Oxidase | Protein Coding | Q9NVS9 | 59 | GC17P047941 | 2.852978706 |
| POLG | DNA Polymerase Gamma, Catalytic Subunit | Protein Coding | P54098 | 60 | GC15M152330 | 2.85024786 |
| ERCC2 | ERCC Excision Repair 2, TFIIH Core Complex Helicase Subunit | Protein Coding | P18074 | 61 | GC19M045349 | 2.843824387 |
| GJB6 | Gap Junction Protein Beta 6 | Protein Coding | O95452 | 55 | GC13M020221 | 2.842281818 |
| PHB2 | Prohibitin 2 | Protein Coding | Q99623 | 54 | GC12M006965 | 2.820870876 |
| CYSLTR1 | Cysteinyl Leukotriene Receptor 1 | Protein Coding | Q9Y271 | 55 | GC0XM078271 | 2.820651054 |
| HSF1 | Heat Shock Transcription Factor 1 | Protein Coding | Q00613 | 59 | GC08P144291 | 2.817540169 |
| BECN1 | Beclin 1 | Protein Coding | Q14457 | 59 | GC17M042810 | 2.809975863 |
| MT-CO2 | Mitochondrially Encoded Cytochrome C Oxidase II | Protein Coding | P00403 | 47 | GCMTP007587 | 2.809867382 |
| ATP5PD | ATP Synthase Peripheral Stalk Subunit D | Protein Coding | O75947 | 48 | GC17M086187 | 2.807975531 |
| TRIM8 | Tripartite Motif Containing 8 | Protein Coding | Q9BZR9 | 50 | GC10P113132 | 2.802046537 |
| ATXN2L | Ataxin 2 Like | Protein Coding | Q8WWM7 | 51 | GC16P096572 | 2.793322086 |
| SQOR | Sulfide Quinone Oxidoreductase | Protein Coding | Q9Y6N5 | 50 | GC15P166257 | 2.793279409 |
| CCNE1 | Cyclin E1 | Protein Coding | P24864 | 62 | GC19P029811 | 2.79094553 |
| SLC39A12 | Solute Carrier Family 39 Member 12 | Protein Coding | Q504Y0 | 47 | GC10P017951 | 2.775977135 |
| INTS11 | Integrator Complex Subunit 11 | Protein Coding | Q5TA45 | 50 | GC01M015013 | 2.771476269 |
| RPL3 | Ribosomal Protein L3 | Protein Coding | P39023 | 53 | GC22M079350 | 2.757341862 |
| VPS4B | Vacuolar Protein Sorting 4 Homolog B | Protein Coding | O75351 | 48 | GC18M063389 | 2.747812271 |
| KDM6A | Lysine Demethylase 6A | Protein Coding | O15550 | 60 | GC0XP044873 | 2.746124744 |
| CGN | Cingulin | Protein Coding | Q9P2M7 | 47 | GC01P167016 | 2.73212719 |
| SLC2A10 | Solute Carrier Family 2 Member 10 | Protein Coding | O95528 | 55 | GC20P046709 | 2.730019808 |
| XPO1 | Exportin 1 | Protein Coding | O14980 | 61 | GC02M061445 | 2.729278088 |
| ATF3 | Activating Transcription Factor 3 | Protein Coding | P18847 | 58 | GC01P212565 | 2.721870899 |
| MIP | Major Intrinsic Protein Of Lens Fiber | Protein Coding | P30301 | 52 | GC12M056449 | 2.719802856 |
| EXO1 | Exonuclease 1 | Protein Coding | Q9UQ84 | 56 | GC01P241847 | 2.719220638 |
| PLXNB1 | Plexin B1 | Protein Coding | O43157 | 50 | GC03M048403 | 2.715437174 |
| DNM1L | Dynamin 1 Like | Protein Coding | O00429 | 61 | GC12P032679 | 2.704446793 |
| PMPCA | Peptidase, Mitochondrial Processing Subunit Alpha | Protein Coding | Q10713 | 56 | GC09P136410 | 2.692551613 |
| NUSAP1 | Nucleolar And Spindle Associated Protein 1 | Protein Coding | Q9BXS6 | 44 | GC15P166126 | 2.692551613 |
| CPEB4 | Cytoplasmic Polyadenylation Element Binding Protein 4 | Protein Coding | Q17RY0 | 43 | GC05P173888 | 2.692551613 |
| TTYH3 | Tweety Family Member 3 | Protein Coding | Q9C0H2 | 40 | GC07P013716 | 2.692551613 |
| SUZ12 | SUZ12 Polycomb Repressive Complex 2 Subunit | Protein Coding | Q15022 | 55 | GC17P031937 | 2.688112974 |
| DLEU2 | Deleted In Lymphocytic Leukemia 2 | RNA Gene |  | 31 | GC13M049913 | 2.683011055 |
| RPS14 | Ribosomal Protein S14 | Protein Coding | P62263 | 54 | GC05M150443 | 2.681888342 |
| LDLR | Low Density Lipoprotein Receptor | Protein Coding | P01130 | 64 | GC19P122311 | 2.681244612 |
| AKT2 | AKT Serine/Threonine Kinase 2 | Protein Coding | P31751 | 67 | GC19M040230 | 2.671184778 |
| NELFCD | Negative Elongation Factor Complex Member C/D | Protein Coding | Q8IXH7 | 50 | GC20P058981 | 2.66993022 |
| MIR501 | MicroRNA 501 | RNA Gene |  | 26 | GC0XP058463 | 2.663314104 |
| GOLM1 | Golgi Membrane Protein 1 | Protein Coding | Q8NBJ4 | 53 | GC09M086026 | 2.660188675 |
| MSTN | Myostatin | Protein Coding | O14793 | 59 | GC02M190055 | 2.65923357 |
| MIR125B1 | MicroRNA 125b-1 | RNA Gene |  | 31 | GC11M130263 | 2.64607954 |
| KMT2C | Lysine Methyltransferase 2C | Protein Coding | Q8NEZ4 | 56 | GC07M152134 | 2.642482996 |
| MIR532 | MicroRNA 532 | RNA Gene |  | 25 | GC0XP058460 | 2.628273487 |
| VPS35L | VPS35 Endosomal Protein Sorting Factor Like | Protein Coding | Q7Z3J2 | 43 | GC16P019555 | 2.608879805 |
| RPL10 | Ribosomal Protein L10 | Protein Coding | P27635 | 56 | GC0XP154389 | 2.607691526 |
| POP4 | POP4 Homolog, Ribonuclease P/MRP Subunit | Protein Coding | O95707 | 50 | GC19P122842 | 2.598862886 |
| DDIT4 | DNA Damage Inducible Transcript 4 | Protein Coding | Q9NX09 | 54 | GC10P072273 | 2.593822479 |
| MAP1LC3A | Microtubule Associated Protein 1 Light Chain 3 Alpha | Protein Coding | Q9H492 | 54 | GC20P034546 | 2.591877699 |
| BDH2 | 3-Hydroxybutyrate Dehydrogenase 2 | Protein Coding | Q9BUT1 | 41 | GC04M103077 | 2.589694977 |
| TATDN1 | TatD DNase Domain Containing 1 | Protein Coding | Q6P1N9 | 43 | GC08M124488 | 2.589532375 |
| PNP | Purine Nucleoside Phosphorylase | Protein Coding | P00491 | 62 | GC14P047738 | 2.574589491 |
| CARMN | Cardiac Mesoderm Enhancer-Associated Non-Coding RNA | RNA Gene |  | 25 | GC05P149397 | 2.562933683 |
| HOXA2 | Homeobox A2 | Protein Coding | O43364 | 52 | GC07M027100 | 2.555747509 |
| FUT4 | Fucosyltransferase 4 | Protein Coding | P22083 | 52 | GC11P096335 | 2.552587509 |
| MANBA | Mannosidase Beta | Protein Coding | O00462 | 55 | GC04M102631 | 2.550487995 |
| PCK2 | Phosphoenolpyruvate Carboxykinase 2, Mitochondrial | Protein Coding | Q16822 | 59 | GC14P024094 | 2.550222158 |
| ACVR2A | Activin A Receptor Type 2A | Protein Coding | P27037 | 58 | GC02P147844 | 2.547921181 |
| ADM | Adrenomedullin | Protein Coding | P35318 | 57 | GC11P012779 | 2.546958923 |
| CDC37 | Cell Division Cycle 37, HSP90 Cochaperone | Protein Coding | Q16543 | 56 | GC19M010391 | 2.543747902 |
| EIF2AK2 | Eukaryotic Translation Initiation Factor 2 Alpha Kinase 2 | Protein Coding | P19525 | 63 | GC02M037099 | 2.543130875 |
| EHBP1 | EH Domain Binding Protein 1 | Protein Coding | Q8NDI1 | 52 | GC02P062673 | 2.542559147 |
| MPIG6B | Megakaryocyte And Platelet Inhibitory Receptor G6b | Protein Coding | O95866 | 51 | GC06P153189 | 2.538079977 |
| SNCA | Synuclein Alpha | Protein Coding | P37840 | 64 | GC04M089724 | 2.535499811 |
| CYP17A1 | Cytochrome P450 Family 17 Subfamily A Member 1 | Protein Coding | P05093 | 62 | GC10M102830 | 2.526318312 |
| ACP5 | Acid Phosphatase 5, Tartrate Resistant | Protein Coding | P13686 | 59 | GC19M011574 | 2.525518894 |
| FLAD1 | Flavin Adenine Dinucleotide Synthetase 1 | Protein Coding | Q8NFF5 | 53 | GC01P154983 | 2.522984982 |
| ABI1 | Abl Interactor 1 | Protein Coding | Q8IZP0 | 54 | GC10M026746 | 2.509703875 |
| INSR | Insulin Receptor | Protein Coding | P06213 | 67 | GC19M007112 | 2.50952363 |
| FIP1L1 | Factor Interacting With PAPOLA And CPSF1 | Protein Coding | Q6UN15 | 52 | GC04P053543 | 2.50661993 |
| POLR1H | RNA Polymerase I Subunit H | Protein Coding | Q9P1U0 | 45 | GC06P155679 | 2.506497145 |
| SLC26A6 | Solute Carrier Family 26 Member 6 | Protein Coding | Q9BXS9 | 51 | GC03M048625 | 2.504556417 |
| ATG10 | Autophagy Related 10 | Protein Coding | Q9H0Y0 | 52 | GC05P081972 | 2.502809048 |
| LRCH1 | Leucine Rich Repeats And Calponin Homology Domain Containing 1 | Protein Coding | Q9Y2L9 | 46 | GC13P046553 | 2.502809048 |
| DLAT | Dihydrolipoamide S-Acetyltransferase | Protein Coding | P10515 | 59 | GC11P112608 | 2.502627373 |
| NHERF1 | NHERF Family PDZ Scaffold Protein 1 | Protein Coding | O14745 | 57 | GC17P123825 | 2.484158516 |
| SELENOS | Selenoprotein S | Protein Coding | Q9BQE4 | 47 | GC15M152459 | 2.479492903 |
| CCNB1 | Cyclin B1 | Protein Coding | P14635 | 60 | GC05P069167 | 2.475667477 |
| RAD51 | RAD51 Recombinase | Protein Coding | Q06609 | 63 | GC15P040694 | 2.474745274 |
| C1GALT1 | Core 1 Synthase, Glycoprotein-N-Acetylgalactosamine 3-Beta-Galactosyltransferase 1 | Protein Coding | Q9NS00 | 52 | GC07P007156 | 2.474466085 |
| SLC26A4 | Solute Carrier Family 26 Member 4 | Protein Coding | O43511 | 56 | GC07P107660 | 2.473800898 |
| MIR16-1 | MicroRNA 16-1 | RNA Gene |  | 29 | GC13M050048 | 2.458698988 |
| CSNK2A1 | Casein Kinase 2 Alpha 1 | Protein Coding | P68400 | 63 | GC20M000472 | 2.454205036 |
| PTK2B | Protein Tyrosine Kinase 2 Beta | Protein Coding | Q14289 | 63 | GC08P027311 | 2.444667578 |
| RIT1 | Ras Like Without CAAX 1 | Protein Coding | Q92963 | 59 | GC01M155897 | 2.439183235 |
| FIBP | FGF1 Intracellular Binding Protein | Protein Coding | O43427 | 54 | GC11M129037 | 2.439183235 |
| NDFIP1 | Nedd4 Family Interacting Protein 1 | Protein Coding | Q9BT67 | 43 | GC05P142108 | 2.439183235 |
| SFMBT1 | Scm Like With Four Mbt Domains 1 | Protein Coding | Q9UHJ3 | 43 | GC03M054180 | 2.439183235 |
| CBLL1 | Cbl Proto-Oncogene Like 1 | Protein Coding | Q75N03 | 46 | GC07P107743 | 2.424970388 |
| PRG2 | Proteoglycan 2, Pro Eosinophil Major Basic Protein | Protein Coding | P13727 | 52 | GC11M057386 | 2.41790247 |
| MOCS2 | Molybdenum Cofactor Synthesis 2 | Protein Coding | O96033 | 55 | GC05M053095 | 2.417195797 |
| SERGEF | Secretion Regulating Guanine Nucleotide Exchange Factor | Protein Coding | Q9UGK8 | 45 | GC11M017994 | 2.41393137 |
| TNK2 | Tyrosine Kinase Non Receptor 2 | Protein Coding | Q07912 | 60 | GC03M195863 | 2.40631628 |
| DPH5 | Diphthamide Biosynthesis 5 | Protein Coding | Q9H2P9 | 45 | GC01M100989 | 2.401838064 |
| PRSS1 | Serine Protease 1 | Protein Coding | P07477 | 57 | GC07P159683 | 2.395483017 |
| ZBTB12 | Zinc Finger And BTB Domain Containing 12 | Protein Coding | Q9Y330 | 39 | GC06M031899 | 2.391336203 |
| CBFA2T2 | CBFA2/RUNX1 Partner Transcriptional Co-Repressor 2 | Protein Coding | O43439 | 46 | GC20P033490 | 2.37923193 |
| OGA | O-GlcNAcase | Protein Coding | O60502 | 54 | GC10M101785 | 2.378879309 |
| RNF25 | Ring Finger Protein 25 | Protein Coding | Q96BH1 | 45 | GC02M218663 | 2.376213074 |
| PRKAB1 | Protein Kinase AMP-Activated Non-Catalytic Subunit Beta 1 | Protein Coding | Q9Y478 | 60 | GC12P119667 | 2.373634338 |
| TBK1 | TANK Binding Kinase 1 | Protein Coding | Q9UHD2 | 62 | GC12P064451 | 2.370123386 |
| NACA | Nascent Polypeptide Associated Complex Subunit Alpha | Protein Coding | E9PAV3 | 46 | GC12M056712 | 2.368801832 |
| IGF2BP1 | Insulin Like Growth Factor 2 MRNA Binding Protein 1 | Protein Coding | Q9NZI8 | 54 | GC17P123324 | 2.368601799 |
| SKP2 | S-Phase Kinase Associated Protein 2 | Protein Coding | Q13309 | 58 | GC05P036151 | 2.364976406 |
| MIR301A | MicroRNA 301a | RNA Gene |  | 29 | GC17M059151 | 2.362259626 |
| WDR41 | WD Repeat Domain 41 | Protein Coding | Q9HAD4 | 46 | GC05M077425 | 2.360190868 |
| VRK1 | VRK Serine/Threonine Kinase 1 | Protein Coding | Q99986 | 60 | GC14P096797 | 2.345494747 |
| MPPED2 | Metallophosphoesterase Domain Containing 2 | Protein Coding | Q15777 | 48 | GC11M030406 | 2.345494747 |
| PRRC1 | Proline Rich Coiled-Coil 1 | Protein Coding | Q96M27 | 41 | GC05P127517 | 2.345494747 |
| LHCGR | Luteinizing Hormone/Choriogonadotropin Receptor | Protein Coding | P22888 | 59 | GC02M048686 | 2.342817068 |
| WWOX | WW Domain Containing Oxidoreductase | Protein Coding | Q9NZC7 | 59 | GC16P078099 | 2.339406967 |
| ZFP91 | ZFP91 Zinc Finger Protein, Atypical E3 Ubiquitin Ligase | Protein Coding | Q96JP5 | 42 | GC11P058912 | 2.334292412 |
| GALK1 | Galactokinase 1 | Protein Coding | P51570 | 60 | GC17M075751 | 2.333852291 |
| CYP4F22 | Cytochrome P450 Family 4 Subfamily F Member 22 | Protein Coding | Q6NT55 | 47 | GC19P015508 | 2.328720093 |
| RING1 | Ring Finger Protein 1 | Protein Coding | Q06587 | 54 | GC06P033208 | 2.326722622 |
| FGF8 | Fibroblast Growth Factor 8 | Protein Coding | P55075 | 61 | GC10M101770 | 2.324841976 |
| TKT | Transketolase | Protein Coding | P29401 | 59 | GC03M053224 | 2.317010641 |
| KHDC4 | KH Domain Containing 4, Pre-MRNA Splicing Factor | Protein Coding | Q7Z7F0 | 39 | GC01M155913 | 2.309404373 |
| PRKACB | Protein Kinase CAMP-Activated Catalytic Subunit Beta | Protein Coding | P22694 | 61 | GC01P084078 | 2.308475494 |
| HIBCH | 3-Hydroxyisobutyryl-CoA Hydrolase | Protein Coding | Q6NVY1 | 54 | GC02M190189 | 2.304534674 |
| SLC9A8 | Solute Carrier Family 9 Member A8 | Protein Coding | Q9Y2E8 | 45 | GC20P050859 | 2.300979137 |
| PRICKLE1 | Prickle Planar Cell Polarity Protein 1 | Protein Coding | Q96MT3 | 58 | GC12M042456 | 2.298340082 |
| TRIM5 | Tripartite Motif Containing 5 | Protein Coding | Q9C035 | 53 | GC11M012739 | 2.288319349 |
| INHBA | Inhibin Subunit Beta A | Protein Coding | P08476 | 58 | GC07M041670 | 2.277018547 |
| DPY30 | Dpy-30 Histone Methyltransferase Complex Regulatory Subunit | Protein Coding | Q9C005 | 47 | GC02M031867 | 2.272647142 |
| RPS11 | Ribosomal Protein S11 | Protein Coding | P62280 | 51 | GC19P049496 | 2.268763304 |
| HERC1 | HECT And RLD Domain Containing E3 Ubiquitin Protein Ligase Family Member 1 | Protein Coding | Q15751 | 52 | GC15M063608 | 2.268068075 |
| AOC3 | Amine Oxidase Copper Containing 3 | Protein Coding | Q16853 | 58 | GC17P042851 | 2.258835554 |
| CXCL14 | C-X-C Motif Chemokine Ligand 14 | Protein Coding | O95715 | 48 | GC05M135617 | 2.253470898 |
| MIR500A | MicroRNA 500a | RNA Gene |  | 25 | GC0XP050008 | 2.253220797 |
| CUL4A | Cullin 4A | Protein Coding | Q13619 | 56 | GC13P113208 | 2.253015995 |
| AGFG1 | ArfGAP With FG Repeats 1 | Protein Coding | P52594 | 53 | GC02P227473 | 2.252066851 |
| VPS35 | VPS35 Retromer Complex Component | Protein Coding | Q96QK1 | 55 | GC16M047331 | 2.251391172 |
| IMPACT | Impact RWD Domain Protein | Protein Coding | Q9P2X3 | 44 | GC18P024426 | 2.245406866 |
| VPS52 | VPS52 Subunit Of GARP Complex | Protein Coding | Q8N1B4 | 44 | GC06M097202 | 2.24042201 |
| SELENBP1 | Selenium Binding Protein 1 | Protein Coding | Q13228 | 55 | GC01M151364 | 2.239574909 |
| SLC12A3 | Solute Carrier Family 12 Member 3 | Protein Coding | P55017 | 60 | GC16P056865 | 2.237003565 |
| TOP1 | DNA Topoisomerase I | Protein Coding | P11387 | 62 | GC20P041028 | 2.233114004 |
| CD151 | CD151 Molecule (Raph Blood Group) | Protein Coding | P48509 | 56 | GC11P012280 | 2.197590351 |
| GPR55 | G Protein-Coupled Receptor 55 | Protein Coding | Q9Y2T6 | 53 | GC02M230907 | 2.196365356 |
| RBM17 | RNA Binding Motif Protein 17 | Protein Coding | Q96I25 | 49 | GC10P006089 | 2.195902586 |
| GPN1 | GPN-Loop GTPase 1 | Protein Coding | Q9HCN4 | 46 | GC02P027628 | 2.195902586 |
| MED16 | Mediator Complex Subunit 16 | Protein Coding | Q9Y2X0 | 43 | GC19M095759 | 2.195902586 |
| EIPR1 | EARP Complex And GARP Complex Interacting Protein 1 | Protein Coding | Q53HC9 | 42 | GC02M003188 | 2.195902586 |
| THADA | THADA Armadillo Repeat Containing | Protein Coding | Q6YHU6 | 50 | GC02M043234 | 2.189594984 |
| CALU | Calumenin | Protein Coding | O43852 | 52 | GC07P128739 | 2.186631441 |
| MRPL28 | Mitochondrial Ribosomal Protein L28 | Protein Coding | Q13084 | 48 | GC16M000366 | 2.186631441 |
| RNASET2 | Ribonuclease T2 | Protein Coding | O00584 | 57 | GC06M166924 | 2.176194429 |
| CASP6 | Caspase 6 | Protein Coding | P55212 | 62 | GC04M109688 | 2.152180672 |
| RNF114 | Ring Finger Protein 114 | Protein Coding | Q9Y508 | 46 | GC20P049936 | 2.15179944 |
| DSC1 | Desmocollin 1 | Protein Coding | Q08554 | 50 | GC18M031129 | 2.148051023 |
| UCP2 | Uncoupling Protein 2 | Protein Coding | P55851 | 59 | GC11M073974 | 2.142276764 |
| QSOX2 | Quiescin Sulfhydryl Oxidase 2 | Protein Coding | Q6ZRP7 | 48 | GC09M136206 | 2.141982794 |
| MIR181A1 | MicroRNA 181a-1 | RNA Gene |  | 28 | GC01M198860 | 2.129945755 |
| CYTH2 | Cytohesin 2 | Protein Coding | Q99418 | 51 | GC19P123373 | 2.127993584 |
| HCG18 | HLA Complex Group 18 | RNA Gene |  | 19 | GC06M097010 | 2.127605915 |
| CSNK2A2 | Casein Kinase 2 Alpha 2 | Protein Coding | P19784 | 57 | GC16M058157 | 2.125748873 |
| COX15 | Cytochrome C Oxidase Assembly Homolog COX15 | Protein Coding | Q7KZN9 | 55 | GC10M099696 | 2.124688148 |
| KCNA3 | Potassium Voltage-Gated Channel Subfamily A Member 3 | Protein Coding | P22001 | 56 | GC01M110654 | 2.123061419 |
| MCM8 | Minichromosome Maintenance 8 Homologous Recombination Repair Factor | Protein Coding | Q9UJA3 | 52 | GC20P009061 | 2.121226072 |
| SLC25A28 | Solute Carrier Family 25 Member 28 | Protein Coding | Q96A46 | 46 | GC10M099610 | 2.111448526 |
| ABCC9 | ATP Binding Cassette Subfamily C Member 9 | Protein Coding | O60706 | 57 | GC12M021797 | 2.107385874 |
| B3GALT4 | Beta-1,3-Galactosyltransferase 4 | Protein Coding | O96024 | 52 | GC06P033277 | 2.10583353 |
| MCM4 | Minichromosome Maintenance Complex Component 4 | Protein Coding | P33991 | 60 | GC08P047970 | 2.103597641 |
| TST | Thiosulfate Sulfurtransferase | Protein Coding | Q16762 | 52 | GC22M037010 | 2.092529297 |
| EIF3C | Eukaryotic Translation Initiation Factor 3 Subunit C | Protein Coding | Q99613 | 48 | GC16P096542 | 2.092529297 |
| ABCF2 | ATP Binding Cassette Subfamily F Member 2 | Protein Coding | Q9UG63 | 51 | GC07M151211 | 2.091109991 |
| FOXP2 | Forkhead Box P2 | Protein Coding | O15409 | 56 | GC07P114086 | 2.072595835 |
| STX8 | Syntaxin 8 | Protein Coding | Q9UNK0 | 52 | GC17M009250 | 2.065957308 |
| GON4L | Gon-4 Like | Protein Coding | Q3T8J9 | 46 | GC01M163997 | 2.056035995 |
| TTC33 | Tetratricopeptide Repeat Domain 33 | Protein Coding | Q6PID6 | 41 | GC05M040512 | 2.056035995 |
| NEK9 | NIMA Related Kinase 9 | Protein Coding | Q8TD19 | 58 | GC14M075079 | 2.051166296 |
| COG5 | Component Of Oligomeric Golgi Complex 5 | Protein Coding | Q9UP83 | 52 | GC07M107201 | 2.051166296 |
| KARS1 | Lysyl-TRNA Synthetase 1 | Protein Coding | Q15046 | 58 | GC16M076029 | 2.049052 |
| CXADR | CXADR Ig-Like Cell Adhesion Molecule | Protein Coding | P78310 | 56 | GC21P017513 | 2.046202421 |
| MPDZ | Multiple PDZ Domain Crumbs Cell Polarity Complex Component | Protein Coding | O75970 | 55 | GC09M013105 | 2.043318987 |
| NRBF2 | Nuclear Receptor Binding Factor 2 | Protein Coding | Q96F24 | 43 | GC10P063133 | 2.037570238 |
| RSPH4A | Radial Spoke Head Component 4A | Protein Coding | Q5TD94 | 49 | GC06P116616 | 2.032002211 |
| STK19 | Serine/Threonine Kinase 19 | Protein Coding | P49842 | 48 | GC06P031971 | 2.032002211 |
| UCKL1 | Uridine-Cytidine Kinase 1 Like 1 | Protein Coding | Q9NWZ5 | 47 | GC20M063939 | 2.032002211 |
| ASAP2 | ArfGAP With SH3 Domain, Ankyrin Repeat And PH Domain 2 | Protein Coding | O43150 | 45 | GC02P009206 | 2.032002211 |
| RPAP3 | RNA Polymerase II Associated Protein 3 | Protein Coding | Q9H6T3 | 42 | GC12M047661 | 2.032002211 |
| ZMAT5 | Zinc Finger Matrin-Type 5 | Protein Coding | Q9UDW3 | 41 | GC22M029730 | 2.032002211 |
| TMA7 | Translation Machinery Associated 7 Homolog | Protein Coding | Q9Y2S6 | 36 | GC03P048440 | 2.032002211 |
| LAMC1 | Laminin Subunit Gamma 1 | Protein Coding | P11047 | 56 | GC01P183023 | 2.025452614 |
| COPS6 | COP9 Signalosome Subunit 6 | Protein Coding | Q7L5N1 | 50 | GC07P100088 | 2.025452614 |
| ITGA1 | Integrin Subunit Alpha 1 | Protein Coding | P56199 | 58 | GC05P052788 | 2.025183201 |
| ATP6V1F | ATPase H+ Transporting V1 Subunit F | Protein Coding | Q16864 | 52 | GC07P128862 | 2.017018318 |
| KAT6B | Lysine Acetyltransferase 6B | Protein Coding | Q8WYB5 | 57 | GC10P074985 | 2.014699697 |
| SLC39A7 | Solute Carrier Family 39 Member 7 | Protein Coding | Q92504 | 54 | GC06P033200 | 2.014699697 |
| TMEM258 | Transmembrane Protein 258 | Protein Coding | P61165 | 40 | GC11M061768 | 1.994990945 |
| STARD13 | StAR Related Lipid Transfer Domain Containing 13 | Protein Coding | Q9Y3M8 | 50 | GC13M033103 | 1.992038488 |
| CYP2A6 | Cytochrome P450 Family 2 Subfamily A Member 6 | Protein Coding | P11509 | 58 | GC19M040843 | 1.989837885 |
| BAZ1A | Bromodomain Adjacent To Zinc Finger Domain 1A | Protein Coding | Q9NRL2 | 53 | GC14M034752 | 1.982058883 |
| CCNL2 | Cyclin L2 | Protein Coding | Q96S94 | 50 | GC01M001385 | 1.978869915 |
| POLR2E | RNA Polymerase II, I And III Subunit E | Protein Coding | P19388 | 53 | GC19M001086 | 1.97484684 |
| QRICH1 | Glutamine Rich 1 | Protein Coding | Q2TAL8 | 49 | GC03M053989 | 1.97484684 |
| ABHD16A | Abhydrolase Domain Containing 16A, Phospholipase | Protein Coding | O95870 | 48 | GC06M097115 | 1.97484684 |
| SCGN | Secretagogin, EF-Hand Calcium Binding Protein | Protein Coding | O76038 | 50 | GC06P025652 | 1.974699497 |
| TTF2 | Transcription Termination Factor 2 | Protein Coding | Q9UNY4 | 53 | GC01P117060 | 1.974360466 |
| PLXDC2 | Plexin Domain Containing 2 | Protein Coding | Q6UX71 | 47 | GC10P019817 | 1.973954678 |
[truncated: 70,421 more chars]
